# Discovery of quantitative trait loci for resistance to parasitic nematode infection in sheep: I. Analysis of outcross pedigrees

**DOI:** 10.1186/1471-2164-7-178

**Published:** 2006-07-18

**Authors:** Allan M Crawford, Korena A Paterson, Ken G Dodds, Cristina Diez Tascon, Penny A Williamson, Meredith Roberts Thomson, Stewart A Bisset, Anne E Beattie, Gordon J Greer, Richard S Green, Roger Wheeler, Richard J Shaw, Kevin Knowler, John C McEwan

**Affiliations:** 1 AgResearch, Invermay Agricultural Centre, Private Bag 50034, Mosgiel, New Zealand; 2 AgResearch, Molecular Biology Unit, Department of Biochemistry, University of Otago, Box 56, Dunedin, New Zealand; 3 AgResearch, Wallaceville Animal Research Centre, PO Box 40063, Upper Hutt, New Zealand; 4 AgResearch, Woodlands, Private Bag 90121, Invercargill, New Zealand; 5 Banco de Tumores, Anatomia Patologica, Complejo Hospitalario de Leon, 24008 Leon, Spain

## Abstract

**Background:**

Currently most pastoral farmers rely on anthelmintic drenches to control gastrointestinal parasitic nematodes in sheep. Resistance to anthelmintics is rapidly increasing in nematode populations such that on some farms none of the drench families are now completely effective. It is well established that host resistance to nematode infection is a moderately heritable trait. This study was undertaken to identify regions of the genome, quantitative trait loci (QTL) that contain genes affecting resistance to parasitic nematodes.

**Results:**

Rams obtained from crossing nematode parasite resistant and susceptible selection lines were used to derive five large half-sib families comprising between 348 and 101 offspring per sire. Total offspring comprised 940 lambs. Extensive measurements for a range of parasite burden and immune function traits in all offspring allowed each lamb in each pedigree to be ranked for relative resistance to nematode parasites.

Initially the 22 most resistant and 22 most susceptible progeny from each pedigree were used in a genome scan that used 203 microsatellite markers spread across all sheep autosomes. This study identified 9 chromosomes with regions showing sufficient linkage to warrant the genotyping of all offspring. After genotyping all offspring with markers covering Chromosomes 1, 3, 4, 5, 8, 12, 13, 22 and 23, the telomeric end of chromosome 8 was identified as having a significant QTL for parasite resistance as measured by the number of *Trichostrongylus spp*. adults in the abomasum and small intestine at the end of the second parasite challenge. Two further QTL for associated immune function traits of total serum IgE and *T. colubiformis *specific serum IgG, at the end of the second parasite challenge, were identified on chromosome 23.

**Conclusion:**

Despite parasite resistance being a moderately heritable trait, this large study was able to identify only a single significant QTL associated with it. The QTL concerned adult parasite burdens at the end of the second parasite challenge when the lambs were approximately 6 months old. Our failure to discover more QTL suggests that most of the genes controlling this trait are of relatively small effect. The large number of suggestive QTL discovered (more than one per family per trait than would be expected by chance) also supports this conclusion.

## Background

Nematode parasites are the major animal health constraint in sheep production on pasture. Chemical control using anthelmintic drenches has been a reliable means of nematode control for the last 40 years but increasingly nematodes are becoming resistant to anthelmintics. A recent survey in New Zealand [1] has shown that approximately 43% and 33% of farms have nematodes resistant to benzimidazoles and macrocyclic lactones respectively. Nematodes resistant to all major classes of anthelmintics have now been documented throughout the world for the three major sheep nematode species – *Haemonchus contortus*, *Teladorsagia circumcincta *and *Trichostrongylus colubriformis *[2]. Multiply drug-resistant *H. contortus *is now making small ruminant production, in some areas of the tropics, unsustainable [3-5].

As a result of this failure of anthelmintic drenches, a major research effort has been underway for the past 15 years to examine alternatives to chemical control. The use of nematode trapping fungi [6], diets high in condensed tannins [7,8], and other plant materials [9] as well as other nutritional approaches [10,11] have all been examined as possible approaches to reduce the impact of nematode parasites in sheep.

A particularly popular nematode control method has been to select sheep which are genetically resistant to parasitic nematodes. This has the advantage of providing a long-term solution which involves challenging and phenotyping only a small proportion of the industry flocks, as the trait can be easily disseminated through the sale of resistant rams [12]. In New Zealand and Australia, lines of sheep selected for resistance and susceptibility to nematode parasites have been in existence since 1979 and 1975 respectively [13]. In addition, industry wide schemes, WormFEC in NZ [14] and NEMESIS in Australia [15], have been operating for more than a decade to help sheep breeders select for this trait.

A wide range of studies in many different sheep breeds has shown that resistance to nematode parasites is a moderately heritable trait with most heritability estimates about 0.3 [16-19]. Certain sheep breeds have also been identified as being more resistant to nematodes than others. For example the Red Massai sheep in Kenya have been shown to be more resistant to *Haemonchus *infection than the South African Dorper breed [16]. French scientists have shown that the Barbados Black Belly sheep is more resistant than the INRA401 composite breed [20].

The major method of measuring parasite burden is by counting parasite eggs in sheep faeces, the so called faecal egg count (FEC), which is an unattractive and labour intensive technique. Furthermore the parasite challenge required can affect lamb growth and other important traits such as faecal soiling around the anus (dags). These constraints have limited the uptake of parasite resistance selection by the sheep breeding industry. A proposed solution to this dilemma has been to try to identify the genes responsible for the heritable trait and offer a more attractive DNA test to help identify resistant sheep [21].

In order to identify suitable DNA markers a number of association studies have been undertaken with markers from the MHC region on sheep chromosome 20 [22-26]. Given that the development of an immune response is associated with resistance to nematodes it is not unexpected that associations have been identified with particular MHC markers or haplotypes. Most of the MHC marker associations have been in populations primarily infected with *T. circumcincta*. Markers located in the sheep gamma interferon gene on chromosome 3q have also shown significant associations with parasite resistance in Romney selection lines and wild populations of Soay sheep on the island of St Kilda off the west coast of Scotland. [27,28]

More global searches for DNA markers involving genome scans for QTL have also been undertaken. This is the fifth such study to be reported for parasite resistance in sheep. In all cases half-sib experimental pedigrees derived from a sire known to be segregating for parasite resistance were used, with the sires derived from crossing either divergent selection lines or crossing resistant and susceptible breeds of sheep. In the first study which used Merino sheep derived from crossing divergent selection lines and an artificial challenge with *Trichostronglus colubriformis *parasites no genome wide significant QTL were identified [29], although one region on chromosome 6 was significant at the chromosome wide level. In the second study Merino sheep were artificially challenged with *H. contortus *on 2 separate occasions at 6 months and 13 months of age. Four half-sib pedigrees were involved and a number of QTL were identified, but unfortunately their chromosomal location was not disclosed [30]. The third study involved the use of resistant Indonesian Thin Tail sheep crossed with susceptible Merino sheep and backcrossed to Merino sheep. Two artificial challenges with *H. contortus *larvae 12 weeks apart followed by measurement of faecal egg counts and blood packed cell volume (PCV) were used to generate and measure the parasite burden. As with the previous study, two QTL were identified but their chromosomal location was not disclosed [31].

The fourth study recently published by Davies et al [32] was the most similar to this one in that natural exposure to parasites by grazing was the method of infection. They identified QTL on chromosome 3 and 20 associated with specific IgA activity, on chromosome 2, 3 and 14 associated with *Nematodirus *FEC and on chromosome 3 and 20 associated with Strongyle FEC.

This study reports in full our linkage analysis involving 5 half-sib pedigrees in which sires derived from crossing resistant and susceptible lines of Romney sheep were out-crossed to Coopworth ewes and a total of 18 different measurements for parasite burden and associated traits were used to define the phenotype in the offspring.

## Results

### Initial genome scan results

Decreases in the nematode parasite burden, as measured by FEC and internal worm numbers (the late larval stages and adult worms in the abomasum and small intestine), were the major phenotypes used to measure parasite burden and hence resistance. Given the very high cost of developing the large pedigrees used in this experiment we also measured the humoral immune response, both IgG and IgE, to the parasite challenge, the development of dags and lamb growth rate in the hope that these associated phenotypes may also provide some insight into the genetic basis of parasite resistance. We did however concentrate on parasite burden as the primary trait of interest so that in choosing the most extreme animals for this trait for genotyping we were reducing our chances of finding QTL for the other traits examined.

Results from the initial genome scan using the 44 most extreme offspring in the five pedigrees and the FEC1 and FEC2 traits, indicated the possibility of QTL for parasite resistance on chromosomes 1q, 3, 4, 5, 8, 12, 13, 20 and 23. Subsequently all progeny were genotyped with the markers used on these eight chromosomes. Given the very large number of phenotype and genotype measurements taken the results of all the analyses undertaken are presented in additional chromosome figures with results from each of the 26 chromosomes presented in a series of 21 graphs. [Additional files 6 to 31] The first graph in the series shows the information content across the chromosome; the second and third graphs show the linkage results for all traits from all families combined; the remaining 18 graphs show within family linkage for each of the traits measured. A full list of all microsatellite markers used in the genome scan is also included as Additional Table 1. [Additional file 3].

### QTL discoveries

A total of six QTL with genome wide significance were identified and are summarised in Table 3. These results contain 4 QTL associated with parasite burden and 2 with the immune response to infection. No significant QTL were identified for growth rate or dag traits although as explained in the previous section we focussed our efforts on parasite burden in the search for QTL and it is only on those chromosomes in which all family members were genotyped where we had a good chance of discovering QTL for those traits not highly correlated with parasite burden.

The 2 most interesting QTL are those found in family 124 near the telomere on Chromosome 8. The two phenotypes are complementary in that one is a measure of *Trichostrongylus spp *adults and late stage larvae in the abomasum while the other is for the same nematode genus in the small intestine. The fact that two separate measures of parasite burden both show QTL in the same region of the genome strengthens the evidence for a QTL in this region. These two measures were moderately correlated (correlation of 0.49; P < 0.001 after adjusting for fixed effects including sire but excluding QTL effects).

The across family QTL for *Trichostrongylus spp *adults in the small intestine on chromosome 11 and the family 124 QTL for *Trichostrongylus spp *adults in the abomasum on chromosome 2 are both on chromosomes where only the most extreme animals were genotyped. In our initial genome scan we obtained similar results on the 8 chromosomes intensively genotyped and with the exception of Chromosome 8 the QTL we identified were not confirmed by the additional genotyping. There is a good chance that the chromosome 11 and 2 QTL would not be confirmed by further genotyping.

The two QTL in family 154 on Chromosome 23 are both concerned with the immune response to parasite infection measuring either total serum IgE or serum IgGspecific for the *T. colubriformis *L3 larvae. These two phenotypes have a correlation of 0.25 (P < 0.001). The QTL peaks, although approximately 30 cM apart on this small chromosome, do overlap so we are unable to determine whether the same or different genes are responsible.

As expected with a genome scan involving a large number of measured traits, many suggestive QTL were also identified. We identified 21 of these across families combined (c.f. 26 expected if there are no true QTL) and 123 suggestive QTL within families (90 expected). All the suggestive QTL are indicated by an appropriately coloured asterisk above their most favoured position on the chromosome. The six significant QTL are indicated by a double asterisk.

## Discussion

There is good evidence from a large range of studies in a variety of sheep populations, including the breeds used in this study [17], that parasite resistance is a moderately heritable trait. This indicates that genes and QTL affecting parasite resistance must exist. Unfortunately this study, involving almost 1000 sheep that were extensively phenotyped and genotyped, has only convincingly identified one QTL, on chromosome 8, for parasite resistance. The estimated size of the effect is a 2.3-fold reduction in adult worms and late stage larvae in the abomasum and small intestine in those sheep with the favourable allele, although this is likely to be an overestimate [33].

A further two QTL were identified for associated traits (specific anti- *T. colubriformis *IgG_1 _and total IgE in serum) involved in the immune response to parasite infection. Given that these two QTL are in regions without even suggestive parasite resistance QTL, it is unlikely that the genes affecting these traits directly affect parasite resistance.

It is possible that QTL are being masked by epistatic effects. The recent publication, by Carlborg et al,[34] in which epistasis models were used to find QTL with major effect on chicken growth is encouraging. The data used was from a traditional linkage study which had found no large QTL when modelling a single QTL [35]. The aim of our experiment was to find QTL that could be used in selection schemes without regard to other QTL. Although a single QTL analysis has not revealed any QTL worth pursuing for this purpose, it may be that there are sets of two or more QTL with large combined effects. Such effects could be revealed by scanning for two QTL simultaneously and we plan to undertake such an analysis. This analysis is likely to uncover only very large effects with the present experiment given the smaller subclass sizes involved and the need for stricter testing thresholds to account for the increased number of multiple tests.

The simplest explanation for the small number of identified QTL is that the genes affecting resistance are of small effect. Power calculations indicate that, at the average family size, this genome scan would have a 90% chance of detecting any QTL with an effect greater than 0.8 σ_p_. Given the very high cost of this experiment the option of trebling the size of pedigrees, so that QTL with effects as small as 0.5 σ_p _can be reliably identified, is not attractive.

A more attractive alternative is to switch discovery strategies and use linkage disequilibrium or allele association studies to find chromosomal regions and genes affecting resistance to parasites. The recent development of high-throughput systems for genotyping Single Nucleotide Polymorphisms (SNP's) [36] means that large scale allele association studies are now possible. Recent studies [37] have shown that sheep populations have high levels of linkage disequilibrium meaning that 10–20 thousand SNP markers are likely to define most of the haplotype blocks segregating in the sheep genome. Providing the sheep genomics community can generate a large number of SNP markers suitable for high-throughput genotyping, this approach is feasible. Strategies involving linkage disequilibrium analysis using large numbers of SNP's genotyped across well phenotyped unrelated sheep may be more successful than ours and others linkage studies.

## Conclusion

Despite being a moderately heritable trait this large study was only able to identify a single significant QTL for *Trichostrongylus spp*. resistance in those regions where we had chosen to genotype all the animals. The QTL concerned adult parasite burdens at the end of the second parasite challenge when lambs are approximately 6 months old. Our failure to discover more QTL suggests that most of the genes controlling this trait are of relatively small effect hence we conclude that discovery strategies using linkage analysis should be replaced by strategies using linkage disequilibrium in unrelated populations.

## Methods

### Selection lines and outcross segregation pedigrees

The Wallaceville divergent FEC selection lines of Romneys commenced in 1979 [38]. Selection is currently based on FEC levels after natural challenge, measured twice during the first 8 months of life, in both ram and ewe lambs. At present, the high line has a 40-fold higher FEC than the low line during the autumn. On a logarithmic scale this translates to a 3.6 σ_p _divergence between lines (C.A. Morris, unpublished data). Rams used as the sires of five large half-sib families (Table 1.) were derived from reciprocal crosses of the selection lines. The parents of these rams were born in either 1992 or 1993 and at this stage in the development of the selection lines there would have had an average 8-fold difference in FEC between the lines which equates to 2.6 σ_p_. Studies in these lines have shown FEC to be highly correlated (r = 0.85 – 0.91) with Trichostrongyle adult worm burden in the intestine [39]. The lambs were generated in 1993 and 1994 on two separate properties in Southland New Zealand by mating the F1 rams to unrelated Coopworth ewes, a composite breed derived from Romney and Border Leicester interbreds.

### Parasite challenges

Two natural field challenges of infective nematode larvae [38] were given to all outcross lambs. **Challenge 1 **– after weaning, lambs were drenched with 9 ml of ivermectin (Ivomec liquid, Merial New Zealand) then grazed on pasture known to be contaminated with parasite larvae. A subset of the lambs were monitored weekly by faecal sampling until the average strongyle egg count approached 1500 eggs per gram of faecal material (epg), then all lambs were measured. Three separate faecal samples were taken over 5 days and results (in epg) averaged. Faecal egg counts were determined using a modified McMaster method [40] in which each egg counted represents 50 eggs per gram of faeces. **Challenge 2 **– After all the Challenge 1 faecal samples had been taken, lambs were again drenched (13 ml, ivermectin) then returned to contaminated pasture and monitored. When the parasite burden again reached approximately 1500 epg 3 more faecal samples were taken and measured as described for the first challenge. Mean strongyle and *Nematodirus *infection levels achieved were 1091 epg (FEC1) and 70 epg (NEM1) respectively for the first challenge and 1462 epg (FEC2) and 36 epg (NEM2) respectively for the second challenge.

After the completion of the second field challenge, and the collection of faecal samples, lambs were slaughtered. From each lamb, the abomasum and first 5 m of small intestine were collected with the ends sealed to retain the contents for analysis. Laboratory analysis of the contents was designed to count the number of large L4, L5 larvae and adult worms using the method of Robertson and Elliott [41]. Direct examination of the sexual organs of male adults was the method used to identify the genus of adult nematodes. A full description of all phenotypes measured and used in the QTL analysis is provided in Table 2

### Immunological response measurements

*T. colubriformis *L3 total IgG antibodies [42] were measured by ELISA from serum samples taken on five separate occasions as follows: at weaning, at the end of the fourth week of the first field challenge, at the collection of the final faecal sample of the first field challenge, at the end of the fourth week of the second field challenge, and at the collection of the final faecal sample of the second field challenge just prior to slaughter. Total IgE in the serum was also measured [43] in the samples taken at the end of the second field challenge

### Other traits measured

Liveweight measurements were taken at birth, weaning, and after each challenge. Dag scores were recorded at weaning, and after each challenge. Dagginess was measured by visual examination of the hindquarters of each lamb and a score given from 0 to 5 depending on the degree of faecal contamination of the skin and wool surrounding the anus, with 0 = no contamination and 5 = very heavily soiled hindquarters (SIL Technical Bulletin "Dag Score" at [44]).

### Genotyping

All DNA markers used were microsatellites. The 5 sires were screened to determine whether each of the markers was heterozygous and if heterozygous the genotypes of the progeny determined. Initially only the 22 most resistant and 22 most susceptible progeny within each family were genotyped. These extremes were chosen on the basis of LFEC1, LFEC2, SNEM1 and SNEM2 after adjusting for fixed effects. The adjusted trait values were combined into a single trait for ranking. This trait was the first principal component (the linear combination that explains the most variation), i.e. 0.19 * adjusted LFEC1 + 0.47 * adjusted LFEC2 + 0.49 * adjusted SNEM1 + 0.71 * adjusted SNEM2. If some suggestion of linkage was identified on a particular chromosome after this initial screen all progeny were genotyped for all informative markers for this chromosome. The method of genotyping used radioactively labelled primers and 12% polyacrylamide sequencing gels to resolve the microsatellite allele sizes as described by Crawford et al. [45]. A complete list of all markers used in the genome screen is found in Additional Table 1 [Additional file 3].

With the exception of one marker developed as part of this project to improve the informativeness of Sheep chromosome 23 all information about the markers including, primer sequence, product sizes, and best Mg++ concentration can be found at the following web address, [46]. The marker developed for the project is an (AC)_n _microsatellite called Oar CDT2 and uses the primers 5'-CGTGCACAGAGATTGCATTC-3' and 5'-CACAGTATCAGTCATCTCTAGCTCC-3'. The amplification conditions were as previously described[45]

### Statistical analysis

Raw cohort means and standard deviations of the traits are shown in Additional Table 2. [Additional file 4] Correlations between traits, grouped into related sets, are shown in Additional Table 3 [Additional file 5]. Many of the traits were transformed to make the variance more homogeneous. Worm counts and FEC were transformed using natural logs, log_e_(*x*+*c*), where *x *is the count and *c *is the unit count. Total IgE was also log transformed. *Nematodirus *counts were further transformed by scaling each cohort to the average standard deviation and modelling the standard deviation of cohorts as linearly related to their means.

Genotype data was checked as follows. Sire-offspring pairs were compared for consistency with Mendelian inheritance and sire families for equal segregation of sire alleles. Linkage maps were calculated using CRI-MAP [47] and orders and distances checked for consistency with the map of Maddox et al [48]. Potential errant genotypes were rescored, and when necessary parentage relationships excluded.

The information content [49] was calculated for each family, along each chromosome. Regions of low information content are usually where only the extreme progeny were genotyped, but may also indicate that the sire was not heterozygous for any markers in the region.

The QTL detection method used was least squares interval mapping, using the Haley-Knott regression methods [50]. Fixed effects fitted for all traits were sex, cohort (birth year x farm combination) and sire, as well as the covariate of birth day of year. The QTL effect was fitted as the probability of the progeny receiving a particular sire allele (arbitrarily chosen to be the paternal or maternal alleles for each sire), and fitted within sire. The model for WTFEC2 also included the birth/rearing rank combination (single/single, multiple/single or multiple/multiple) and dam age (2 years or >2 years). The model for the ELISA traits also included the interaction between cohort and birth day.

The marker positions used were those estimated for male meioses, from [48] except that if a marker was not present on that map, its position was determined from the current population, and then placed on the map relative to markers of known position. Haley-Knott regression was performed at 2 cM intervals and both within family and across family (i.e. genotype probability within family) statistics collected. Genome-wise significant (P < 0.05) and suggestive (where the expected number of false positives is one) thresholds were determined by permutation methods [51] using 1000 permutations. Confidence intervals for the locations of significant QTL were found using bootstrap methods [52] using 500 replicates.

The suggestive and significant permutation thresholds for all traits and pedigree combinations are provided in Additional Figure 1 (All families combined) [Additional file 1] and Additional Figure 2 (each individual family) [Additional file 2]. These are compared to theoretical values derived using the methods of Lander and Kruglyak [53].

## Authors' contributions

AMC: experimental design, genotype analysis and manuscript preparation; KAP: oversight of genotyping, marker development; KGD: experimental design and statistical analysis; CDT: genotyping, marker development; PAW: genotyping; MRT: genotyping, marker development; SAB: Selection line development and provision of F1 rams; AEB: Statistical analysis; GJG: Oversight of phenotype measurement; RSG: measurement of serum IgG; RW: animal husbandry and phenotype measurement; RJS: measurement of serum total IgE; KK: oversight of animal husbandry; JCM: conception of the experiment and overall management of project.

## Supplementary Material

Additional File 1 Additonal figure 1. Significance thresholds for each trait across all families as determined by permutation. Click here for file

Additional File 2 Additonal figure 2. Significance thresholds for each trait/trait combination as determined by permutation. Click here for file

Additional File 3 Additional table 1. List of all DNA markers used in the linkage analysis. Click here for file

Additional File 4 Additional table 2. Raw means and standard deviations by cohort for traits analysed. Click here for file

Additional File 5 Additional table 3. Raw correlations between groups of traits. Click here for file

Additional File 6 Chr 1. Haley Knott linkage analysis of sheep chromosome 1. Click here for file

Additional File 7 Chr 2. Haley Knott linkage analysis of sheep chromosome 2. Click here for file

Additional File 8 Chr 3. Haley Knott linkage analysis of sheep chromosome 3. Click here for file

Additional File 9 Chr 4. Haley Knott linkage analysis of sheep chromosome 4. Click here for file

Additional File 10 Chr 5. Haley Knott linkage analysis of sheep chromosome 5. Click here for file

Additional File 11 Chr 6. Haley Knott linkage analysis of sheep chromosome 6. Click here for file

Additional File 12 Chr 7. Haley Knott linkage analysis of sheep chromosome 7. Click here for file

Additional File 13 Chr 8. Haley Knott linkage analysis of sheep chromosome 8. Click here for file

Additional File 14 Chr 9. Haley Knott linkage analysis of sheep chromosome 9. Click here for file

Additional File 15 Chr 10. Haley Knott linkage analysis of sheep chromosome 10. Click here for file

Additional File 16 Chr 11. Haley Knott linkage analysis of sheep chromosome 11. Click here for file

Additional File 17 Chr 12. Haley Knott linkage analysis of sheep chromosome 12. Click here for file

Additional File 18 Chr 13. Haley Knott linkage analysis of sheep chromosome 13. Click here for file

Additional File 19 Chr 14. Haley Knott linkage analysis of sheep chromosome 14. Click here for file

Additional File 20 Chr 15. Haley Knott linkage analysis of sheep chromosome 15. Click here for file

Additional File 21 Chr 16. Haley Knott linkage analysis of sheep chromosome 16. Click here for file

Additional File 22 Chr 17. Haley Knott linkage analysis of sheep chromosome 17. Click here for file

Additional File 23 Chr 18. Haley Knott linkage analysis of sheep chromosome 18. Click here for file

Additional File 24 Chr 19. Haley Knott linkage analysis of sheep chromosome 19. Click here for file

Additional File 25 Chr 20. Haley Knott linkage analysis of sheep chromosome 20. Click here for file

Additional File 26 Chr 21. Haley Knott linkage analysis of sheep chromosome 21. Click here for file

Additional File 27 Chr 22. Haley Knott linkage analysis of sheep chromosome 22. Click here for file

Additional File 28 Chr 23. Haley Knott linkage analysis of sheep chromosome 23. Click here for file

Additional File 29 Chr 24. Haley Knott linkage analysis of sheep chromosome 24. Click here for file

Additional File 30 Chr 25. Haley Knott linkage analysis of sheep chromosome 25. Click here for file

Additional File 31 Chr 26. Haley Knott linkage analysis of sheep chromosome 26. Click here for file

## Figures and Tables

**Table 1:** Half-sib outcross sheep families used to detect QTL for disease resistance

**Family**	**Sire**	**Number of progeny**
1	92/0066	225
2	92/0153	175
3	92/0154	348
4	92/0155	111
5	93/0124	101

**Table 2:** A description of the phenotype measurements used in the linkage analysis and their associated abbreviation

**Abbreviation**	**Description of phenotype measurement**	**Units of measurement**	σ_p_*
DAG0	Dag score at weaning	0–5 scale	0.56
DAG1	Dag score at the end of the first parasite challenge	0–5 scale	0.89
DAG2	Dag score at the end of the second parasite challenge	0–5 scale	0.82
ELISA1	IgG specific to *T.colubriformis *in serum collected at weaning	OD_490 _using reference sera as standards	0.079
ELISA2	IgG specific to *T.colubriformis *in serum collected 4 weeks after weaning	OD_490 _using reference sera as standards	0.095
ELISA3	IgG specific to *T.colubriformis *in serum collected at the end of the first parasite challenge	OD_490 _using reference sera as standards	0.157
ELISA4	IgG specific to *T.colubriformis *in serum collected 4 weeks after the start of the second field challenge	OD_490 _using reference sera as standards	0.158
ELISA5	IgG specific to *T.colubriformis *in serum collected at the end of the second field challenge	OD_490 _using reference sera as standards	0.171
LAOST	Log transformed estimates of *T. circumcincta *adults and late stage larvae found in the abomasum collected at slaughter at the end of the second parasite challenge	Number of individuals	0.92
LATRICH	Log transformed estimates of *Trichostrongylus spp*. adults and late stage larvae found in the abomasum collected at slaughter at the end of the second parasite challenge	Number of individuals	0.69
LFEC1	Log transformed mean estimate of *Strongyle *eggs per gram of faecal material collected and measured 3 times over five days at the end of the first parasite challenge	Number of eggs per gram of faeces	0.51
LFEC2	Log transformed mean estimate of *Strongyle *eggs per gram of faecal material collected and measured 3 times over five days at the end of the second parasite challenge	Number of eggs per gram of faeces	0.68
LIGE	Log transformed estimates of total IgE in serum collected at the end of the second parasite challenge	OD_450 _using reference sera as standards	0.70
LSINEM	Log transformed estimates of *Nematadirus sp*. adults and late stage larvae found in the small intestine collected at slaughter at the end of the second parasite challenge	Number of individuals	1.22
LSITRI	Log transformed estimates of *Trichostrongylus spp*. adults and late stage larvae found in the small intestine collected at slaughter at the end of the second parasite challenge	Number of individuals	0.82
SNEM1	Scaled mean estimate of *Nematodirus spp*. eggs per gram of faecal material collected and measured 3 times over five days at the end of the first parasite challenge	Number of eggs per gram of faeces	0.76
SNEM2	Scaled mean estimate of *Nematodirus spp*. eggs per gram of faecal material collected and measured 3 times over five days at the end of the second parasite challenge	Number of eggs per gram of faeces	0.82
WTFEC2	Liveweight of lambs at the end of the second parasite challenge	kilograms	3.63

* σ_p _= residual SD from a model containing all fixed effects including sire (but no marker effects).

**Table 3:** QTL Discovered with genome-wide significant linkage

**Chromosome**	**Trait Abbreviation**	**Sire**	**Estimate of effect ± SE**	**Nearest marker**	**Bracketing markers**	**95% Bootstrap confidence interval**
2	LATRICH	124	1.63 ± 0.41	BM81124	BMS1341/HH30	BMS1341 – FCB11
8	LATRICH	124	0.82 ± 0.20	BM3215	Telomere/BM4208	Whole chromosome
8	LSITRI	124	0.87 ± 0.24	BM3215	Telomere/BM4208	Whole chromosome
11	LSITRI	All sires combined		BM9202	Centromere/ETH3	BM9202 – MB087
23	ELISA4	154	0.07 ± 0.02	ILSTS65	McMA1/ADCYCAP1	CDT2 – ADCYAP1
23	LIGE	154	0.20 ± 0.08	ILSTS42	Centomere/BM226	Whole chromosome
