# Supplementary figures and images for: Discovery of quantitative trait loci for resistance to parasitic nematode infection in sheep: I. Analysis of outcross pedigrees
Source: BMC Genomics. 2006 Jul 18;7:178. doi: 10.1186/1471-2164-7-178 (PMC1574317; doi:10.1186/1471-2164-7-178)

**Significance Thresholds for each trait across all families as determined by permutation**

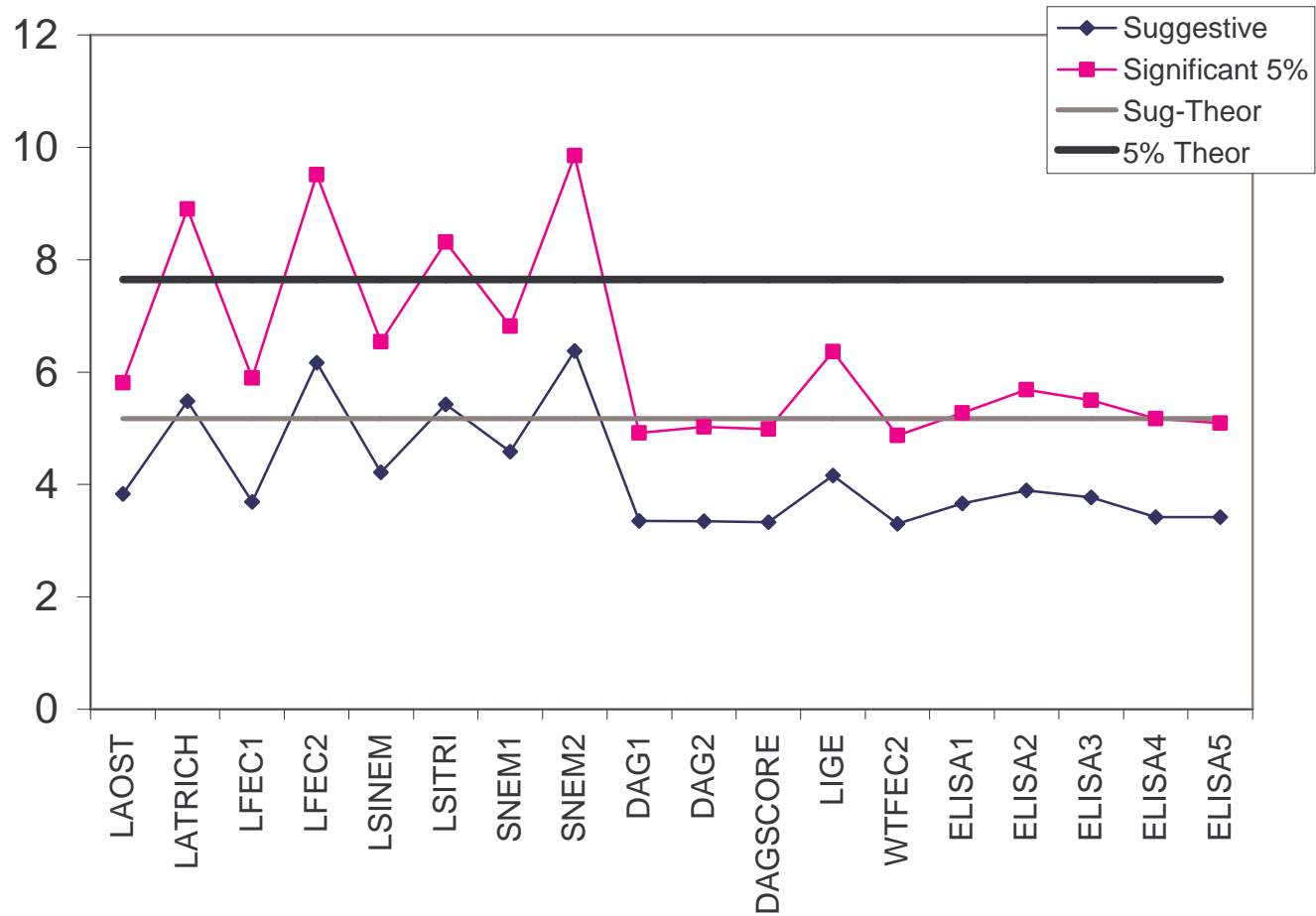

Supplement: Additional File 1 — Additonal figure 1. Significance thresholds for each trait across all families as determined by permutation. [file 1471-2164-7-178-S1.pdf]

Significance Thresholds for each family / trait combination determined by permutation

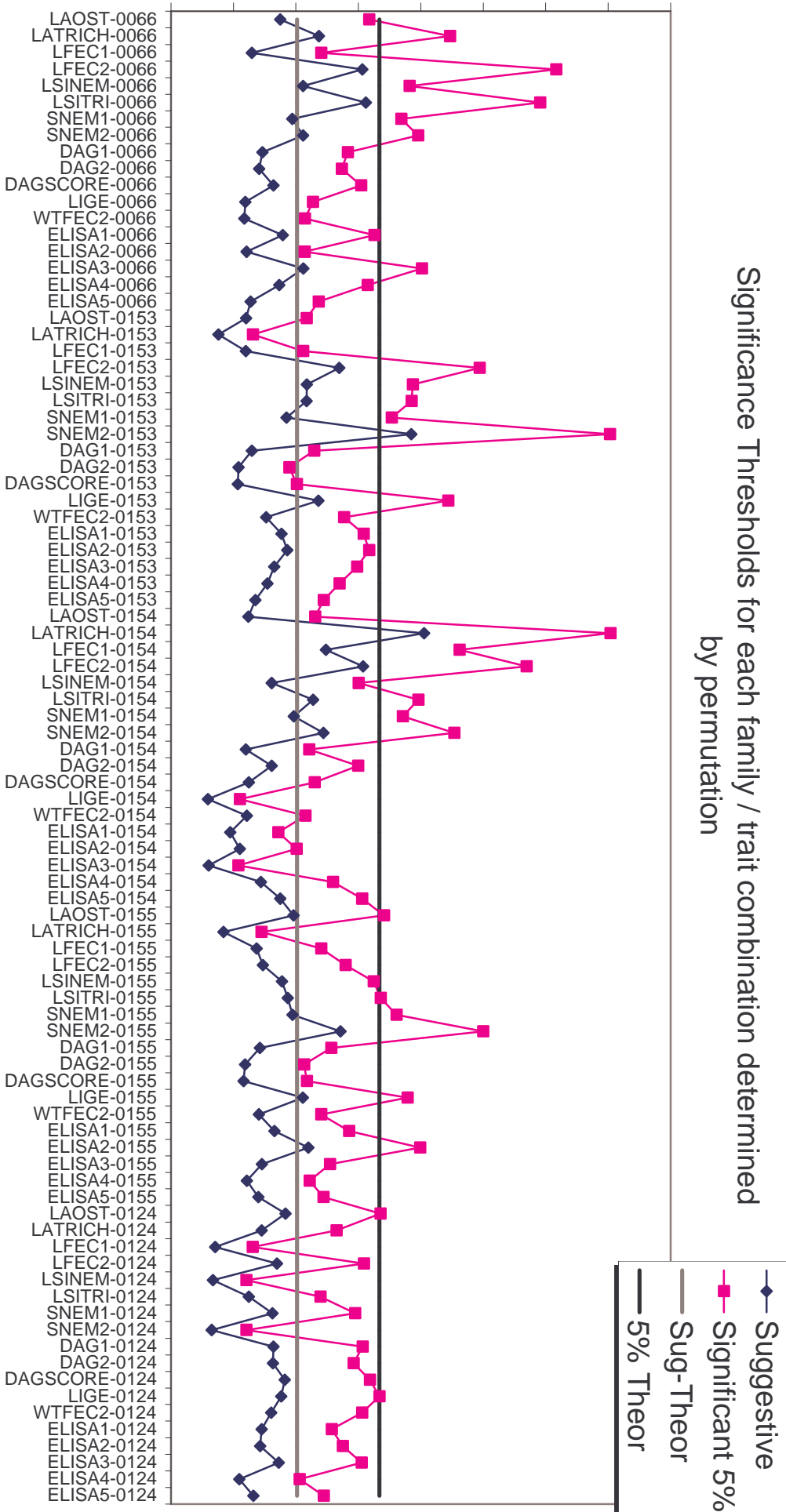

Supplement: Additional File 2 — Additonal figure 2. Significance thresholds for each trait/trait combination as determined by permutation. [file 1471-2164-7-178-S2.pdf]
