# Supplementary material for: Discovery of quantitative trait loci for resistance to parasitic nematode infection in sheep: I. Analysis of outcross pedigrees
Source: BMC Genomics. 2006 Jul 18;7:178. doi: 10.1186/1471-2164-7-178 (PMC1574317; doi:10.1186/1471-2164-7-178)
Supplement: Additional File 3 — Additional table 1. List of all DNA markers used in the linkage analysis. [file 1471-2164-7-178-S3.doc]

## Additional Table 1. Markers used in the genome scan of 5 outcross pedigrees segregating for parasite resistance

| **Marker name*** | **Chromosome** | **Number of informative mieoses** |
| --- | --- | --- |
| BL41 | 1 | 531 |
| BM4129 | 1 | 546 |
| BM6465 | 1 | 686 |
| BM6506 | 1 | 333 |
| BMS2145 | 1 | 376 |
| BMS2263 | 1 | 659 |
| BMS482 | 1 | 85 |
| BMS599 | 1 | 67 |
| BMS835 | 1 | 110 |
| CSSM32 | 1 | 148 |
| CSSM04 | 1 | 479 |
| DB6 | 1 | 684 |
| EPCDV10 | 1 | 350 |
| ILSTS29 | 1 | 542 |
| ILSTS04 | 1 | 706 |
| ILSTS44 | 1 | 616 |
| INRA11 | 1 | 443 |
| KAP8 | 1 | 80 |
| MAF109 | 1 | 601 |
| MAF4 | 1 | 743 |
| MAF64 | 1 | 107 |
| MCM130 | 1 | 244 |
| TGLA49 | 1 | 335 |
| FCB226 | 2 | 92 |
| MCM64 | 2 | 60 |
| BMS1341 | 2 | 124 |
| TGLA10 | 2 | 156 |
| BM81124 | 2 | 99 |
| CP79 | 2 | 90 |
| HH30 | 2 | 103 |
| FCB20 | 2 | 109 |
| BMS2626 | 2 | 87 |
| ARO28 | 2 | 20 |
| BM6444 | 2 | 101 |
| FCB11 | 2 | 162 |
| BMS1350 | 3 | 722 |
| CP34 | 3 | 664 |
| TGLA77 | 3 | 224 |
| FCB129 | 3 | 61 |
| ILSTS49 | 3 | 618 |
| RM96 | 3 | 54 |
| BM827 | 3 | 533 |
| ILSTS42 | 3 | 83 |
| FCB5 | 3 | 305 |
| BMC1009 | 3 | 221 |
| IFNG | 3 | 18 |
| VH34 | 3 | 71 |
| MAF23 | 3 | 484 |
| CP43 | 3 | 174 |
| BM8230 | 3 | 600 |
| BMS1248 | 3 | 143 |
| BMS772 | 3 | 359 |
| BMS1788 | 4 | 449 |
| MCM218 | 4 | 610 |
| BMS1172 | 4 | 606 |
| MAF70 | 4 | 116 |
| TGLA116 | 4 | 236 |
| MAF50 | 4 | 409 |
| CP26 | 4 | 407 |
| HH35 | 4 | 643 |
| BM3212 | 4 | 260 |
| HH64 | 4 | 433 |
| BM1853 | 5 | 169 |
| BM9289 | 5 | 69 |
| BMS1247 | 5 | 58 |
| BMS792 | 5 | 151 |
| MCM108 | 5 | 160 |
| MCM527 | 5 | 96 |
| SHP1 | 5 | 174 |
| TGLA137 | 5 | 147 |
| TGLA176 | 5 | 52 |
| TGLA48 | 5 | 322 |
| CP125 | 6 | 198 |
| BM1329 | 6 | 22 |
| AE101 | 6 | 696 |
| HH55 | 6 | 32 |
| BM4621 | 6 | 132 |
| BM4311 | 6 | 165 |
| JMP4 | 6 | 70 |
| JMP8 | 6 | 587 |
| JMP12 | 6 | 351 |
| BM3033 | 7 | 28 |
| BMS528 | 7 | 95 |
| BMS861 | 7 | 41 |
| INRA71 | 7 | 64 |
| BMS1620 | 7 | 80 |
| AE64 | 7 | 187 |
| BM1227 | 8 | 70 |
| BM2504 | 8 | 42 |
| UWCA9 | 8 | 19 |
| CP21 | 8 | 725 |
| TGLA73 | 8 | 533 |
| BM4208 | 8 | 269 |
| BM3215 | 8 | 267 |
| BM757 | 9 | 77 |
| BMS1678 | 9 | 70 |
| ILSTS11 | 9 | 60 |
| BM4630 | 9 | 137 |
| MAF33 | 9 | 268 |
| BMS1304 | 9 | 140 |
| CP9 | 9 | 96 |
| HH41 | 10 | 147 |
| BMS975 | 10 | 143 |
| ILSTS56 | 10 | 36 |
| BMS585 | 10 | 93 |
| DB3 | 10 | 64 |
| CP38 | 10 | 79 |
| BM9202 | 11 | 78 |
| HEL10 | 11 | 56 |
| TGLA51 | 11 | 33 |
| BM17132 | 11 | 148 |
| MB087 | 11 | 78 |
| ETH3 | 11 | 103 |
| BM4025 | 12 | 385 |
| BM719 | 12 | 89 |
| BMS357 | 12 | 61 |
| BMS538 | 12 | 222 |
| CSAP001E | 12 | 309 |
| HUJ625 | 12 | 55 |
| INRA13 | 12 | 229 |
| MCM507 | 12 | 72 |
| MCMA52 | 12 | 353 |
| TGLA53 | 12 | 181 |
| BM4509 | 13 | 21 |
| HUJ616 | 13 | 80 |
| IL2RA | 13 | 449 |
| MAF18 | 13 | 83 |
| MCM152 | 13 | 417 |
| MMP9 | 13 | 414 |
| TEXAN10 | 14 | 24 |
| MT2 | 14 | 75 |
| BM7109 | 14 | 69 |
| ILSTS02 | 14 | 146 |
| BR3510 | 15 | 94 |
| ADCYC | 15 | 30 |
| FSHB | 15 | 47 |
| CAT | 15 | 155 |
| ILSTS27 | 15 | 60 |
| RM004 | 15 | 41 |
| BM848 | 15 | 95 |
| RM106 | 16 | 151 |
| BM1225 | 16 | 114 |
| CP99 | 16 | 45 |
| MAF214 | 16 | 65 |
| HH62 | 16 | 105 |
| BM5004 | 16 | 19 |
| CP49 | 17 | 81 |
| VH116 | 17 | 51 |
| MAF209 | 17 | 135 |
| CP16 | 17 | 16 |
| BL50 | 17 | 66 |
| ILSTS58 | 17 | 42 |
| TGLA322 | 17 | 129 |
| BM3413 | 18 | 32 |
| CP134 | 18 | 80 |
| UWCA4 | 18 | 69 |
| HH47 | 18 | 105 |
| CSSM18 | 18 | 67 |
| BMS1561 | 18 | 44 |
| BM1558 | 19 | 158 |
| AE119 | 19 | 252 |
| BM2613 | 19 | 291 |
| BMS390 | 19 | 49 |
| FCB304 | 19 | 487 |
| BM1258 | 20 | 91 |
| TGLA387 | 20 | 109 |
| SMHCC1 | 20 | 140 |
| BP34 | 20 | 85 |
| ILSTS19 | 21 | 106 |
| CP20 | 21 | 107 |
| HH22 | 21 | 24 |
| OCAM | 21 | 77 |
| BMC1206 | 21 | 149 |
| BMS1948 | 21 | 127 |
| BMS651 | 22 | 60 |
| BMS907 | 22 | 788 |
| BM1314 | 22 | 351 |
| BM4505 | 22 | 496 |
| MAF92 | 22 | 259 |
| MAF36 | 22 | 521 |
| CDT2 | 23 | 181 |
| BL6 | 23 | 585 |
| BM226 | 23 | 296 |
| BMS2526 | 23 | 489 |
| MCMA1 | 23 | 427 |
| BMS2270 | 23 | 191 |
| ILSTS65 | 23 | 513 |
| ADCYAP1 | 23 | 282 |
| MAF35 | 23 | 136 |
| BMS1332 | 23 | 657 |
| MCM136 | 23 | 744 |
| BMS514 | 24 | 79 |
| JMP29 | 24 | 77 |
| ILSTS43 | 24 | 34 |
| BP28 | 24 | 107 |
| EL01 | 24 | 65 |
| BL25 | 25 | 21 |
| VH72 | 25 | 128 |
| AE54 | 25 | 146 |
| BM6526 | 26 | 91 |
| CSSM43 | 26 | 33 |
| JMP23 | 26 | 68 |
| BM203 | 26 | 34 |

*Details of all markers including, primer sequence, product sizes, and best Mg++ concentration can be found at [45]
