# Supplementary material for: Discovery of quantitative trait loci for resistance to parasitic nematode infection in sheep: I. Analysis of outcross pedigrees
Source: BMC Genomics. 2006 Jul 18;7:178. doi: 10.1186/1471-2164-7-178 (PMC1574317; doi:10.1186/1471-2164-7-178)
Supplement: Additional File 4 — Additional table 2. Raw means and standard deviations by cohort for traits analysed. [file 1471-2164-7-178-s4.doc]

## Additional Table 2 – Raw means and standard deviations by cohort for traits analysed

|  | Cohort^†^ | | | | | | | |
| --- | --- | --- | --- | --- | --- | --- | --- | --- |
|  | G93 | |  | G94 | |  | W94 | |
| Trait* | Mean | sd |  | Mean | sd |  | Mean | sd |
| FEC1 | 992 | 738 |  | 1429 | 586 |  | 855 | 438 |
| FEC2 | 1603 | 874 |  | 1463 | 1074 |  | 1330 | 803 |
| NEM1 | 8 | 13 |  | 51 | 57 |  | 152 | 143 |
| NEM2 | 16 | 22 |  | 47 | 79 |  | 44 | 112 |
| SINEM | 1485 | 1512 |  | 636 | 1574 |  | 706 | 1771 |
| SITRI | 10027 | 4858 |  | 23715 | 14649 |  | 12818 | 8299 |
| AOST | 7702 | 4679 |  | 4523 | 4729 |  | 4482 | 3841 |
| ATRICH | 5294 | 2880 |  | 5274 | 3227 |  | 4299 | 2185 |
| DAG0 | 0.5 | 0.5 |  | 0.5 | 0.6 |  | 0.6 | 0.5 |
| DAG1 | 1.8 | 0.9 |  | 1.6 | 1.0 |  | 1.4 | 0.9 |
| DAG2 | 1.1 | 0.9 |  | 1.4 | 1.0 |  | 1.1 | 0.7 |
| ELISA1 | 0.25 | 0.06 |  | 0.35 | 0.09 |  | 0.27 | 0.09 |
| ELISA2 | 0.41 | 0.09 |  | 0.33 | 0.09 |  | 0.37 | 0.11 |
| ELISA3 | 0.35 | 0.10 |  | 0.73 | 0.19 |  | 0.70 | 0.19 |
| ELISA4 | 0.65 | 0.17 |  | 0.76 | 0.17 |  | 0.77 | 0.15 |
| ELISA5 | 0.85 | 0.19 |  | 0.80 | 0.17 |  | 0.77 | 0.16 |
| IGE | 0.91 | 0.33 |  | 0.59 | 0.37 |  | 0.47 | 0.34 |
| WTFEC2 | 30.7 | 4.3 |  | 29.6 | 4.3 |  | 31.4 | 4.6 |

* Traits are as shown in Table 2, except where the first letter L or S has been dropped to indicate untransformed values.

^†^ The number of animals in each cohort were 323 in G93, 317 in G94 and 320 in W94.
