# Supplementary material for: Discovery of quantitative trait loci for resistance to parasitic nematode infection in sheep: I. Analysis of outcross pedigrees
Source: BMC Genomics. 2006 Jul 18;7:178. doi: 10.1186/1471-2164-7-178 (PMC1574317; doi:10.1186/1471-2164-7-178)
Supplement: Additional File 5 — Additional table 3. Raw correlations between groups of traits. [file 1471-2164-7-178-S5.doc]

## Additional Table 3 – Raw correlations between groups of traits

| First trait^†^ | Second trait | | | | | | | | | | | | | |
| --- | --- | --- | --- | --- | --- | --- | --- | --- | --- | --- | --- | --- | --- | --- |
|  | SINEM |  | SITRI |  | AOST |  | ATRICH |  |  |  |  |  |  |  |
| SINEM | 1 |  |  |  |  |  |  |  |  |  |  |  |  |  |
| SITRI | 0.06 |  | 1 |  |  |  |  |  |  |  |  |  |  |  |
| AOST | 0.22 | *** | 0.22 | *** | 1 |  |  |  |  |  |  |  |  |  |
| ATRICH | 0.14 | *** | 0.45 | *** | 0.43 | *** | 1 |  |  |  |  |  |  |  |
|  |  |  |  |  |  |  |  |  |  |  |  |  |  |  |
|  | ELISA1 |  | ELISA2 |  | ELISA3 |  | ELISA4 |  | ELISA5 |  | IGE |  |  |  |
| ELISA1 | 1 |  |  |  |  |  |  |  |  |  |  |  |  |  |
| ELISA2 | -0.13 | *** | 1 |  |  |  |  |  |  |  |  |  |  |  |
| ELISA3 | 0.21 | *** | 0.02 |  | 1 |  |  |  |  |  |  |  |  |  |
| ELISA4 | 0.08 | * | 0.22 | *** | 0.57 | *** | 1 |  |  |  |  |  |  |  |
| ELISA5 | -0.04 |  | 0.18 | *** | 0.21 | *** | 0.46 | *** | 1 |  |  |  |  |  |
| IGE | -0.10 | * | 0.20 | *** | -0.19 | *** | 0.04 |  | 0.35 | *** | 1 |  |  |  |
|  |  |  |  |  |  |  |  |  |  |  |  |  |  |  |
|  | DAG0 |  | DAG1 |  | DAG2 |  |  |  |  |  |  |  |  |  |
| DAG0 | 1 |  |  |  |  |  |  |  |  |  |  |  |  |  |
| DAG1 | 0.16 | *** | 1 |  |  |  |  |  |  |  |  |  |  |  |
| DAG2 | 0.13 | *** | 0.53 | *** | 1 |  |  |  |  |  |  |  |  |  |
|  |  |  |  |  |  |  |  |  |  |  |  |  |  |  |
|  | FEC1 |  | FEC2 |  | NEM1 |  | NEM2 |  | SITRI |  | ELISA5 |  | DAG2 |  |
| FEC1 | 1 |  |  |  |  |  |  |  |  |  |  |  |  |  |
| FEC2 | 0.23 | *** | 1 |  |  |  |  |  |  |  |  |  |  |  |
| NEM1 | 0.03 |  | 0.05 |  | 1 |  |  |  |  |  |  |  |  |  |
| NEM2 | 0.12 | *** | 0.30 | *** | 0.30 | *** | 1 |  |  |  |  |  |  |  |
| SITRI | 0.39 | *** | 0.48 | *** | 0.13 | *** | 0.26 | *** | 1 |  |  |  |  |  |
| ELISA5 | 0.07 | * | -0.13 | *** | -0.15 | *** | -0.16 | *** | -0.07 | * | 1 |  |  |  |
| DAG2 | 0.09 | * | -0.01 |  | -0.06 |  | -0.04 |  | 0.18 | *** | 0.06 |  | 1 |  |

^†^ Traits are as shown in Table 2, except where the first letter L or S has been dropped to indicate untransformed values.

^*^ P<0.05; ^**^ P<0.01; ^***^ P<0.001
