# Supplementary material for: Discovery of quantitative trait loci for resistance to parasitic nematode infection in sheep: I. Analysis of outcross pedigrees
Source: BMC Genomics. 2006 Jul 18;7:178. doi: 10.1186/1471-2164-7-178 (PMC1574317; doi:10.1186/1471-2164-7-178)

Linkage Analysis in the Parasite Outcross Flock: Chromosome 1

Information Content: Chromosome 1

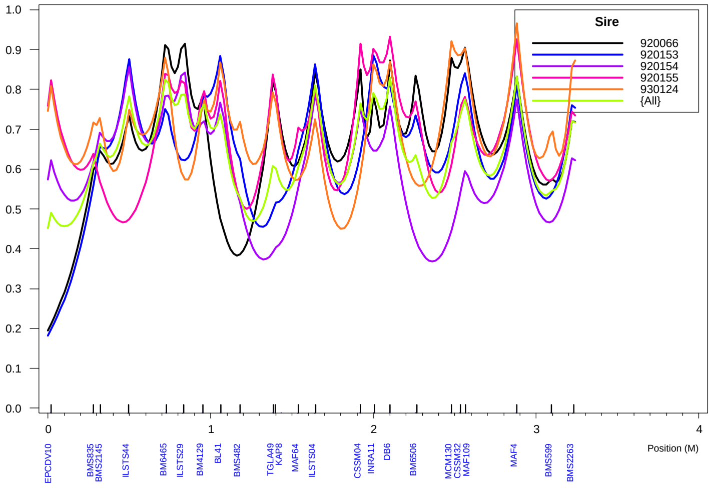

Haley-Knott QTL Analysis: Chromosome 1

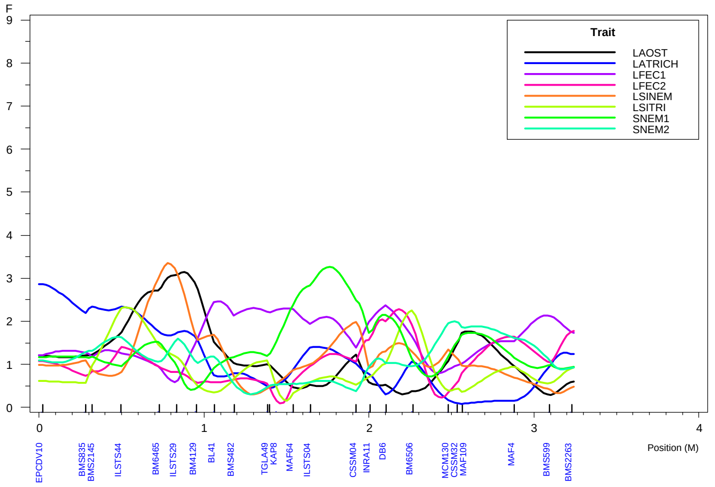

Haley-Knott QTL Analysis: Chromosome 1

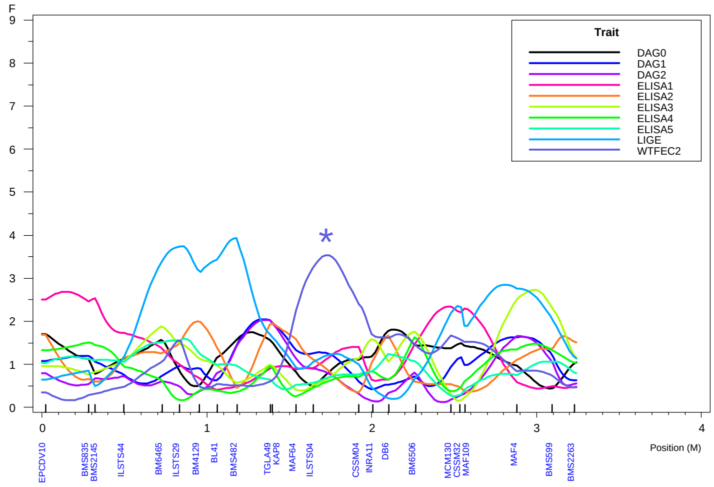

Haley-Knott QTL Analysis: Chromosome 1
LFEC1

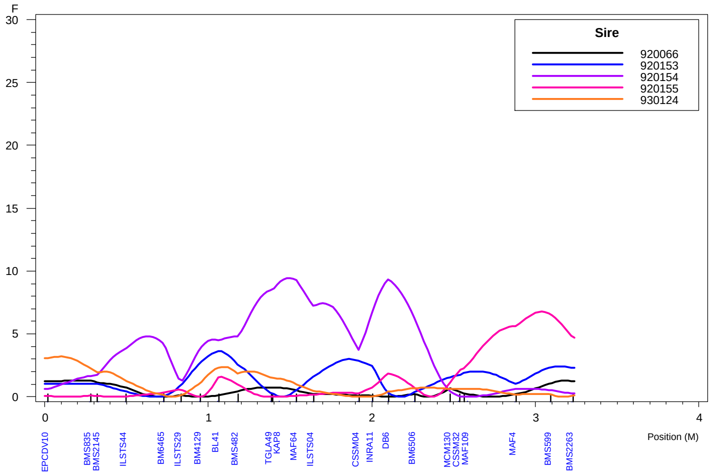

Haley-Knott QTL Analysis: Chromosome 1
LFEC2

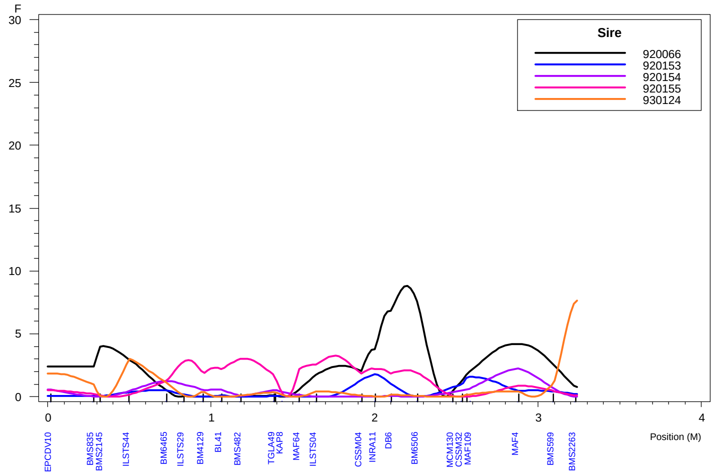

Haley-Knott QTL Analysis: Chromosome 1
SNEM1

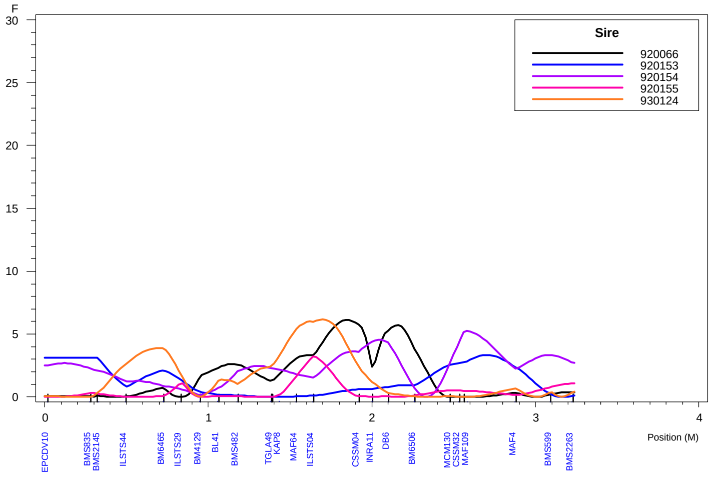

Haley-Knott QTL Analysis: Chromosome 1
SNEM2

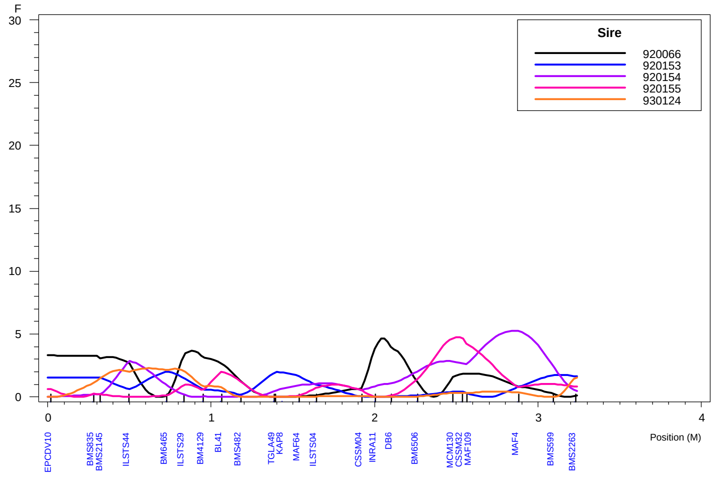

Haley-Knott QTL Analysis: Chromosome 1
LSINEM

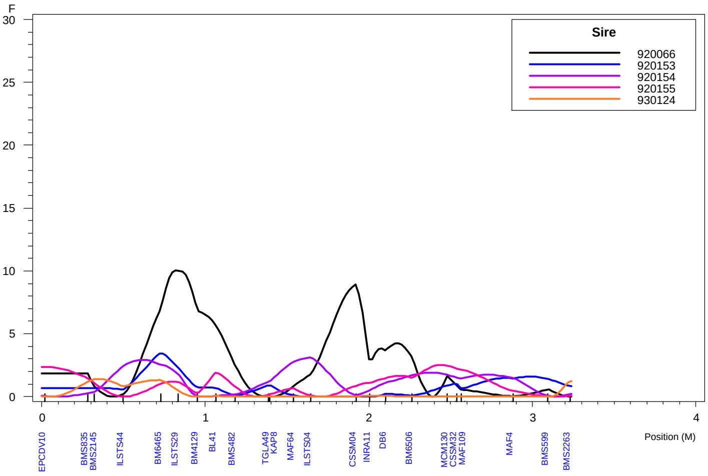

Haley-Knott QTL Analysis: Chromosome 1
LSITRI

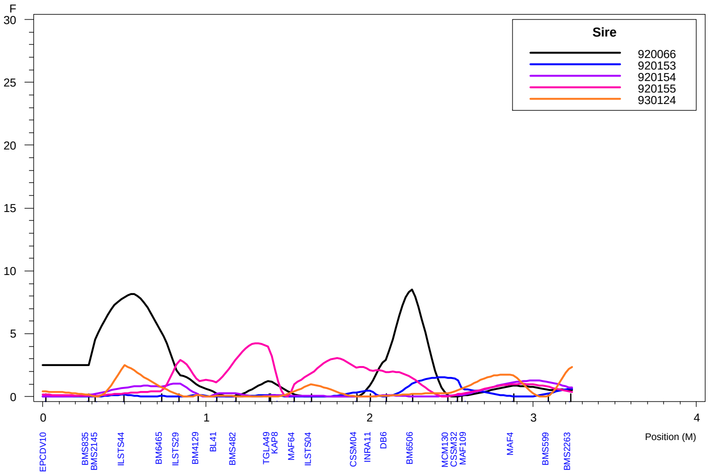

Haley-Knott QTL Analysis: Chromosome 1
LAOST

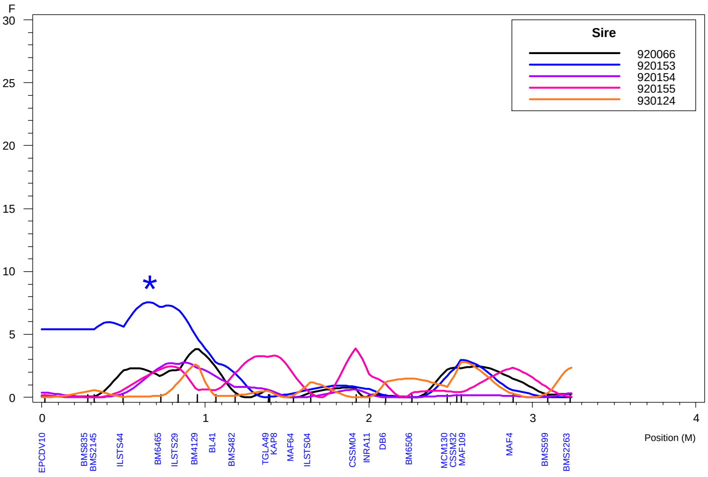

Haley-Knott QTL Analysis: Chromosome 1
LATRICH

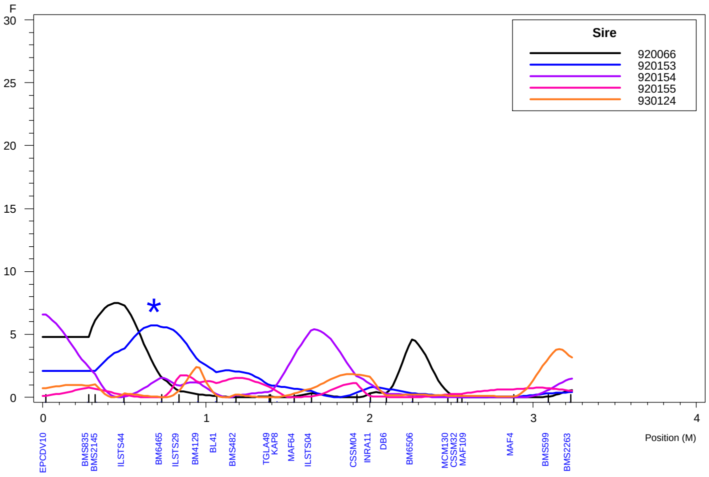

Haley-Knott QTL Analysis: Chromosome 1
DAG0

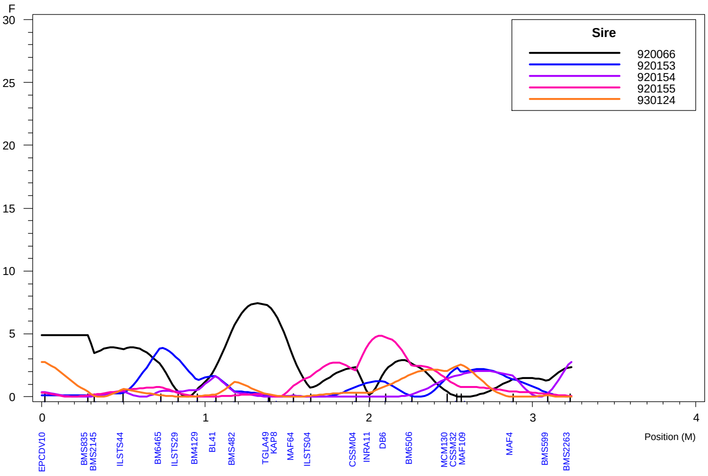

Haley-Knott QTL Analysis: Chromosome 1
DAG1

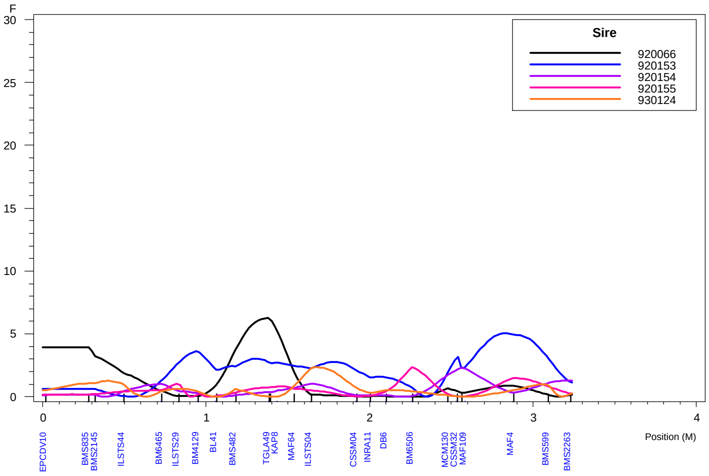

Haley-Knott QTL Analysis: Chromosome 1
DAG2

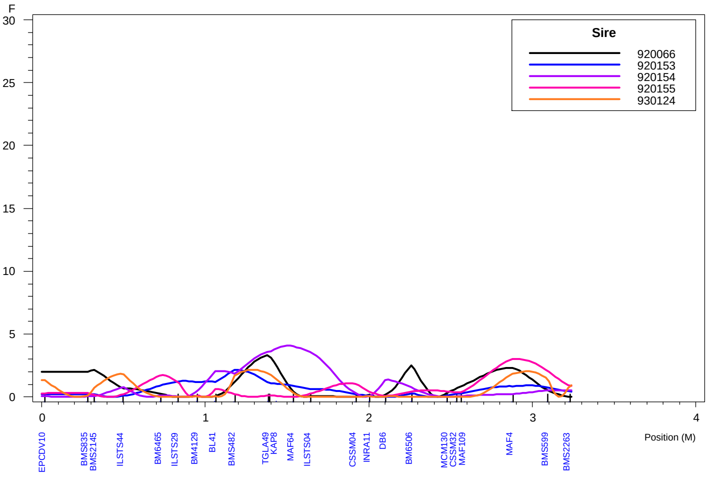

Haley-Knott QTL Analysis: Chromosome 1
ELISA1

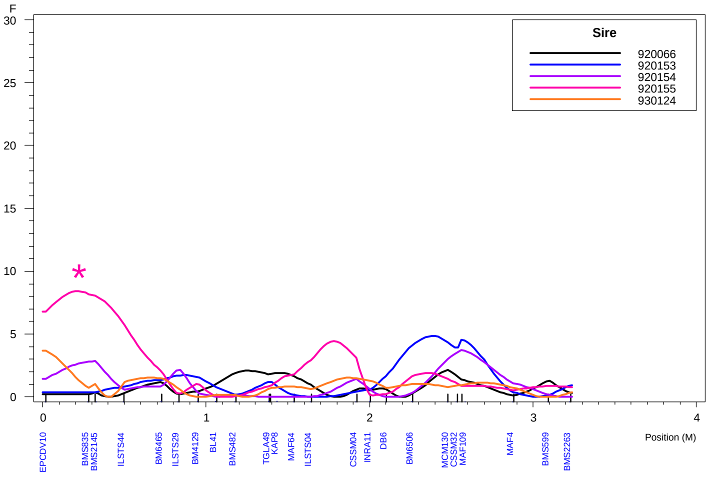

Haley-Knott QTL Analysis: Chromosome 1
ELISA2

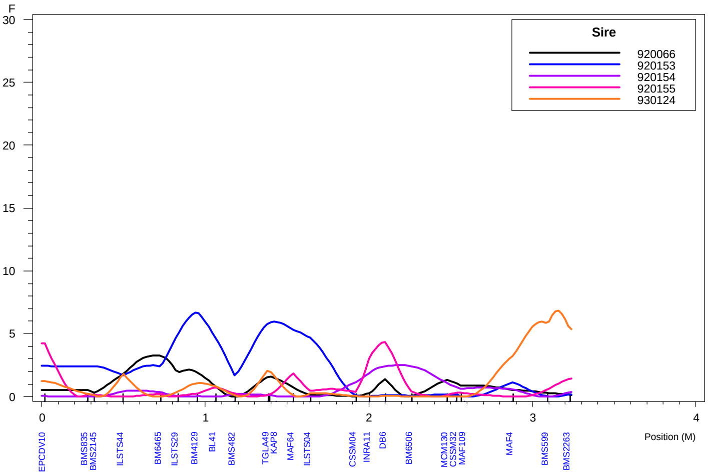

Haley-Knott QTL Analysis: Chromosome 1
ELISA3

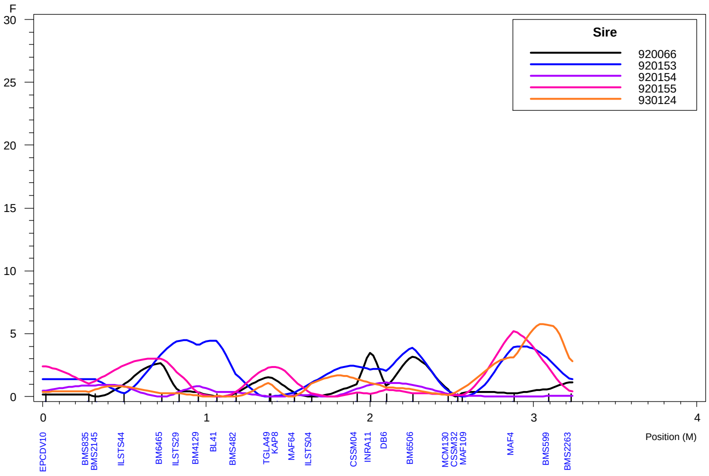

Haley-Knott QTL Analysis: Chromosome 1
ELISA4

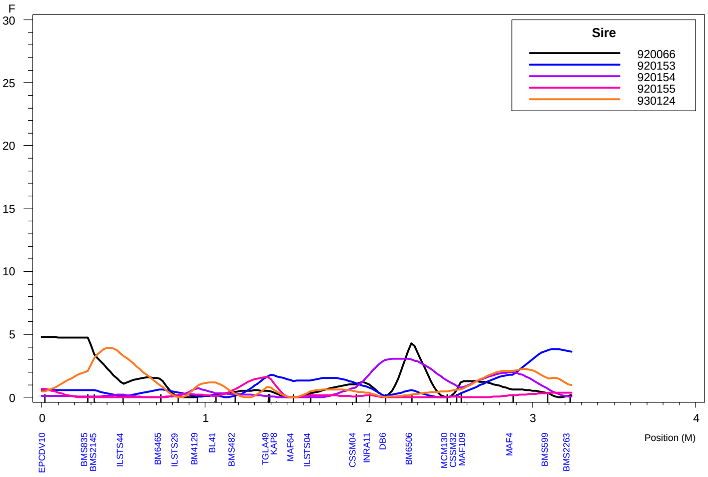

Haley-Knott QTL Analysis: Chromosome 1
ELISA5

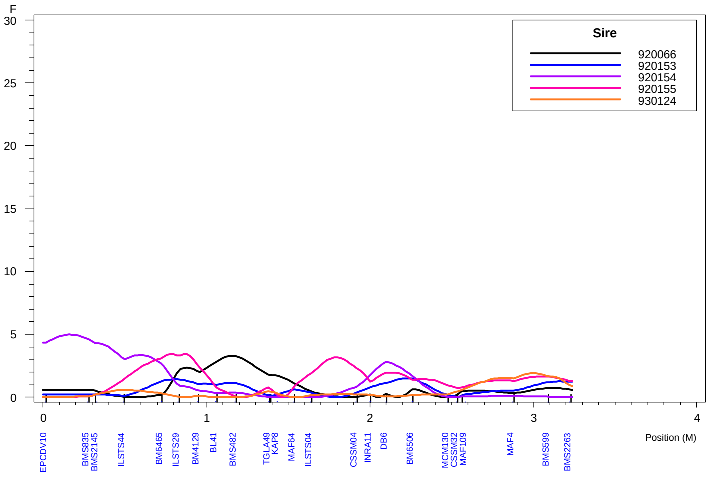

Haley-Knott QTL Analysis: Chromosome 1
LIGE

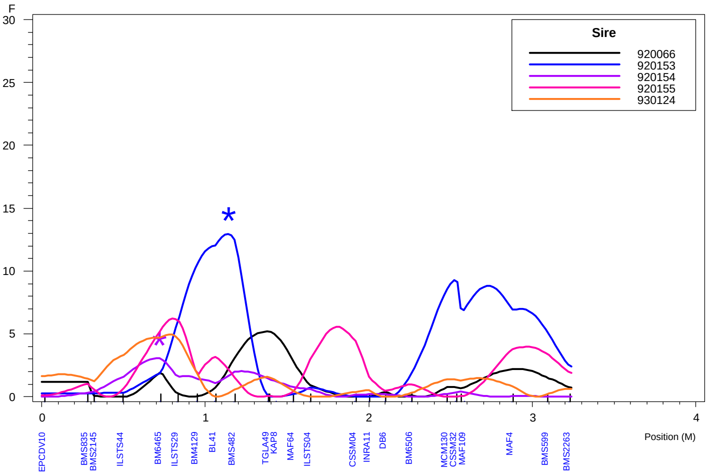

Haley-Knott QTL Analysis: Chromosome 1
WTFEC2

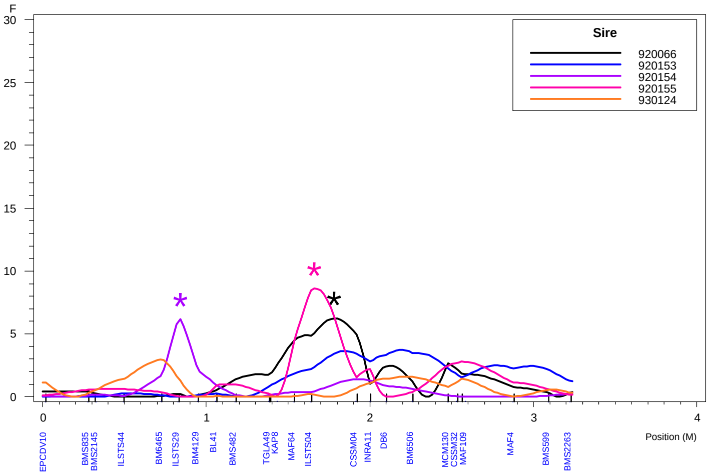

Supplement: Additional File 6 — Chr 1. Haley Knott linkage analysis of sheep chromosome 1. [file 1471-2164-7-178-S6.pdf]
