# Supplementary material for: Discovery of quantitative trait loci for resistance to parasitic nematode infection in sheep: I. Analysis of outcross pedigrees
Source: BMC Genomics. 2006 Jul 18;7:178. doi: 10.1186/1471-2164-7-178 (PMC1574317; doi:10.1186/1471-2164-7-178)

Linkage Analysis in the Parasite Outcross Flock: Chromosome 2

Information Content: Chromosome 2

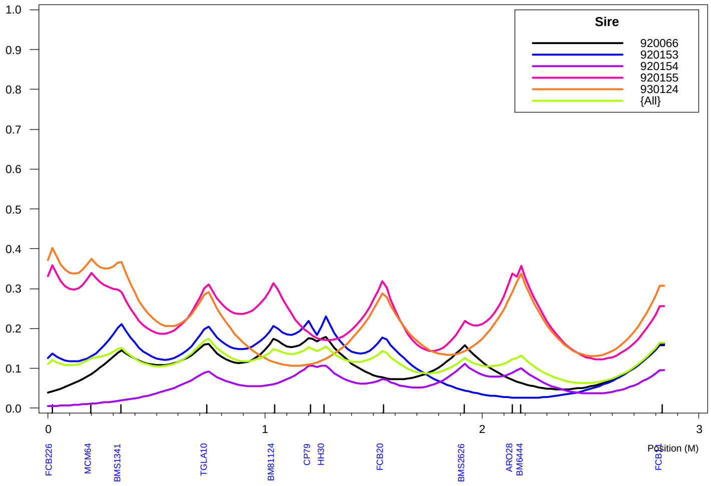

Haley-Knott QTL Analysis: Chromosome 2

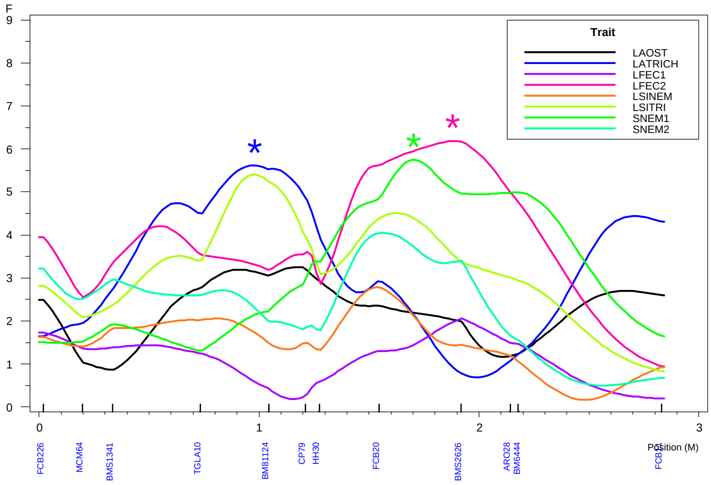

Haley-Knott QTL Analysis: Chromosome 2

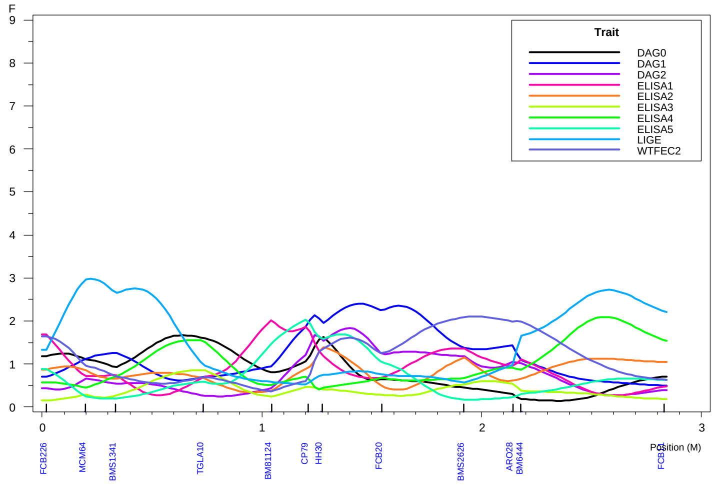

Haley-Knott QTL Analysis: Chromosome 2
LFEC1

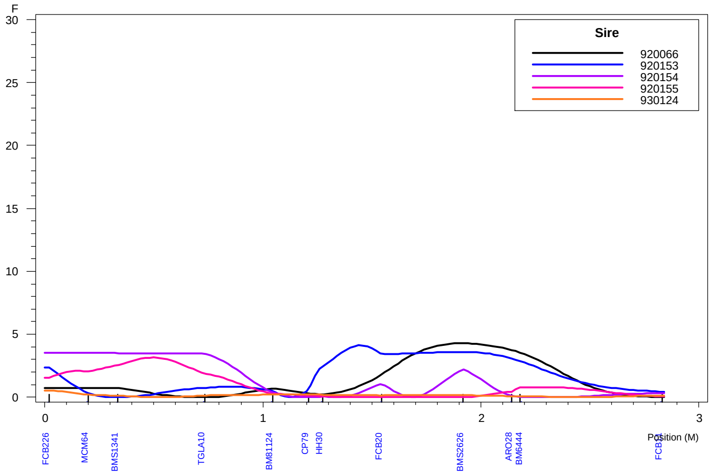

Haley-Knott QTL Analysis: Chromosome 2
LFEC2

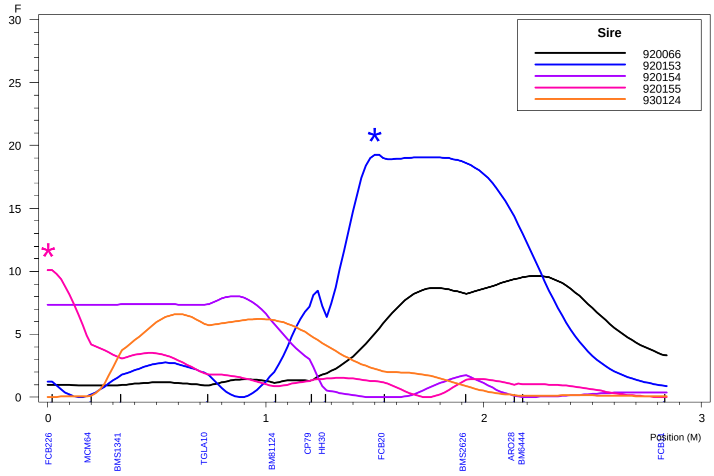

Haley-Knott QTL Analysis: Chromosome 2
SNEM1

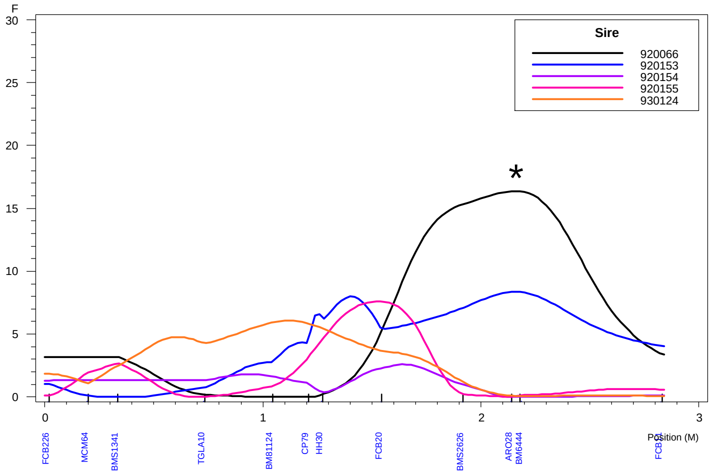

Haley-Knott QTL Analysis: Chromosome 2
SNEM2

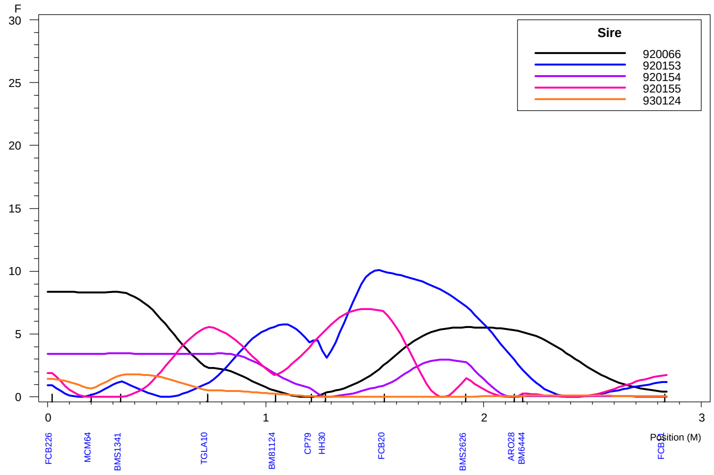

Haley-Knott QTL Analysis: Chromosome 2
LSINEM

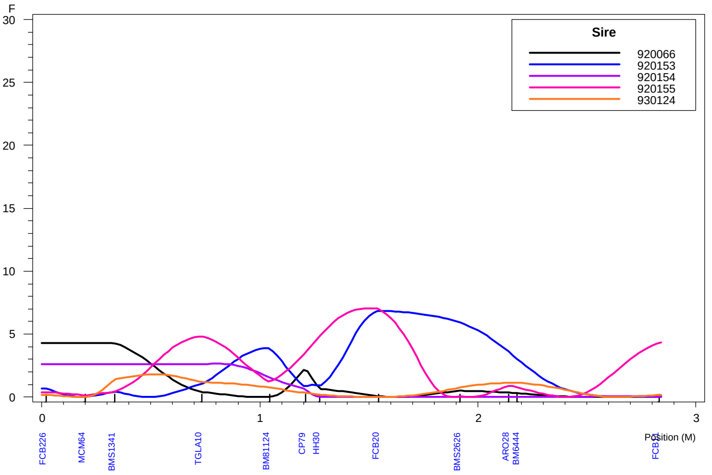

Haley-Knott QTL Analysis: Chromosome 2
LSITRI

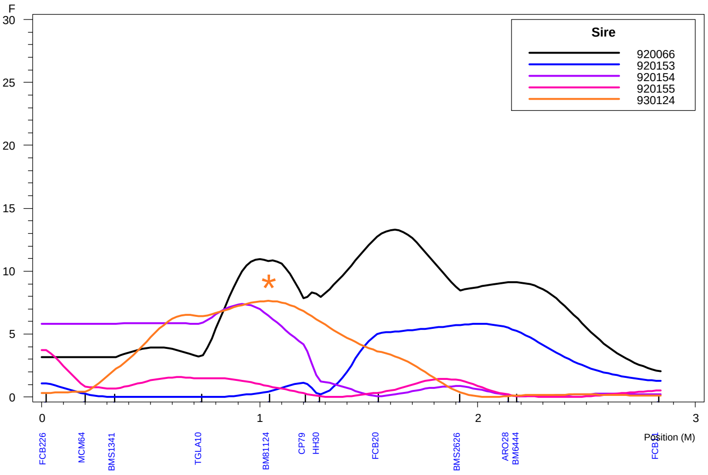

Haley-Knott QTL Analysis: Chromosome 2
LAOST

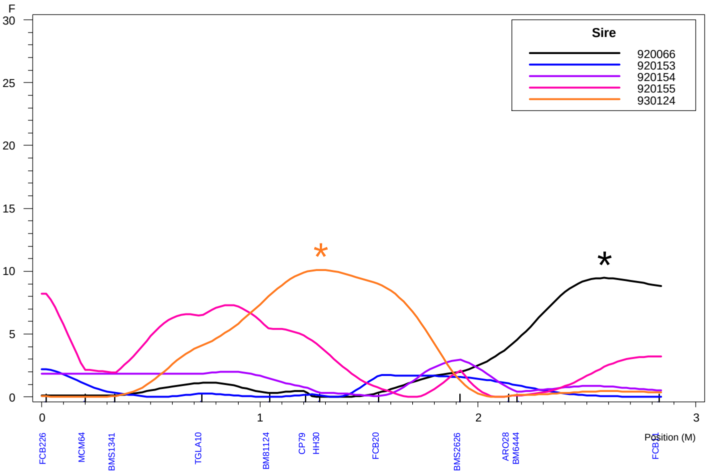

Haley-Knott QTL Analysis: Chromosome 2
LATRICH

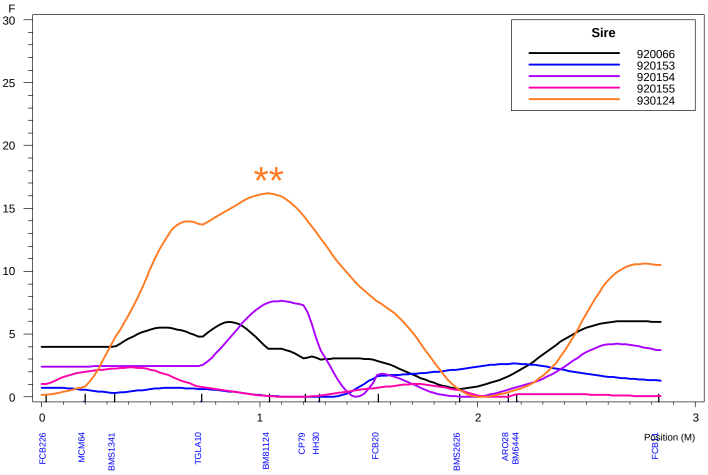

Haley-Knott QTL Analysis: Chromosome 2
DAG0

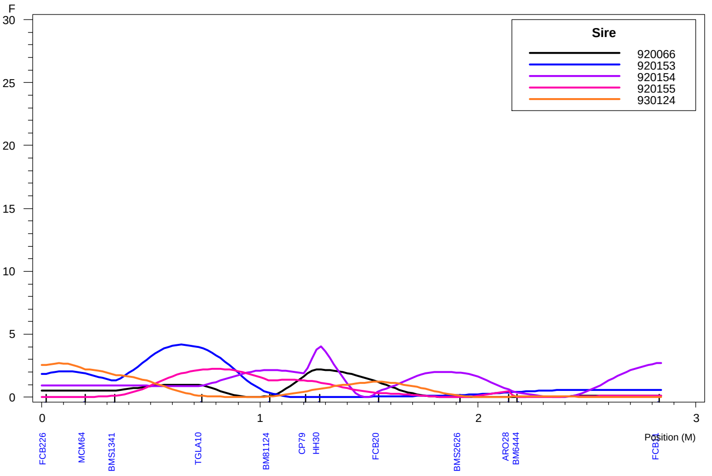

Haley-Knott QTL Analysis: Chromosome 2
DAG1

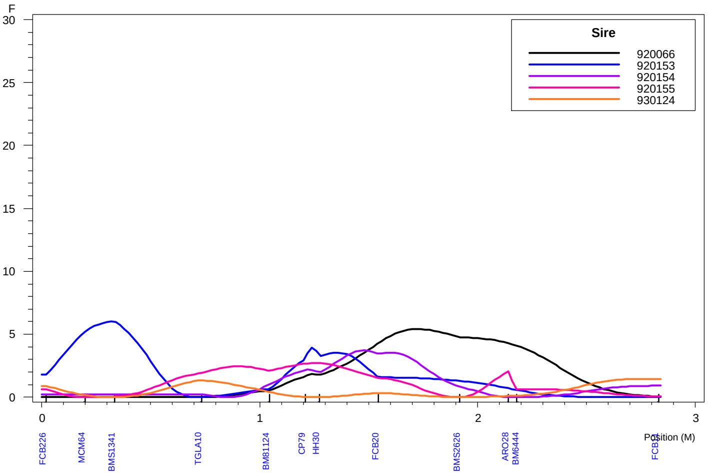

Haley-Knott QTL Analysis: Chromosome 2
DAG2

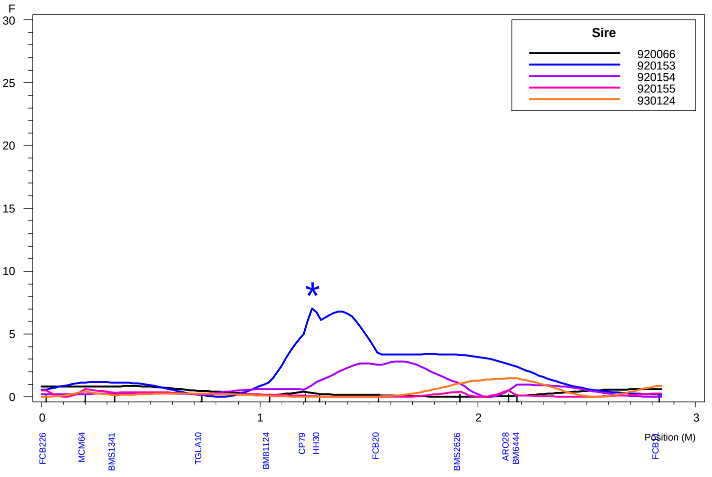

Haley-Knott QTL Analysis: Chromosome 2
ELISA1

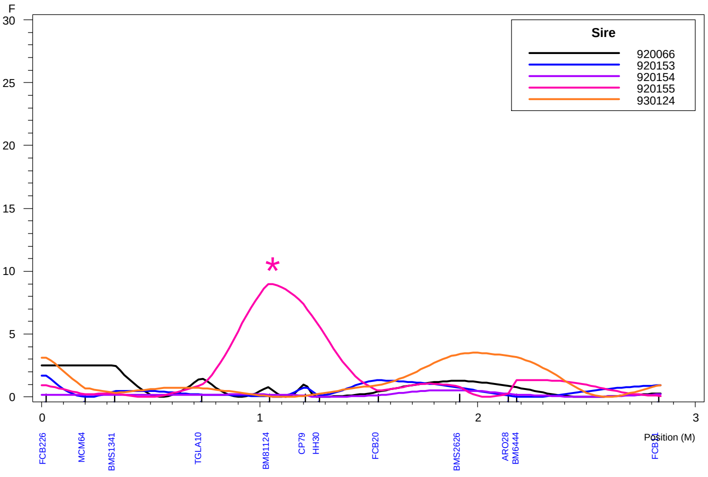

Haley-Knott QTL Analysis: Chromosome 2
ELISA2

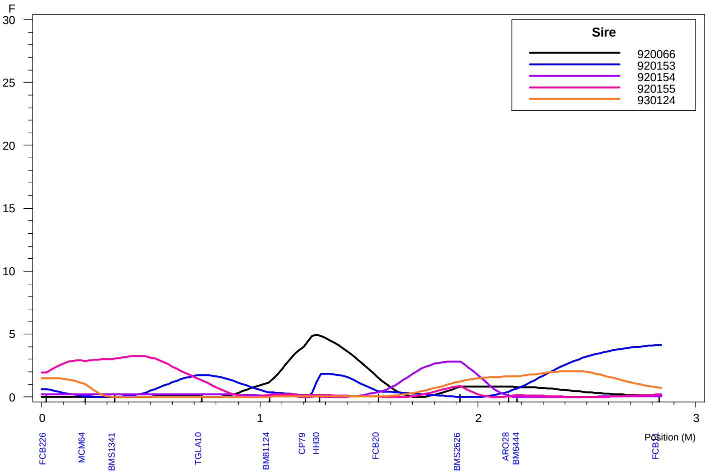

Haley-Knott QTL Analysis: Chromosome 2
ELISA3

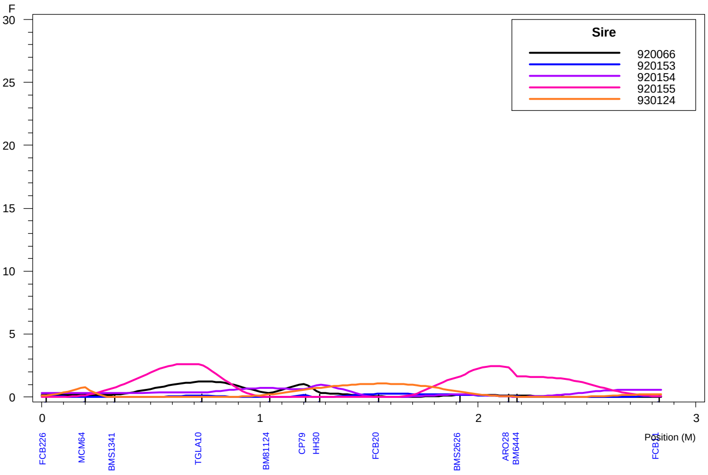

Haley-Knott QTL Analysis: Chromosome 2
ELISA4

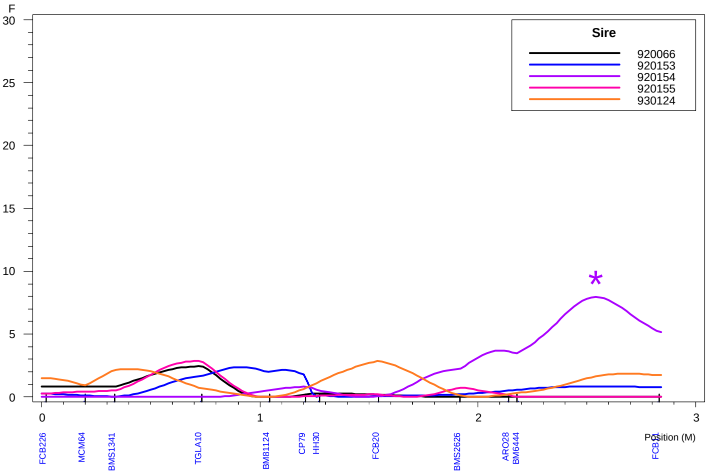

Haley-Knott QTL Analysis: Chromosome 2
ELISA5

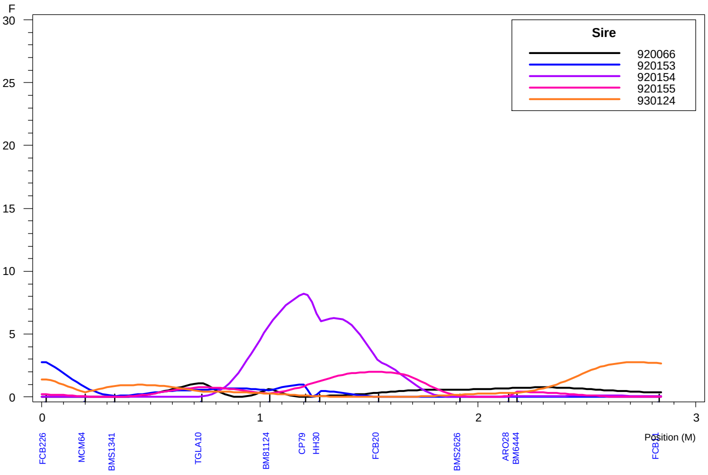

Haley-Knott QTL Analysis: Chromosome 2
LIGE

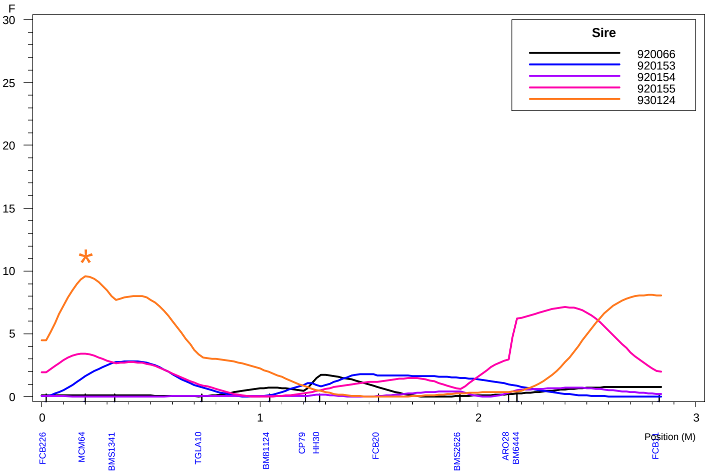

Haley-Knott QTL Analysis: Chromosome 2
WTFEC2

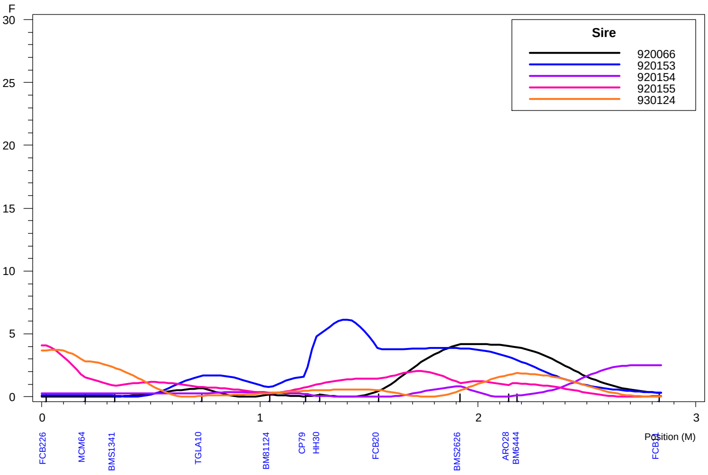

Supplement: Additional File 7 — Chr 2. Haley Knott linkage analysis of sheep chromosome 2. [file 1471-2164-7-178-S7.pdf]
