# Supplementary material for: Discovery of quantitative trait loci for resistance to parasitic nematode infection in sheep: I. Analysis of outcross pedigrees
Source: BMC Genomics. 2006 Jul 18;7:178. doi: 10.1186/1471-2164-7-178 (PMC1574317; doi:10.1186/1471-2164-7-178)

# Linkage Analysis in the Parasite Outcross Flock: Chromosome 3

Information Content: Chromosome 3

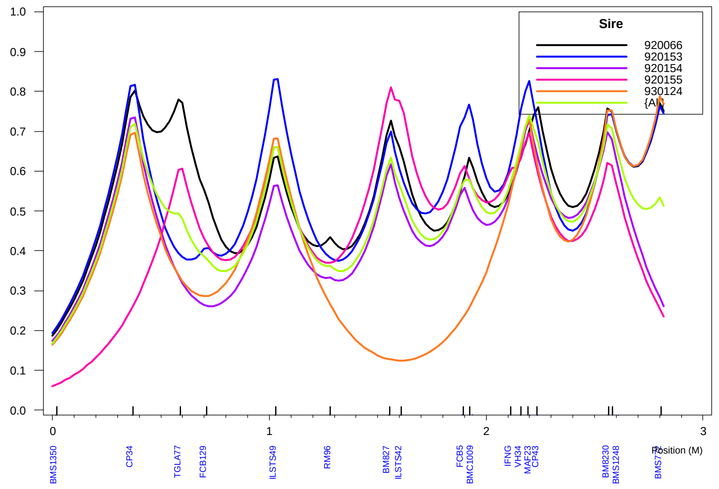


Haley-Knott QTL Analysis: Chromosome 3

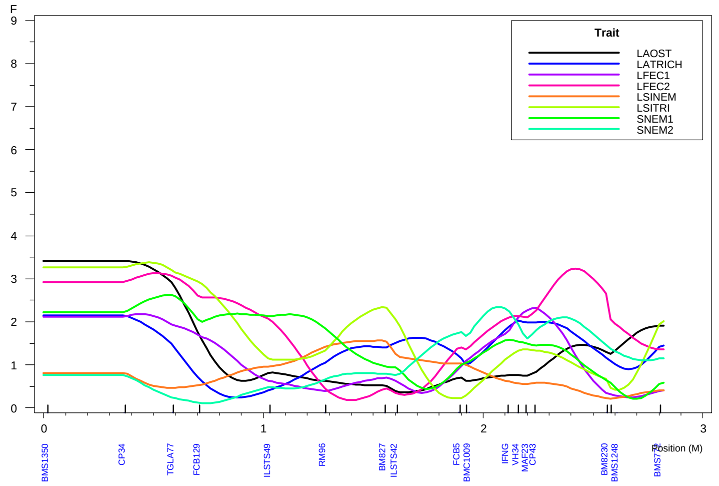


Haley-Knott QTL Analysis: Chromosome 3

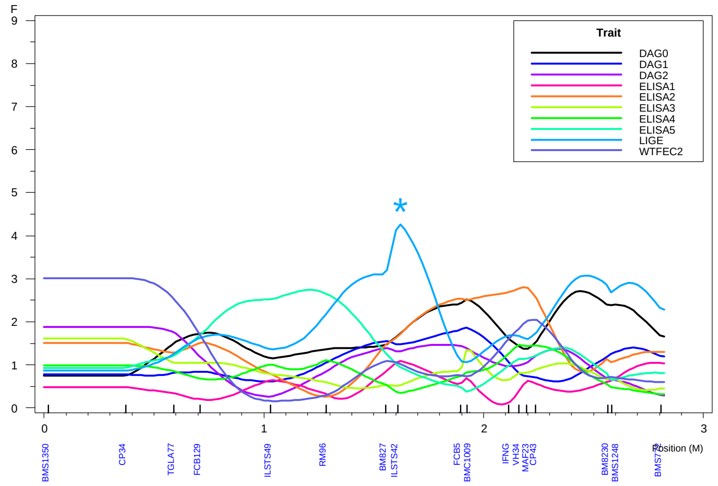


Haley-Knott QTL Analysis: Chromosome 3
LFEC1

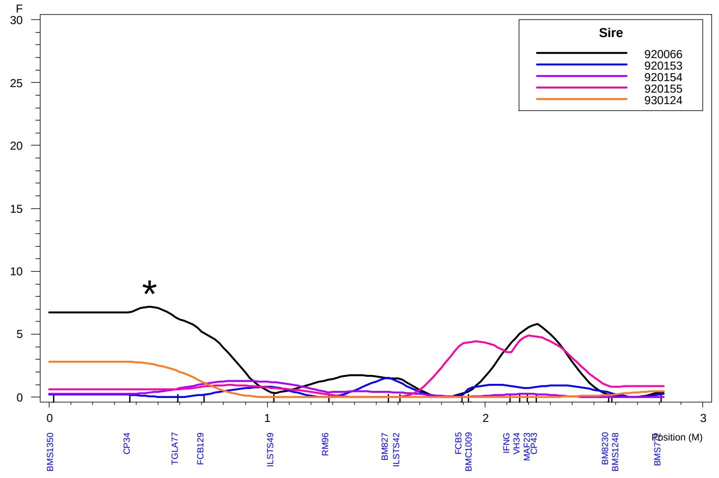


Haley-Knott QTL Analysis: Chromosome 3
LFEC2

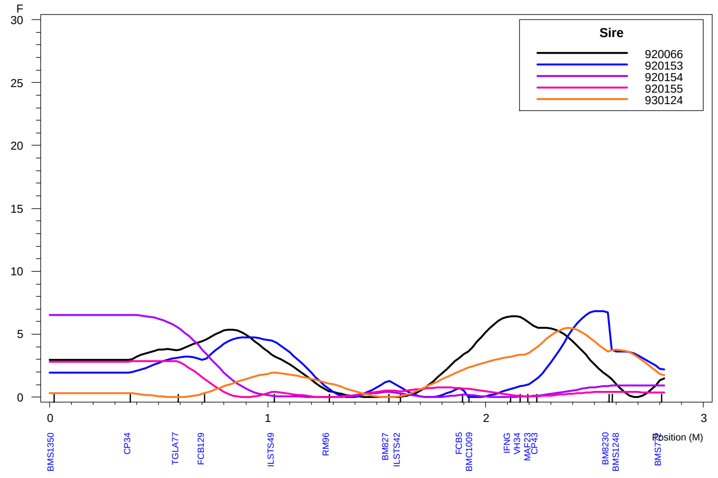


Haley-Knott QTL Analysis: Chromosome 3
SNEM1

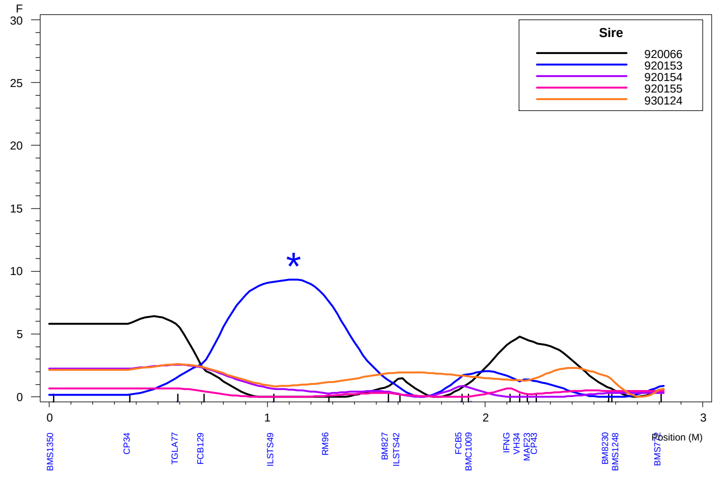


Haley-Knott QTL Analysis: Chromosome 3
SNEM2

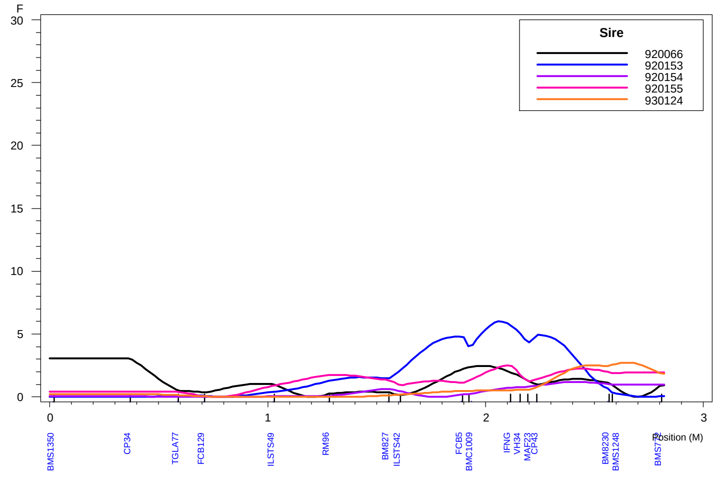

Haley-Knott QTL Analysis: Chromosome 3
LSINEM

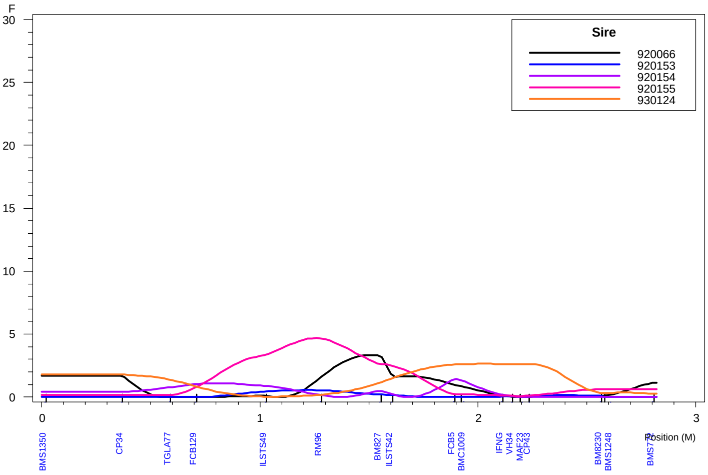


Haley-Knott QTL Analysis: Chromosome 3
LSITRI

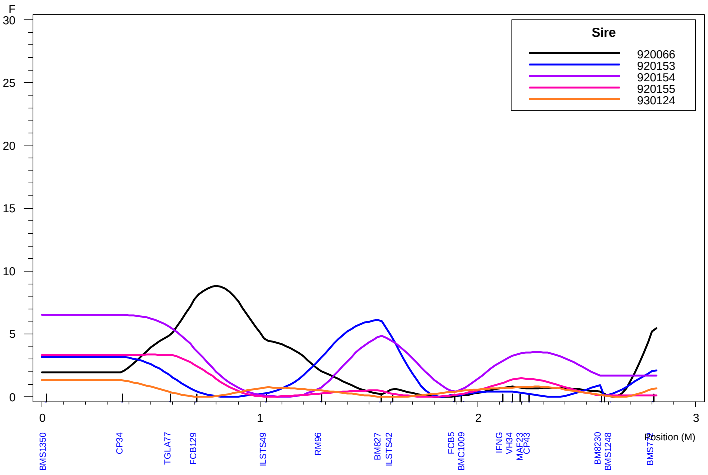


Haley-Knott QTL Analysis: Chromosome 3
LAOST

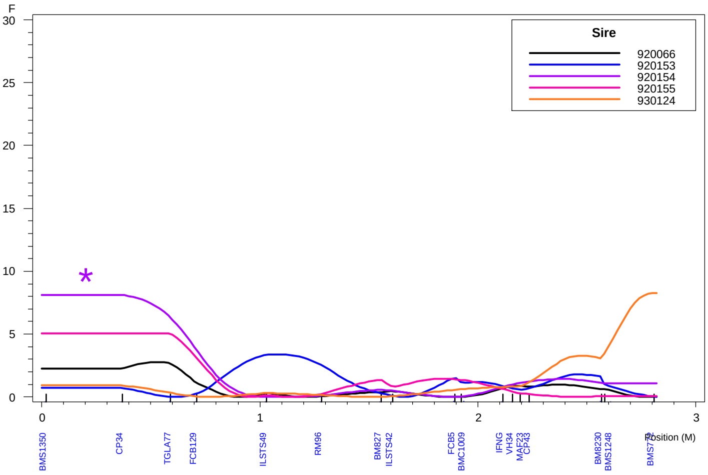


Haley-Knott QTL Analysis: Chromosome 3
LATRICH

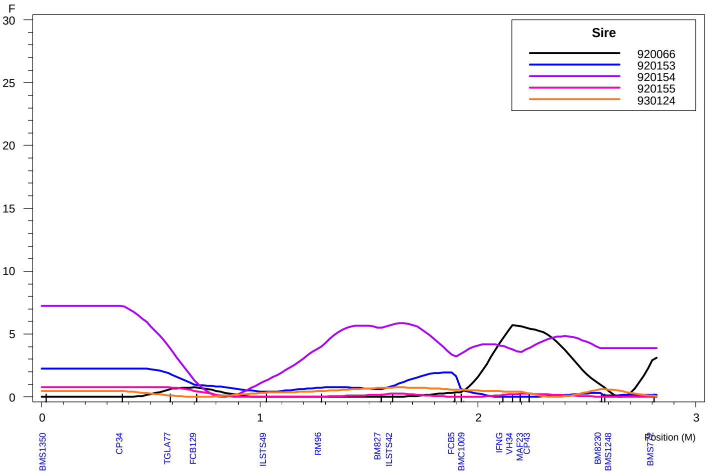


Haley-Knott QTL Analysis: Chromosome 3
DAG0

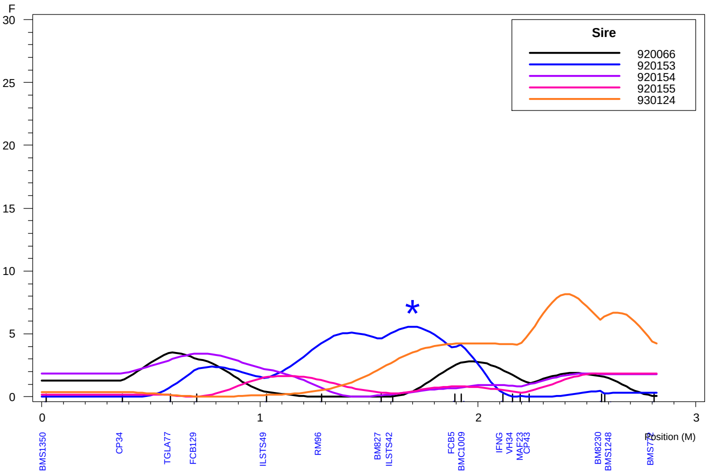


Haley-Knott QTL Analysis: Chromosome 3
DAG1

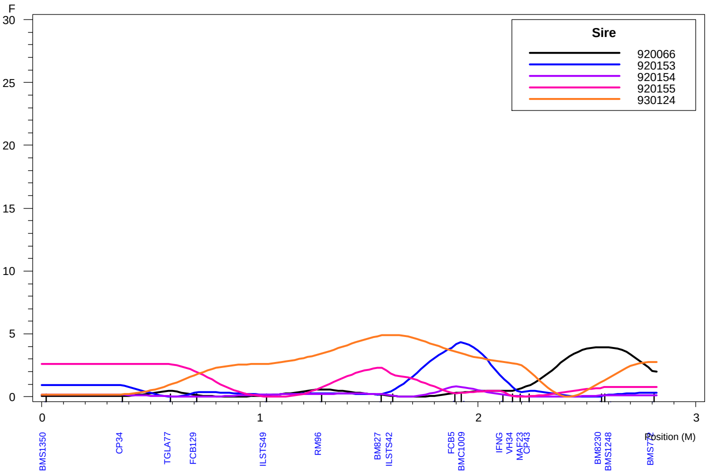


Haley-Knott QTL Analysis: Chromosome 3
DAG2

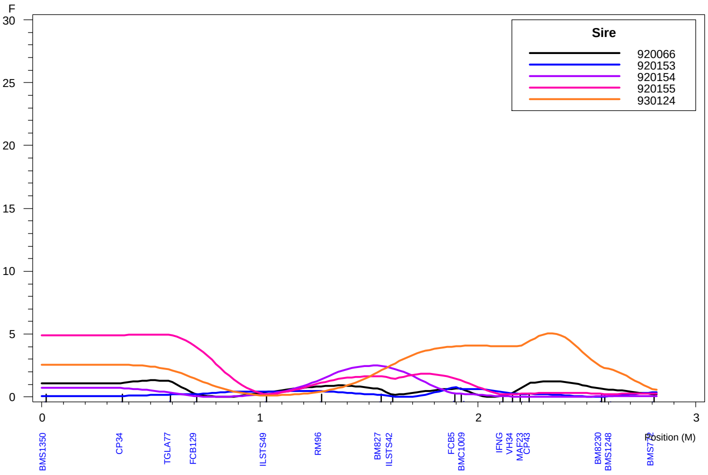


Haley-Knott QTL Analysis: Chromosome 3
ELISA1

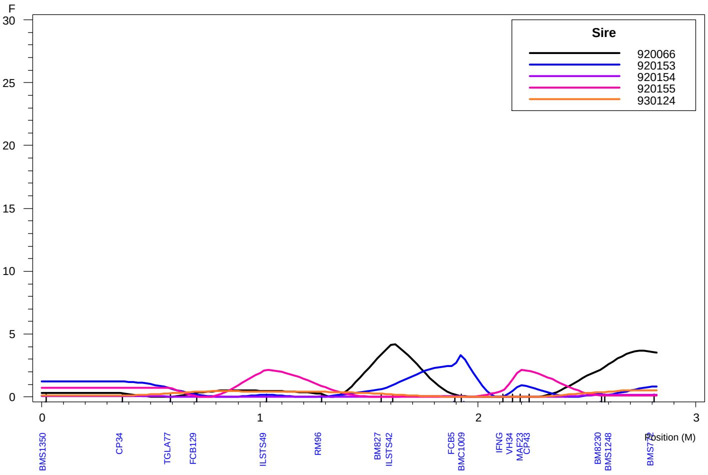

Haley-Knott QTL Analysis: Chromosome 3
ELISA2

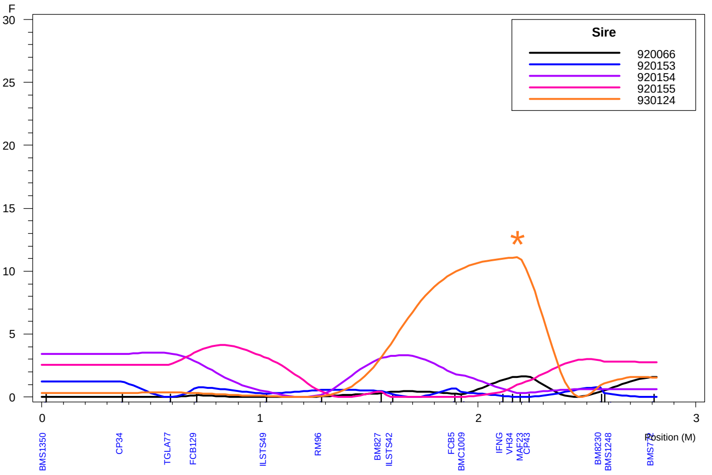


Haley-Knott QTL Analysis: Chromosome 3
ELISA3

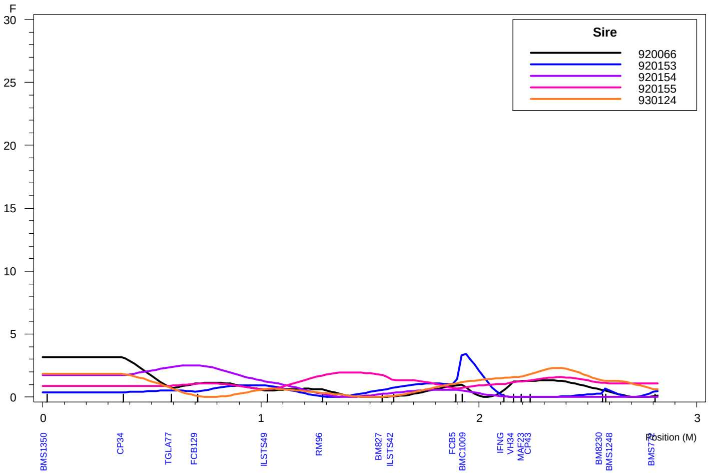


Haley-Knott QTL Analysis: Chromosome 3
ELISA4

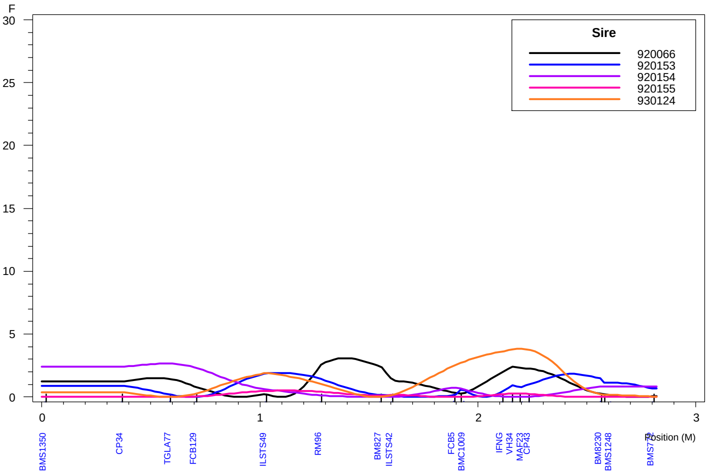


Haley-Knott QTL Analysis: Chromosome 3
ELISA5

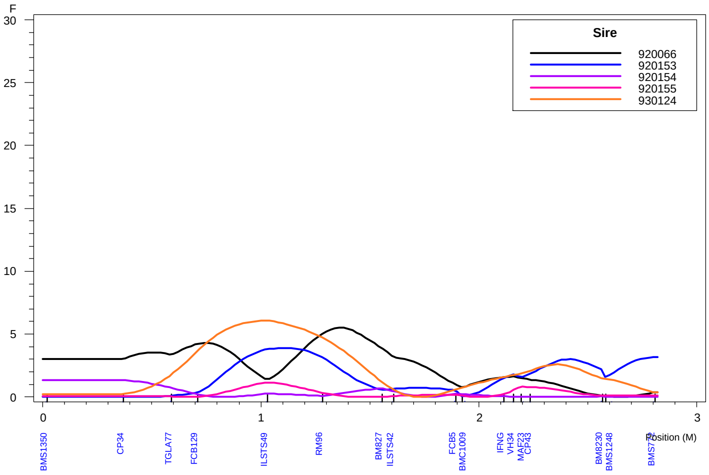


Haley-Knott QTL Analysis: Chromosome 3
LIGE

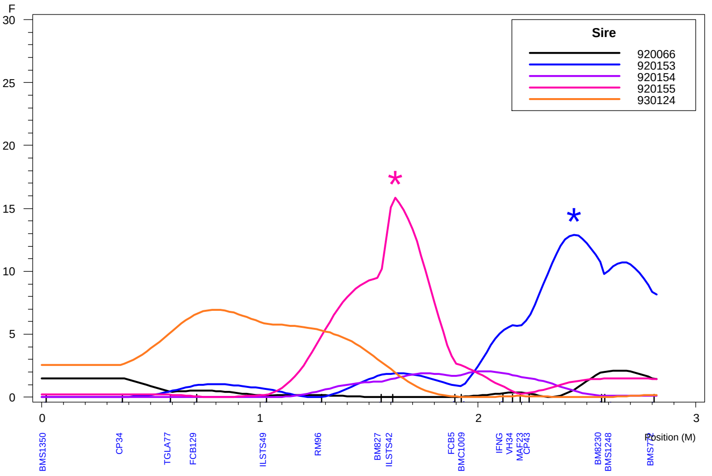


Haley-Knott QTL Analysis: Chromosome 3
WTFEC2

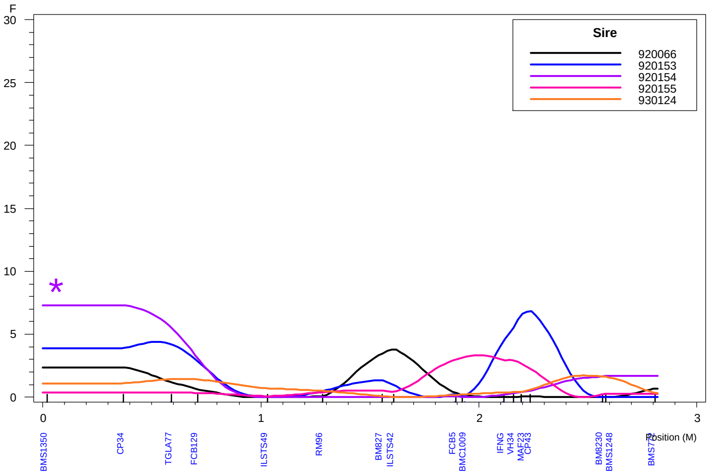

Supplement: Additional File 8 — Chr 3. Haley Knott linkage analysis of sheep chromosome 3. [file 1471-2164-7-178-S8.pdf]
