# Supplementary material for: Discovery of quantitative trait loci for resistance to parasitic nematode infection in sheep: I. Analysis of outcross pedigrees
Source: BMC Genomics. 2006 Jul 18;7:178. doi: 10.1186/1471-2164-7-178 (PMC1574317; doi:10.1186/1471-2164-7-178)

Linkage Analysis in the Parasite Outcross Flock: Chromosome 4

Information Content: Chromosome 4

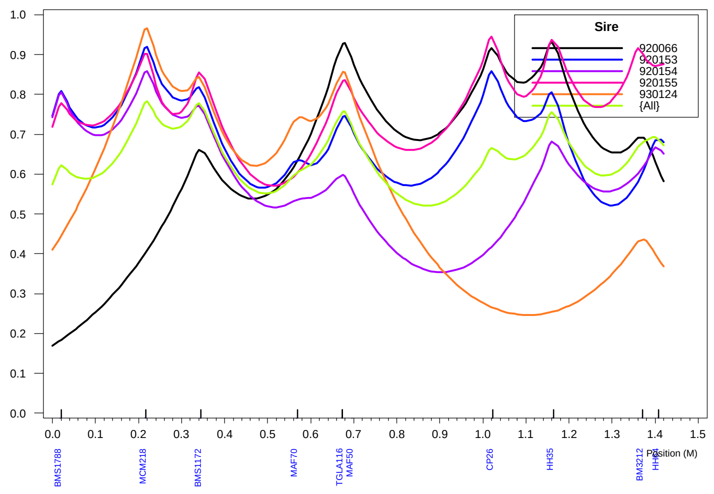

Haley-Knott QTL Analysis: Chromosome 4

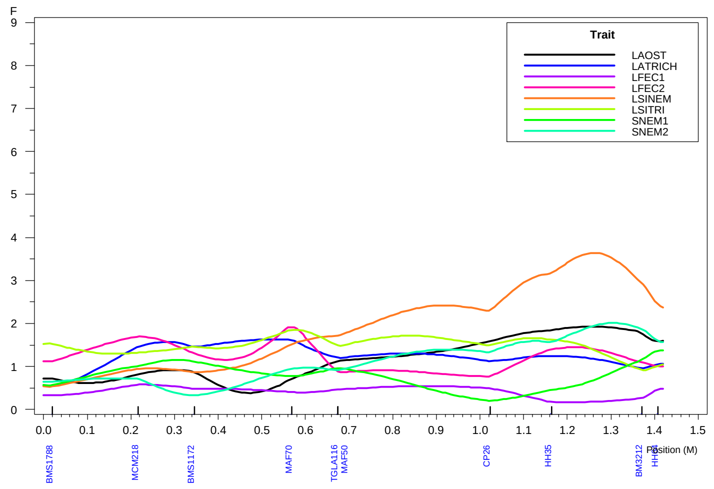

Haley-Knott QTL Analysis: Chromosome 4

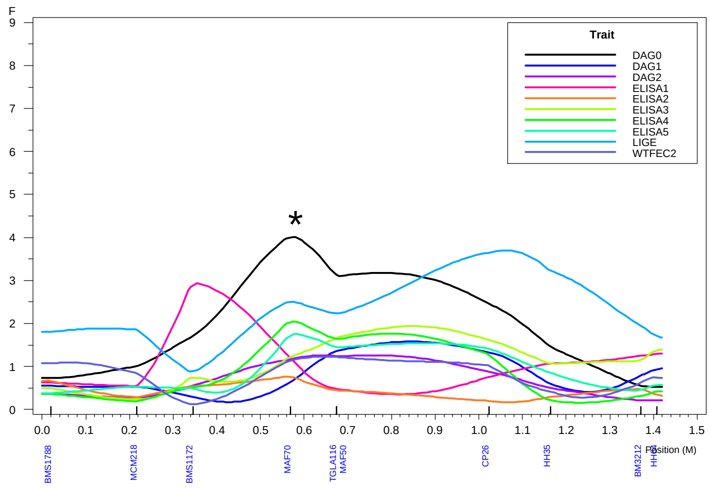

Haley-Knott QTL Analysis: Chromosome 4
LFEC1

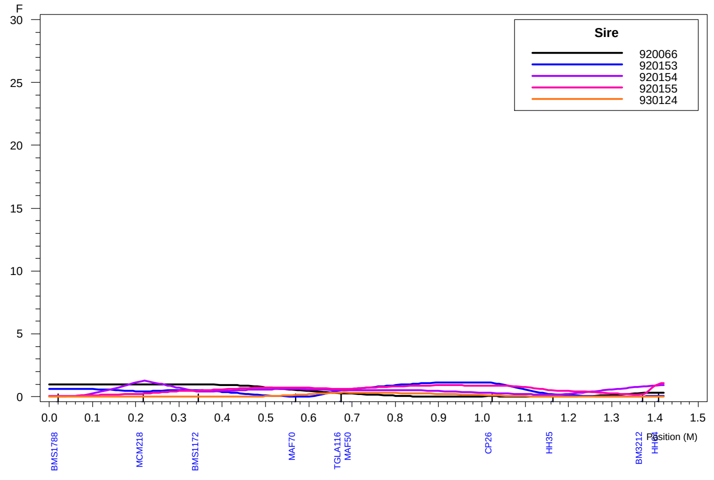

Haley-Knott QTL Analysis: Chromosome 4
LFEC2

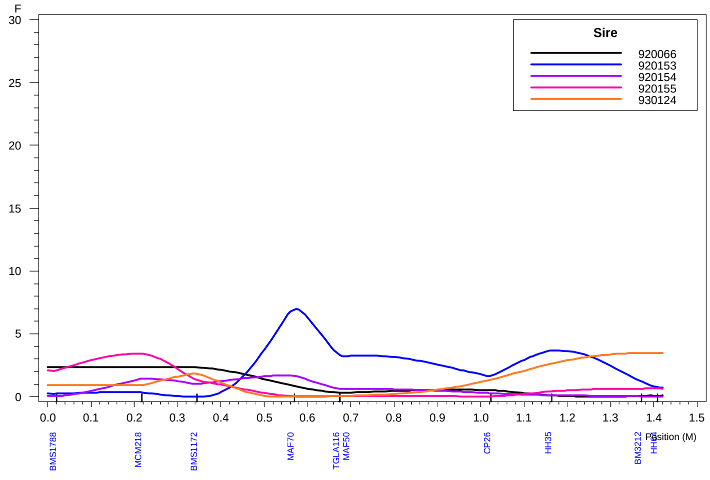

Haley-Knott QTL Analysis: Chromosome 4
SNEM1

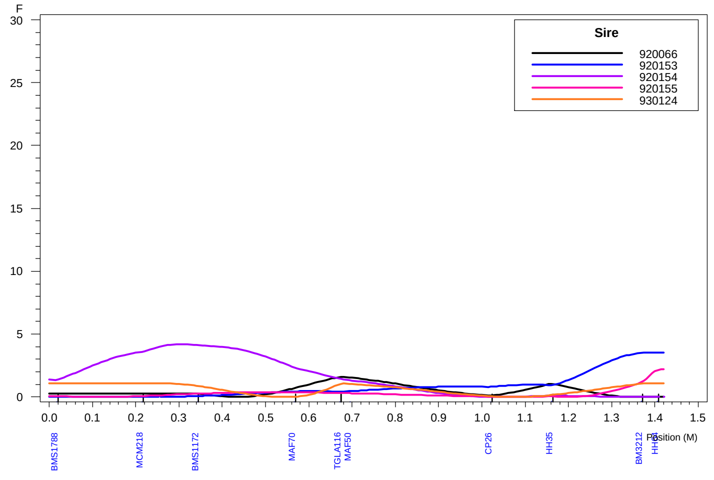

Haley-Knott QTL Analysis: Chromosome 4
SNEM2

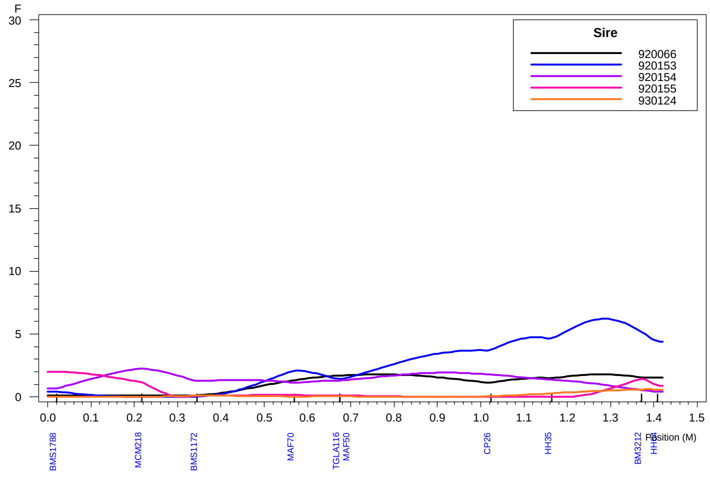

Haley-Knott QTL Analysis: Chromosome 4
LSINEM

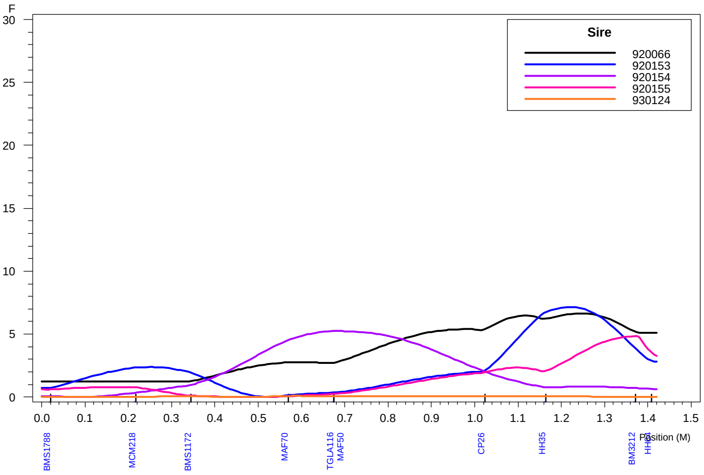

Haley-Knott QTL Analysis: Chromosome 4
LSITRI

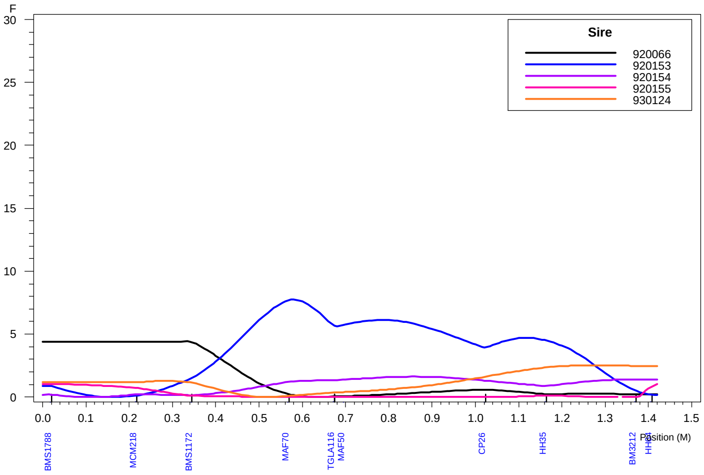

Haley-Knott QTL Analysis: Chromosome 4
LAOST

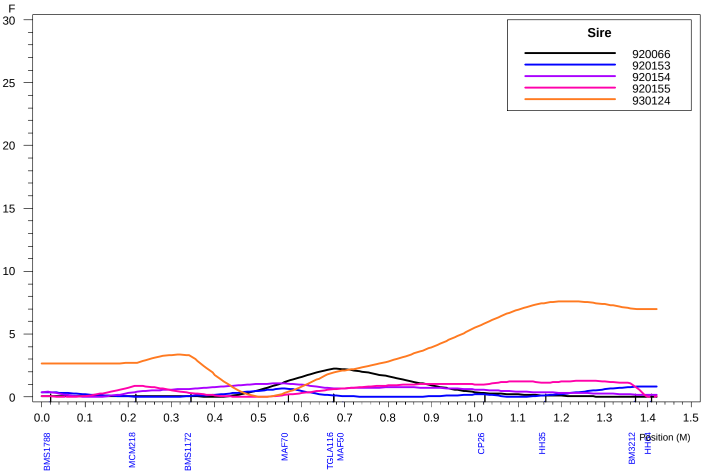

Haley-Knott QTL Analysis: Chromosome 4
LATRICH

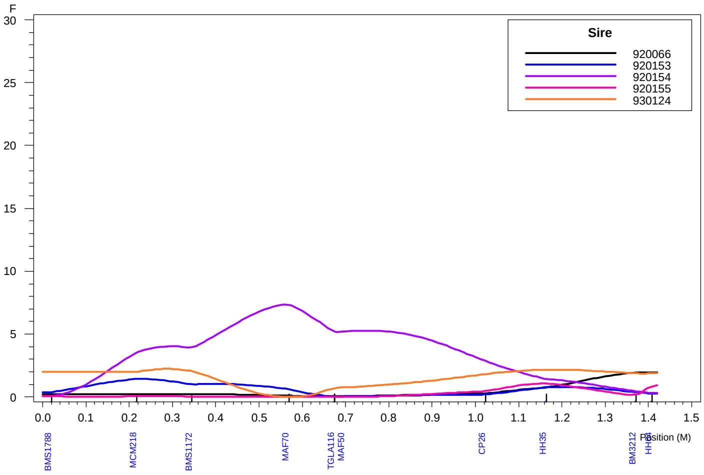

Haley-Knott QTL Analysis: Chromosome 4
DAG0

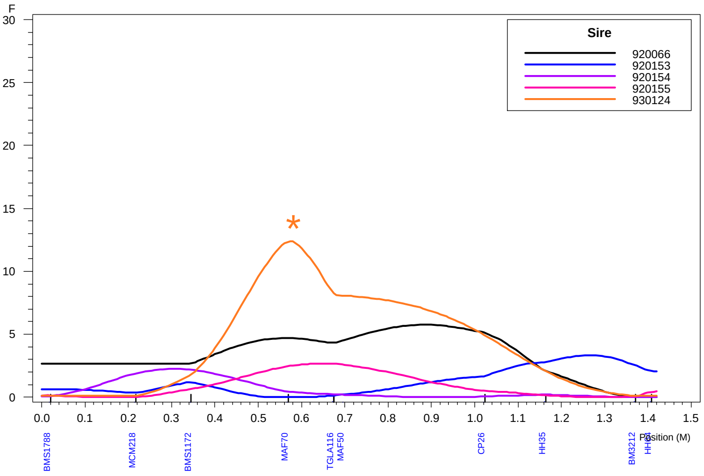

Haley-Knott QTL Analysis: Chromosome 4
DAG1

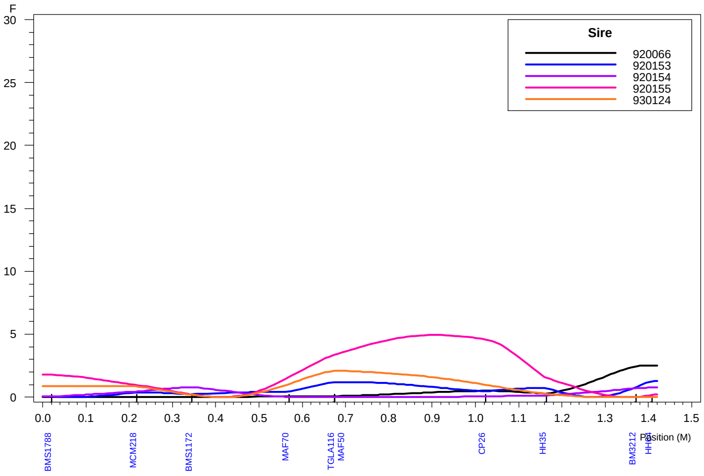

Haley-Knott QTL Analysis: Chromosome 4
DAG2

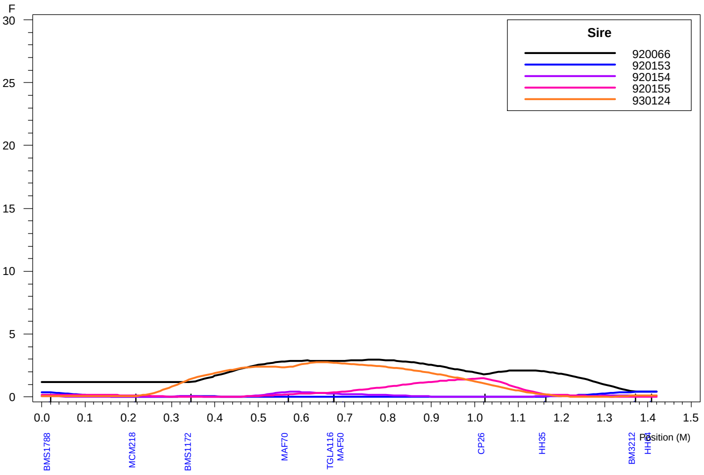

Haley-Knott QTL Analysis: Chromosome 4
ELISA1

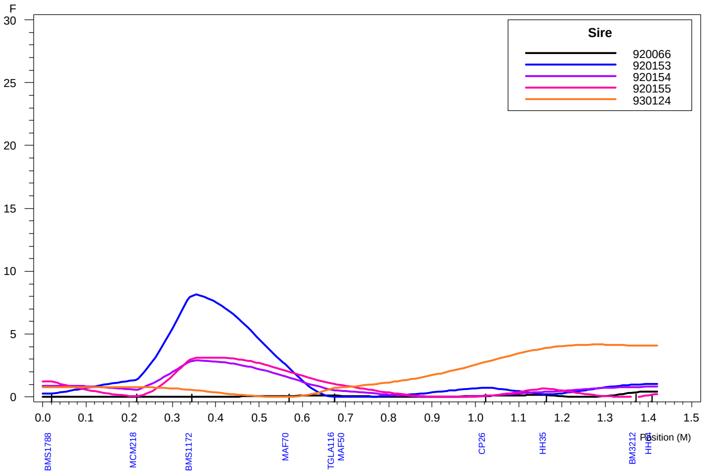

Haley-Knott QTL Analysis: Chromosome 4
ELISA2

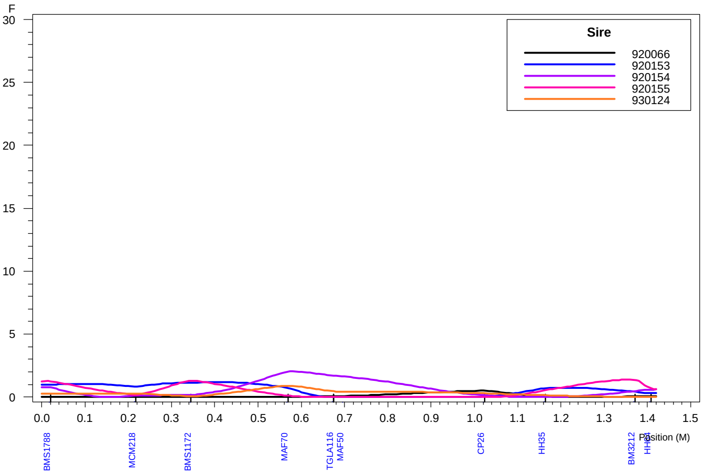

Haley-Knott QTL Analysis: Chromosome 4
ELISA3

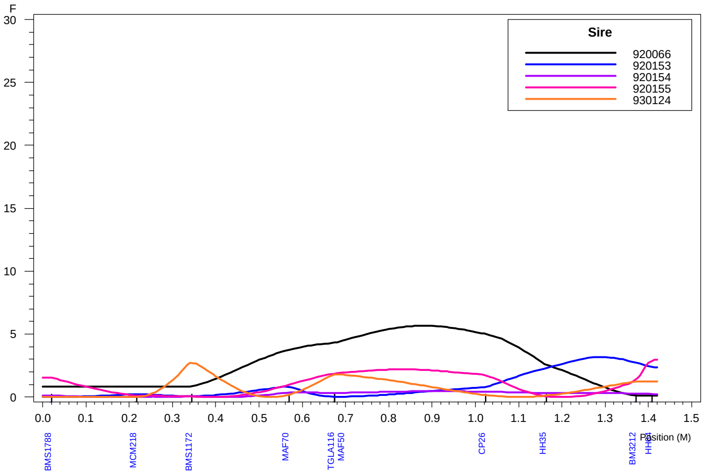

Haley-Knott QTL Analysis: Chromosome 4
ELISA4

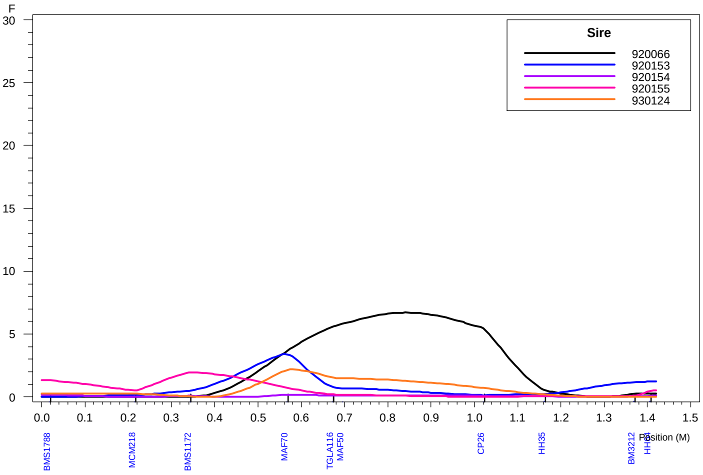

Haley-Knott QTL Analysis: Chromosome 4
ELISA5

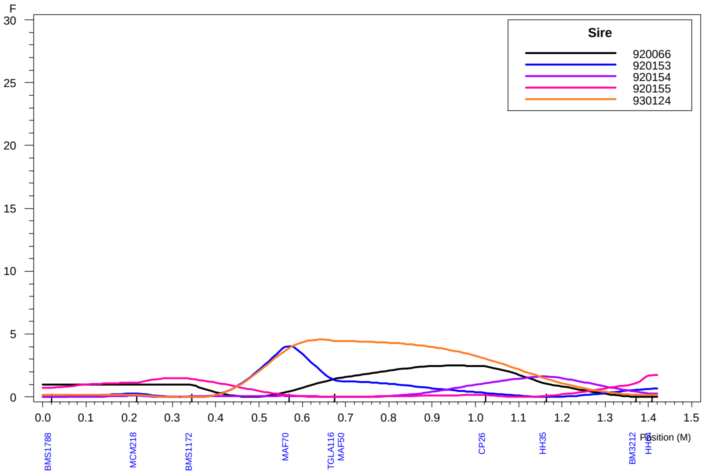

Haley-Knott QTL Analysis: Chromosome 4
LIGE

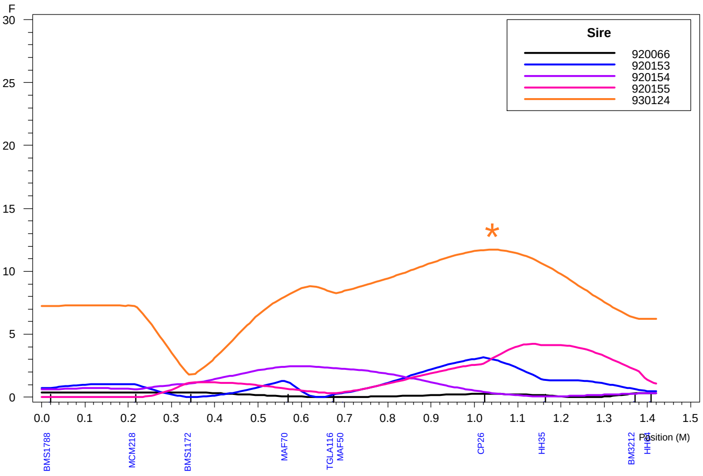

Haley-Knott QTL Analysis: Chromosome 4
WTFEC2

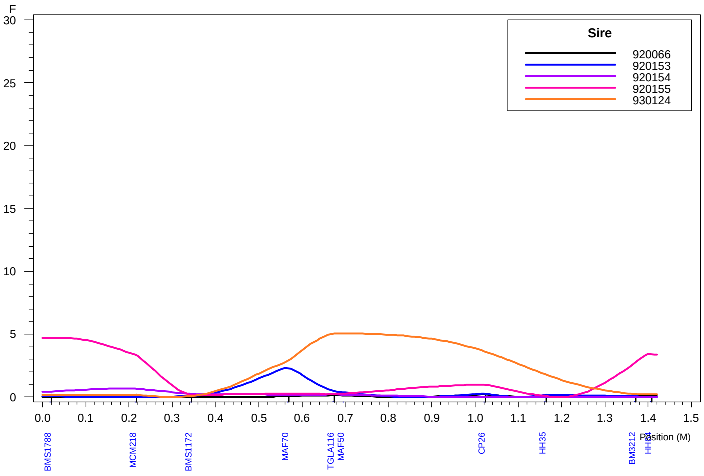

Supplement: Additional File 9 — Chr 4. Haley Knott linkage analysis of sheep chromosome 4. [file 1471-2164-7-178-S9.pdf]
