# Supplementary material for: Discovery of quantitative trait loci for resistance to parasitic nematode infection in sheep: I. Analysis of outcross pedigrees
Source: BMC Genomics. 2006 Jul 18;7:178. doi: 10.1186/1471-2164-7-178 (PMC1574317; doi:10.1186/1471-2164-7-178)

Linkage Analysis in the Parasite Outcross Flock: Chromosome 5

Information Content: Chromosome 5

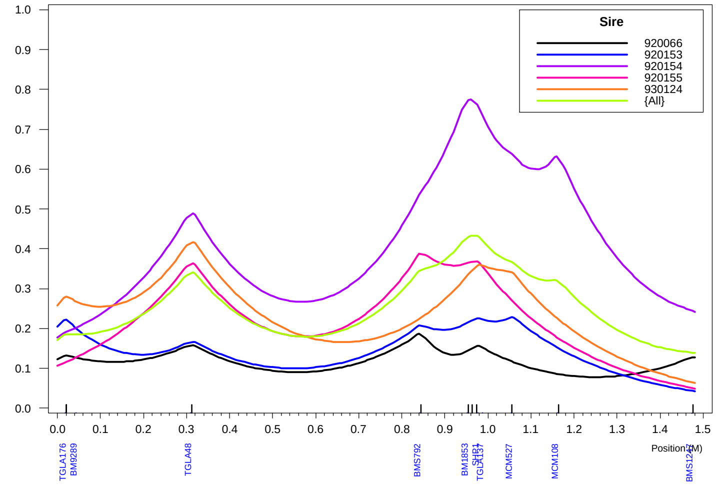

Haley-Knott QTL Analysis: Chromosome 5

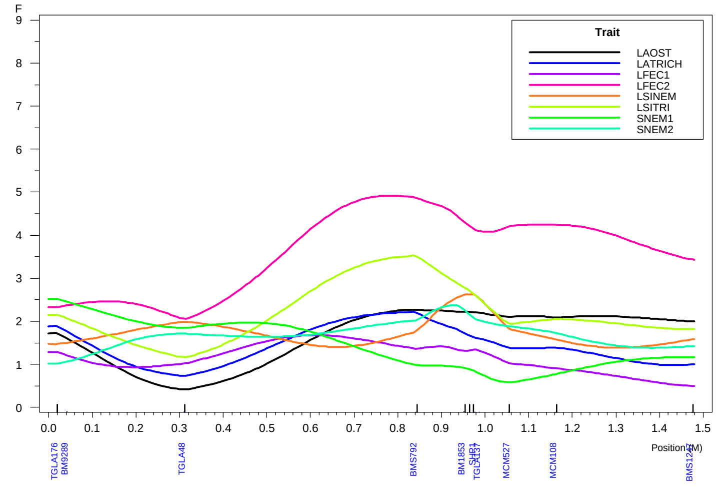

Haley-Knott QTL Analysis: Chromosome 5

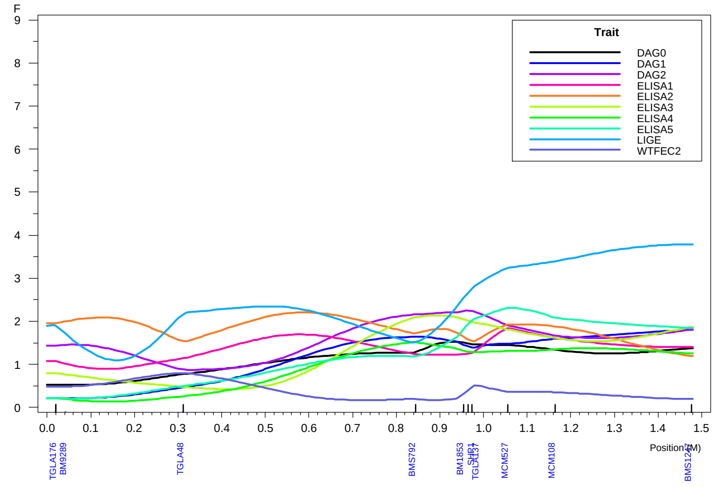

Haley-Knott QTL Analysis: Chromosome 5
LFEC1

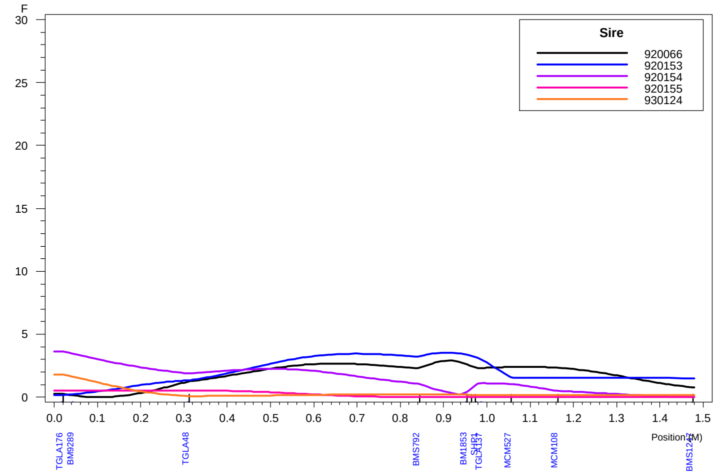

Haley-Knott QTL Analysis: Chromosome 5
LFEC2

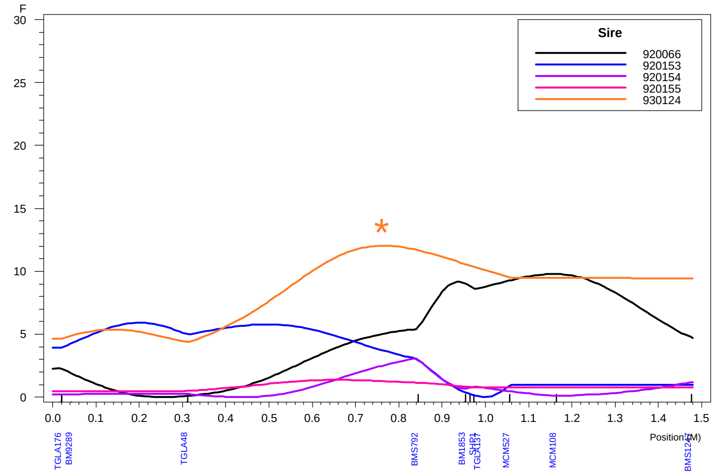

Haley-Knott QTL Analysis: Chromosome 5
SNEM1

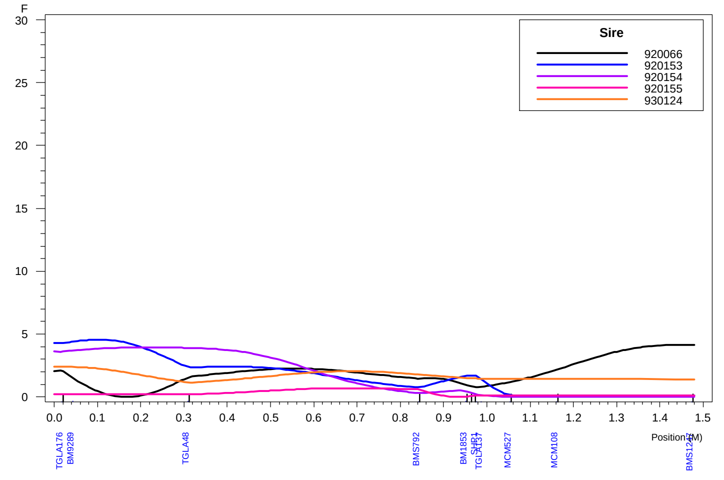

Haley-Knott QTL Analysis: Chromosome 5
SNEM2

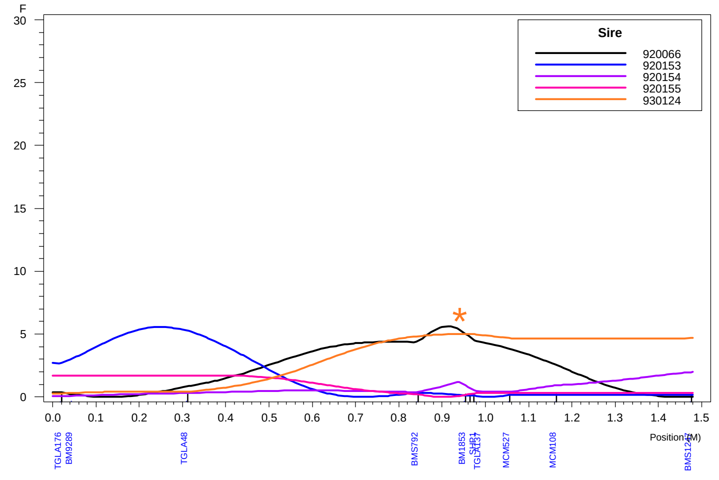

Haley-Knott QTL Analysis: Chromosome 5
LSINEM

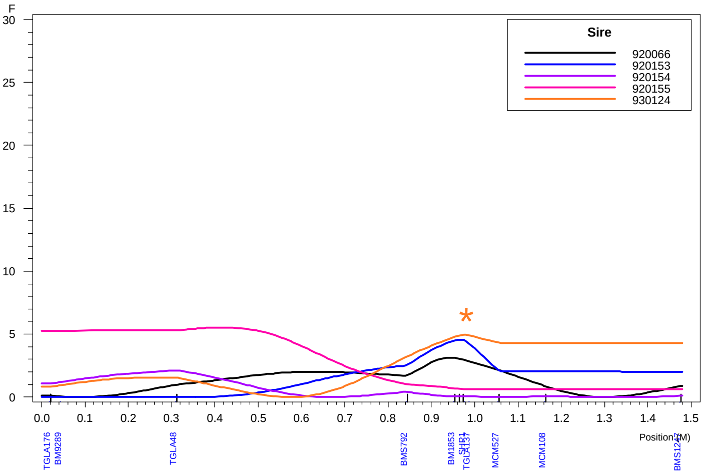

Haley-Knott QTL Analysis: Chromosome 5
LSITRI

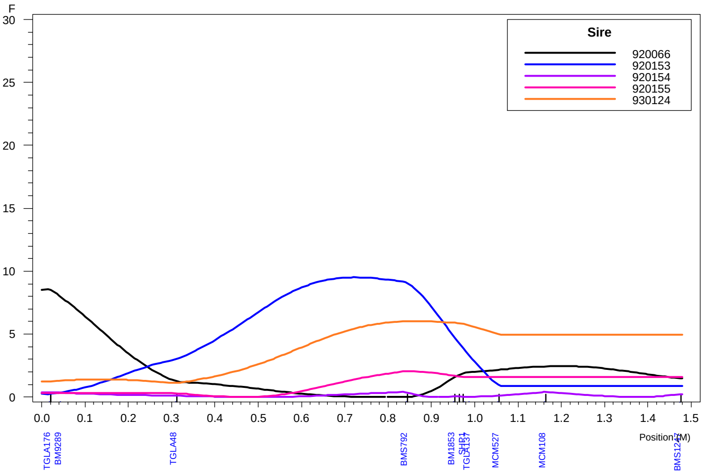

Haley-Knott QTL Analysis: Chromosome 5
LAOST

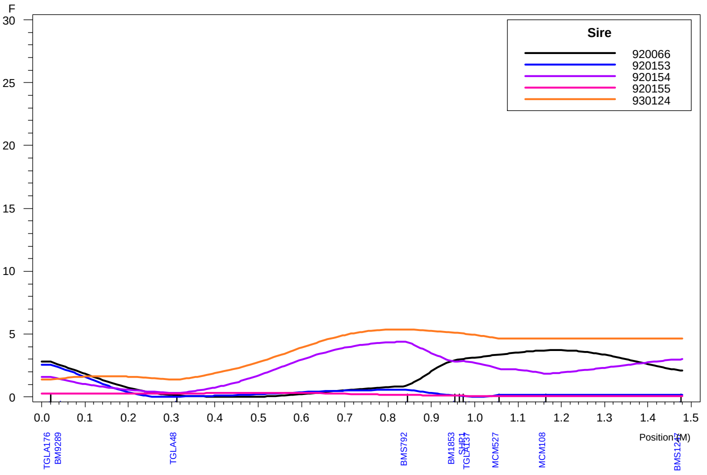

Haley-Knott QTL Analysis: Chromosome 5
LATRICH

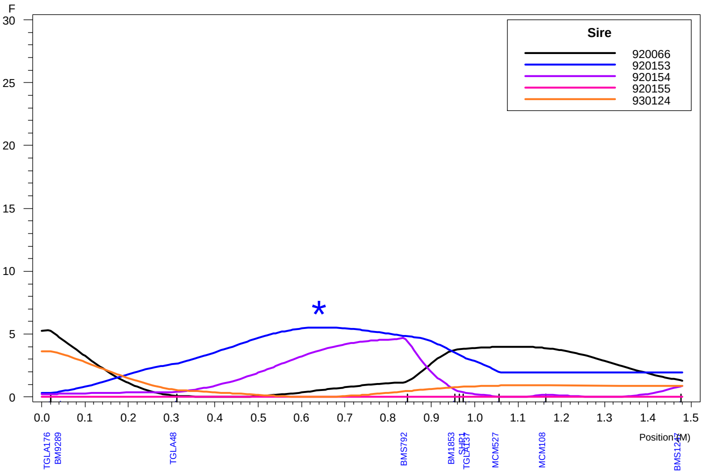

Haley-Knott QTL Analysis: Chromosome 5
DAG0

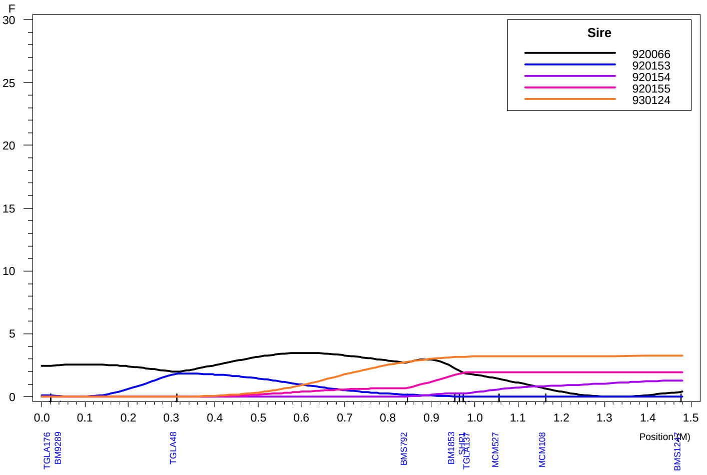

Haley-Knott QTL Analysis: Chromosome 5
DAG1

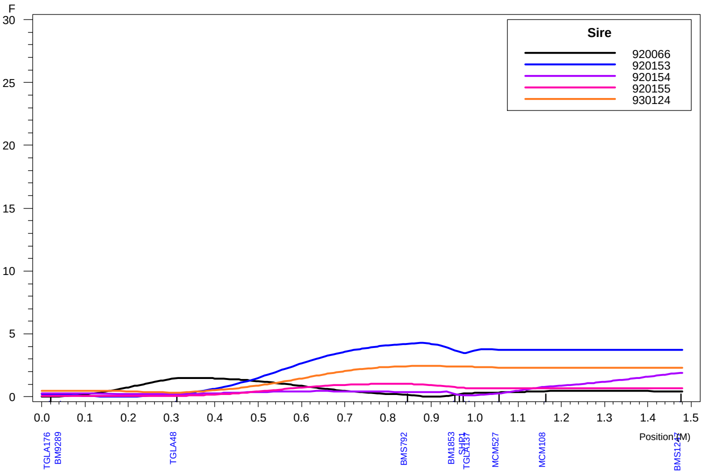

Haley-Knott QTL Analysis: Chromosome 5
DAG2

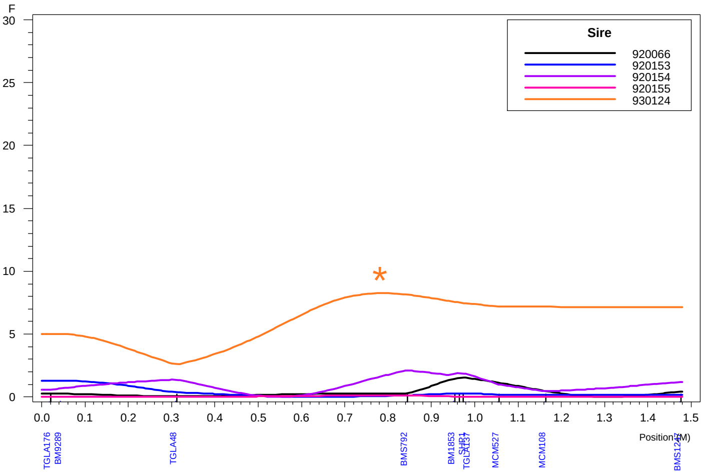

Haley-Knott QTL Analysis: Chromosome 5
ELISA1

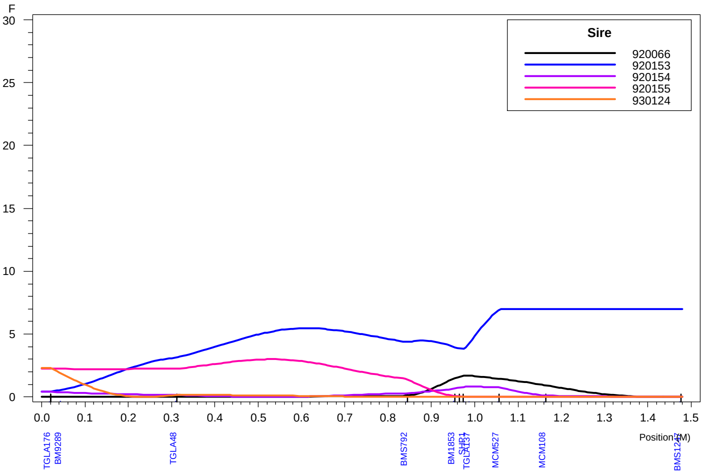

Haley-Knott QTL Analysis: Chromosome 5
ELISA2

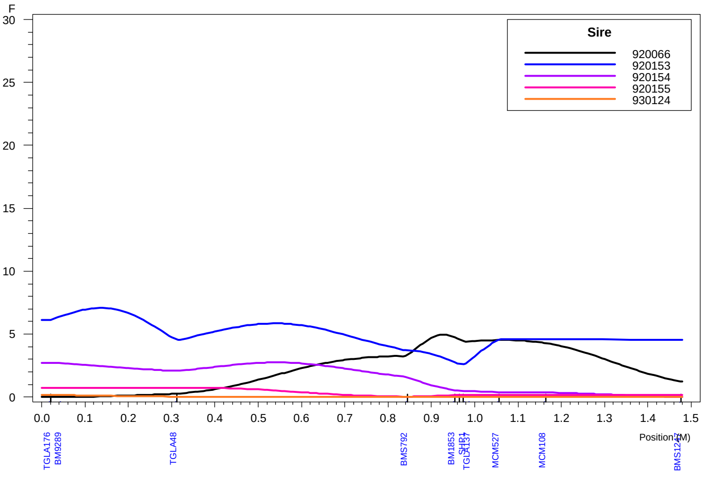

Haley-Knott QTL Analysis: Chromosome 5
ELISA3

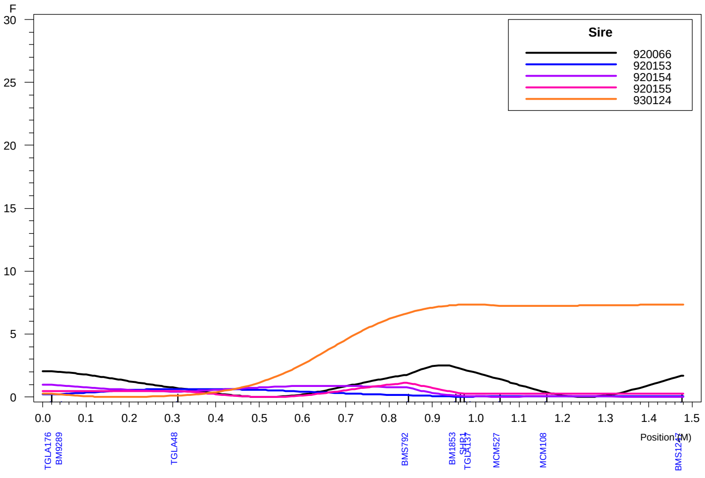

Haley-Knott QTL Analysis: Chromosome 5
ELISA4

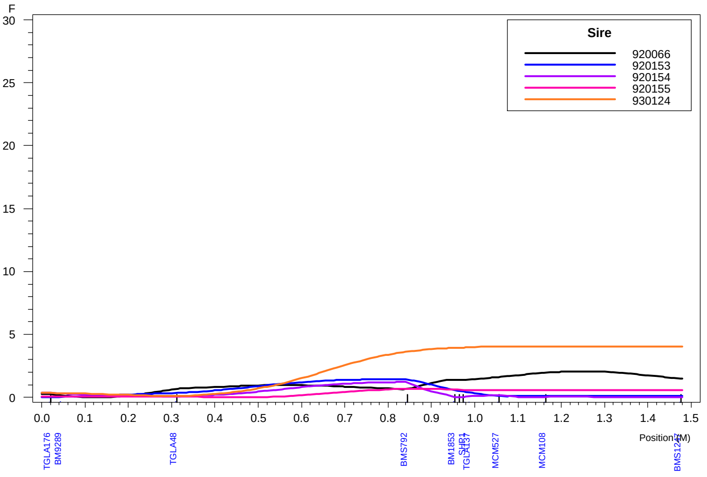

Haley-Knott QTL Analysis: Chromosome 5
ELISA5

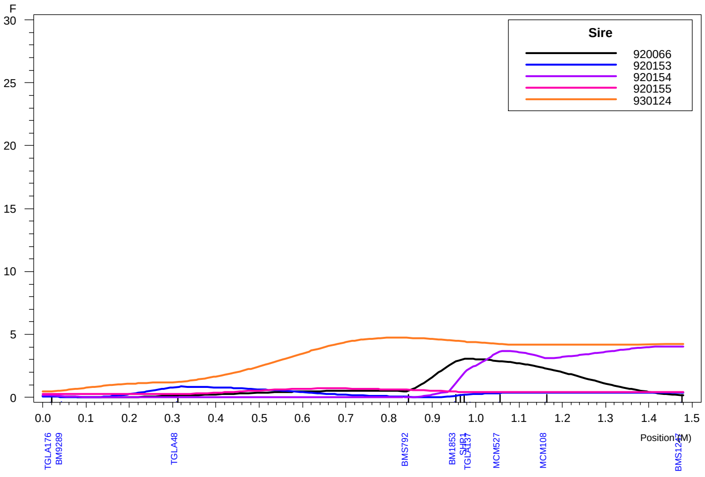

Haley-Knott QTL Analysis: Chromosome 5
LIGE

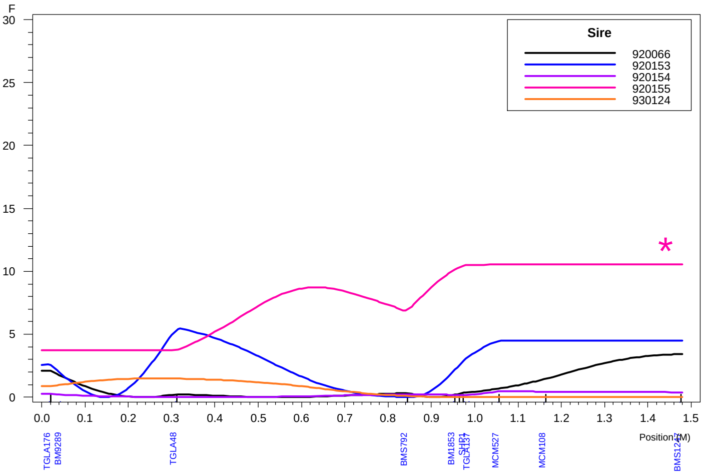

Haley-Knott QTL Analysis: Chromosome 5
WTFEC2

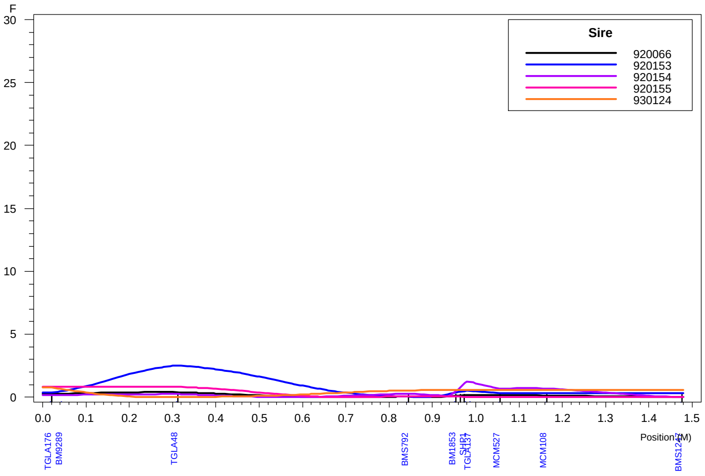

Supplement: Additional File 10 — Chr 5. Haley Knott linkage analysis of sheep chromosome 5. [file 1471-2164-7-178-S10.pdf]
