# Supplementary material for: Discovery of quantitative trait loci for resistance to parasitic nematode infection in sheep: I. Analysis of outcross pedigrees
Source: BMC Genomics. 2006 Jul 18;7:178. doi: 10.1186/1471-2164-7-178 (PMC1574317; doi:10.1186/1471-2164-7-178)

Linkage Analysis in the Parasite Outcross Flock: Chromosome 6

Information Content: Chromosome 6

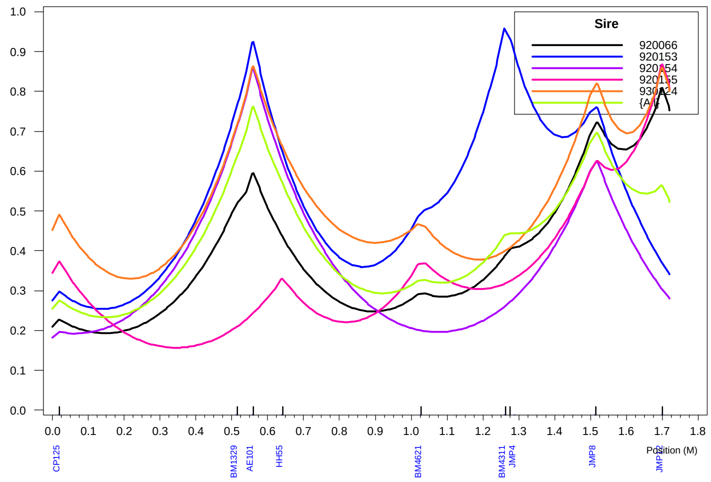

Haley-Knott QTL Analysis: Chromosome 6

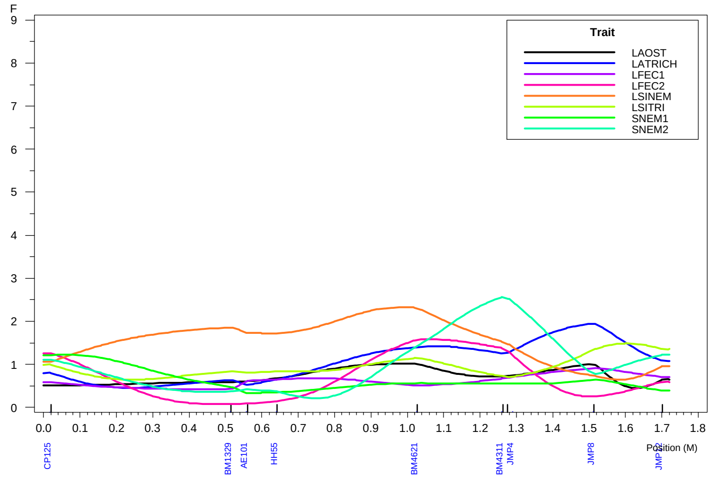

Haley-Knott QTL Analysis: Chromosome 6

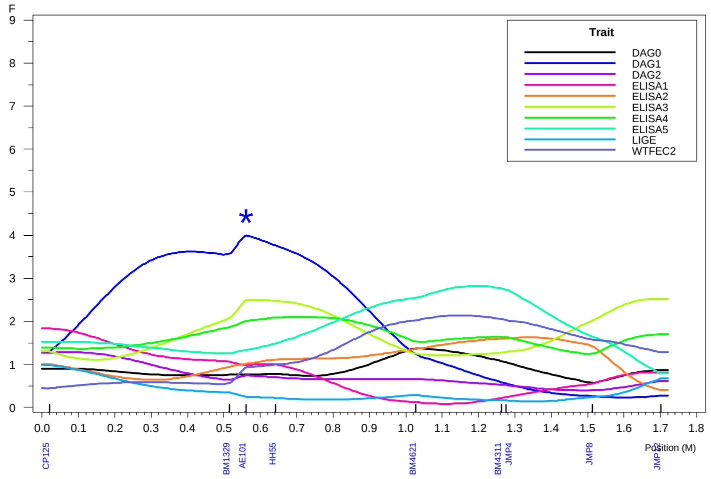

Haley-Knott QTL Analysis: Chromosome 6
LFEC1

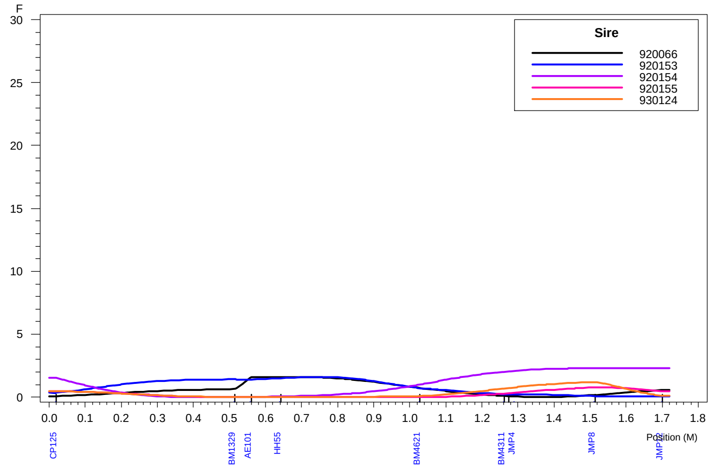

Haley-Knott QTL Analysis: Chromosome 6
LFEC2

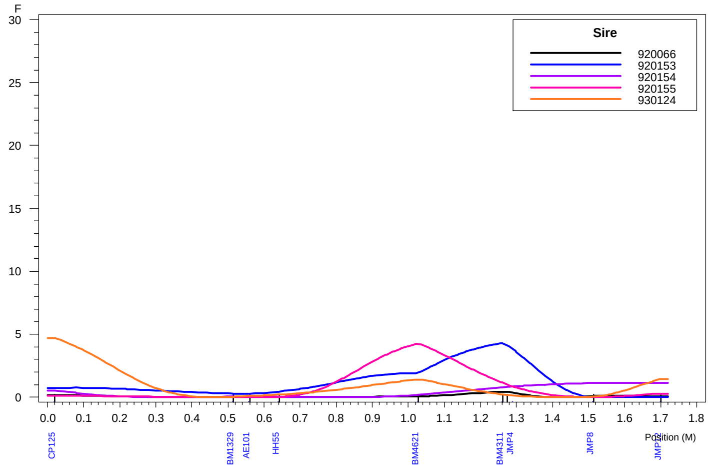

Haley-Knott QTL Analysis: Chromosome 6
SNEM1

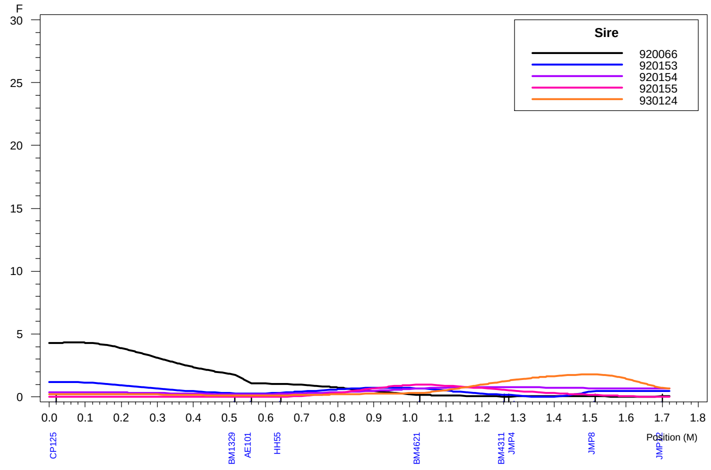

Haley-Knott QTL Analysis: Chromosome 6
SNEM2

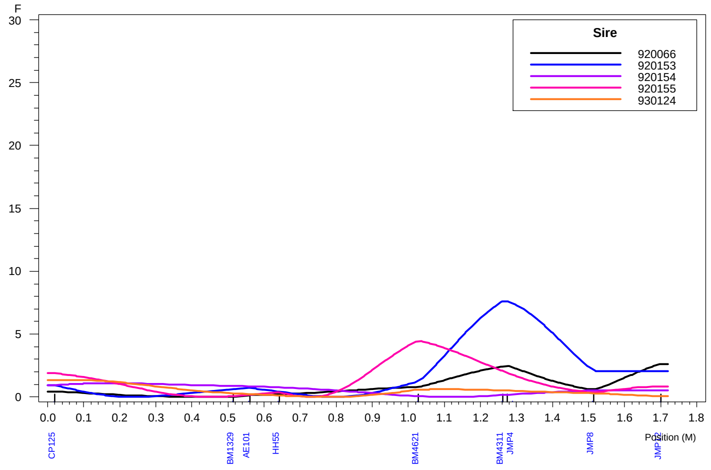

Haley-Knott QTL Analysis: Chromosome 6
LSINEM

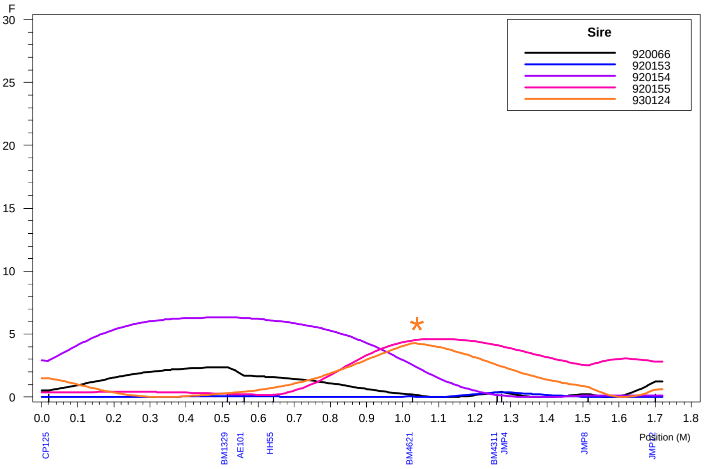

Haley-Knott QTL Analysis: Chromosome 6
LSITRI

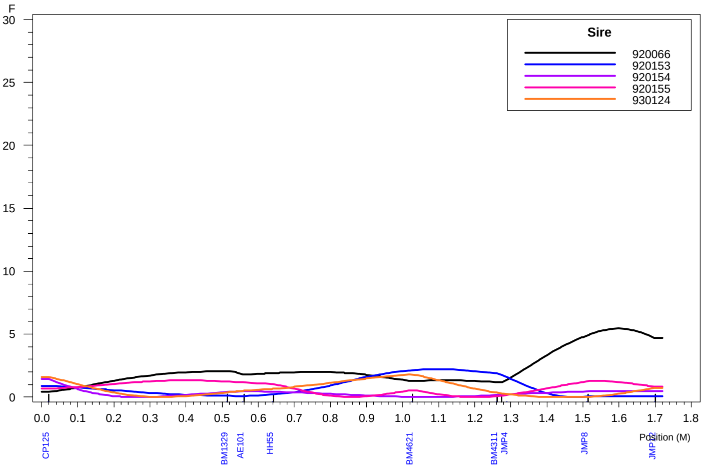

Haley-Knott QTL Analysis: Chromosome 6
LAOST

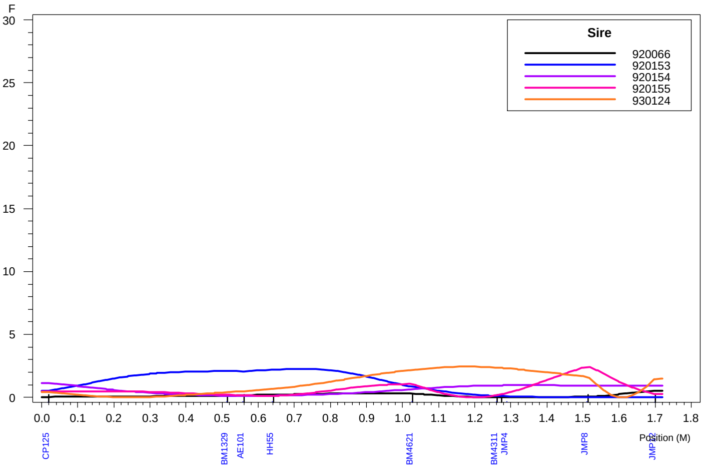

Haley-Knott QTL Analysis: Chromosome 6
LATRICH

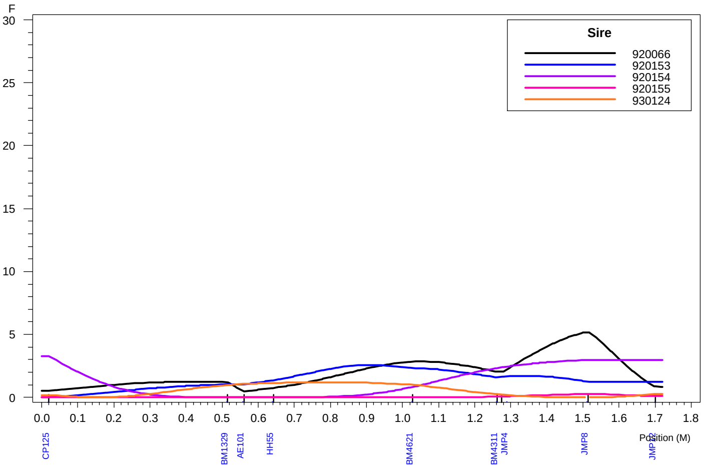

Haley-Knott QTL Analysis: Chromosome 6
DAG0

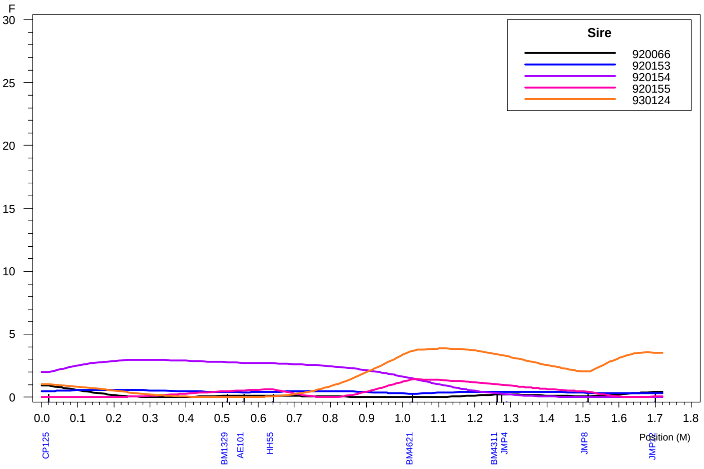

Haley-Knott QTL Analysis: Chromosome 6
DAG1

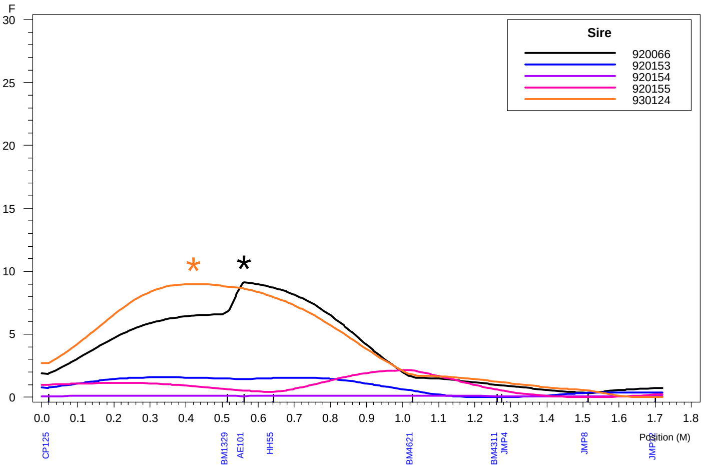

Haley-Knott QTL Analysis: Chromosome 6
DAG2

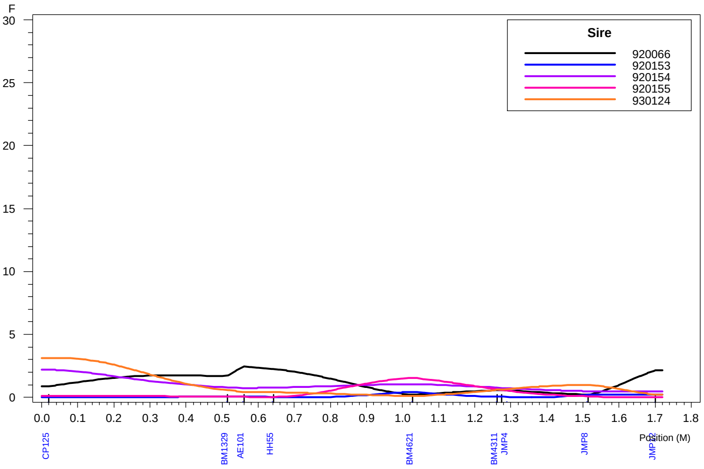

Haley-Knott QTL Analysis: Chromosome 6
ELISA1

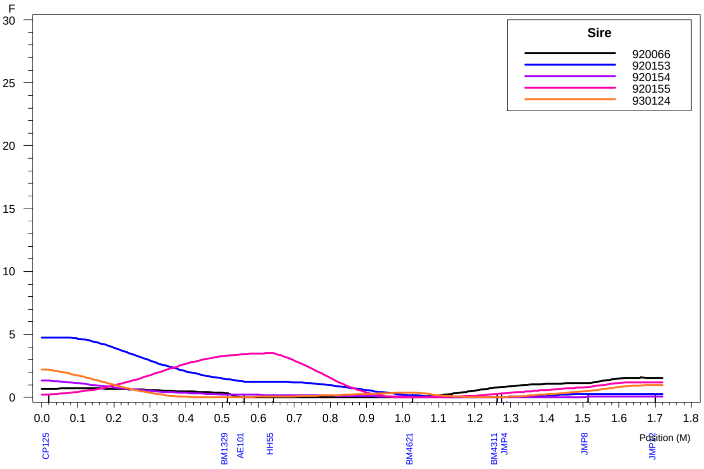

Haley-Knott QTL Analysis: Chromosome 6
ELISA2

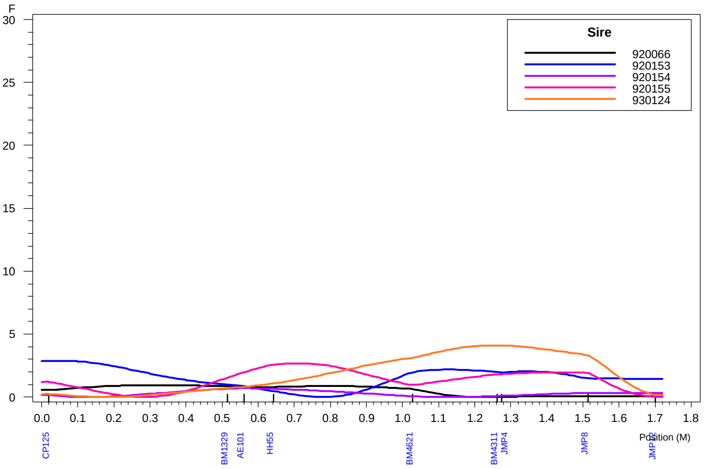

Haley-Knott QTL Analysis: Chromosome 6
ELISA3

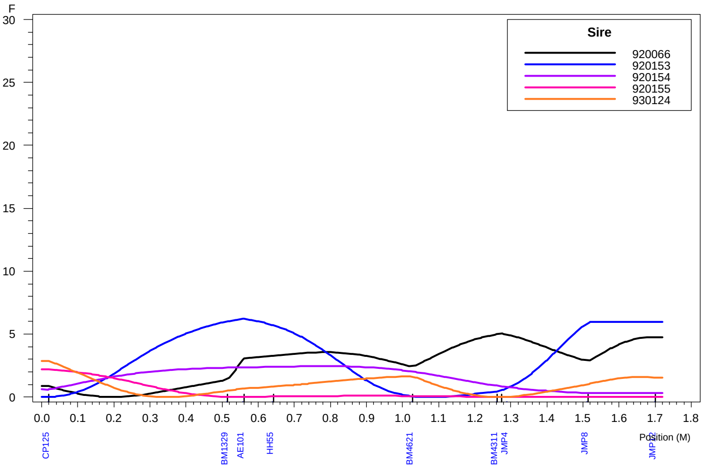

Haley-Knott QTL Analysis: Chromosome 6
ELISA4

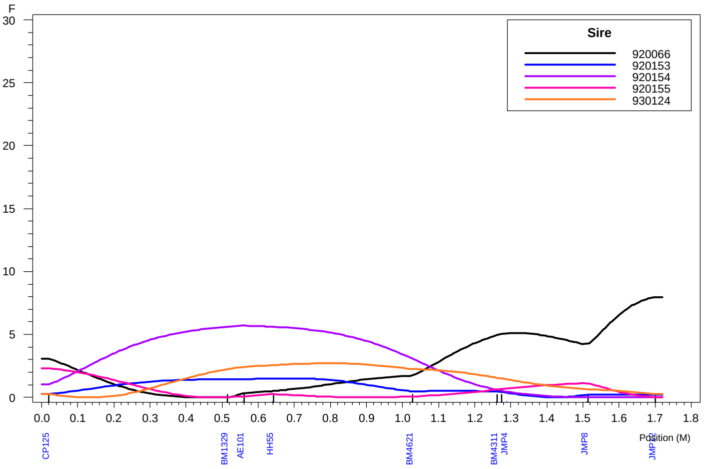

Haley-Knott QTL Analysis: Chromosome 6
ELISA5

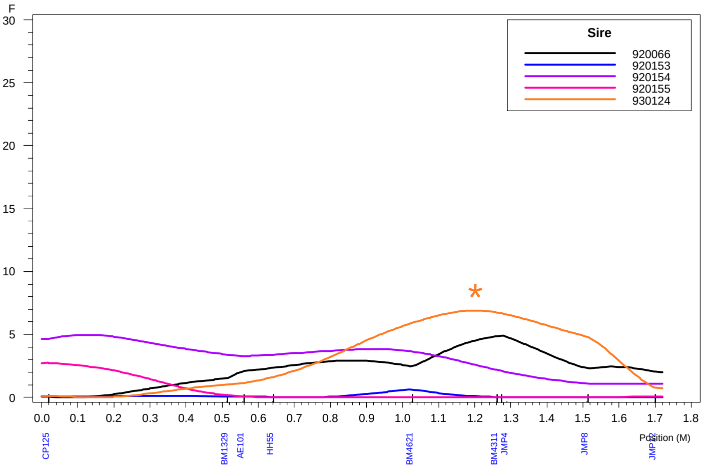

Haley-Knott QTL Analysis: Chromosome 6
LIGE

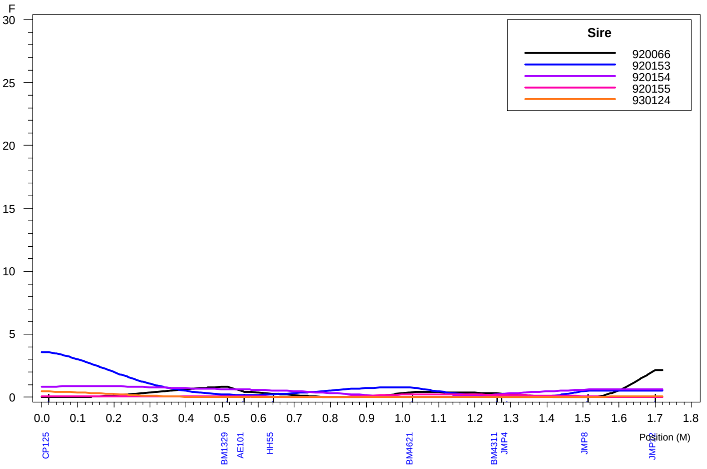

Haley-Knott QTL Analysis: Chromosome 6
WTFEC2

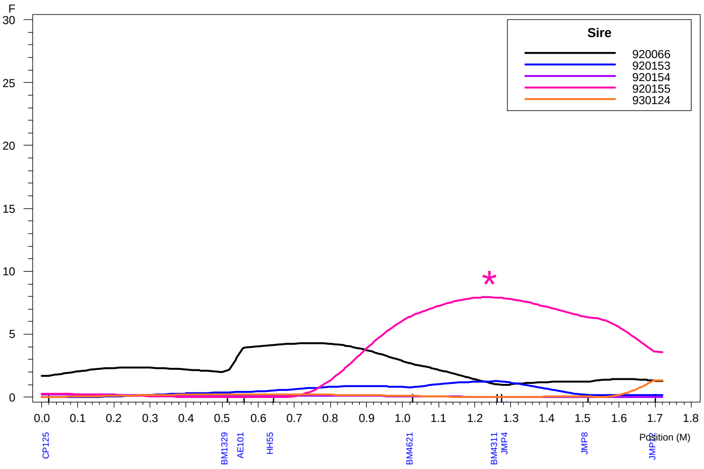

Supplement: Additional File 11 — Chr 6. Haley Knott linkage analysis of sheep chromosome 6. [file 1471-2164-7-178-S11.pdf]
