# Supplementary material for: Discovery of quantitative trait loci for resistance to parasitic nematode infection in sheep: I. Analysis of outcross pedigrees
Source: BMC Genomics. 2006 Jul 18;7:178. doi: 10.1186/1471-2164-7-178 (PMC1574317; doi:10.1186/1471-2164-7-178)

Linkage Analysis in the Parasite Outcross Flock: Chromosome 7

Information Content: Chromosome 7

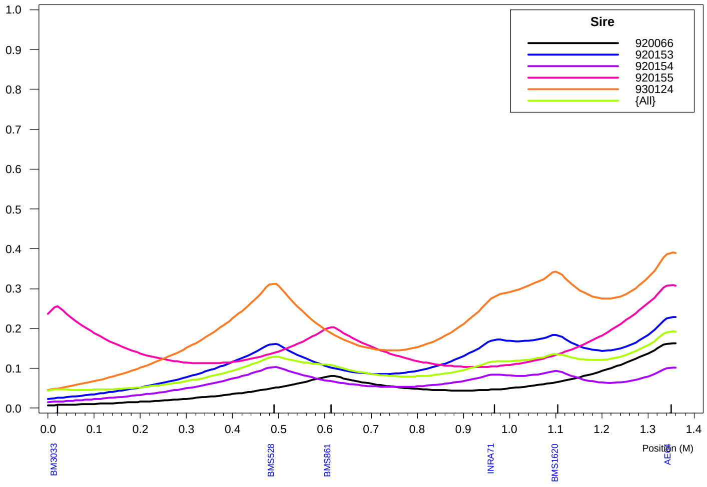

Haley-Knott QTL Analysis: Chromosome 7

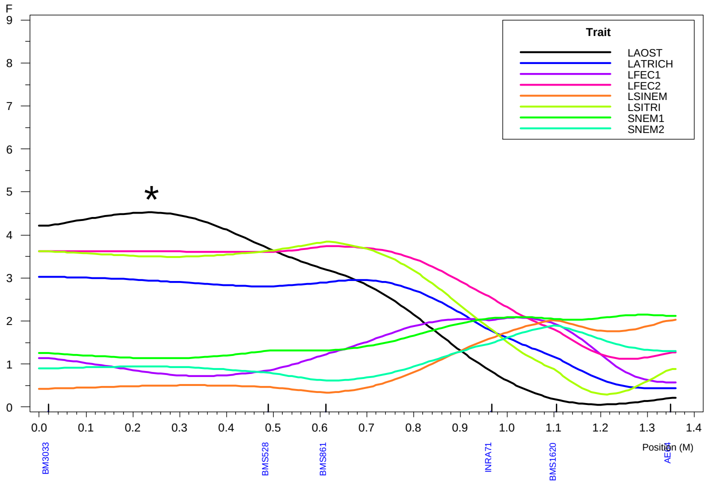

Haley-Knott QTL Analysis: Chromosome 7

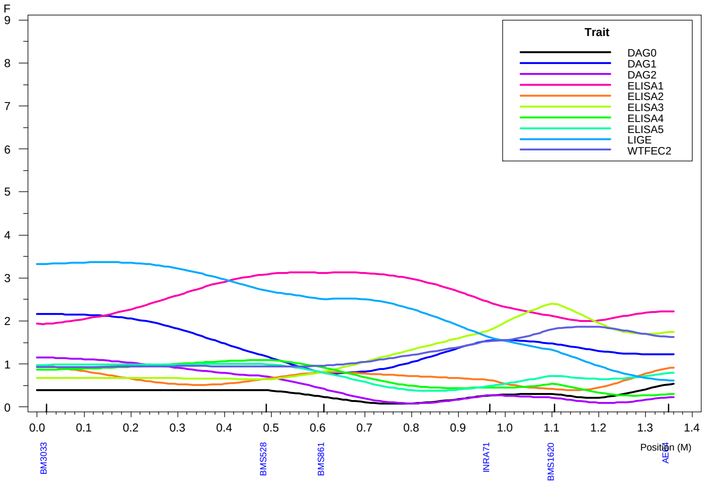

Haley-Knott QTL Analysis: Chromosome 7
LFEC1

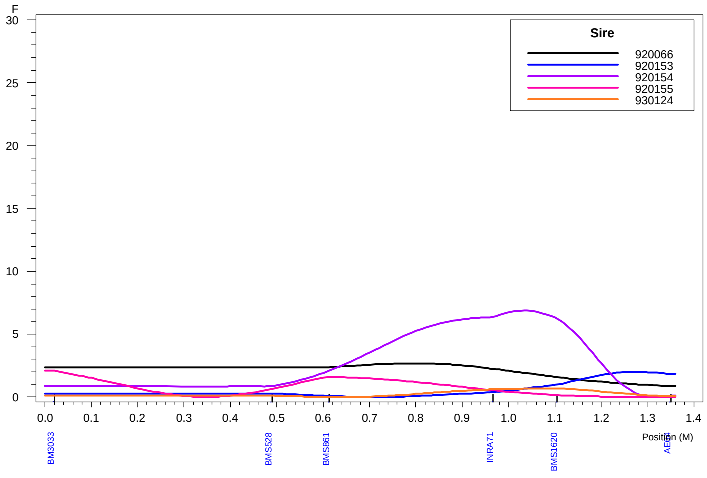

Haley-Knott QTL Analysis: Chromosome 7
LFEC2

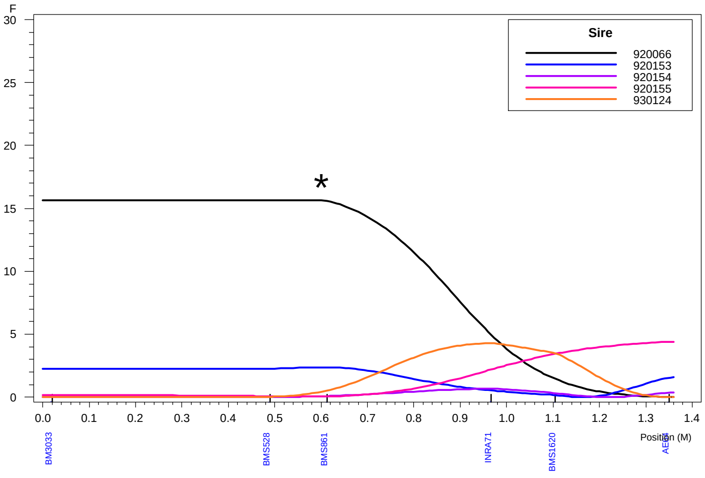

Haley-Knott QTL Analysis: Chromosome 7
SNEM1

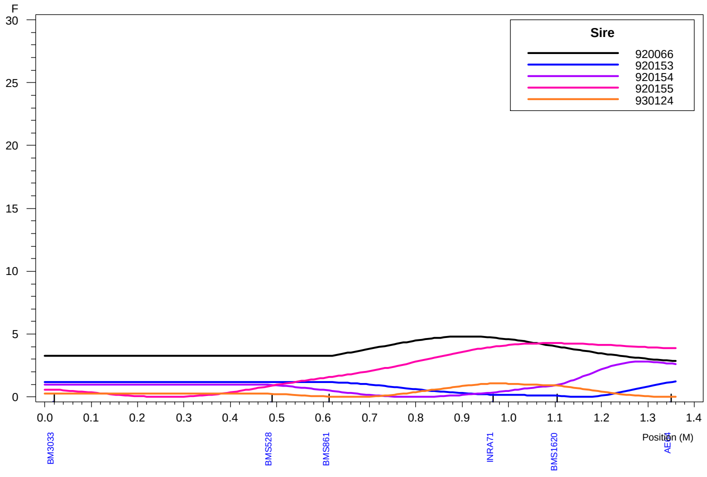

Haley-Knott QTL Analysis: Chromosome 7
SNEM2

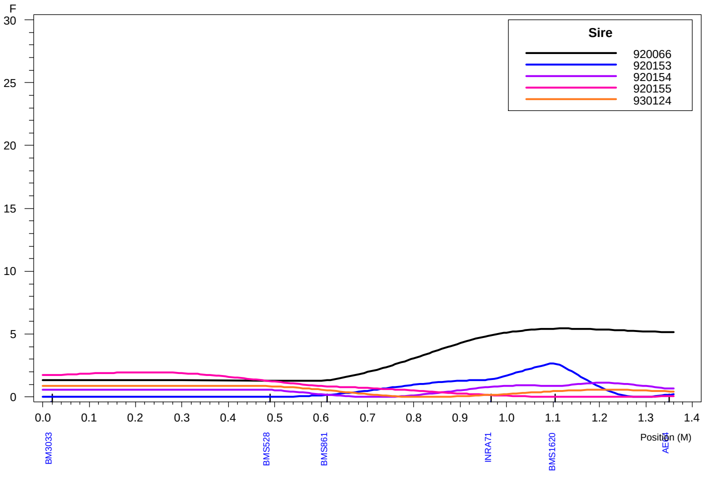

Haley-Knott QTL Analysis: Chromosome 7
LSINEM

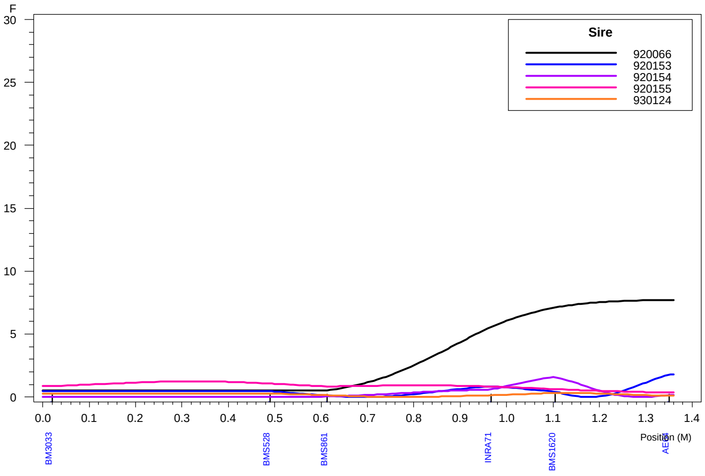

Haley-Knott QTL Analysis: Chromosome 7
LSITRI

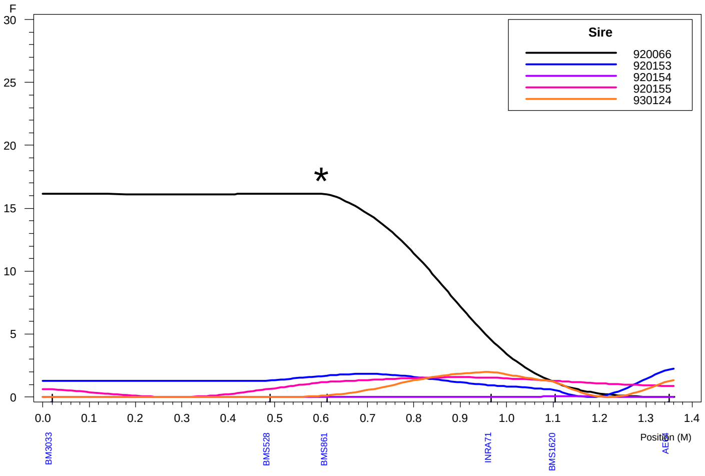

Haley-Knott QTL Analysis: Chromosome 7
LAOST

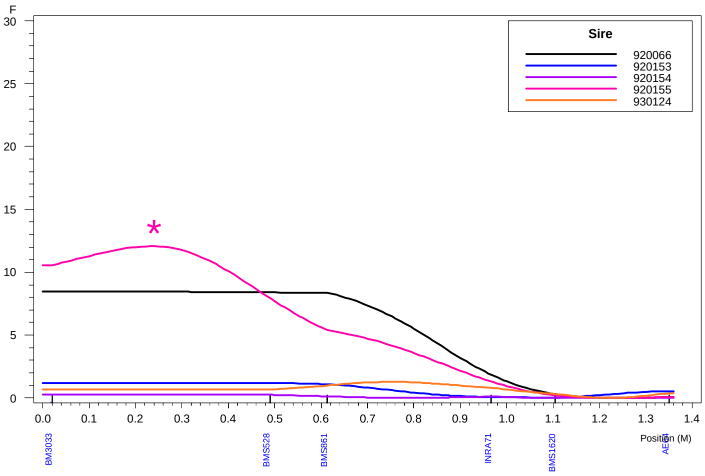

Haley-Knott QTL Analysis: Chromosome 7
LATRICH

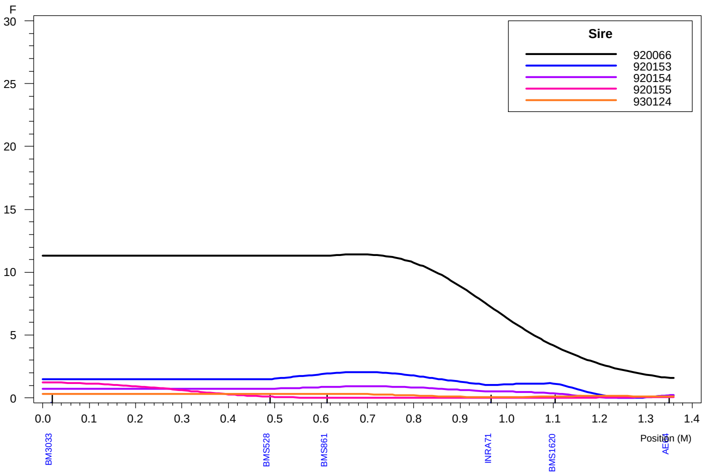

Haley-Knott QTL Analysis: Chromosome 7
DAG0

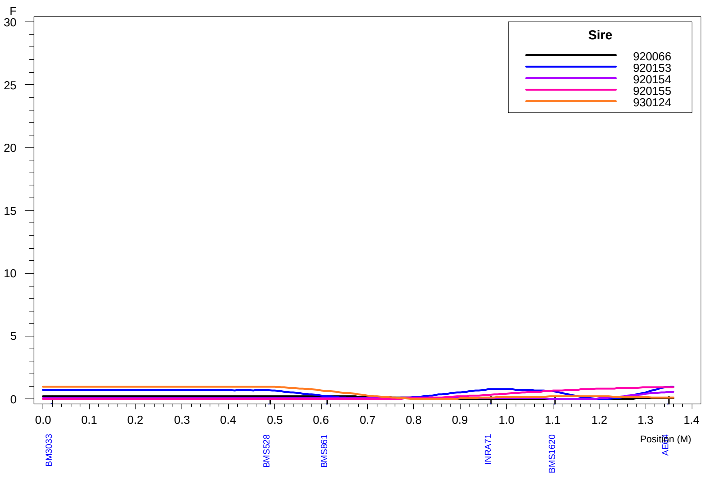

Haley-Knott QTL Analysis: Chromosome 7
DAG1

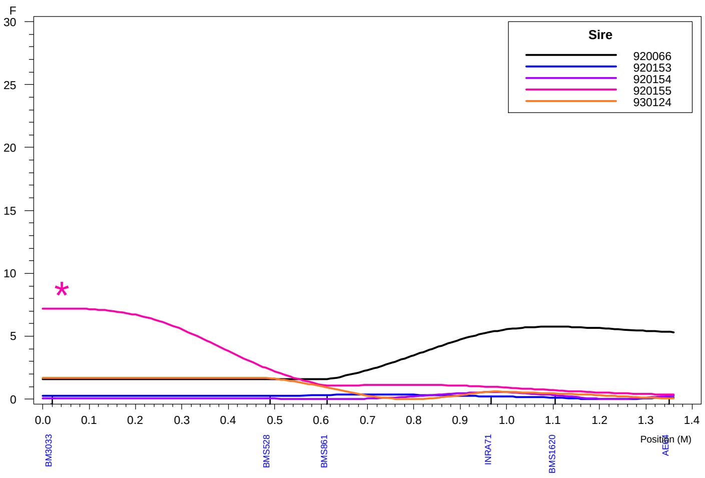

Haley-Knott QTL Analysis: Chromosome 7
DAG2

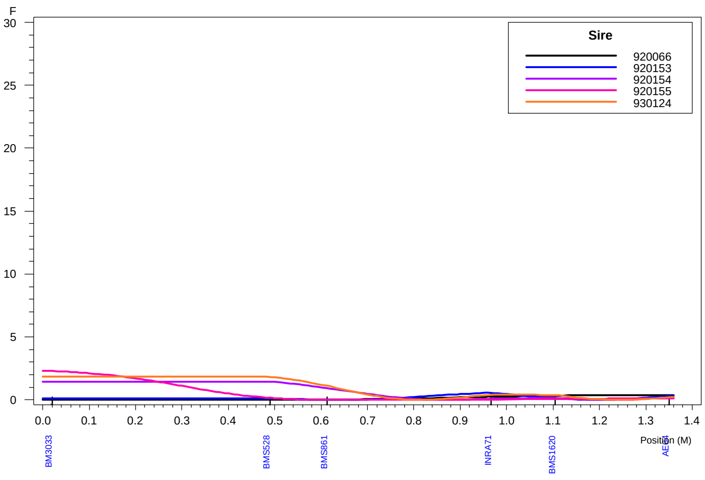

Haley-Knott QTL Analysis: Chromosome 7
ELISA1

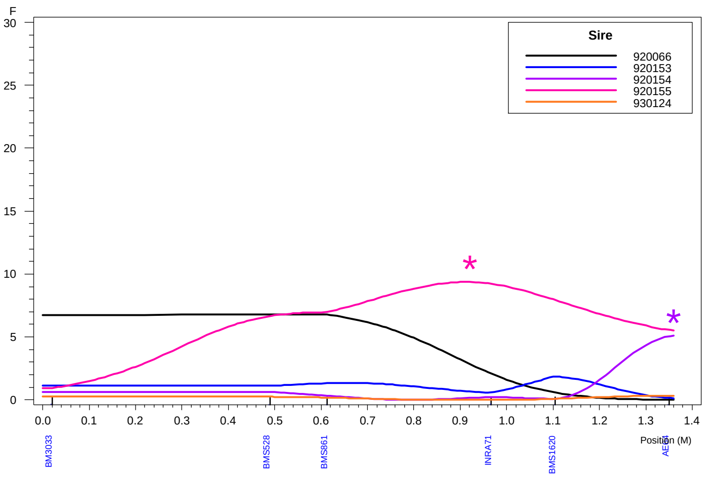

Haley-Knott QTL Analysis: Chromosome 7
ELISA2

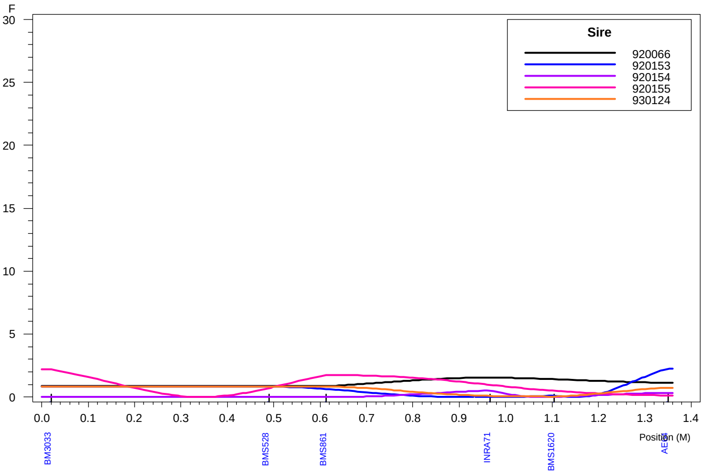

Haley-Knott QTL Analysis: Chromosome 7
ELISA3

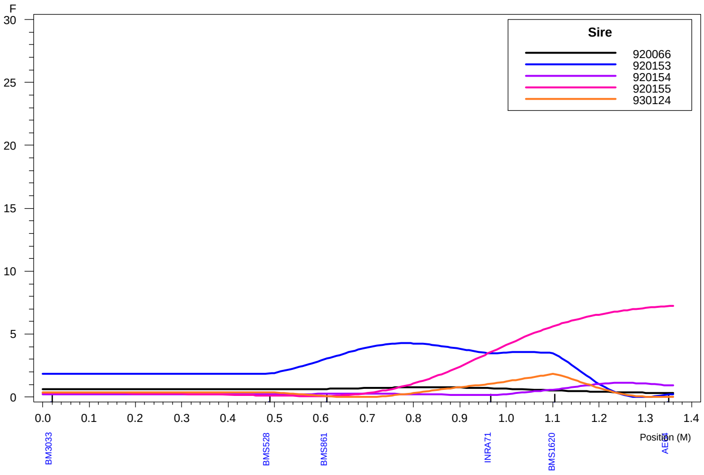

Haley-Knott QTL Analysis: Chromosome 7
ELISA4

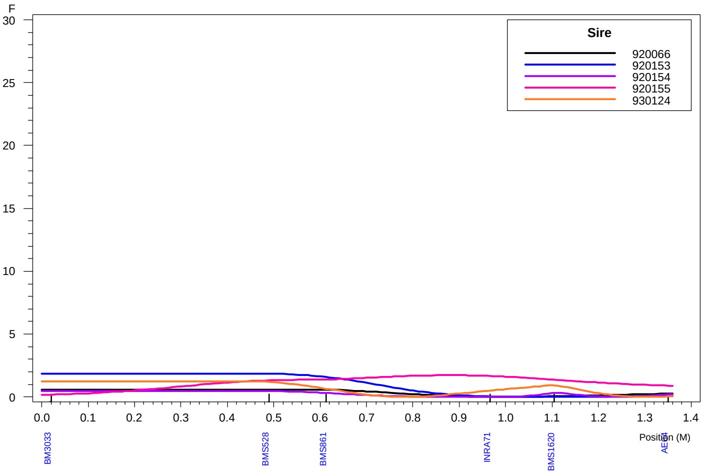

Haley-Knott QTL Analysis: Chromosome 7
ELISA5

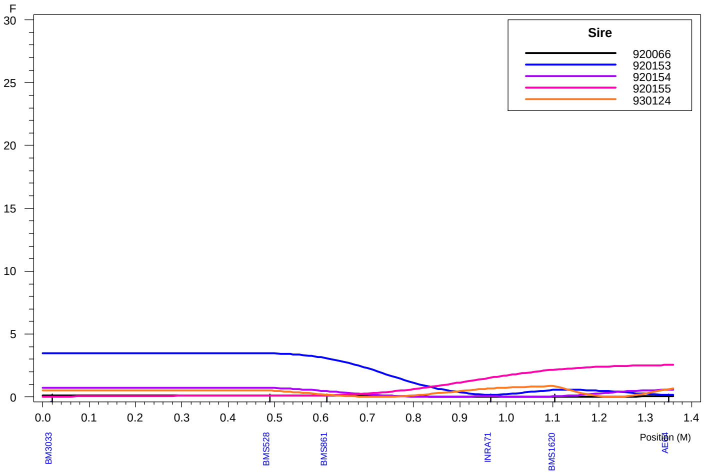

Haley-Knott QTL Analysis: Chromosome 7
LIGE

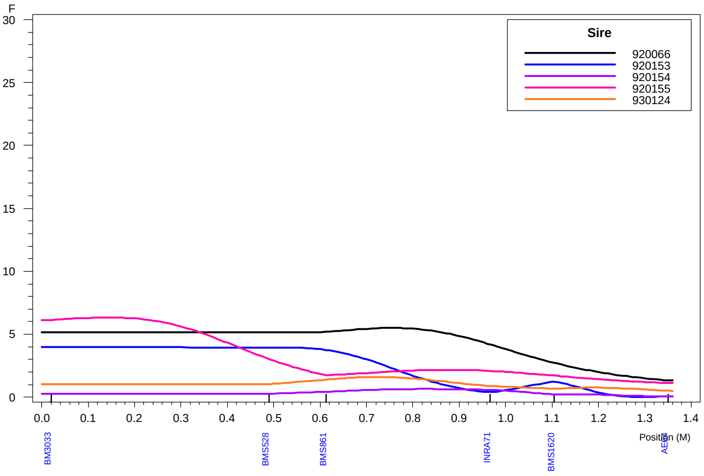

Haley-Knott QTL Analysis: Chromosome 7
WTFEC2

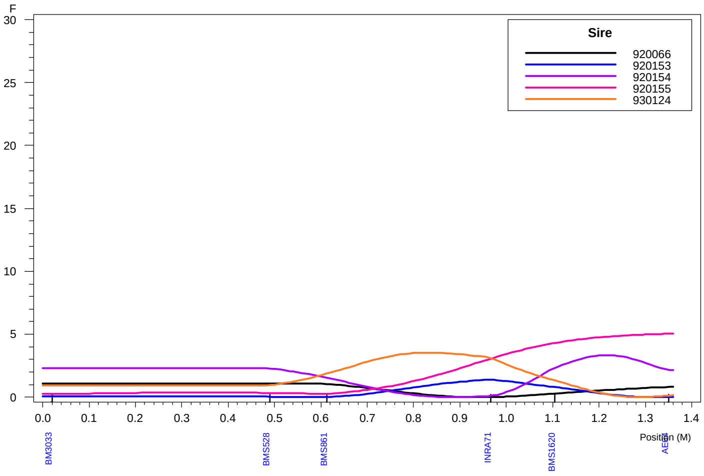

Supplement: Additional File 12 — Chr 7. Haley Knott linkage analysis of sheep chromosome 7. [file 1471-2164-7-178-S12.pdf]
