# Supplementary material for: Discovery of quantitative trait loci for resistance to parasitic nematode infection in sheep: I. Analysis of outcross pedigrees
Source: BMC Genomics. 2006 Jul 18;7:178. doi: 10.1186/1471-2164-7-178 (PMC1574317; doi:10.1186/1471-2164-7-178)

Linkage Analysis in the Parasite Outcross Flock: Chromosome 8

Information Content: Chromosome 8

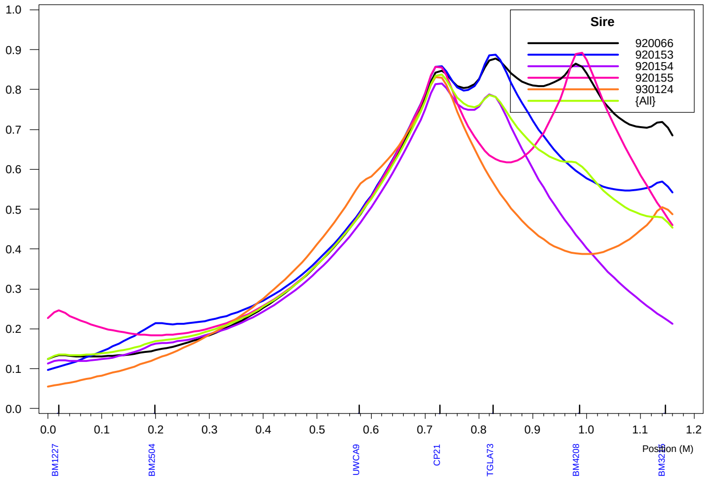

Haley-Knott QTL Analysis: Chromosome 8

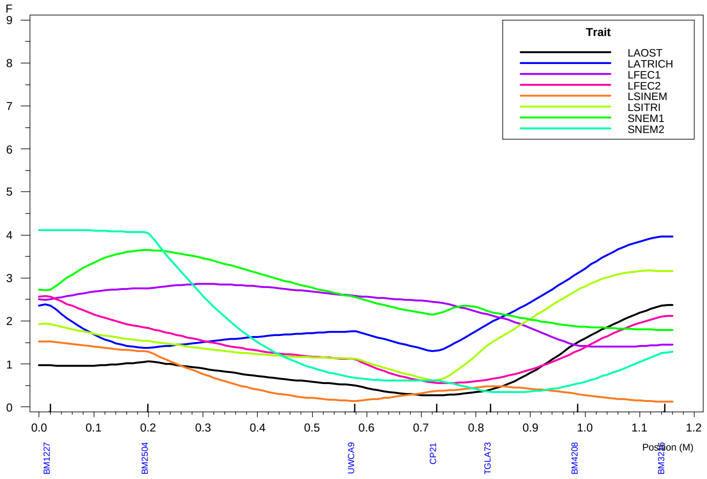

Haley-Knott QTL Analysis: Chromosome 8

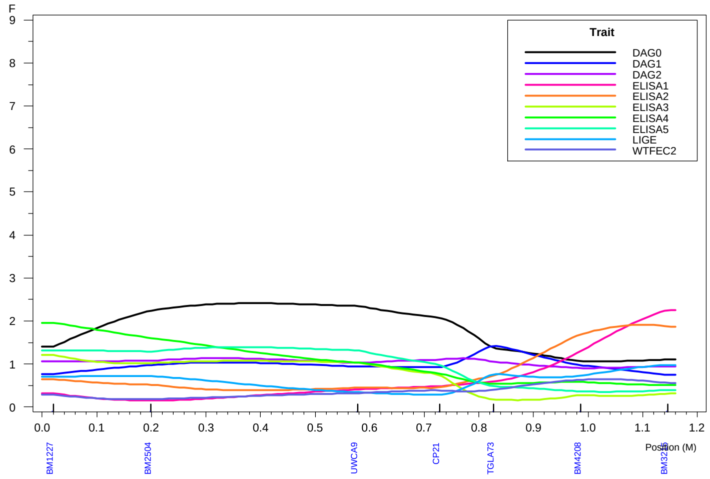

Haley-Knott QTL Analysis: Chromosome 8
LFEC1

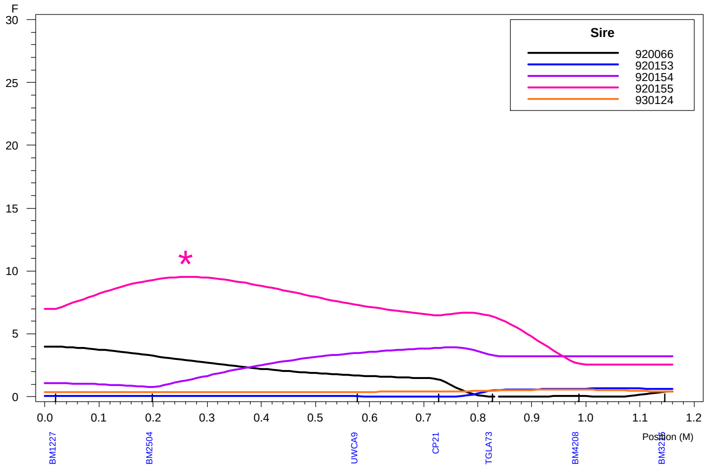

Haley-Knott QTL Analysis: Chromosome 8
LFEC2

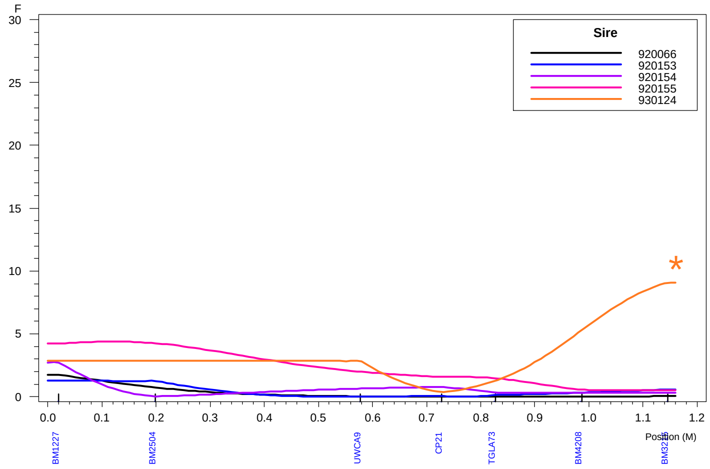

Haley-Knott QTL Analysis: Chromosome 8
SNEM1

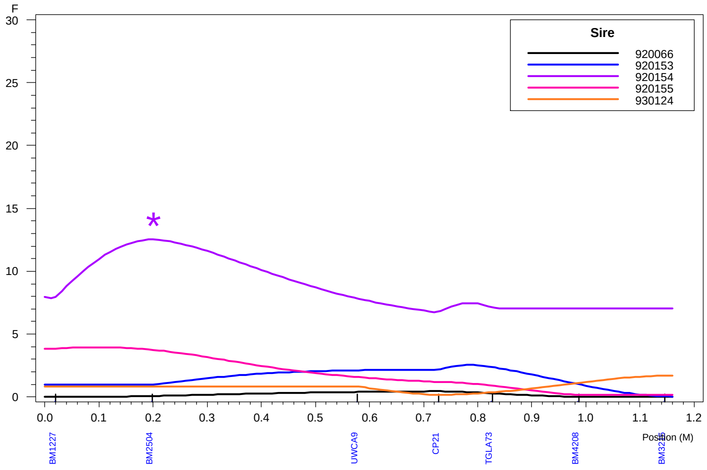

Haley-Knott QTL Analysis: Chromosome 8
SNEM2

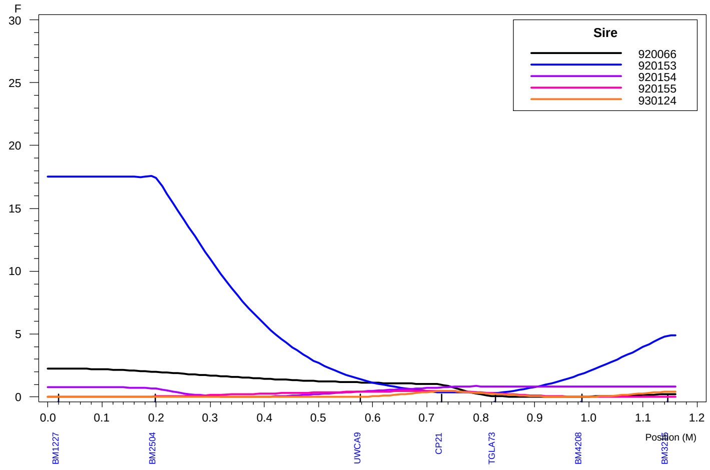

Haley-Knott QTL Analysis: Chromosome 8
LSINEM

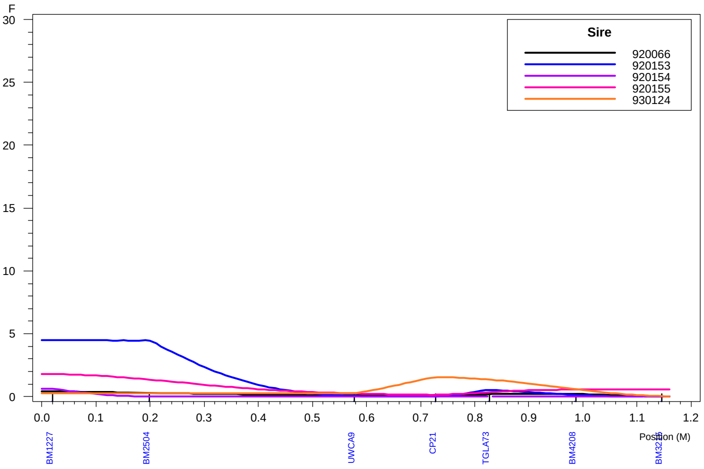

Haley-Knott QTL Analysis: Chromosome 8
LSITRI

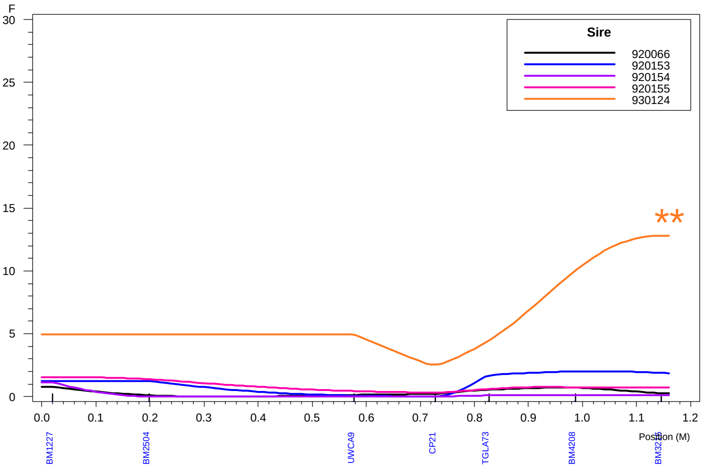

Haley-Knott QTL Analysis: Chromosome 8
LAOST

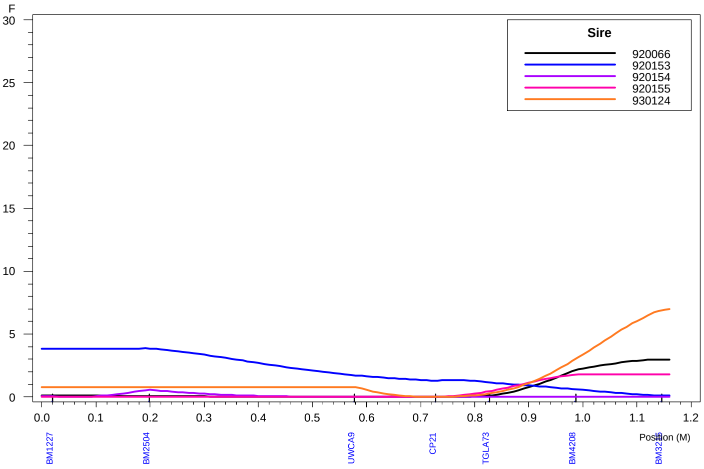

Haley-Knott QTL Analysis: Chromosome 8
LATRICH

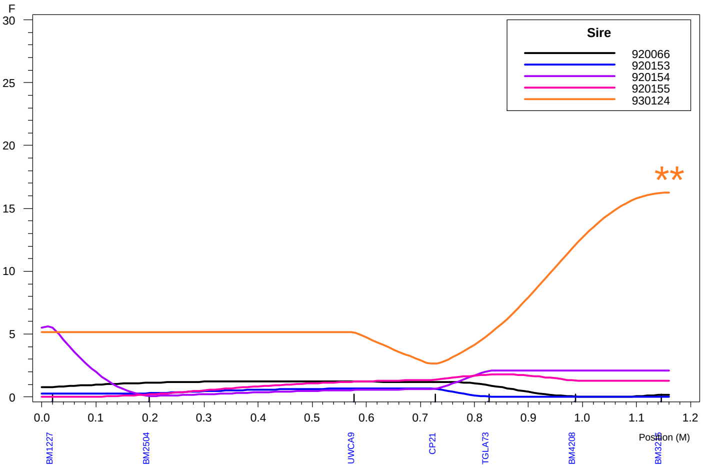

Haley-Knott QTL Analysis: Chromosome 8
DAG0

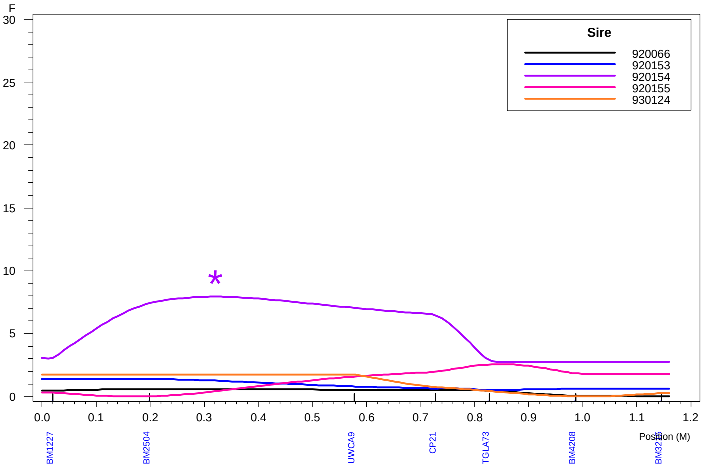

Haley-Knott QTL Analysis: Chromosome 8
DAG1

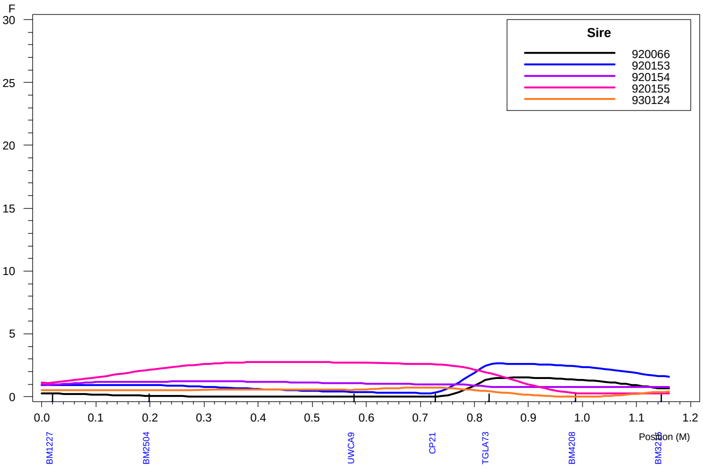

Haley-Knott QTL Analysis: Chromosome 8
DAG2

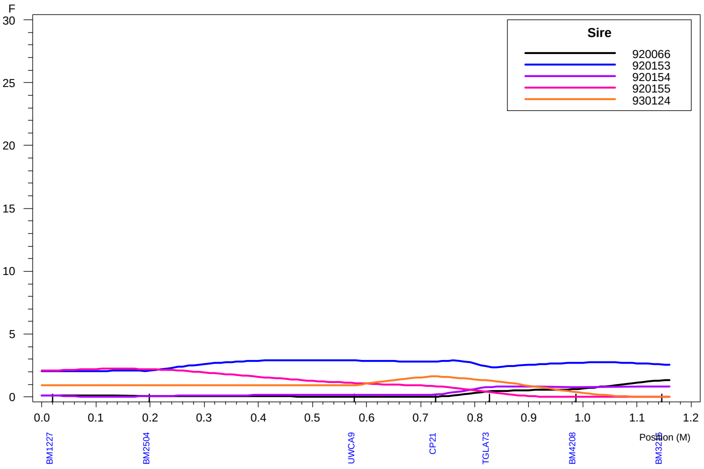

Haley-Knott QTL Analysis: Chromosome 8
ELISA1

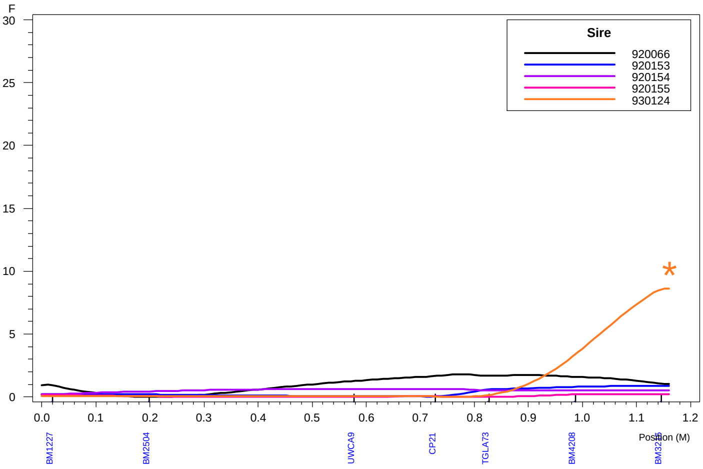

Haley-Knott QTL Analysis: Chromosome 8
ELISA2

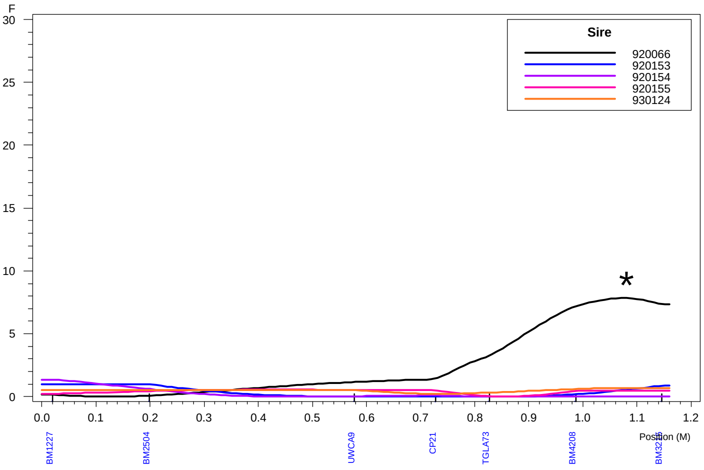

Haley-Knott QTL Analysis: Chromosome 8
ELISA3

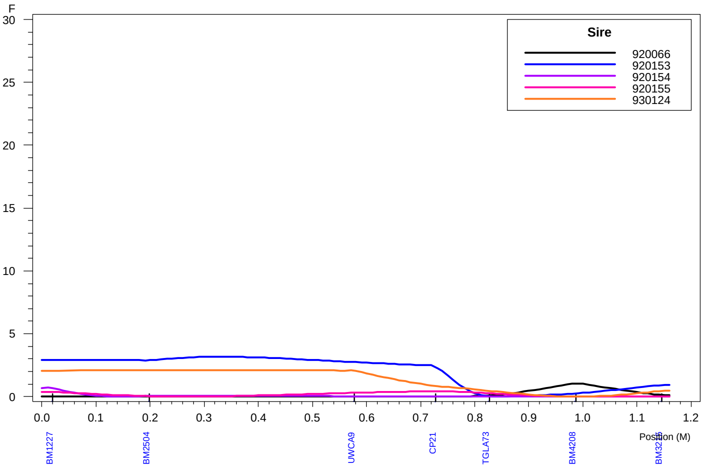

Haley-Knott QTL Analysis: Chromosome 8
ELISA4

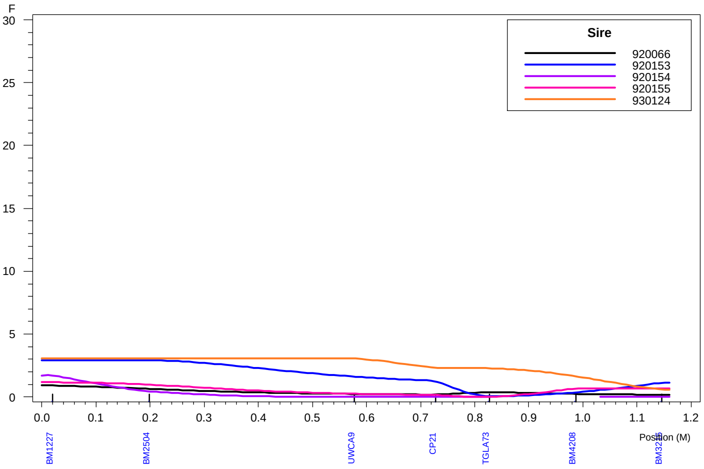

Haley-Knott QTL Analysis: Chromosome 8
ELISA5

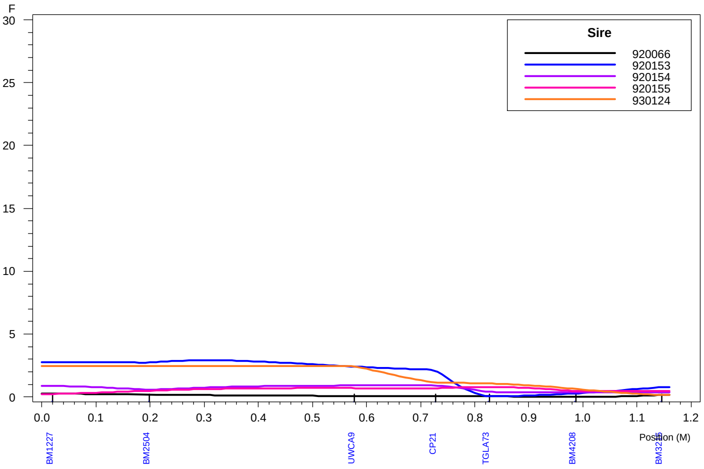

Haley-Knott QTL Analysis: Chromosome 8
LIGE

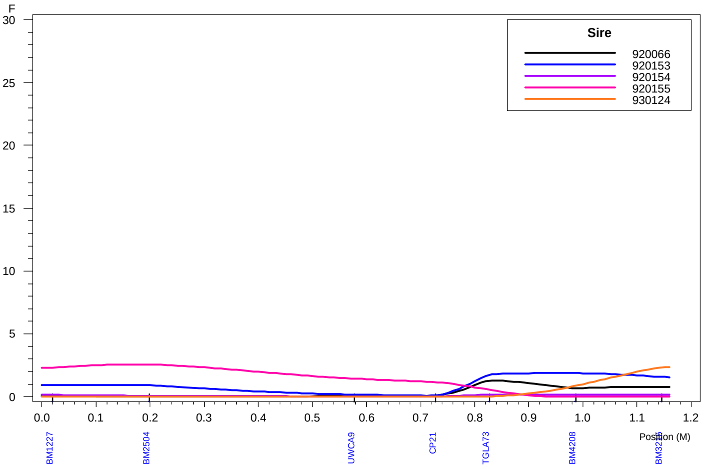

Haley-Knott QTL Analysis: Chromosome 8
WTFEC2

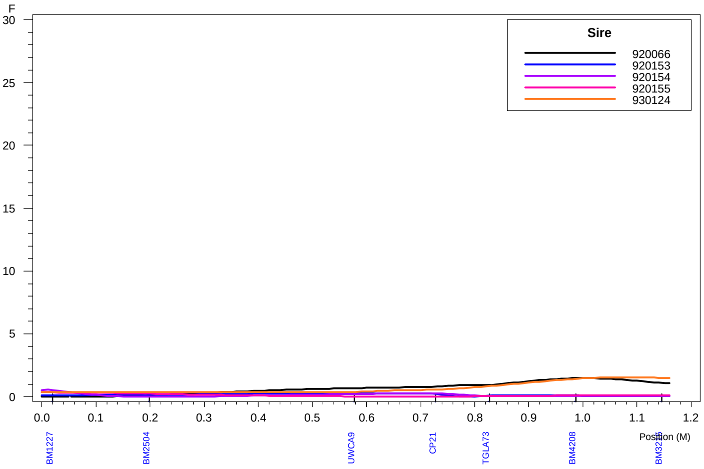

Supplement: Additional File 13 — Chr 8. Haley Knott linkage analysis of sheep chromosome 8. [file 1471-2164-7-178-S13.pdf]
