# Supplementary material for: Discovery of quantitative trait loci for resistance to parasitic nematode infection in sheep: I. Analysis of outcross pedigrees
Source: BMC Genomics. 2006 Jul 18;7:178. doi: 10.1186/1471-2164-7-178 (PMC1574317; doi:10.1186/1471-2164-7-178)

Linkage Analysis in the Parasite Outcross Flock: Chromosome 9

Information Content: Chromosome 9

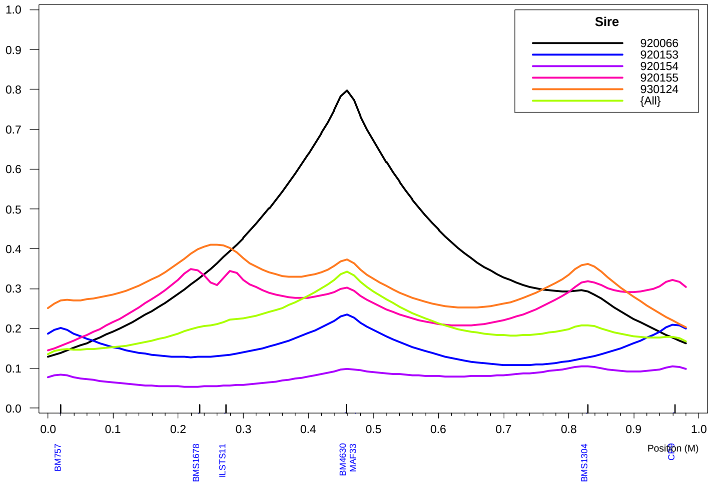

Haley-Knott QTL Analysis: Chromosome 9

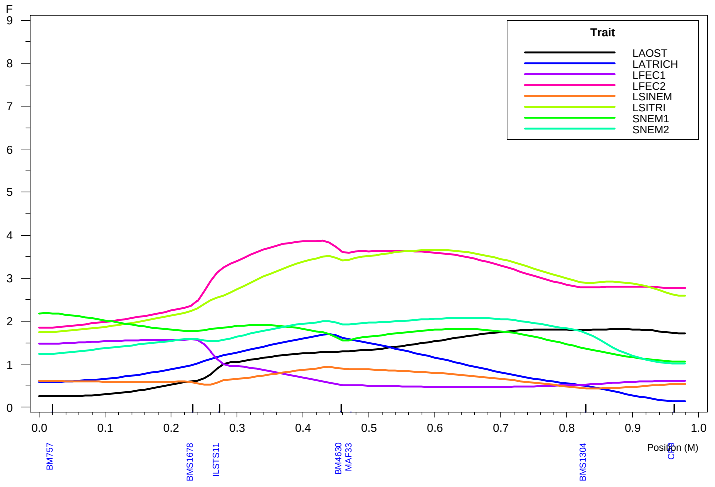

Haley-Knott QTL Analysis: Chromosome 9

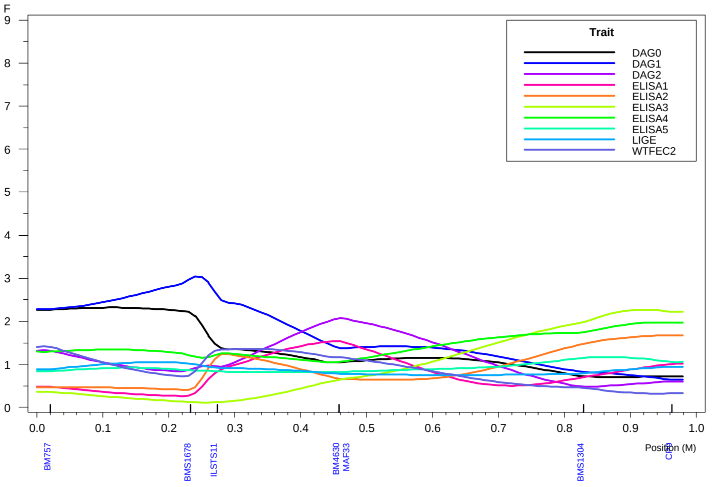

Haley-Knott QTL Analysis: Chromosome 9
LFEC1

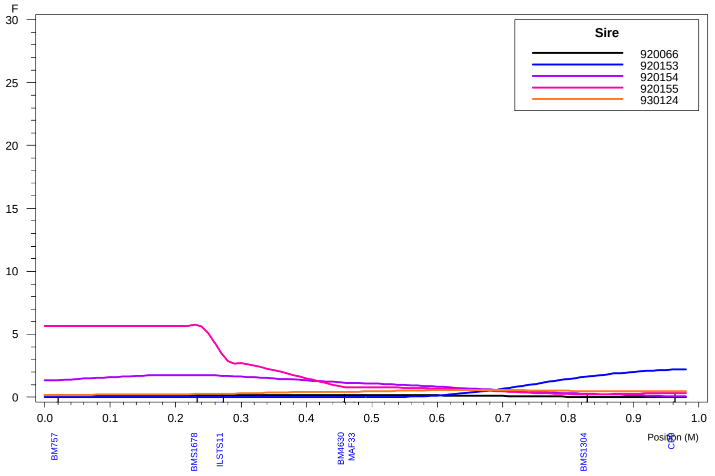

Haley-Knott QTL Analysis: Chromosome 9
LFEC2

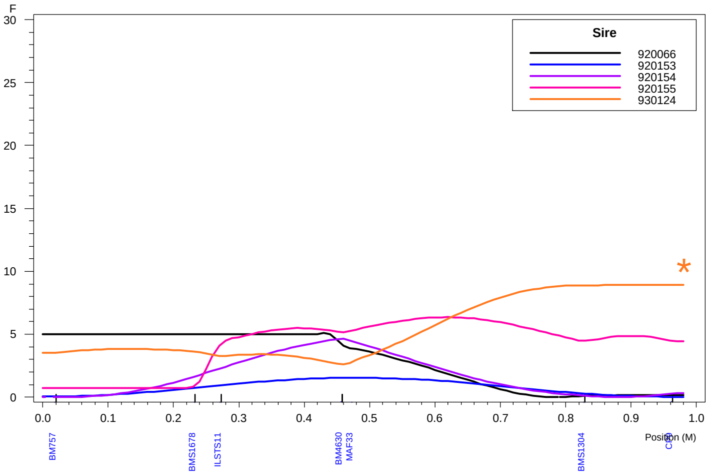

Haley-Knott QTL Analysis: Chromosome 9
SNEM1

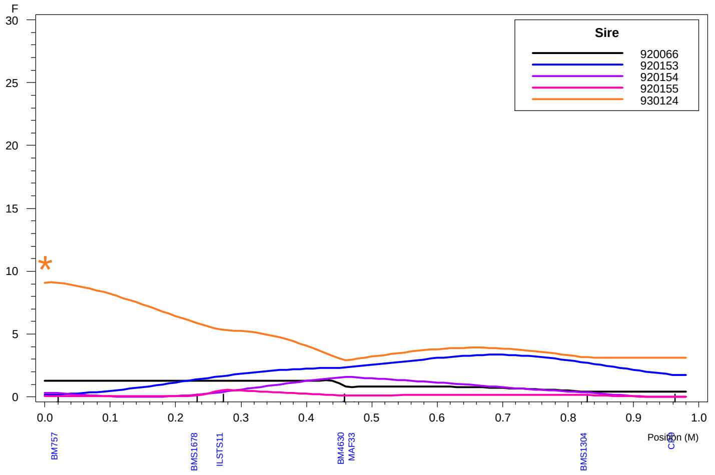

Haley-Knott QTL Analysis: Chromosome 9
SNEM2

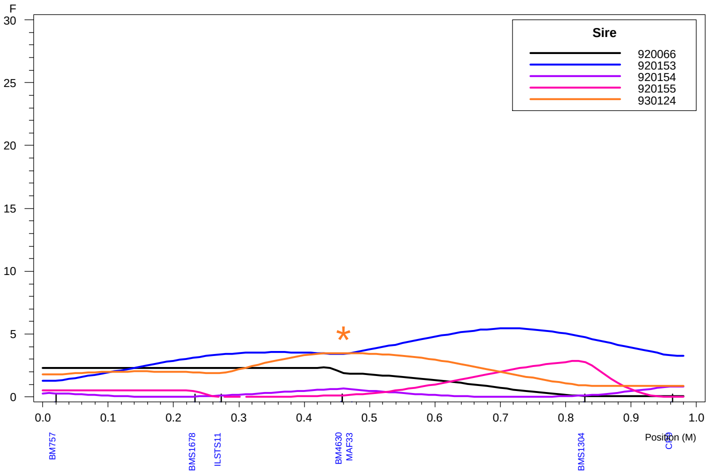

Haley-Knott QTL Analysis: Chromosome 9
LSINEM

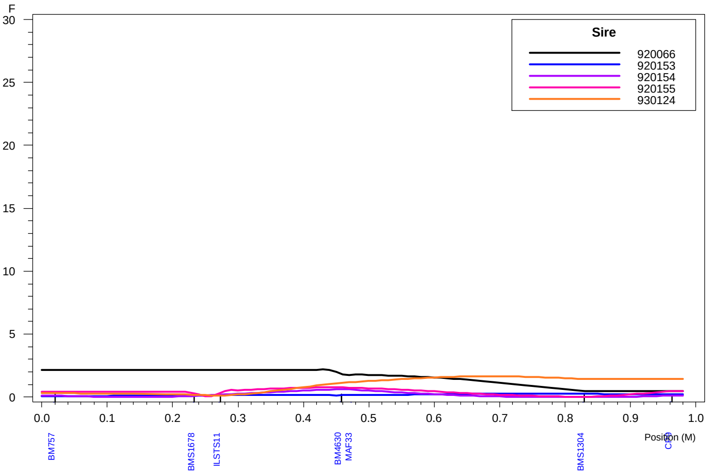

Haley-Knott QTL Analysis: Chromosome 9
LSITRI

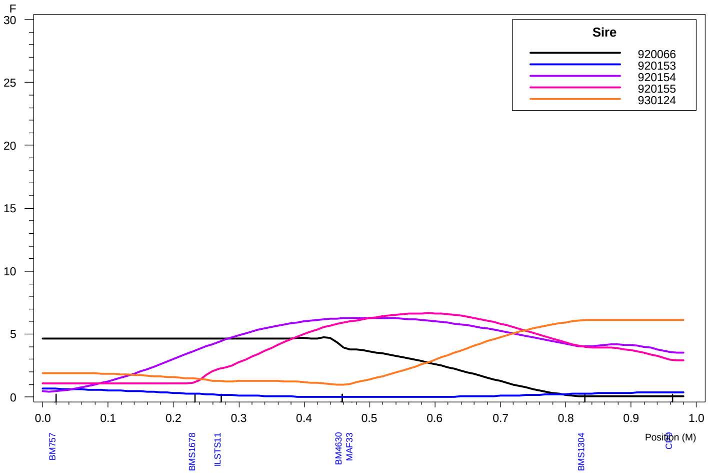

Haley-Knott QTL Analysis: Chromosome 9
LAOST

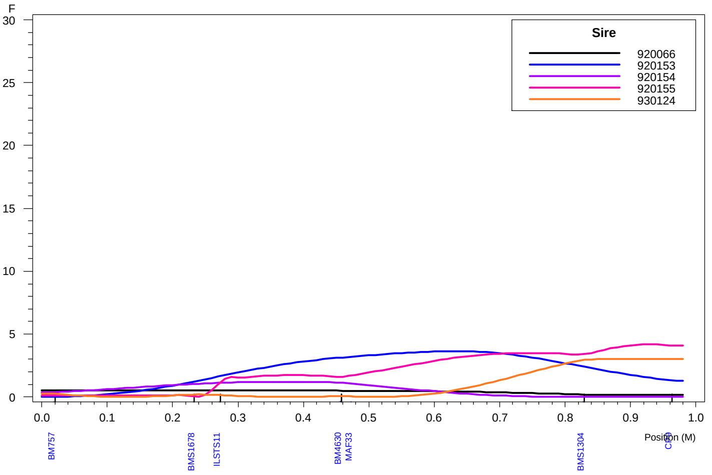

Haley-Knott QTL Analysis: Chromosome 9
LATRICH

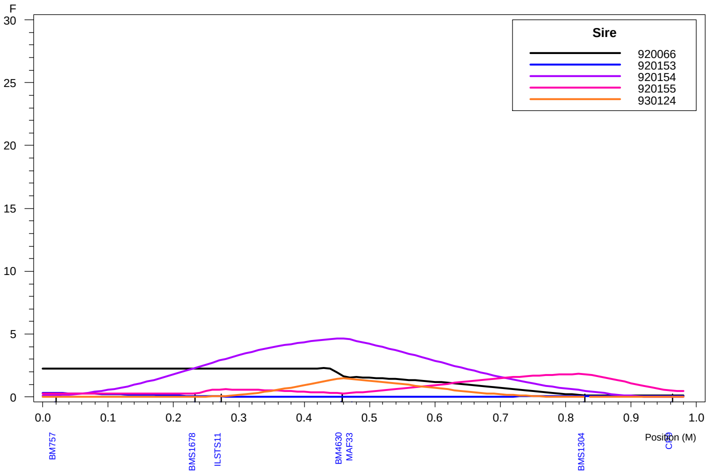

Haley-Knott QTL Analysis: Chromosome 9
DAG0

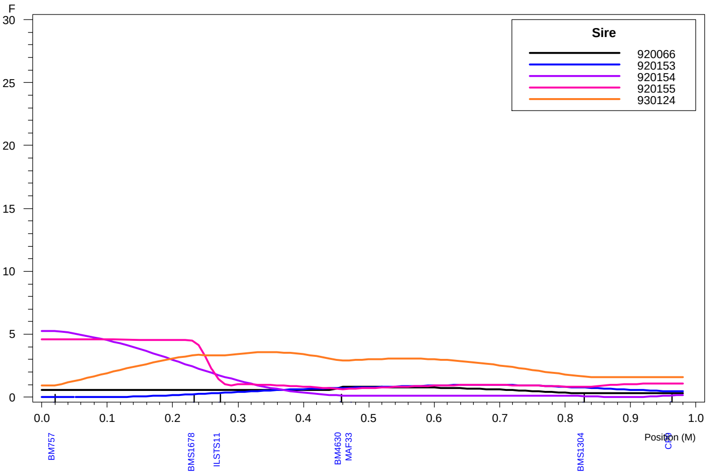

Haley-Knott QTL Analysis: Chromosome 9
DAG1

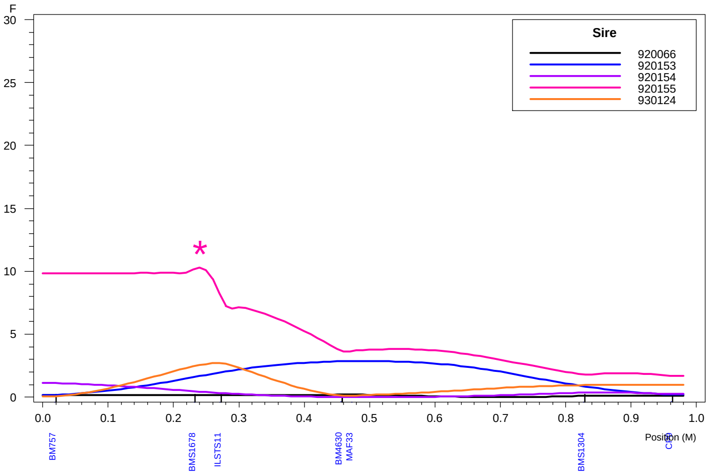

Haley-Knott QTL Analysis: Chromosome 9
DAG2

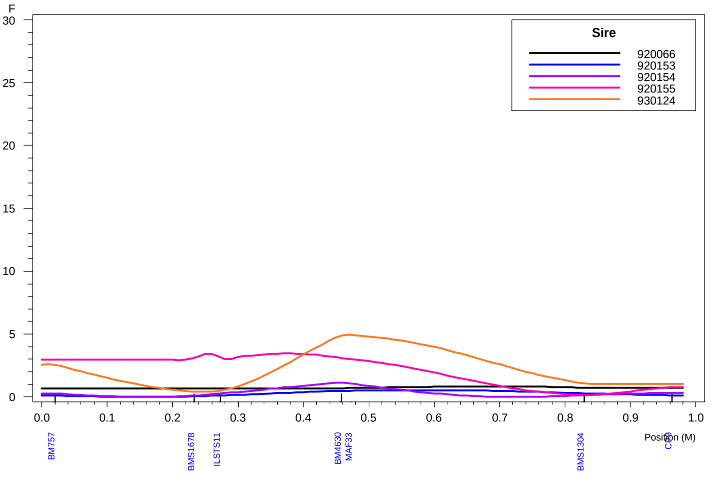

Haley-Knott QTL Analysis: Chromosome 9
ELISA1

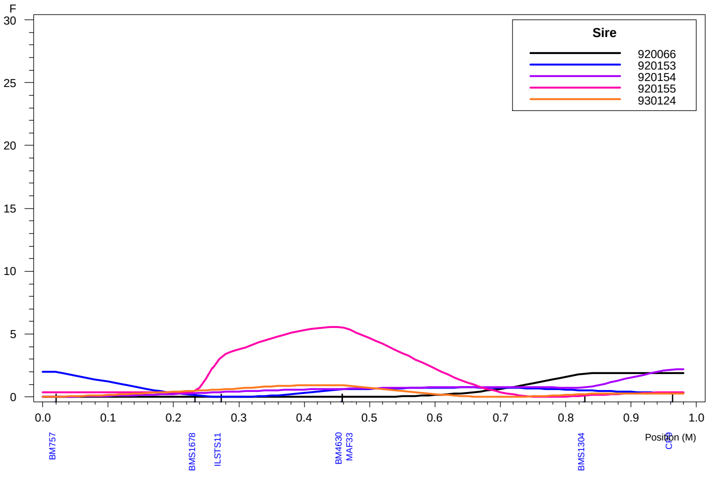

Haley-Knott QTL Analysis: Chromosome 9
ELISA2

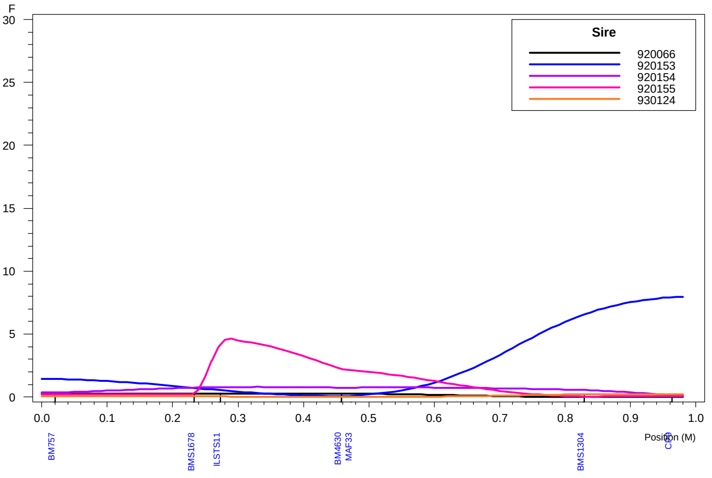

Haley-Knott QTL Analysis: Chromosome 9
ELISA3

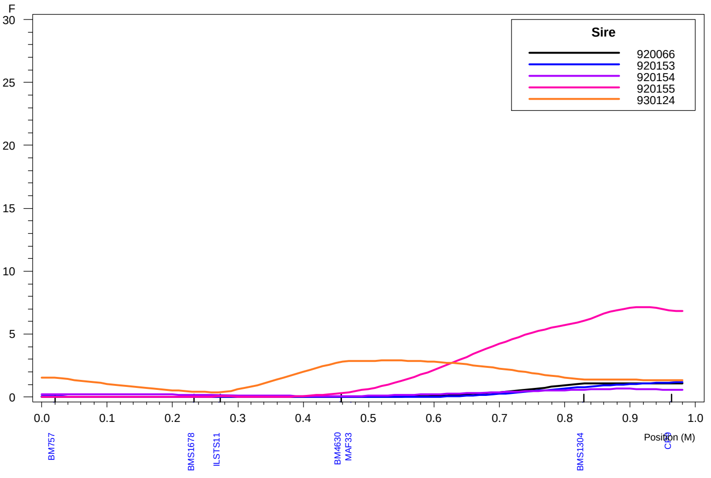

Haley-Knott QTL Analysis: Chromosome 9
ELISA4

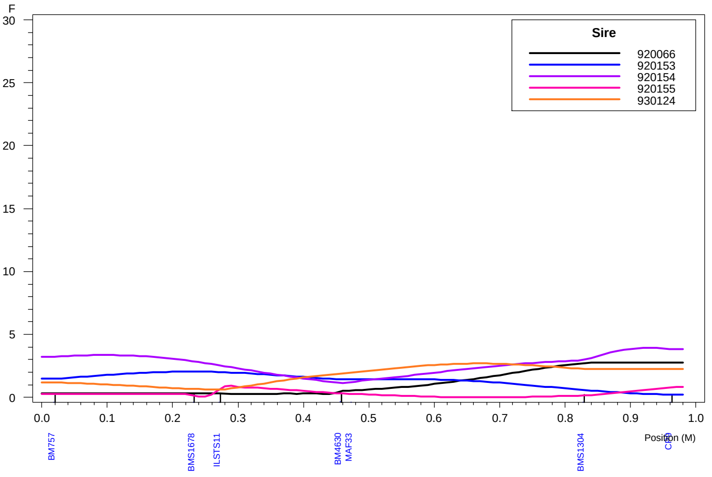

Haley-Knott QTL Analysis: Chromosome 9
ELISA5

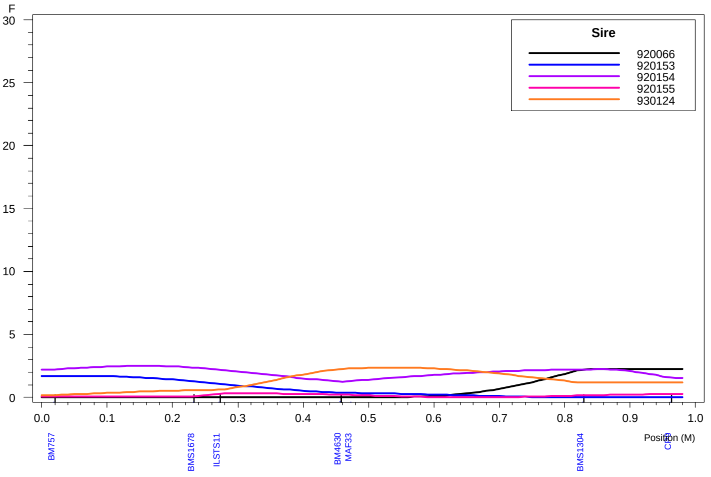

Haley-Knott QTL Analysis: Chromosome 9
LIGE

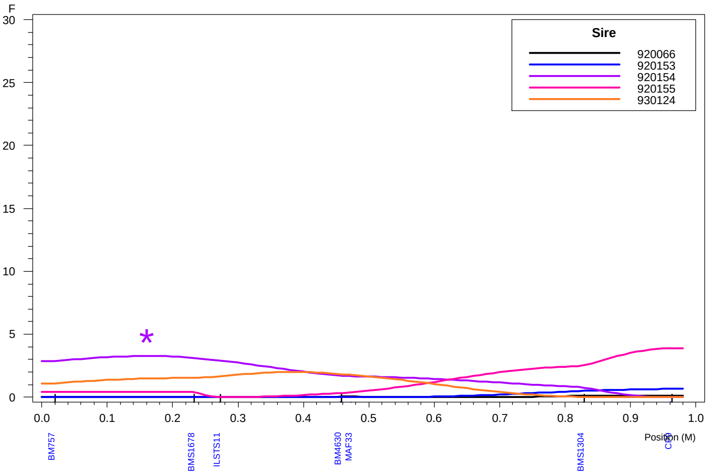

Haley-Knott QTL Analysis: Chromosome 9
WTFEC2

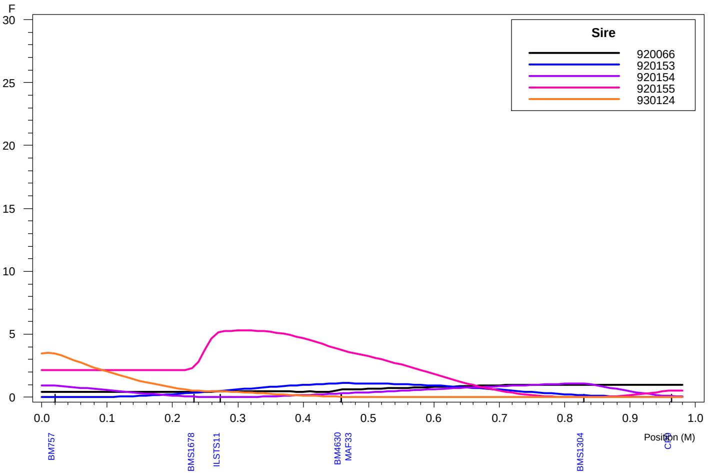

Supplement: Additional File 14 — Chr 9. Haley Knott linkage analysis of sheep chromosome 9. [file 1471-2164-7-178-S14.pdf]
