# Supplementary material for: Discovery of quantitative trait loci for resistance to parasitic nematode infection in sheep: I. Analysis of outcross pedigrees
Source: BMC Genomics. 2006 Jul 18;7:178. doi: 10.1186/1471-2164-7-178 (PMC1574317; doi:10.1186/1471-2164-7-178)

Linkage Analysis in the Parasite Outcross Flock: Chromosome 10

Information Content: Chromosome 10

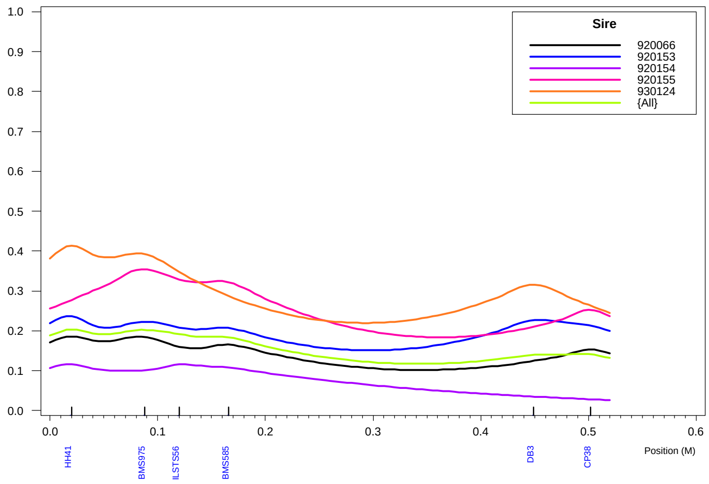

Haley-Knott QTL Analysis: Chromosome 10

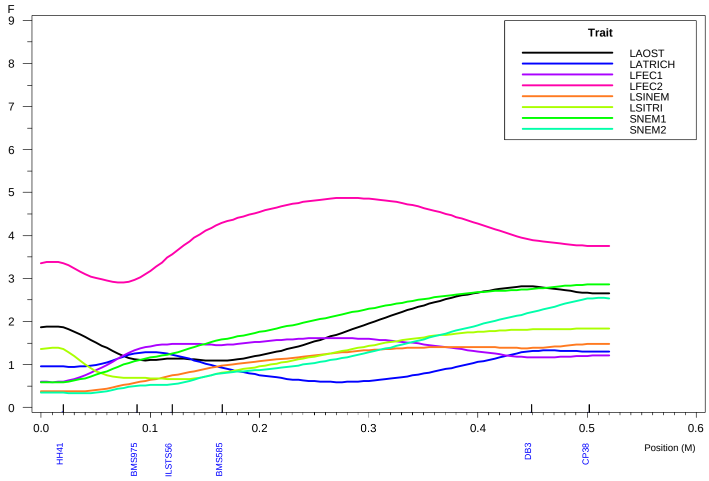

Haley-Knott QTL Analysis: Chromosome 10

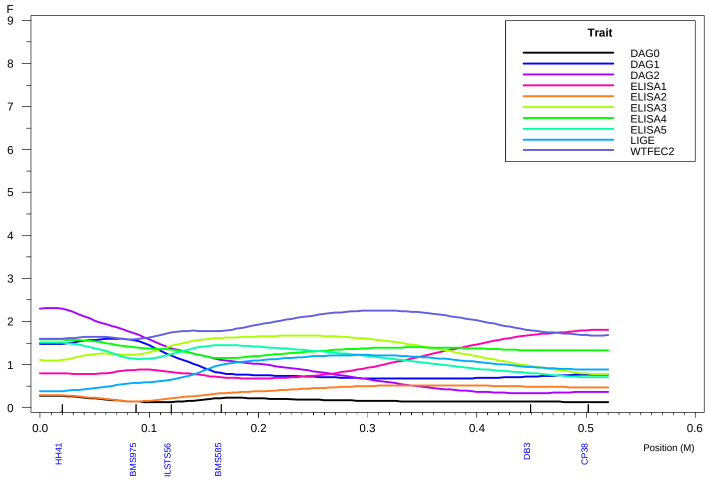

Haley-Knott QTL Analysis: Chromosome 10
LFEC1

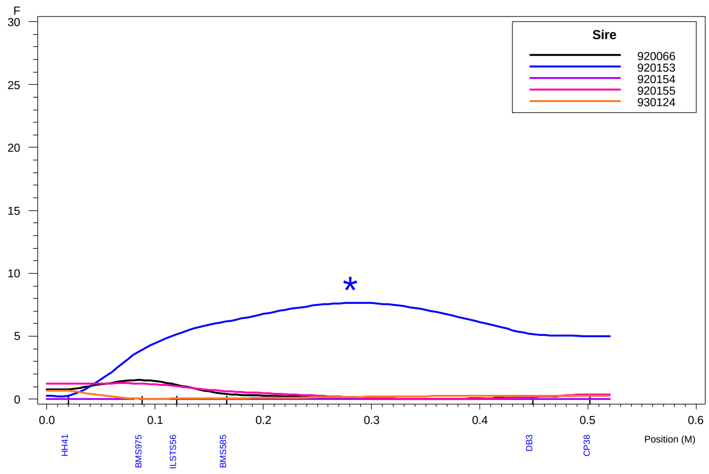

Haley-Knott QTL Analysis: Chromosome 10
LFEC2

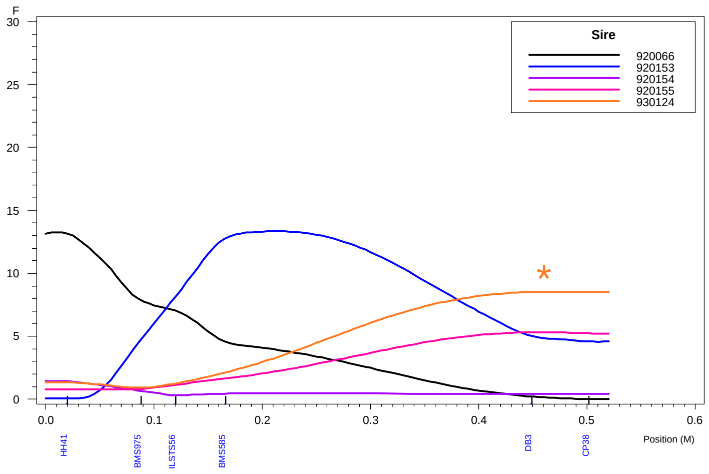

Haley-Knott QTL Analysis: Chromosome 10
SNEM1

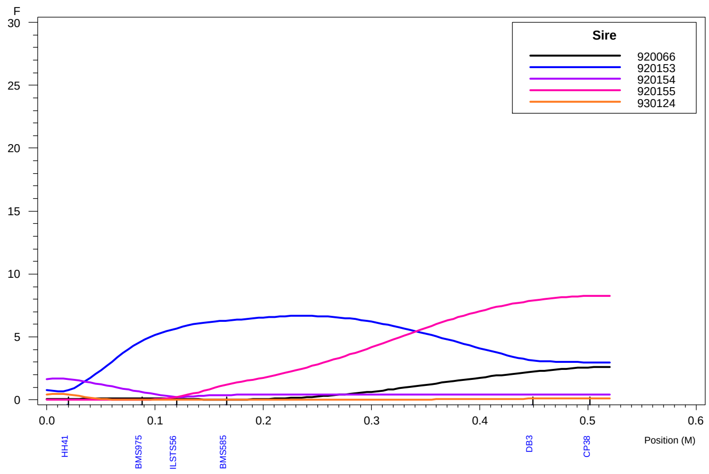

Haley-Knott QTL Analysis: Chromosome 10
SNEM2

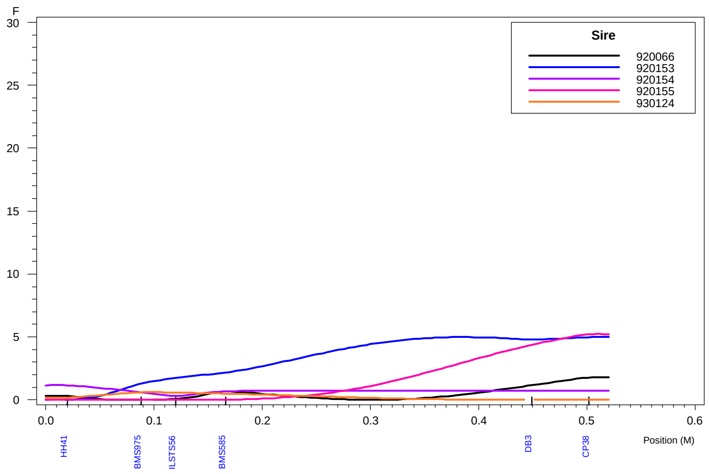

Haley-Knott QTL Analysis: Chromosome 10
LSINEM

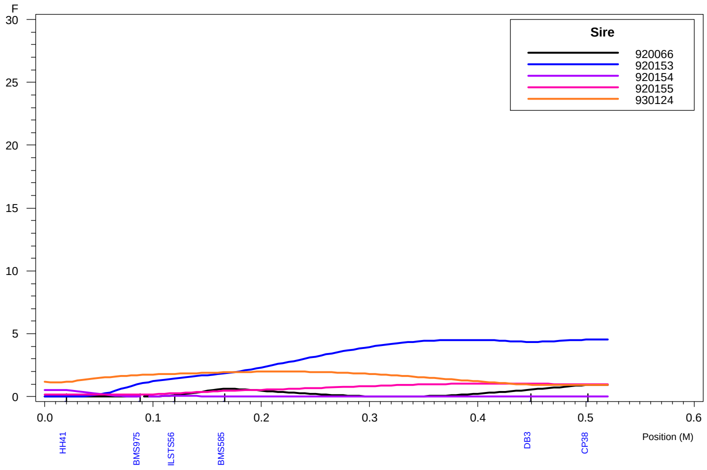

Haley-Knott QTL Analysis: Chromosome 10
LSITRI

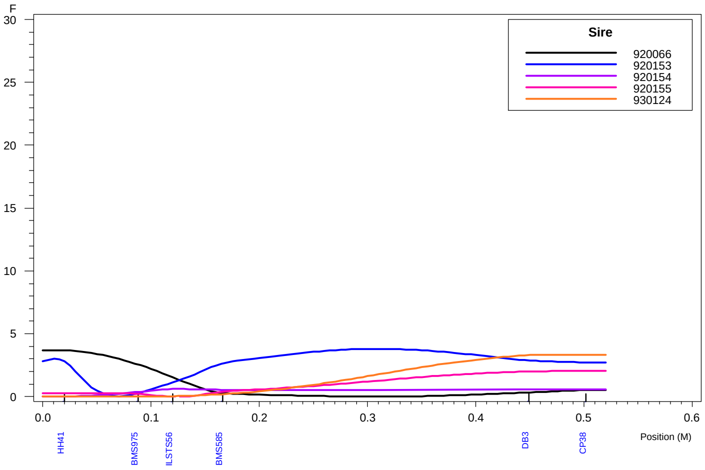

Haley-Knott QTL Analysis: Chromosome 10
LAOST

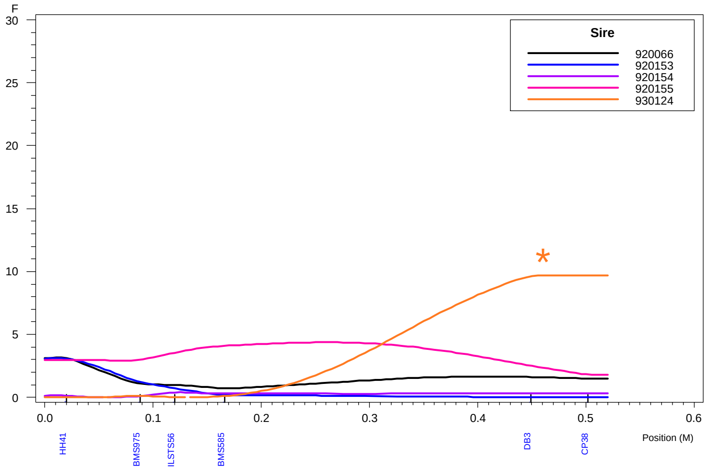

Haley-Knott QTL Analysis: Chromosome 10
LATRICH

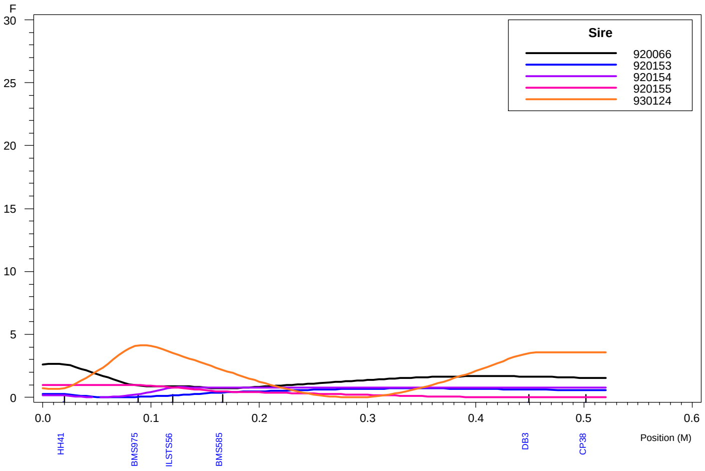

Haley-Knott QTL Analysis: Chromosome 10
DAG0

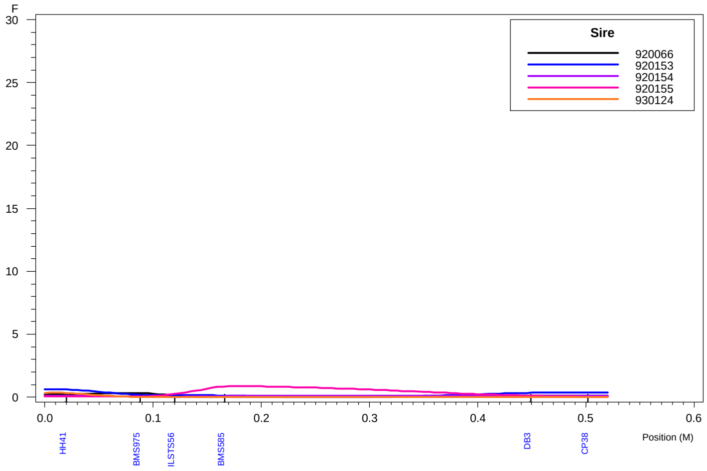

Haley-Knott QTL Analysis: Chromosome 10
DAG1

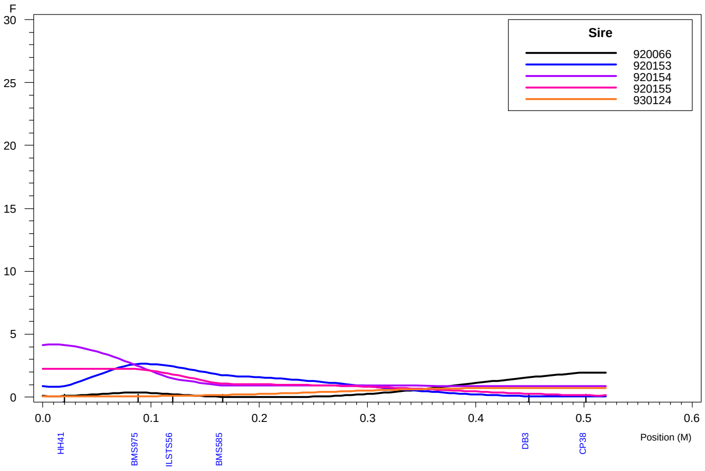

Haley-Knott QTL Analysis: Chromosome 10
DAG2

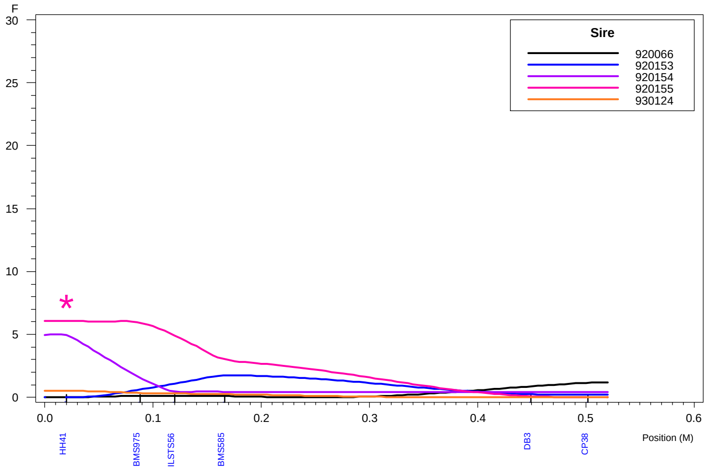

Haley-Knott QTL Analysis: Chromosome 10
ELISA1

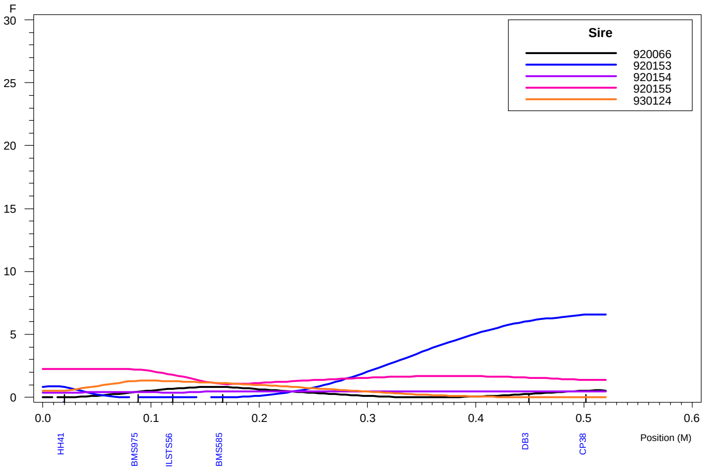

Haley-Knott QTL Analysis: Chromosome 10
ELISA2

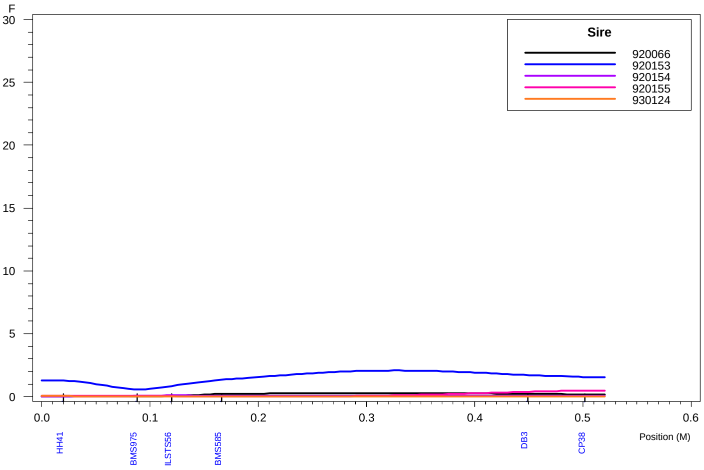

Haley-Knott QTL Analysis: Chromosome 10
ELISA3

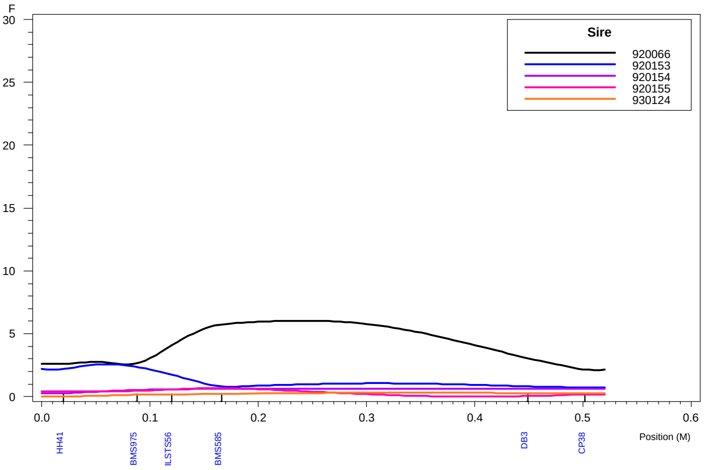

Haley-Knott QTL Analysis: Chromosome 10
ELISA4

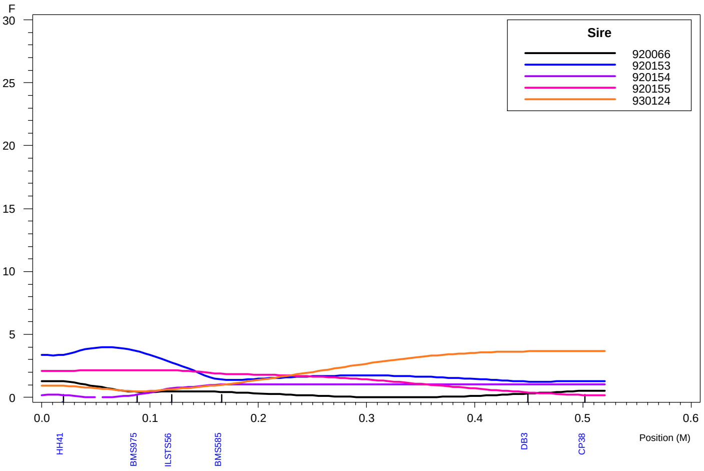

Haley-Knott QTL Analysis: Chromosome 10
ELISA5

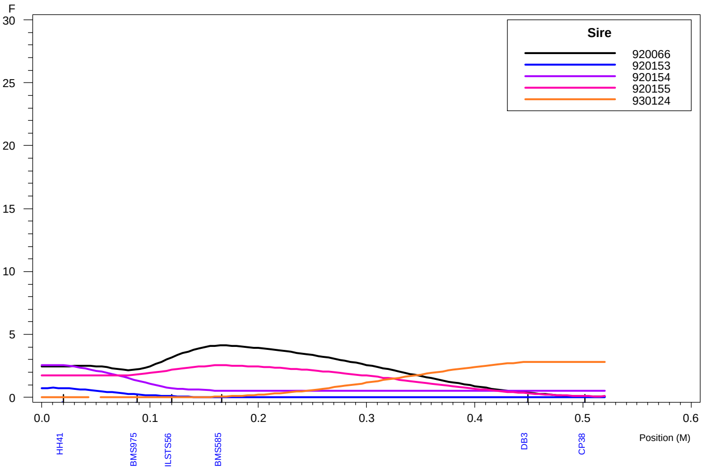

Haley-Knott QTL Analysis: Chromosome 10
LIGE

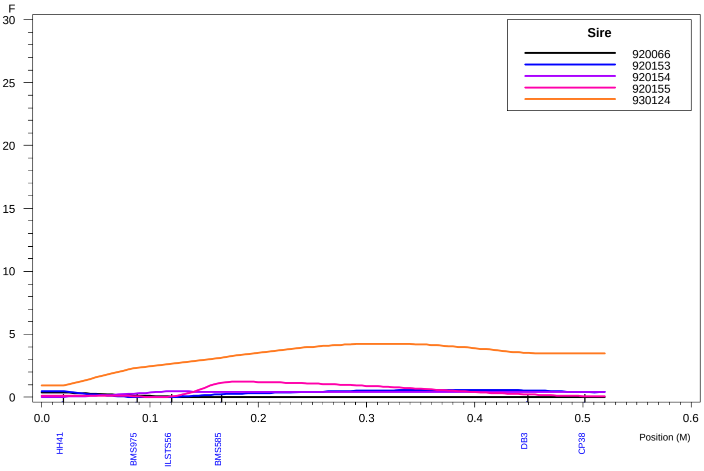

Haley-Knott QTL Analysis: Chromosome 10
WTFEC2

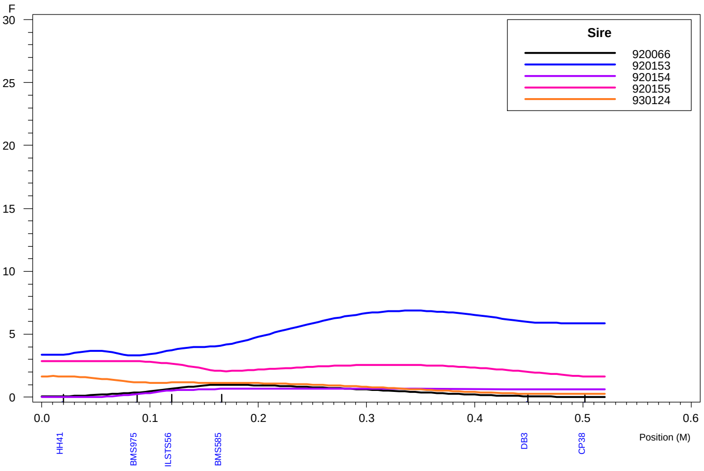

Supplement: Additional File 15 — Chr 10. Haley Knott linkage analysis of sheep chromosome 10. [file 1471-2164-7-178-S15.pdf]
