# Supplementary material for: Discovery of quantitative trait loci for resistance to parasitic nematode infection in sheep: I. Analysis of outcross pedigrees
Source: BMC Genomics. 2006 Jul 18;7:178. doi: 10.1186/1471-2164-7-178 (PMC1574317; doi:10.1186/1471-2164-7-178)

Linkage Analysis in the Parasite Outcross Flock: Chromosome 11

Information Content: Chromosome 11

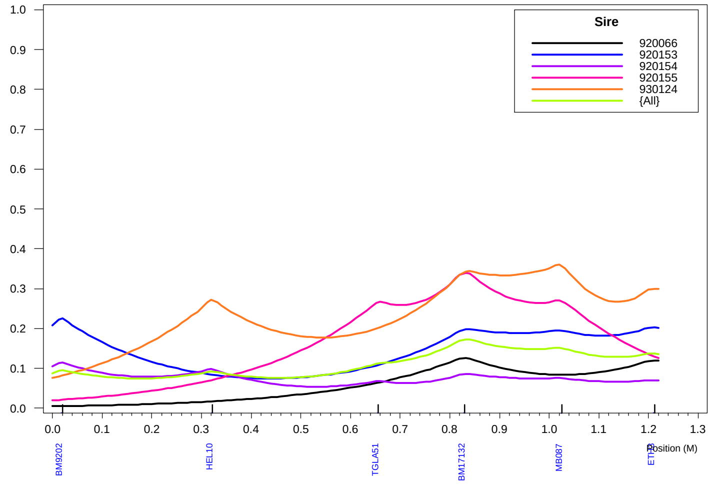

Haley-Knott QTL Analysis: Chromosome 11

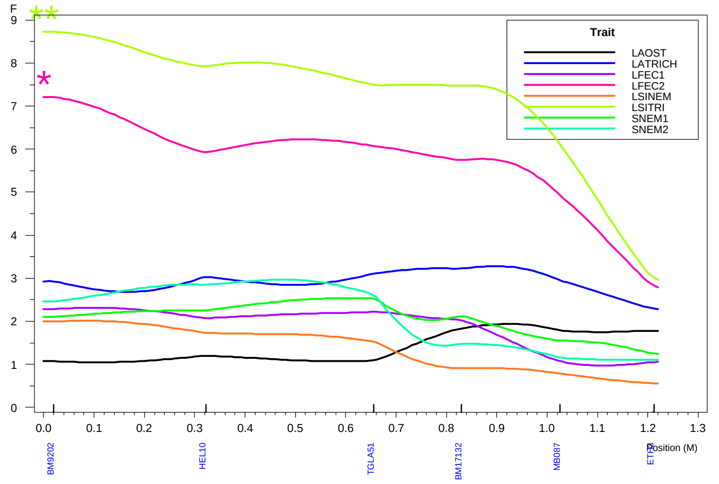

Haley-Knott QTL Analysis: Chromosome 11

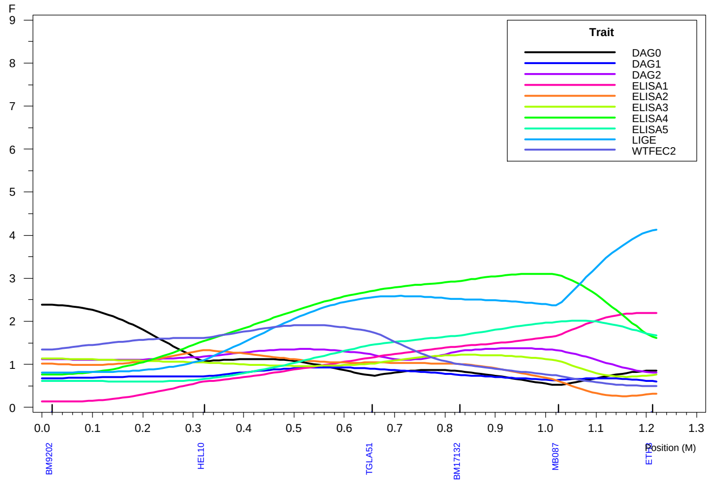

Haley-Knott QTL Analysis: Chromosome 11
LFEC1

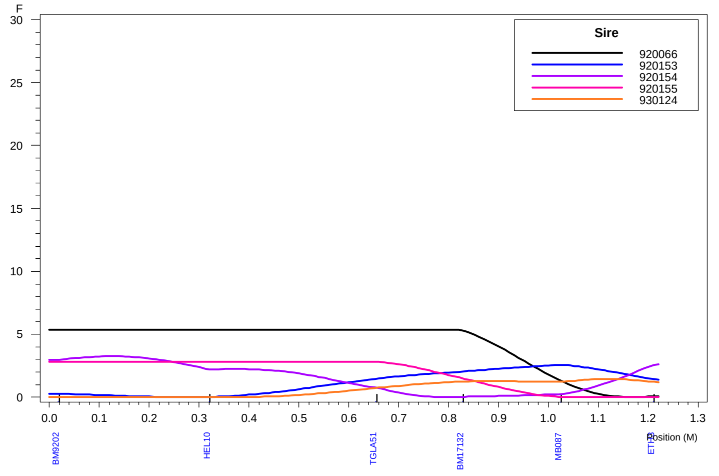

Haley-Knott QTL Analysis: Chromosome 11
LFEC2

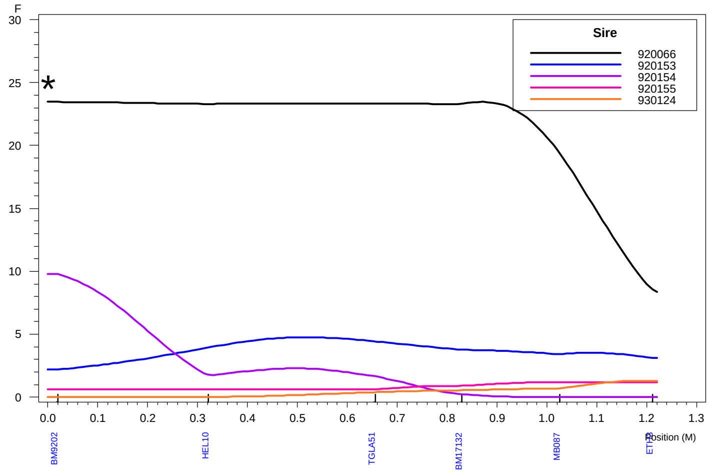

Haley-Knott QTL Analysis: Chromosome 11
SNEM1

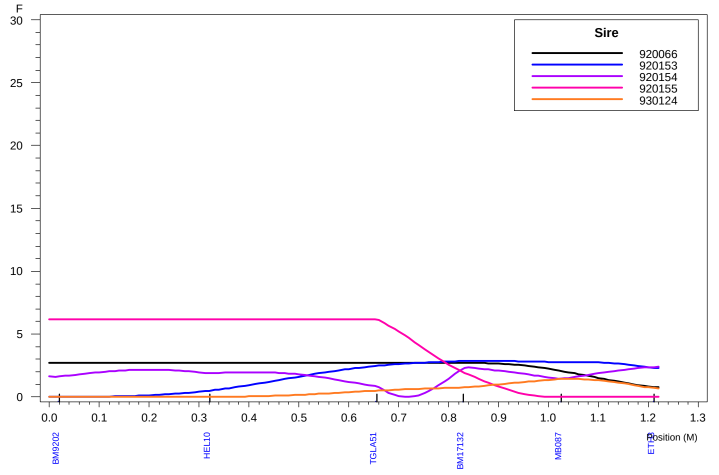

Haley-Knott QTL Analysis: Chromosome 11
SNEM2

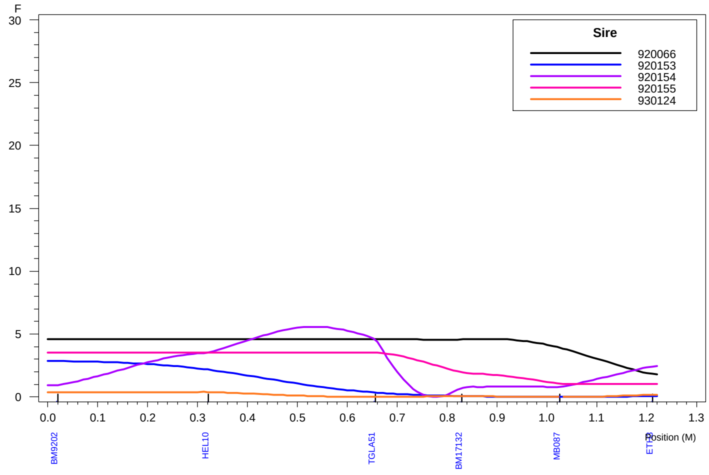

Haley-Knott QTL Analysis: Chromosome 11
LSINEM

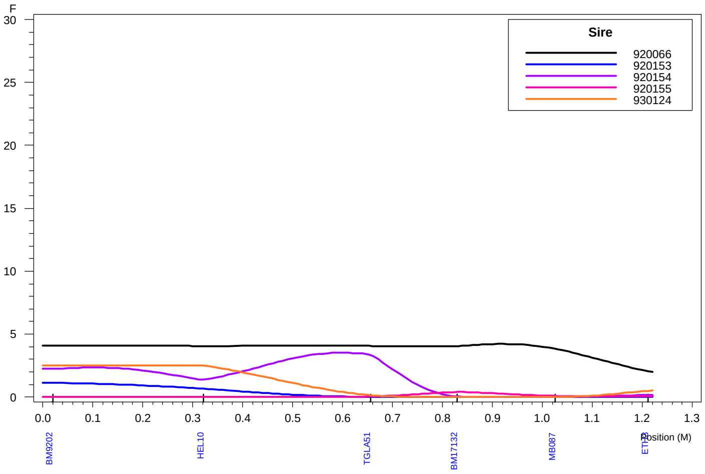

Haley-Knott QTL Analysis: Chromosome 11
LSITRI

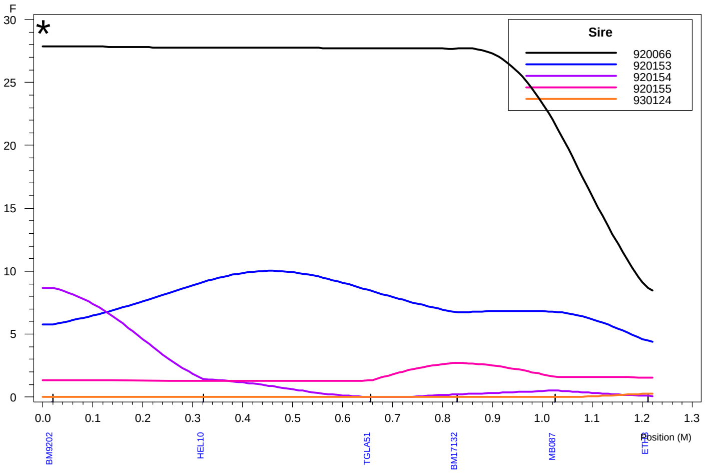

Haley-Knott QTL Analysis: Chromosome 11
LAOST

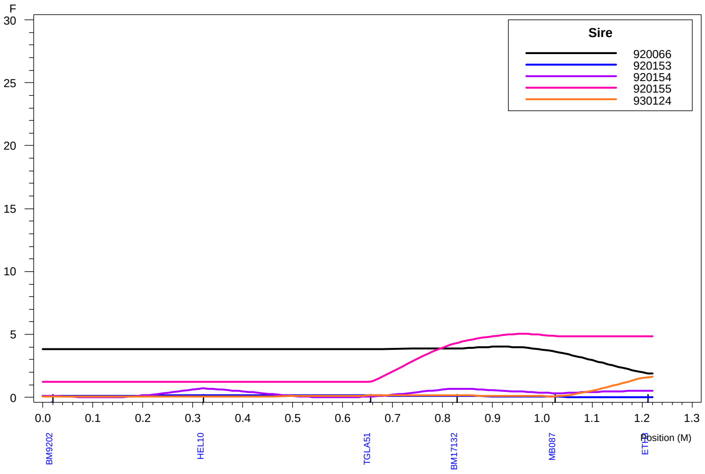

Haley-Knott QTL Analysis: Chromosome 11
LATRICH

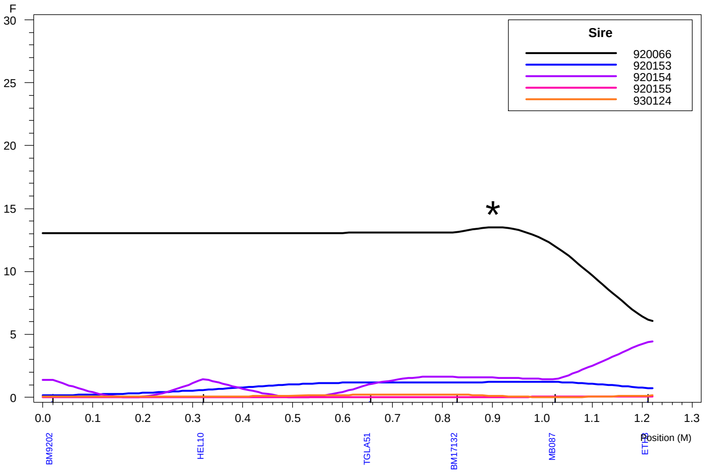

Haley-Knott QTL Analysis: Chromosome 11
DAG0

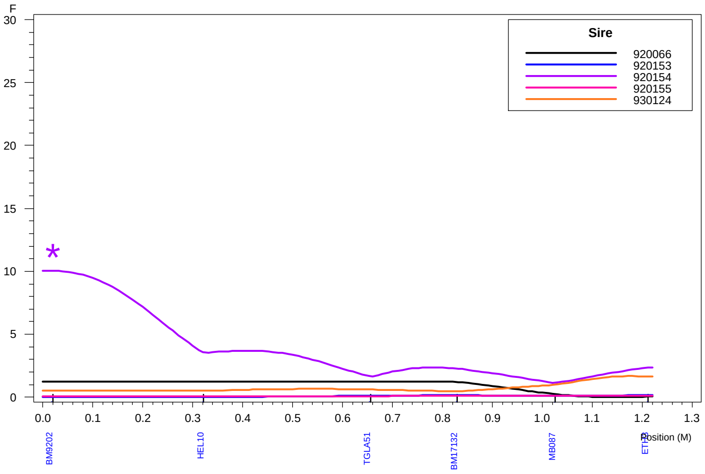

Haley-Knott QTL Analysis: Chromosome 11
DAG1

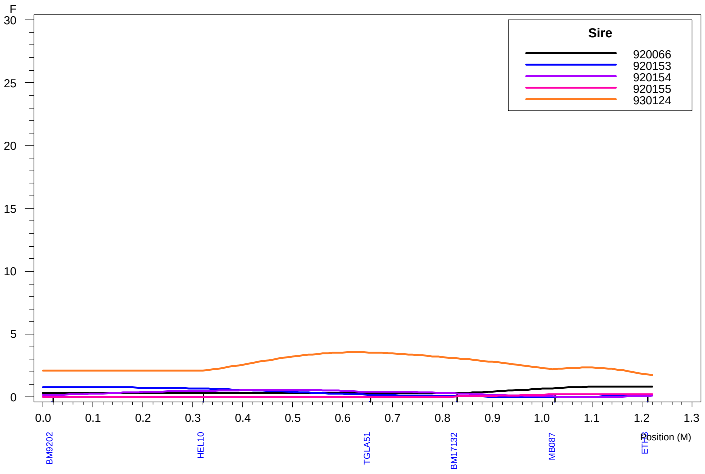

Haley-Knott QTL Analysis: Chromosome 11
DAG2

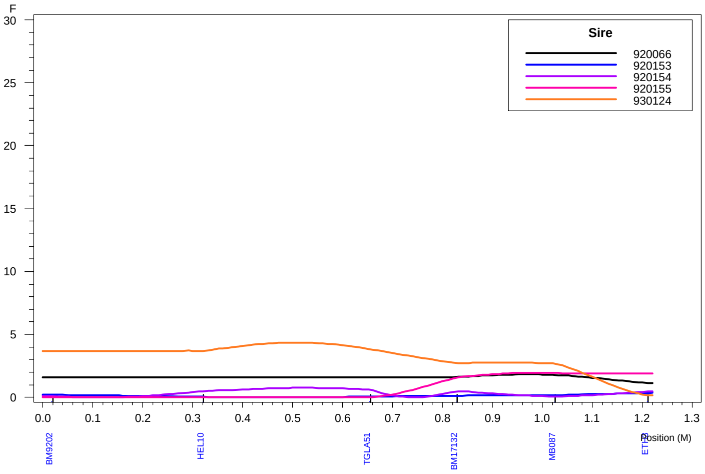

Haley-Knott QTL Analysis: Chromosome 11
ELISA1

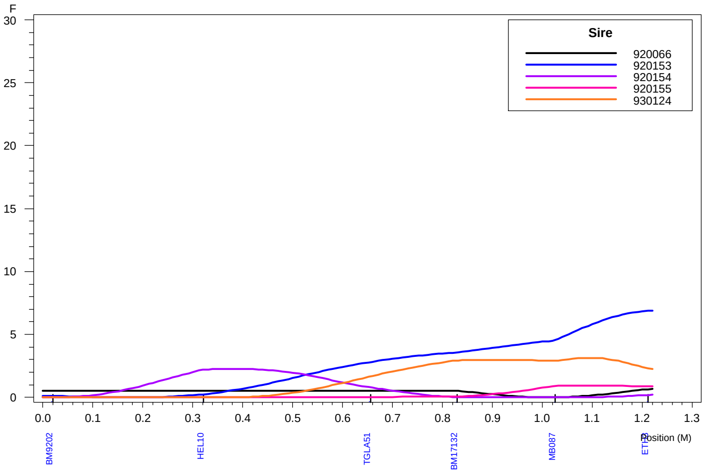

Haley-Knott QTL Analysis: Chromosome 11
ELISA2

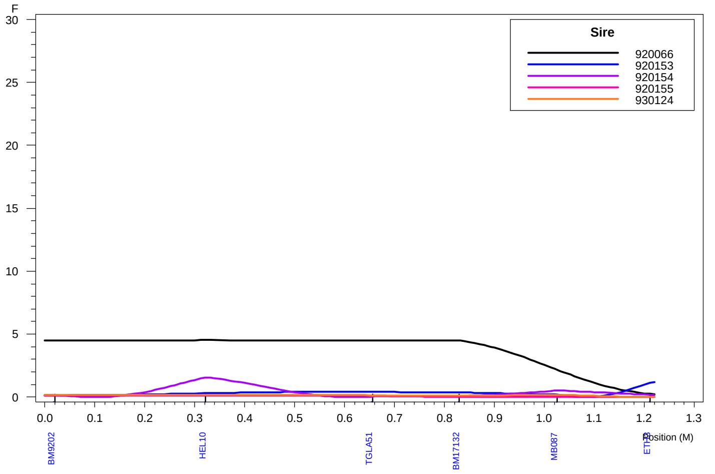

Haley-Knott QTL Analysis: Chromosome 11
ELISA3

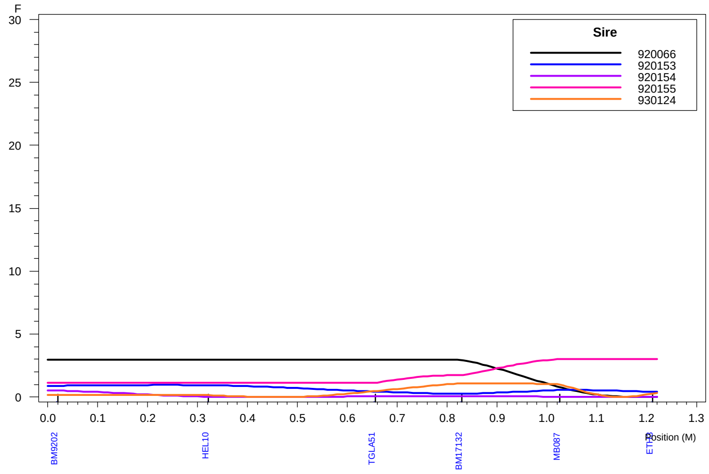

Haley-Knott QTL Analysis: Chromosome 11
ELISA4

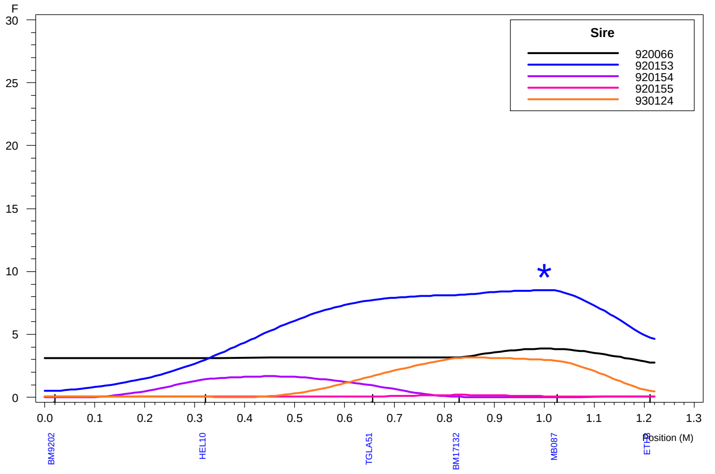

Haley-Knott QTL Analysis: Chromosome 11
ELISA5

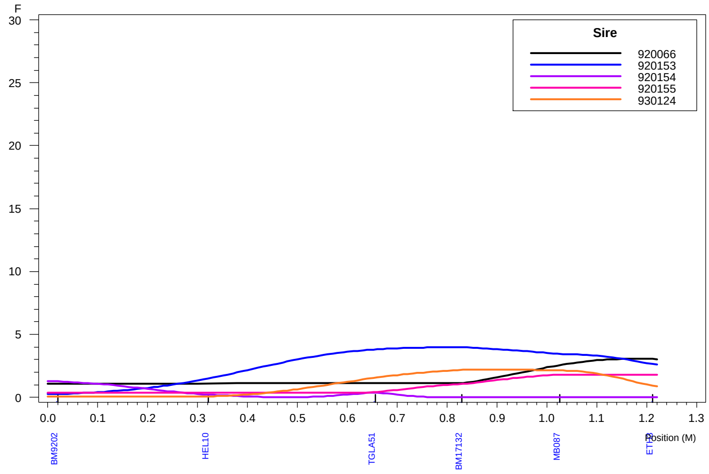

Haley-Knott QTL Analysis: Chromosome 11
LIGE

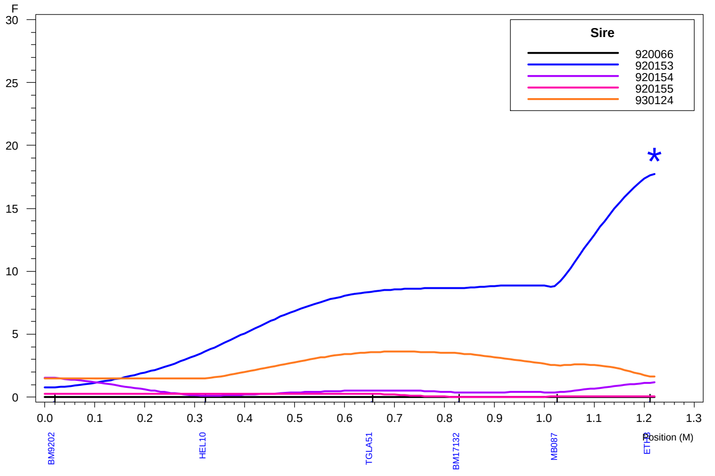

Haley-Knott QTL Analysis: Chromosome 11
WTFEC2

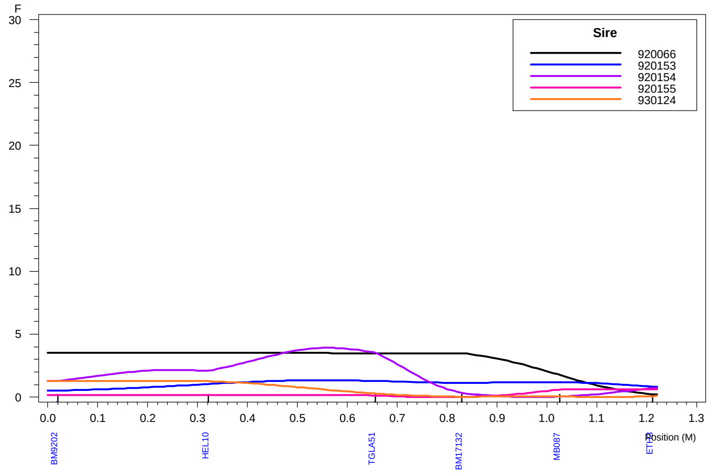

Supplement: Additional File 16 — Chr 11. Haley Knott linkage analysis of sheep chromosome 11. [file 1471-2164-7-178-S16.pdf]
