# Supplementary material for: Discovery of quantitative trait loci for resistance to parasitic nematode infection in sheep: I. Analysis of outcross pedigrees
Source: BMC Genomics. 2006 Jul 18;7:178. doi: 10.1186/1471-2164-7-178 (PMC1574317; doi:10.1186/1471-2164-7-178)

Linkage Analysis in the Parasite Outcross Flock: Chromosome 12

Information Content: Chromosome 12

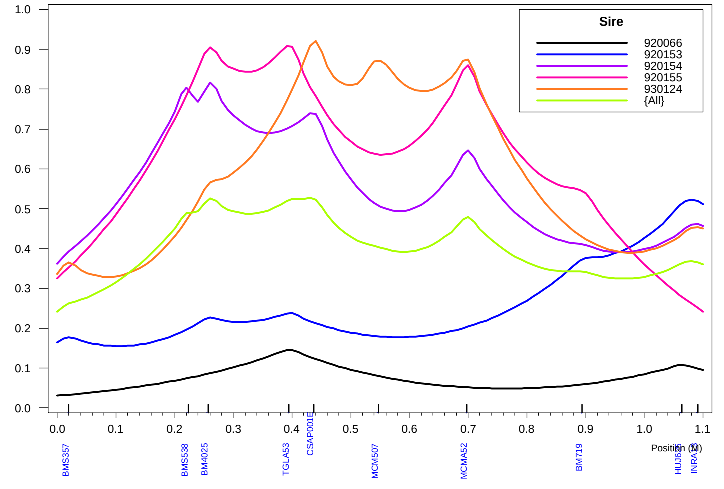

Haley-Knott QTL Analysis: Chromosome 12

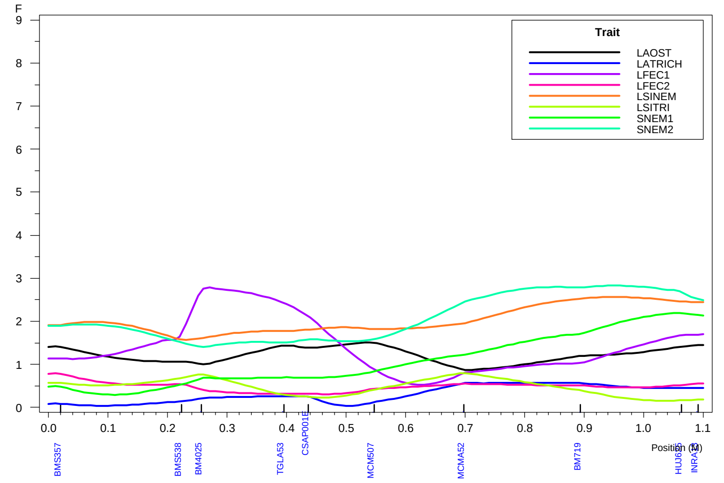

Haley-Knott QTL Analysis: Chromosome 12

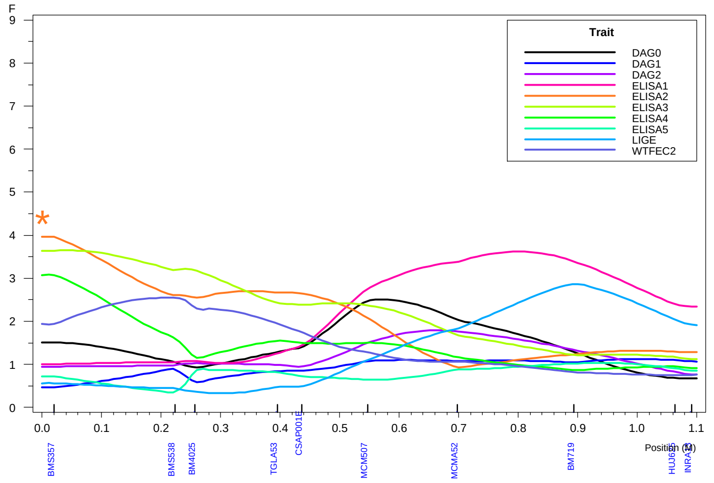

Haley-Knott QTL Analysis: Chromosome 12
LFEC1

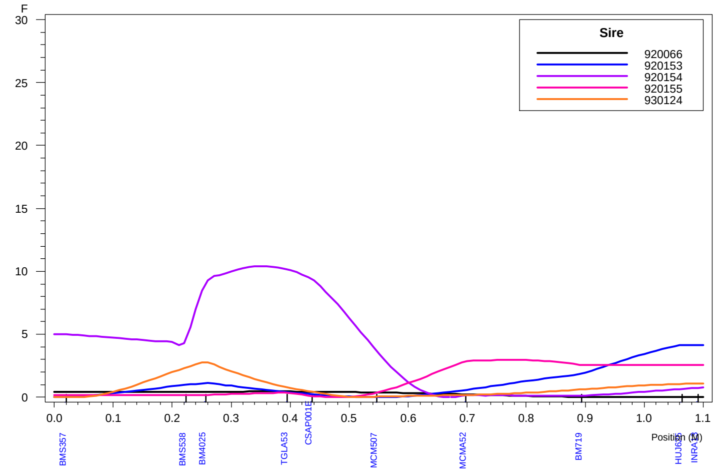

Haley-Knott QTL Analysis: Chromosome 12
LFEC2

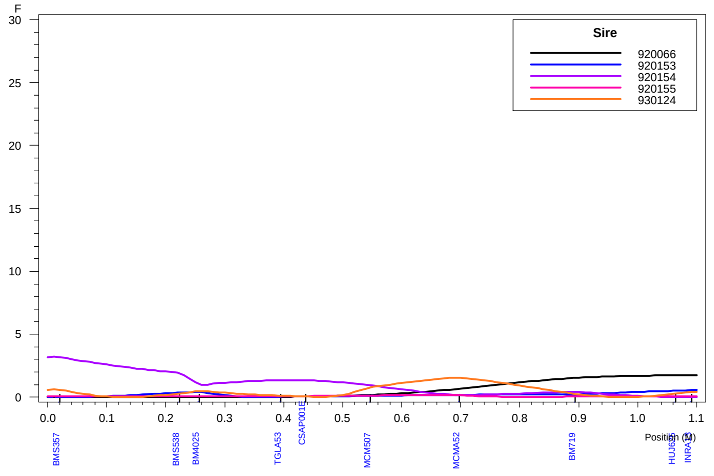

Haley-Knott QTL Analysis: Chromosome 12
SNEM1

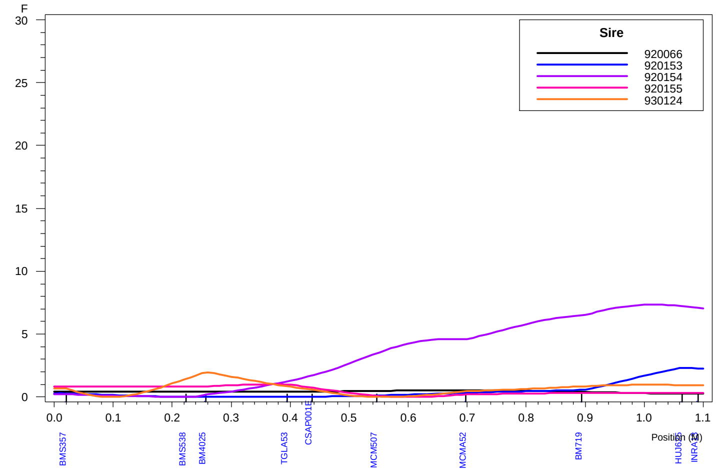

Haley-Knott QTL Analysis: Chromosome 12
SNEM2

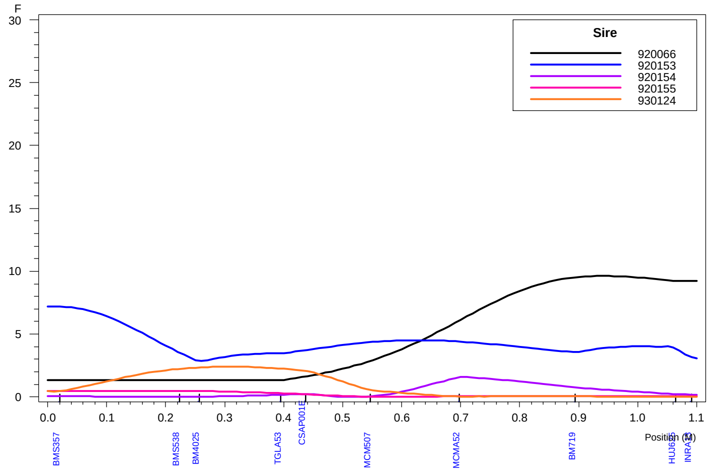

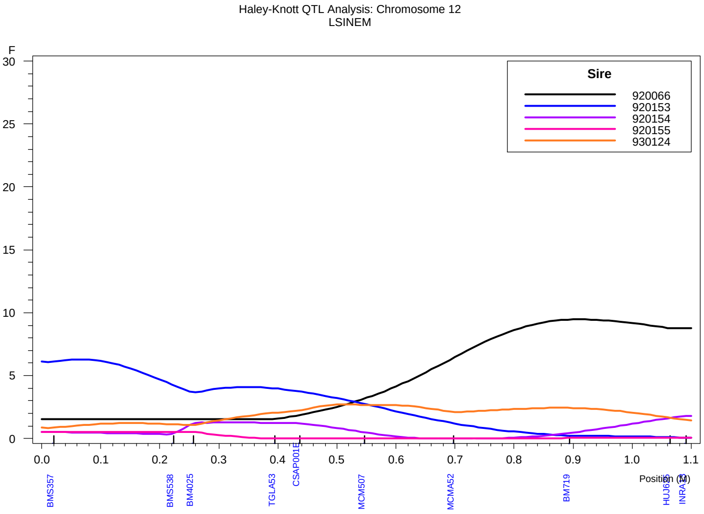
Haley-Knott QTL Analysis: Chromosome 12
LSINEM
Sire
920066
920153
920154
920155
930124
F
Position (M)
BMS357
BMS538
BM4025
TGLA53
CSAP001E
MCM507
MCMA52
BM719

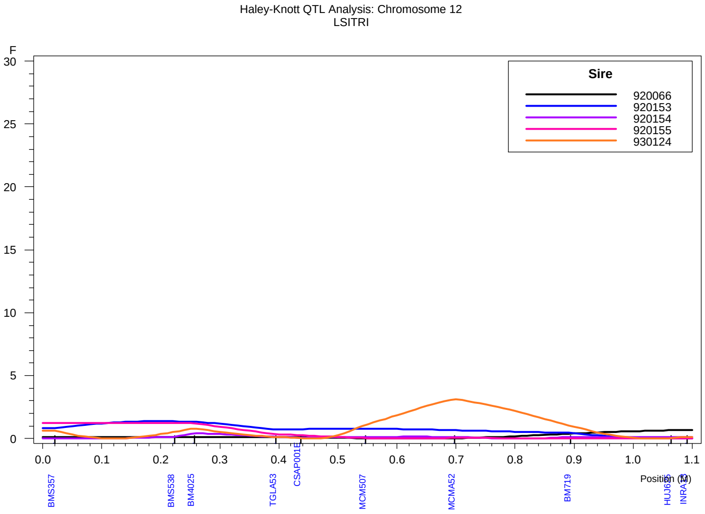
Haley-Knott QTL Analysis: Chromosome 12
LSITRI
Sire
920066
920153
920154
920155
930124
F
Position (M)
BMS357
BMS538
BM4025
TGLA53
CSAP001E
MCM507
MCMA52
BM719

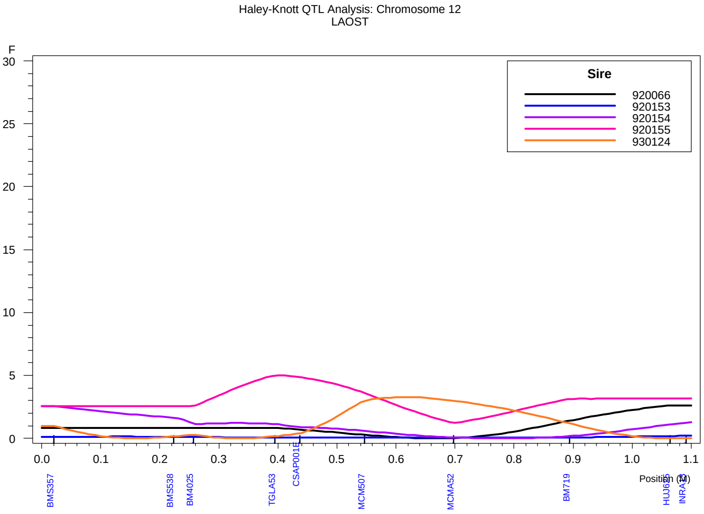
Haley-Knott QTL Analysis: Chromosome 12
LAOST
Sire
920066
920153
920154
920155
930124
F
Position (M)
BMS357
BMS538
BM4025
TGLA53
CSAP001E
MCM507
MCMA52
BM719

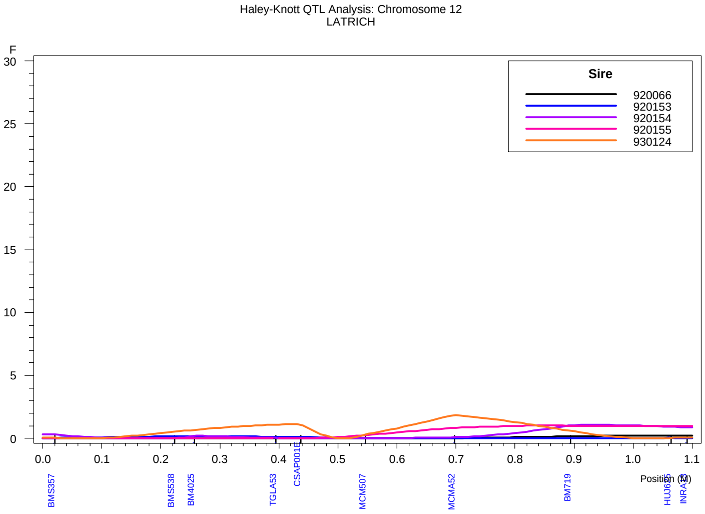
Haley-Knott QTL Analysis: Chromosome 12
LATRICH
Sire
920066
920153
920154
920155
930124
F
Position (M)
BMS357
BMS538
BM4025
TGLA53
CSAP001E
MCM507
MCMA52
BM719

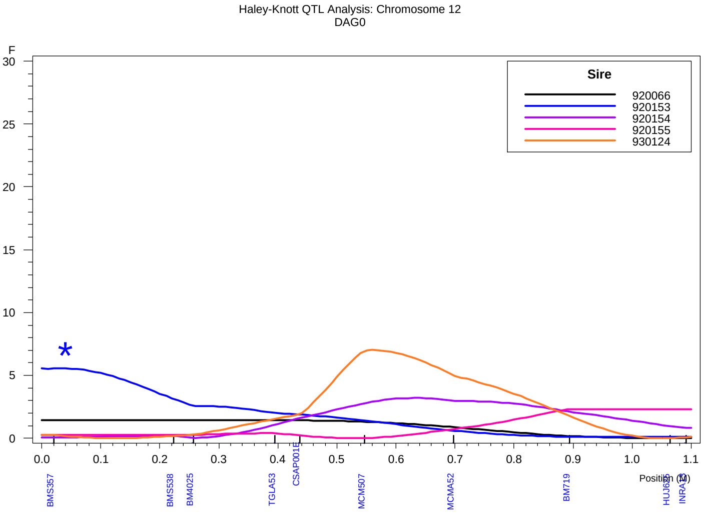
Haley-Knott QTL Analysis: Chromosome 12
DAG0
Sire
920066
920153
920154
920155
930124
F
Position (M)
BMS357
BMS538
BM4025
TGLA53
CSAP001E
MCM507
MCMA52
BM719

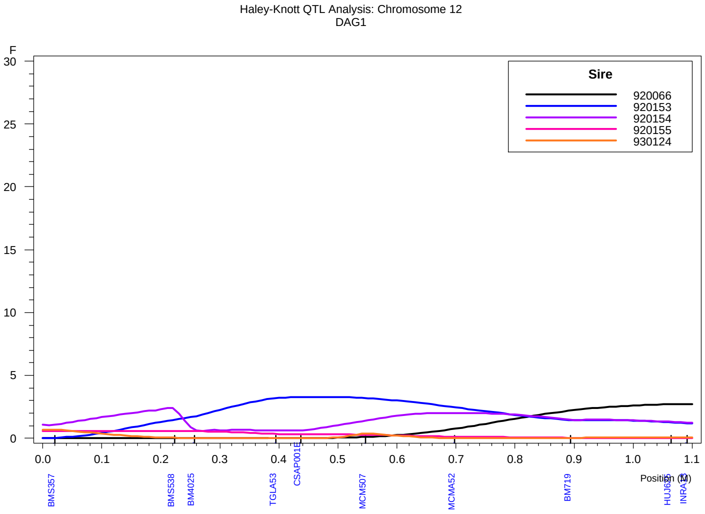
Haley-Knott QTL Analysis: Chromosome 12
DAG1
Sire
920066
920153
920154
920155
930124
F
Position (M)
BMS357
BMS538
BM4025
TGLA53
CSAP001E
MCM507
MCMA52
BM719

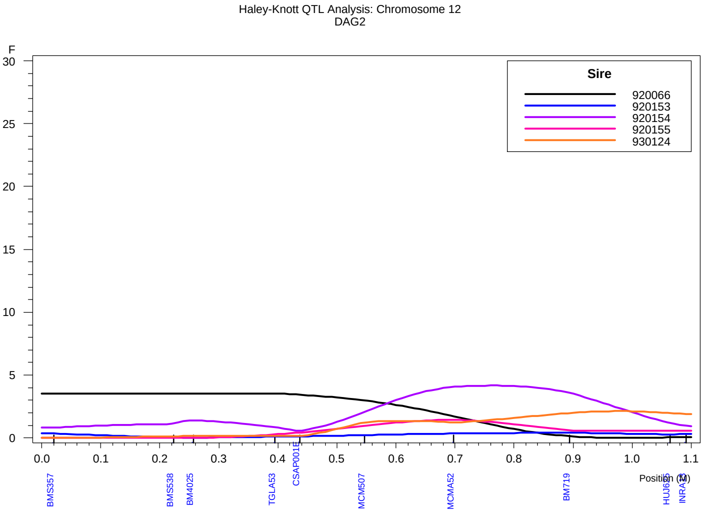
Haley-Knott QTL Analysis: Chromosome 12
DAG2
Sire
920066
920153
920154
920155
930124
F
Position (M)
BMS357
BMS538
BM4025
TGLA53
CSAP001E
MCM507
MCMA52
BM719

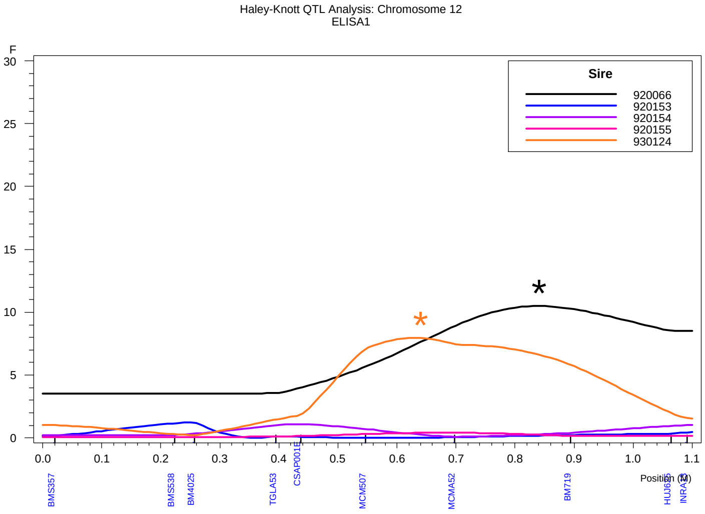
Haley-Knott QTL Analysis: Chromosome 12
ELISA1
Sire
920066
920153
920154
920155
930124
F
Position (M)
BMS357
BMS538
BM4025
TGLA53
CSAP001E
MCM507
MCMA52
BM719

Haley-Knott QTL Analysis: Chromosome 12
ELISA2

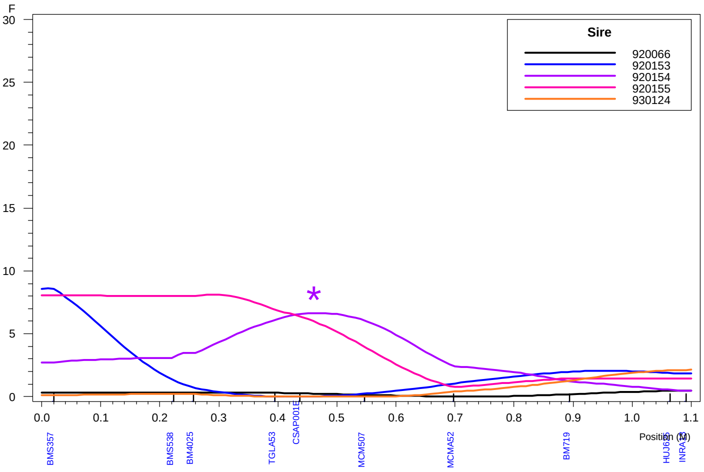

Haley-Knott QTL Analysis: Chromosome 12
ELISA3

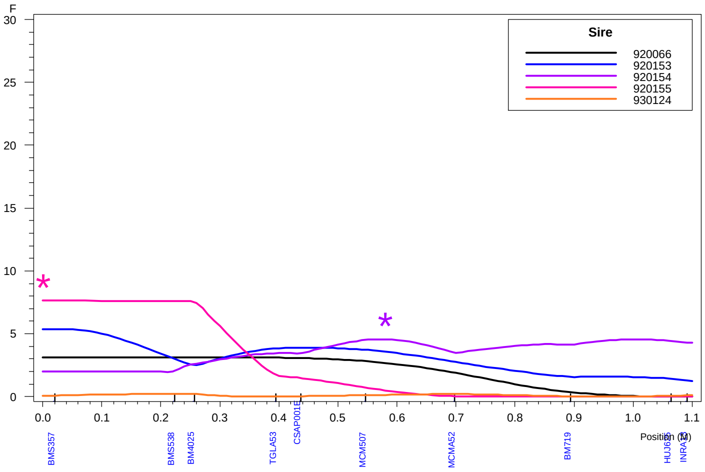

Haley-Knott QTL Analysis: Chromosome 12
ELISA4

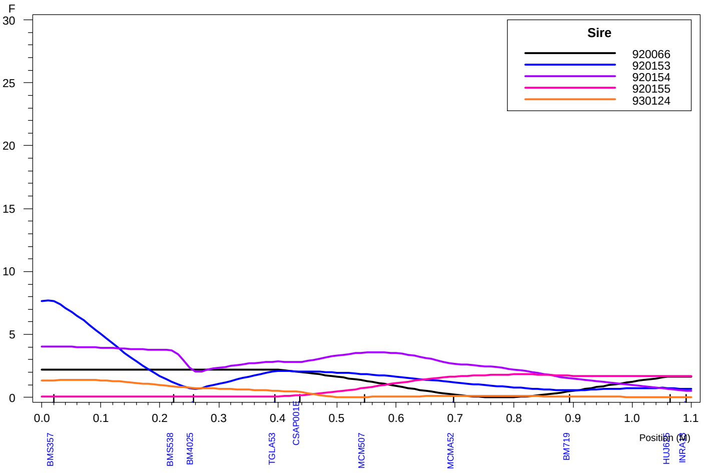

Haley-Knott QTL Analysis: Chromosome 12
ELISA5

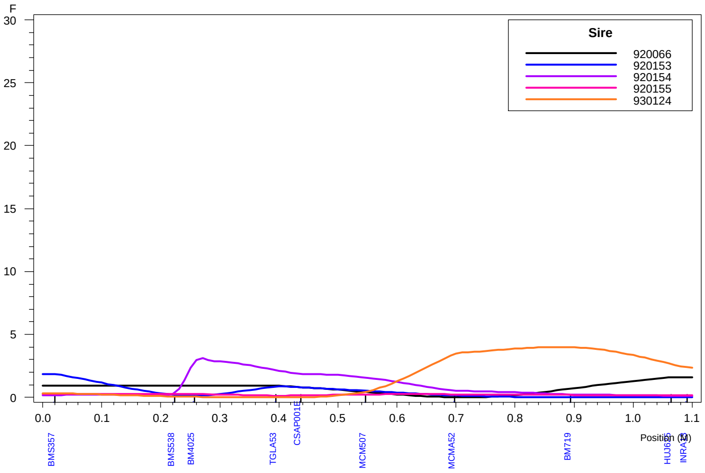

Haley-Knott QTL Analysis: Chromosome 12
LIGE

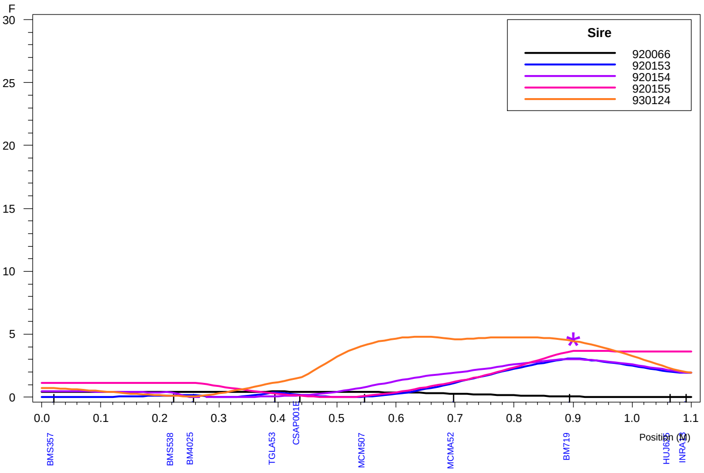

Haley-Knott QTL Analysis: Chromosome 12
WTFEC2

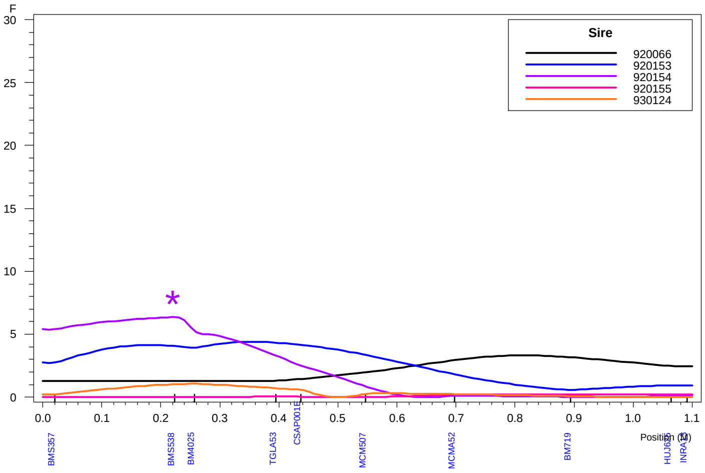

Supplement: Additional File 17 — Chr 12. Haley Knott linkage analysis of sheep chromosome 12. [file 1471-2164-7-178-S17.pdf]
