# Supplementary material for: Discovery of quantitative trait loci for resistance to parasitic nematode infection in sheep: I. Analysis of outcross pedigrees
Source: BMC Genomics. 2006 Jul 18;7:178. doi: 10.1186/1471-2164-7-178 (PMC1574317; doi:10.1186/1471-2164-7-178)
Supplement: Additional File 18 — Chr 13. Haley Knott linkage analysis of sheep chromosome 13. [file 1471-2164-7-178-S18.pdf]

Linkage Analysis in the Parasite Outcross Flock: Chromosome 13

Information Content: Chromosome 13

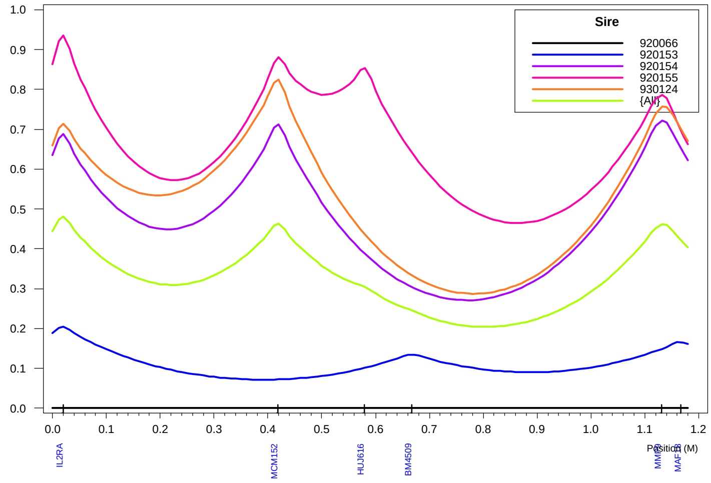

Haley-Knott QTL Analysis: Chromosome 13

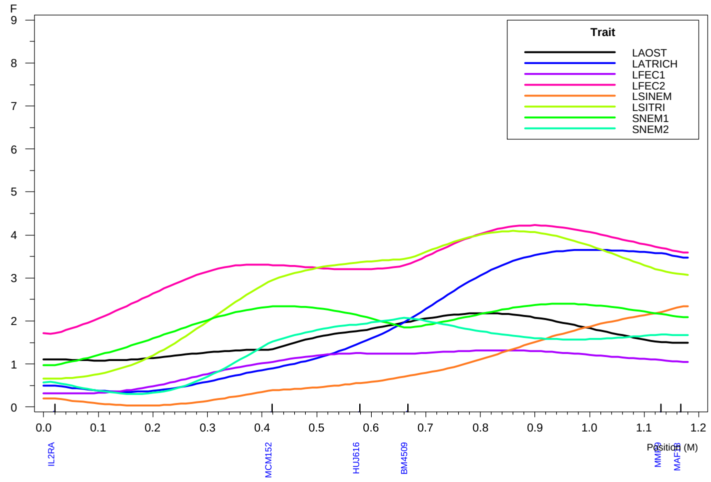

Haley-Knott QTL Analysis: Chromosome 13

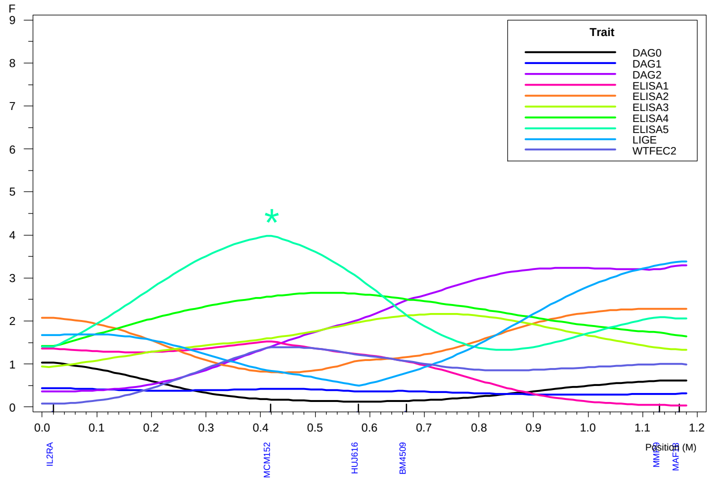

Haley-Knott QTL Analysis: Chromosome 13
LFEC1

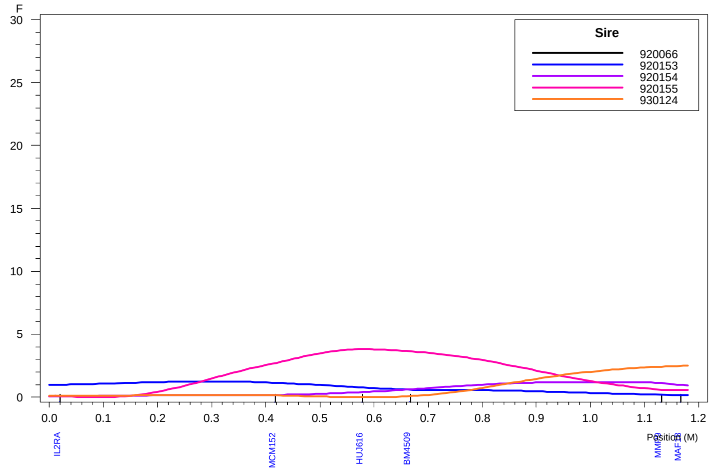

Haley-Knott QTL Analysis: Chromosome 13
LFEC2

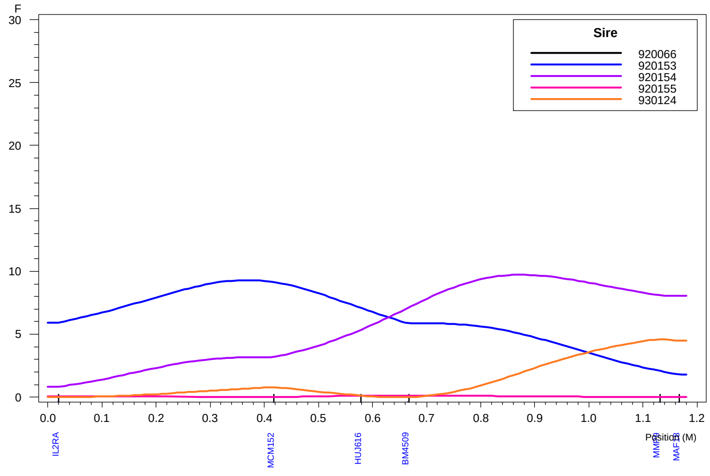

Haley-Knott QTL Analysis: Chromosome 13
SNEM1

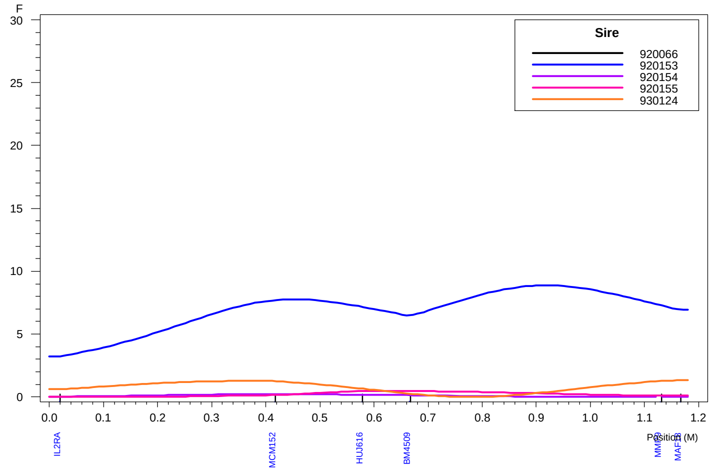

Haley-Knott QTL Analysis: Chromosome 13
SNEM2

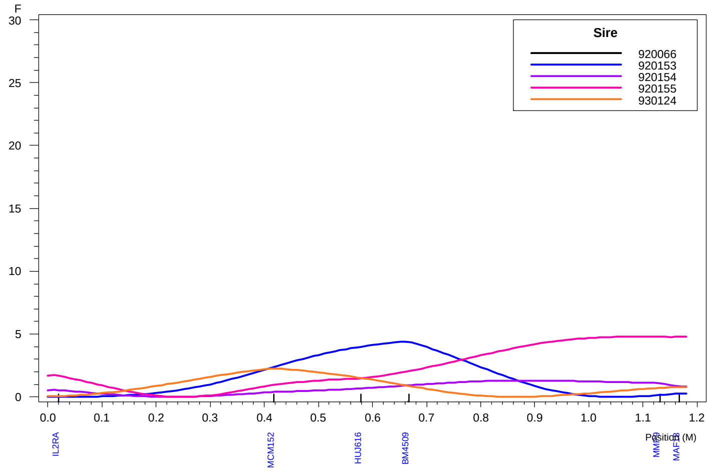

Haley-Knott QTL Analysis: Chromosome 13
LSINEM

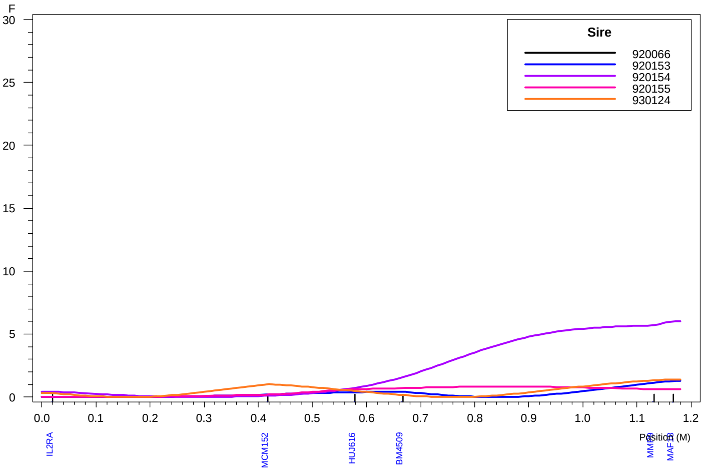

Haley-Knott QTL Analysis: Chromosome 13
LSITRI

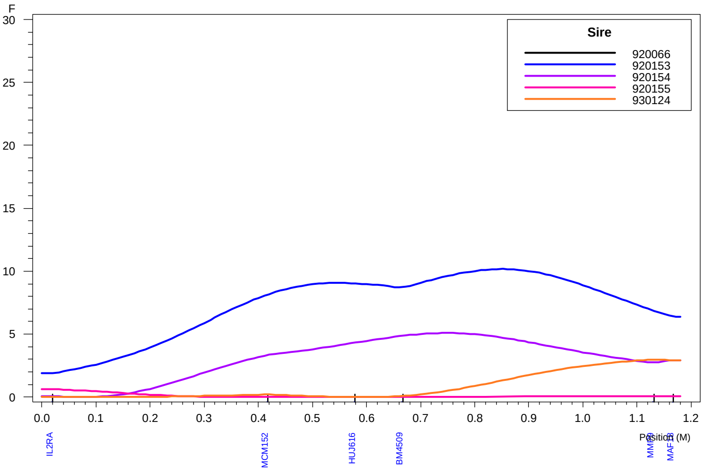

Haley-Knott QTL Analysis: Chromosome 13
LAOST

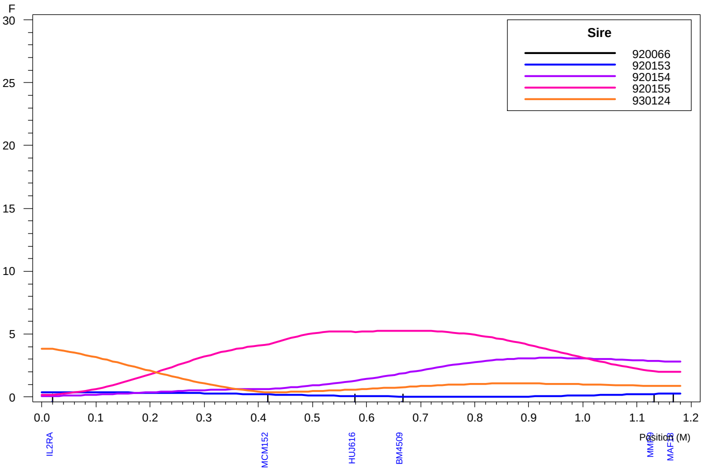

Haley-Knott QTL Analysis: Chromosome 13
LATRICH

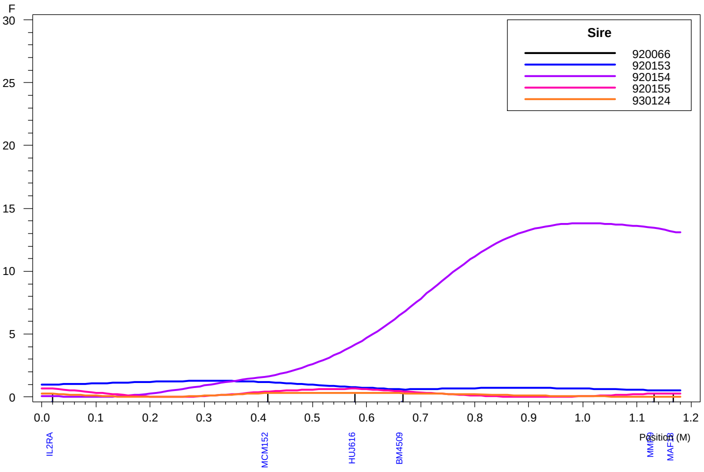

Haley-Knott QTL Analysis: Chromosome 13
DAG0

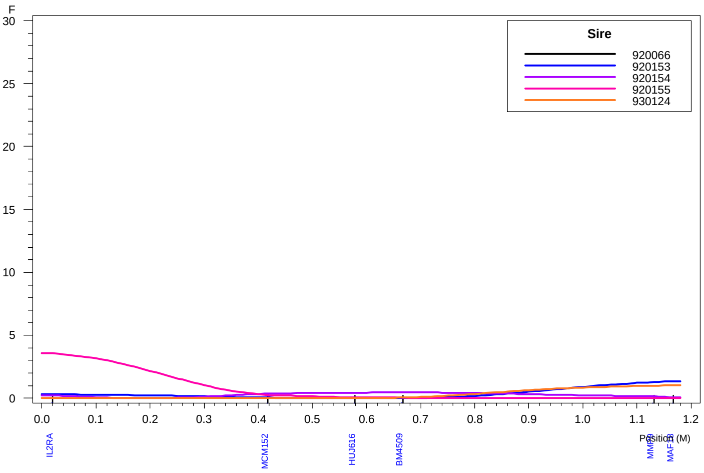

Haley-Knott QTL Analysis: Chromosome 13
DAG1

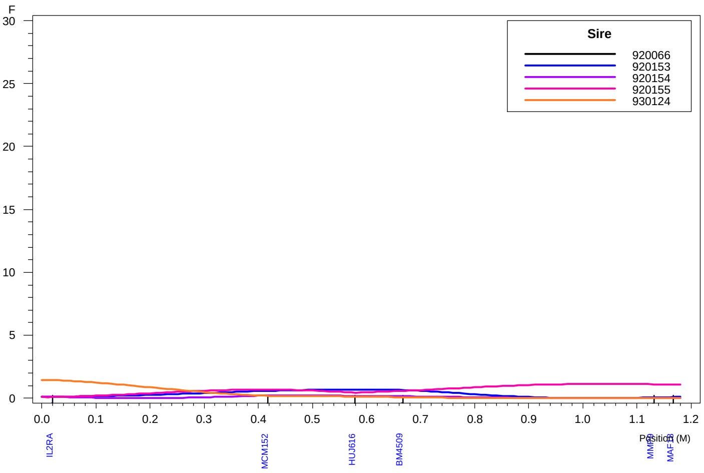

Haley-Knott QTL Analysis: Chromosome 13
DAG2

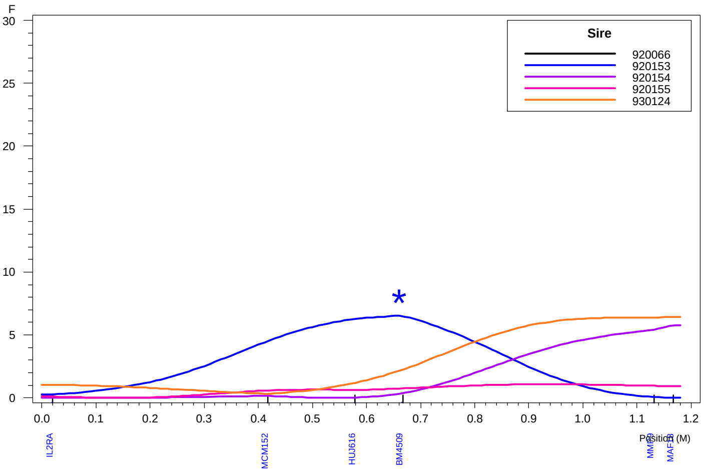

Haley-Knott QTL Analysis: Chromosome 13
ELISA1

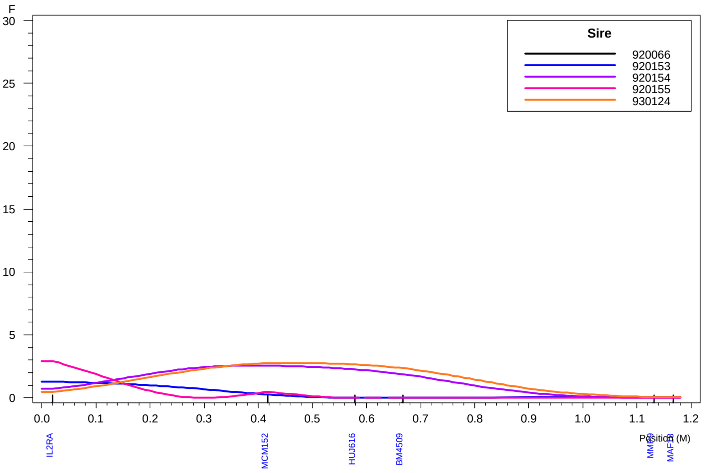

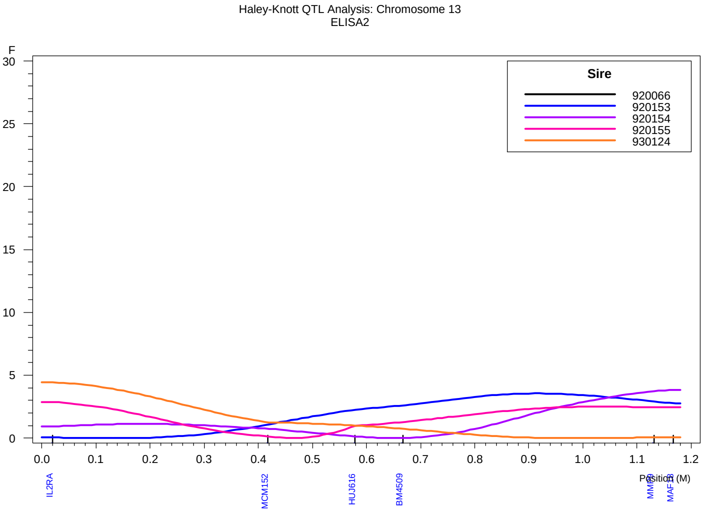
Haley-Knott QTL Analysis: Chromosome 13
ELISA2
F
Sire
920066
920153
920154
920155
930124
Position (M)
IL2RA
MCM152
HUJ616
BM4509

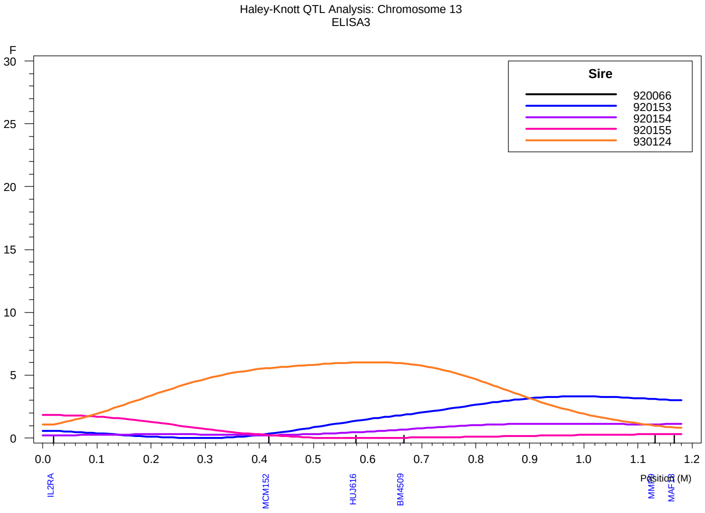
Haley-Knott QTL Analysis: Chromosome 13
ELISA3
F
Sire
920066
920153
920154
920155
930124
Position (M)
IL2RA
MCM152
HUJ616
BM4509

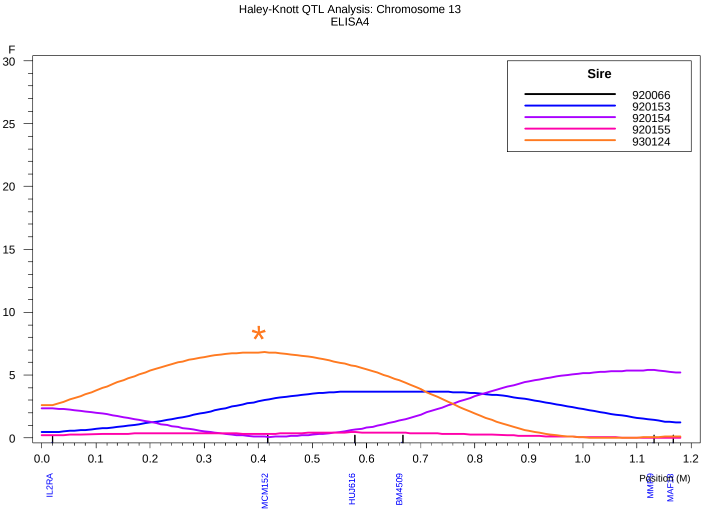
Haley-Knott QTL Analysis: Chromosome 13
ELISA4
F
Sire
920066
920153
920154
920155
930124
Position (M)
IL2RA
MCM152
HUJ616
BM4509

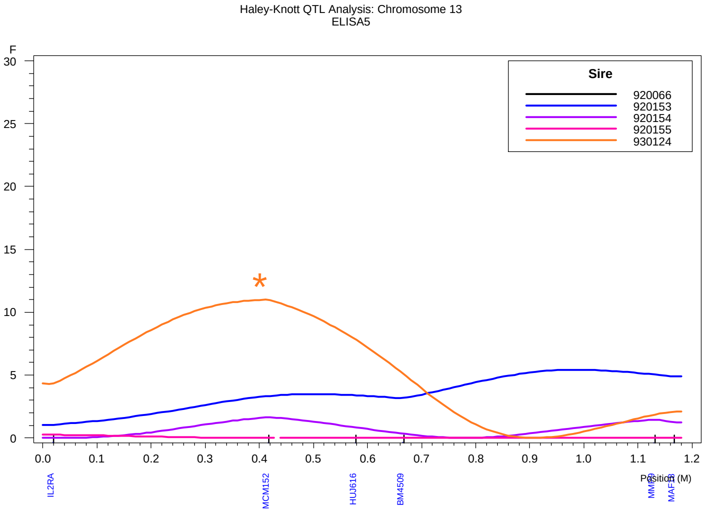
Haley-Knott QTL Analysis: Chromosome 13
ELISA5
F
Sire
920066
920153
920154
920155
930124
Position (M)
IL2RA
MCM152
HUJ616
BM4509

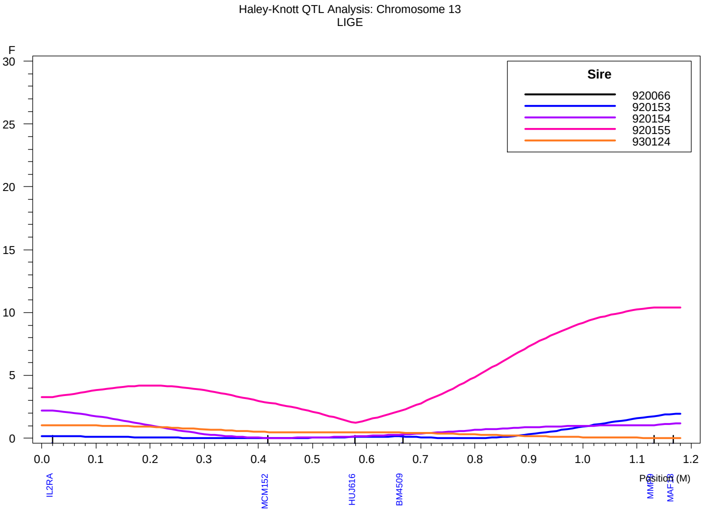
Haley-Knott QTL Analysis: Chromosome 13
LIGE
F
Sire
920066
920153
920154
920155
930124
Position (M)
IL2RA
MCM152
HUJ616
BM4509

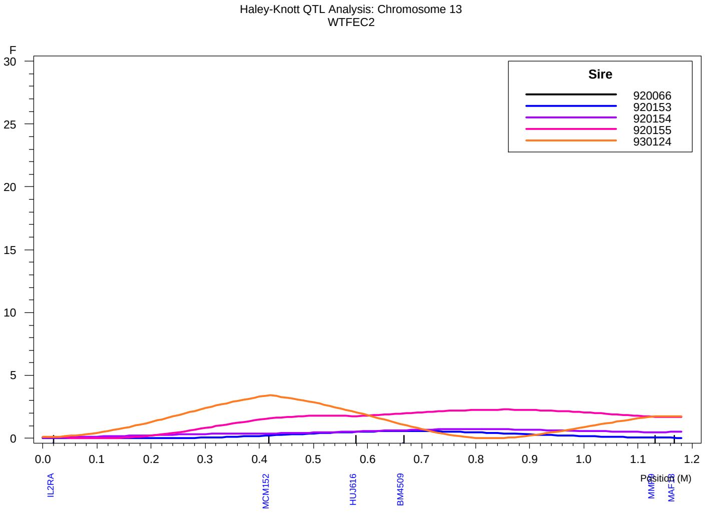
Haley-Knott QTL Analysis: Chromosome 13
WTFEC2
F
Sire
920066
920153
920154
920155
930124
Position (M)
IL2RA
MCM152
HUJ616
BM4509
