# Supplementary material for: Discovery of quantitative trait loci for resistance to parasitic nematode infection in sheep: I. Analysis of outcross pedigrees
Source: BMC Genomics. 2006 Jul 18;7:178. doi: 10.1186/1471-2164-7-178 (PMC1574317; doi:10.1186/1471-2164-7-178)

Linkage Analysis in the Parasite Outcross Flock: Chromosome 14

Information Content: Chromosome 14

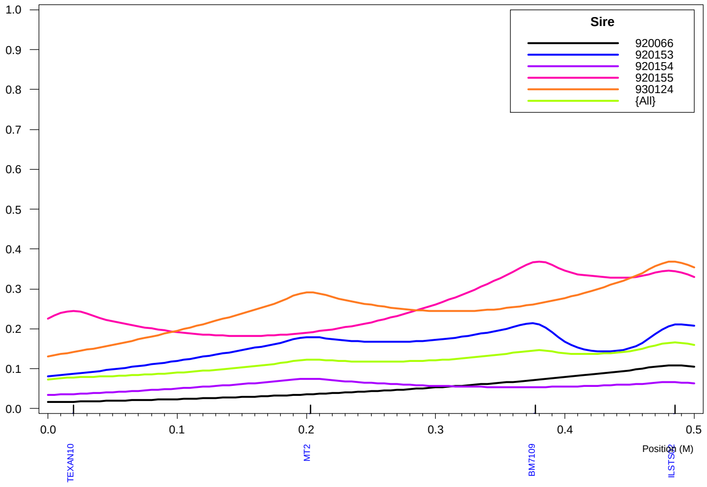

Haley-Knott QTL Analysis: Chromosome 14

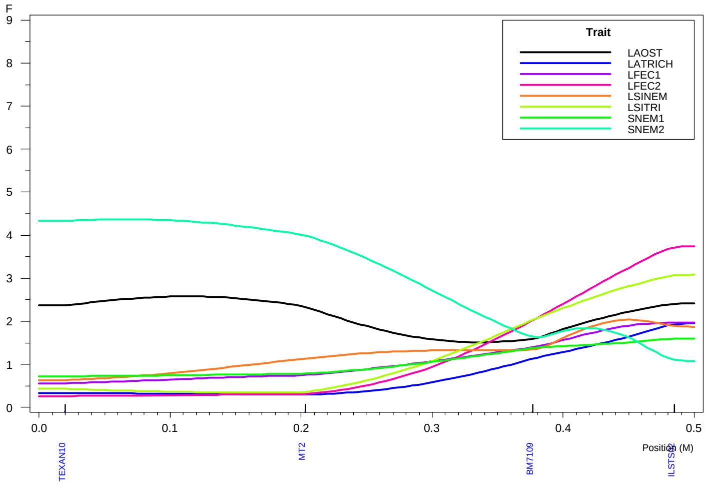

Haley-Knott QTL Analysis: Chromosome 14

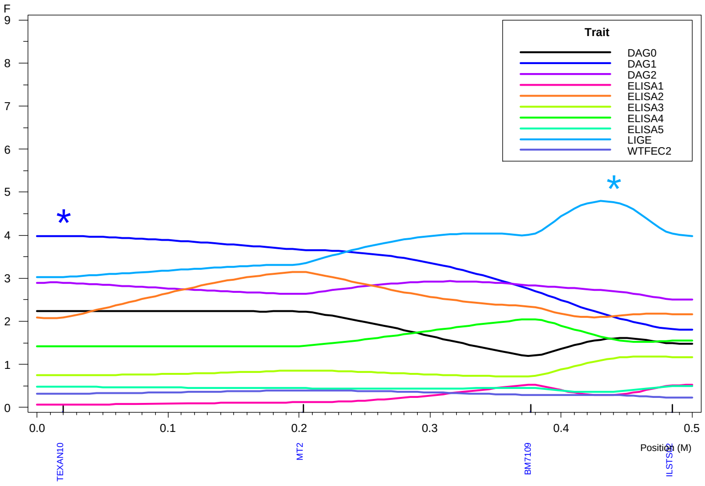

Haley-Knott QTL Analysis: Chromosome 14
LFEC1

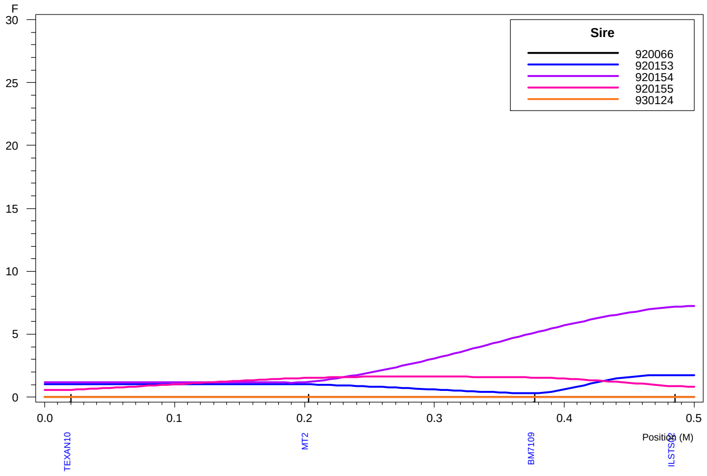

Haley-Knott QTL Analysis: Chromosome 14
LFEC2

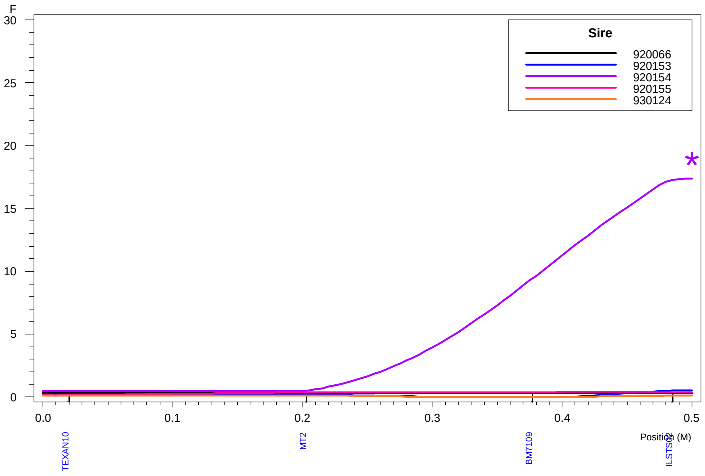

Haley-Knott QTL Analysis: Chromosome 14
SNEM1

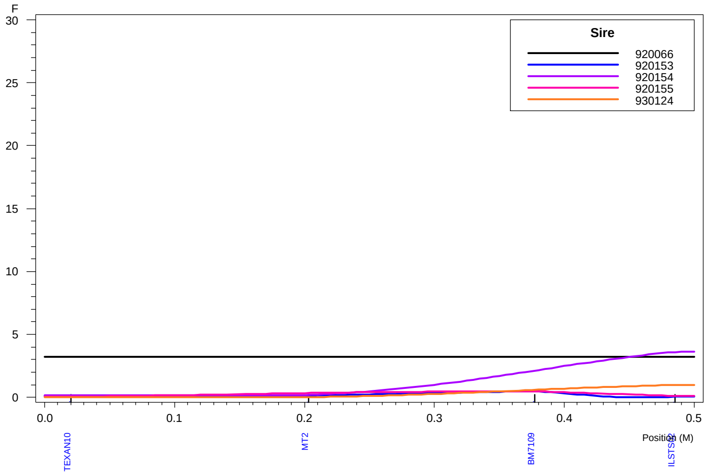

Haley-Knott QTL Analysis: Chromosome 14
SNEM2

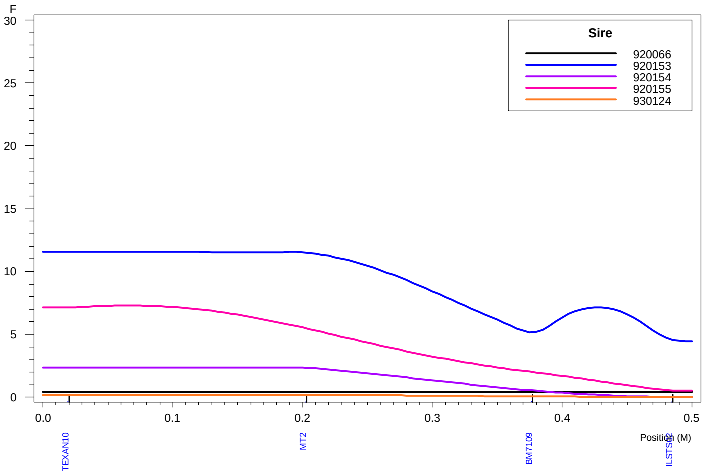

Haley-Knott QTL Analysis: Chromosome 14
LSINEM

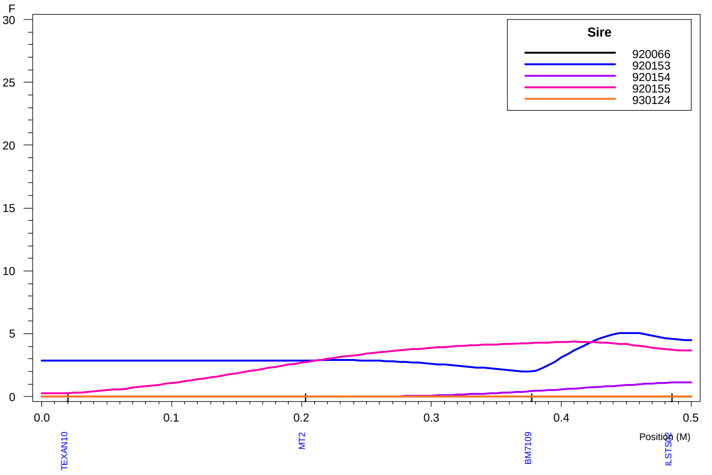

Haley-Knott QTL Analysis: Chromosome 14
LSITRI

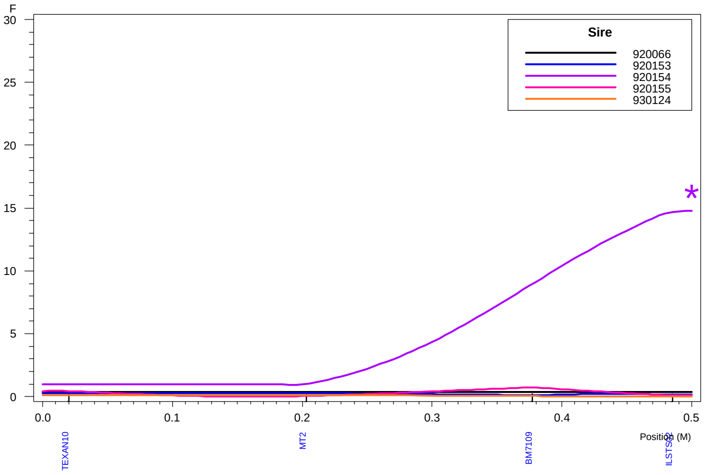

Haley-Knott QTL Analysis: Chromosome 14
LAOST

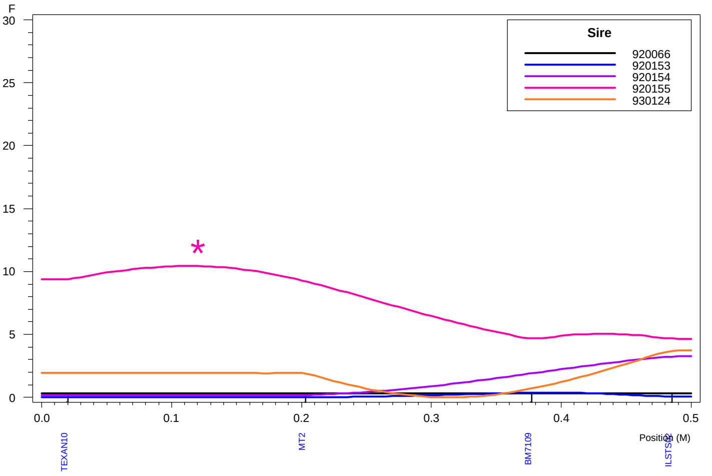

Haley-Knott QTL Analysis: Chromosome 14
LATRICH

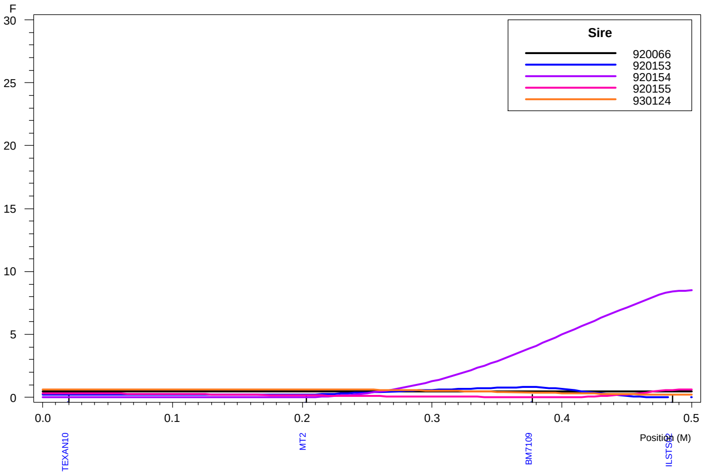

Haley-Knott QTL Analysis: Chromosome 14
DAG0

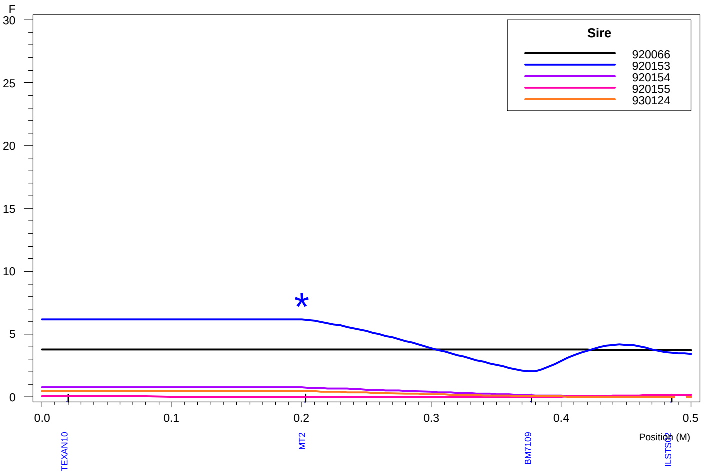

Haley-Knott QTL Analysis: Chromosome 14
DAG1

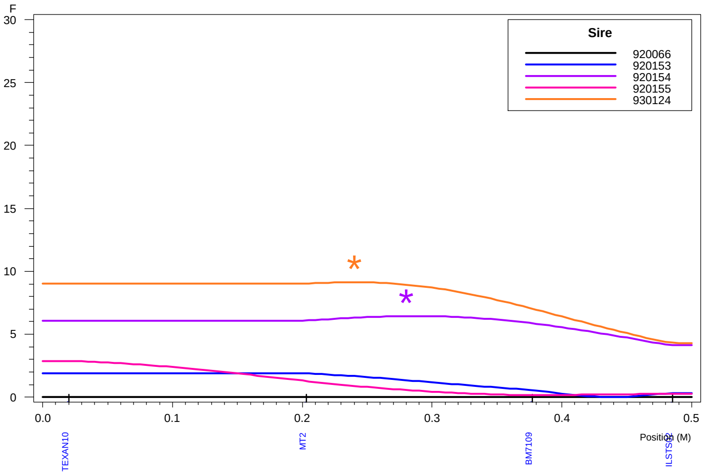

Haley-Knott QTL Analysis: Chromosome 14
DAG2

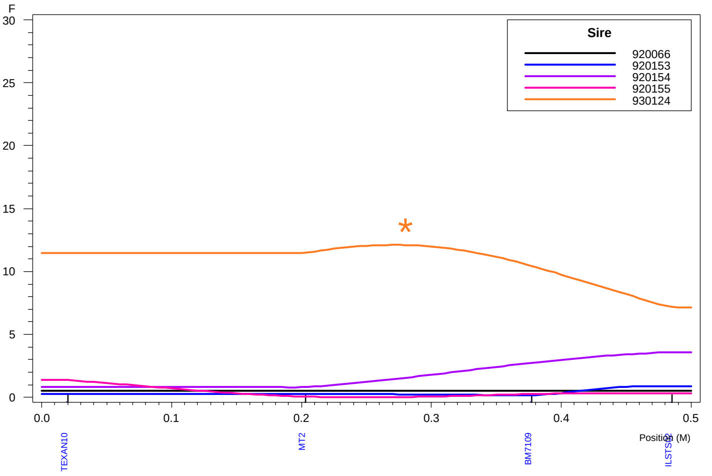

Haley-Knott QTL Analysis: Chromosome 14
ELISA1

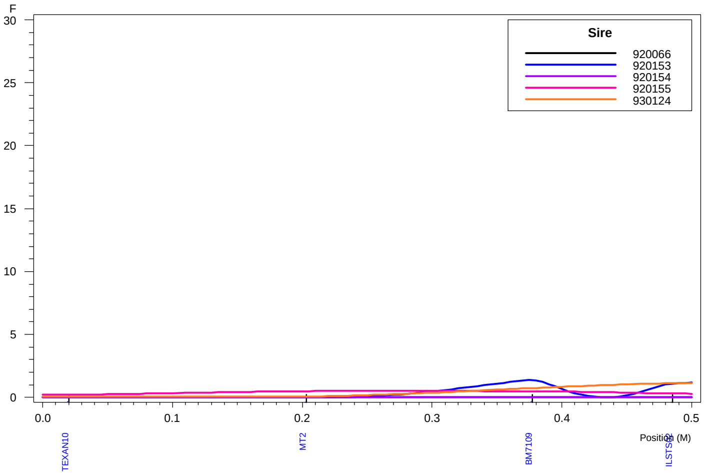

Haley-Knott QTL Analysis: Chromosome 14
ELISA2

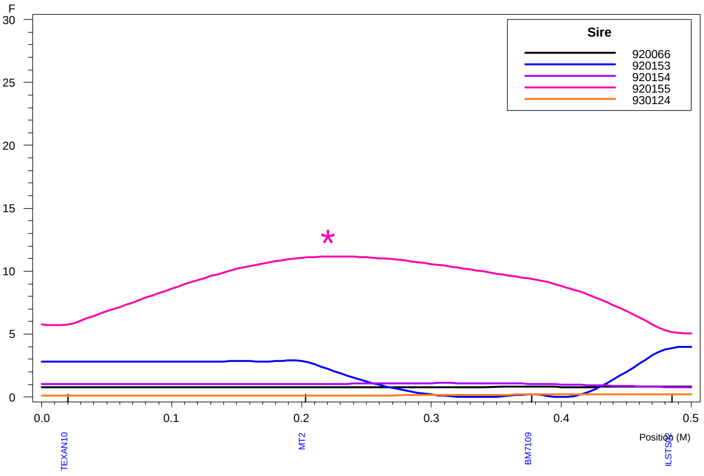

Haley-Knott QTL Analysis: Chromosome 14
ELISA3

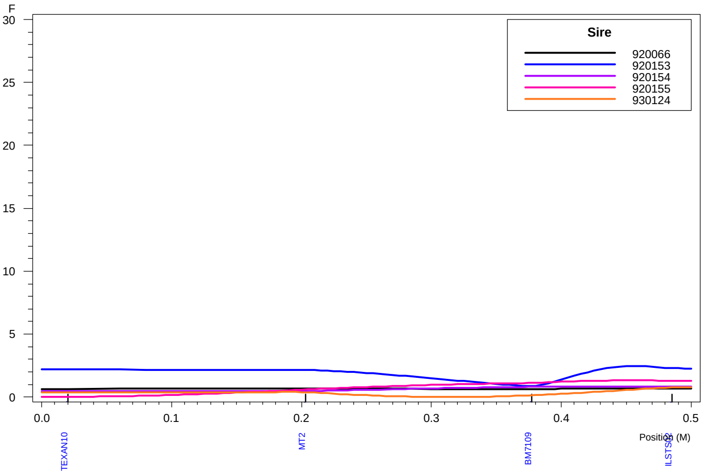

Haley-Knott QTL Analysis: Chromosome 14
ELISA4

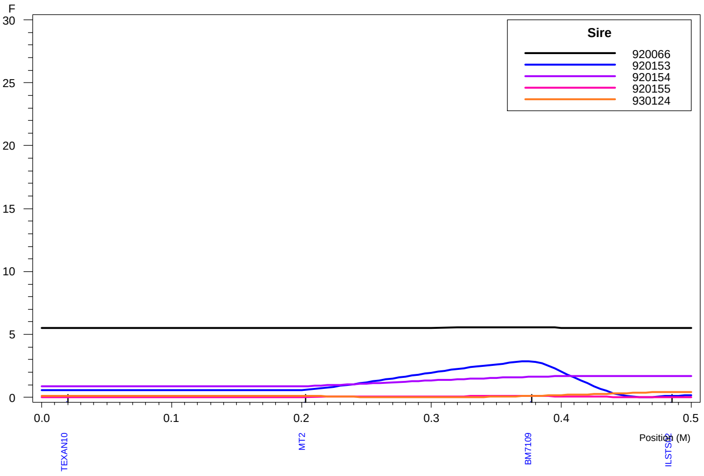

Haley-Knott QTL Analysis: Chromosome 14
ELISA5

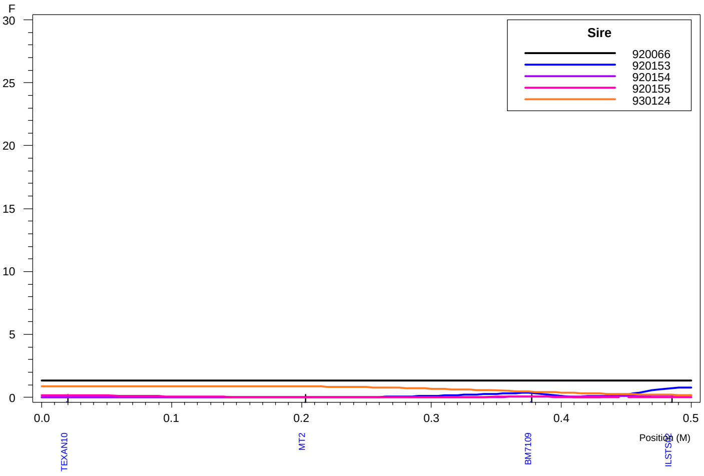

Haley-Knott QTL Analysis: Chromosome 14
LIGE

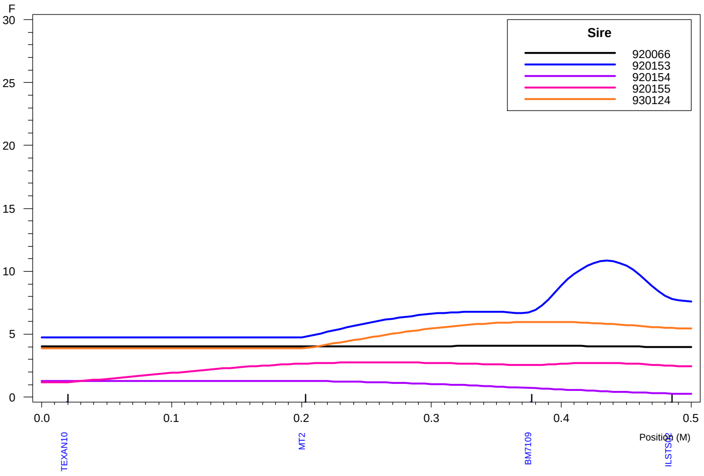

Haley-Knott QTL Analysis: Chromosome 14
WTFEC2

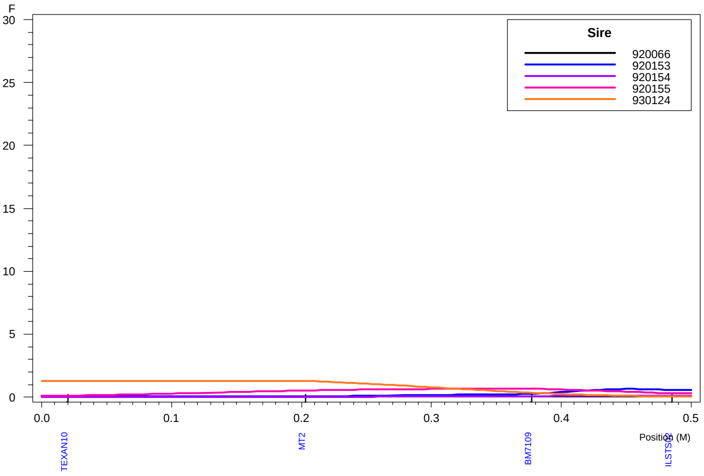

Supplement: Additional File 19 — Chr 14. Haley Knott linkage analysis of sheep chromosome 14. [file 1471-2164-7-178-S19.pdf]
