# Supplementary material for: Discovery of quantitative trait loci for resistance to parasitic nematode infection in sheep: I. Analysis of outcross pedigrees
Source: BMC Genomics. 2006 Jul 18;7:178. doi: 10.1186/1471-2164-7-178 (PMC1574317; doi:10.1186/1471-2164-7-178)

Linkage Analysis in the Parasite Outcross Flock: Chromosome 15

Information Content: Chromosome 15

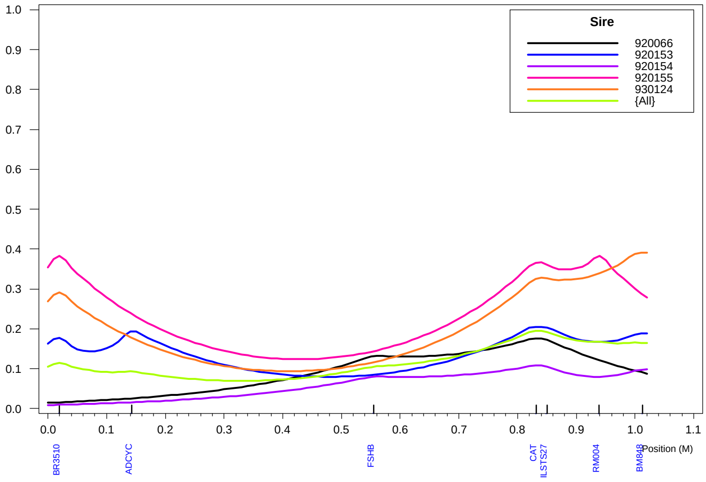

Haley-Knott QTL Analysis: Chromosome 15

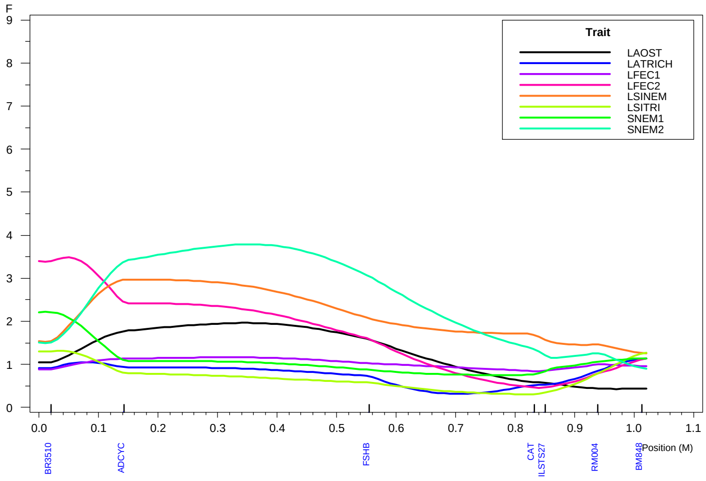

Haley-Knott QTL Analysis: Chromosome 15

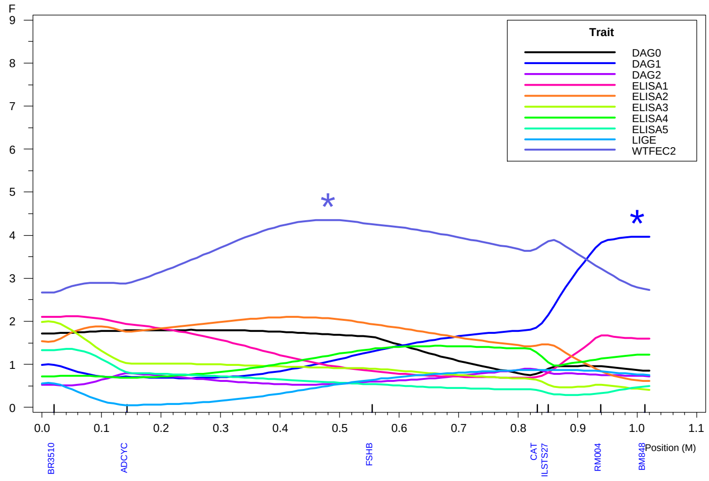

Haley-Knott QTL Analysis: Chromosome 15
LFEC1

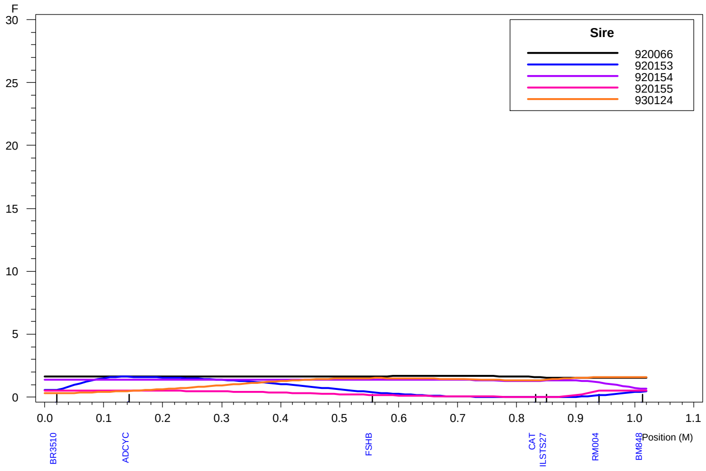

Haley-Knott QTL Analysis: Chromosome 15
LFEC2

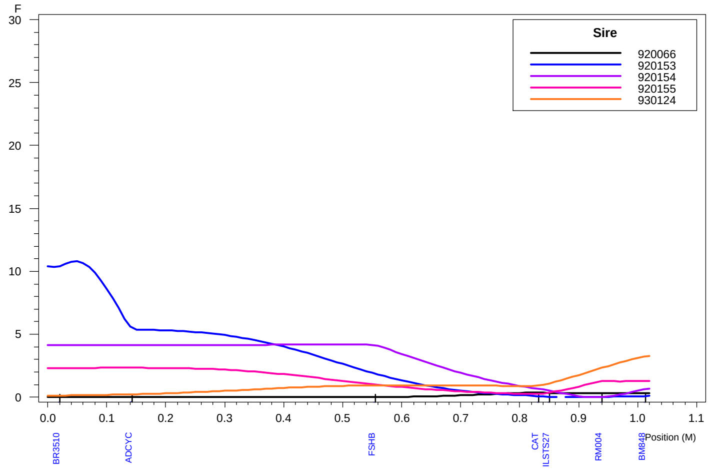

Haley-Knott QTL Analysis: Chromosome 15
SNEM1

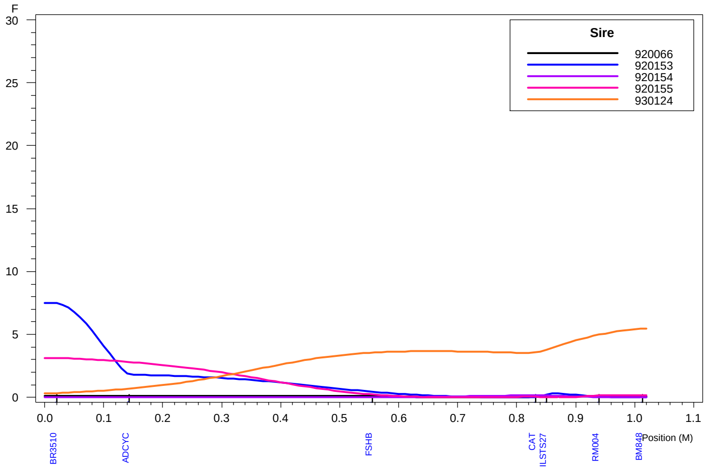

Haley-Knott QTL Analysis: Chromosome 15
SNEM2

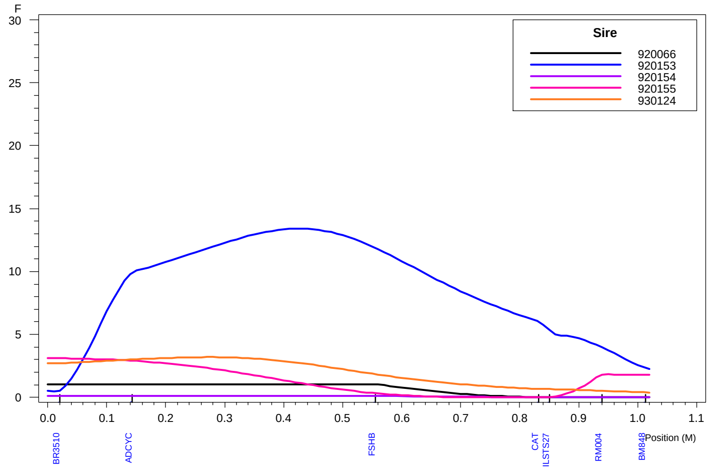

Haley-Knott QTL Analysis: Chromosome 15
LSINEM

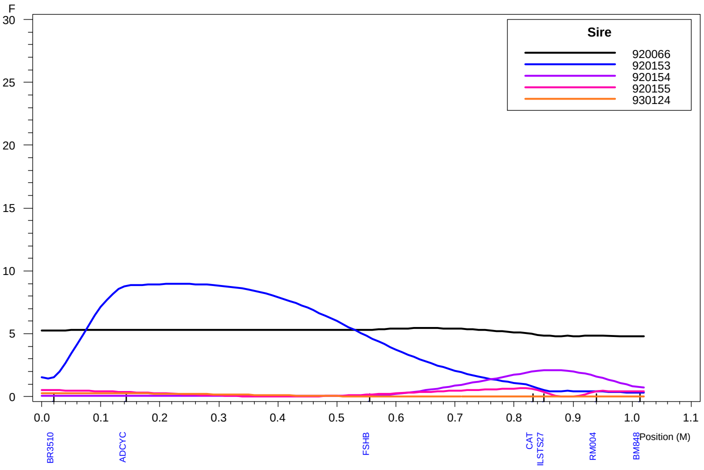

Haley-Knott QTL Analysis: Chromosome 15
LSITRI

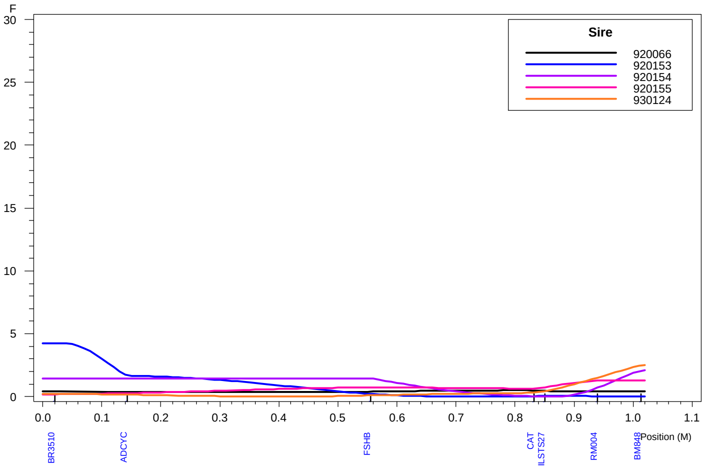

Haley-Knott QTL Analysis: Chromosome 15
LAOST

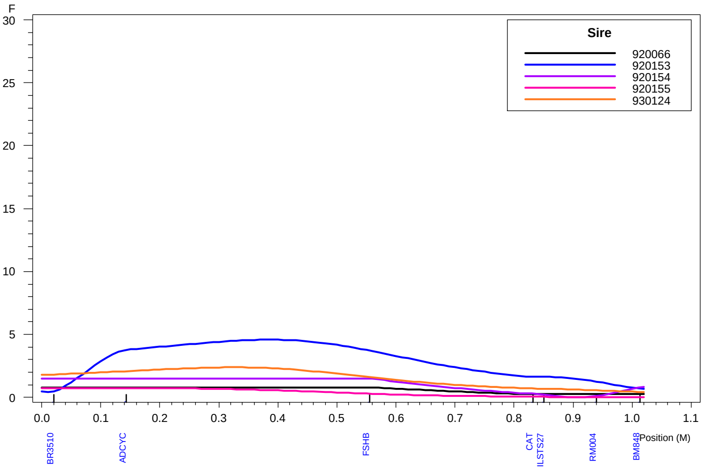

Haley-Knott QTL Analysis: Chromosome 15
LATRICH

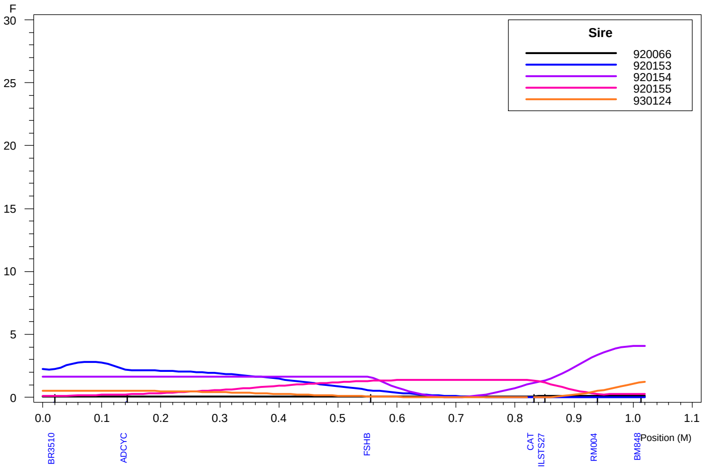

Haley-Knott QTL Analysis: Chromosome 15
DAG0

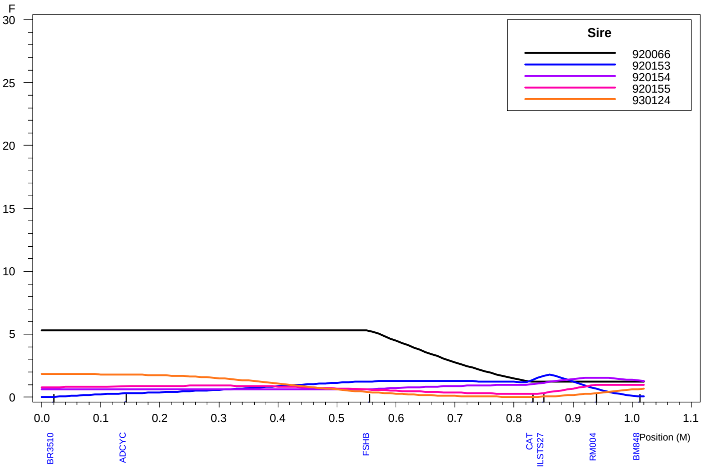

Haley-Knott QTL Analysis: Chromosome 15
DAG1

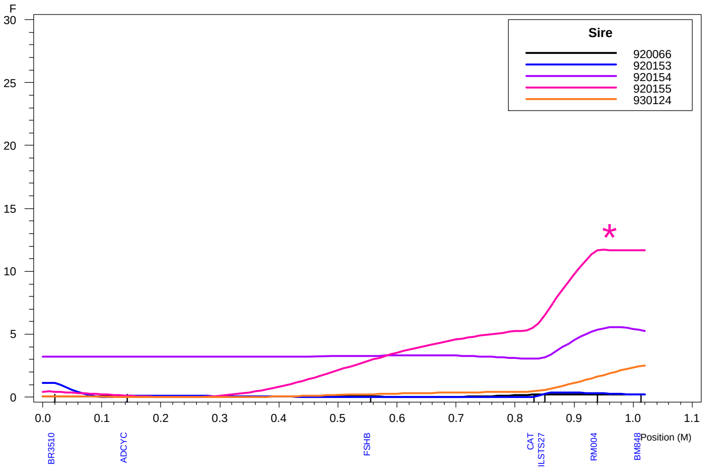

Haley-Knott QTL Analysis: Chromosome 15
DAG2

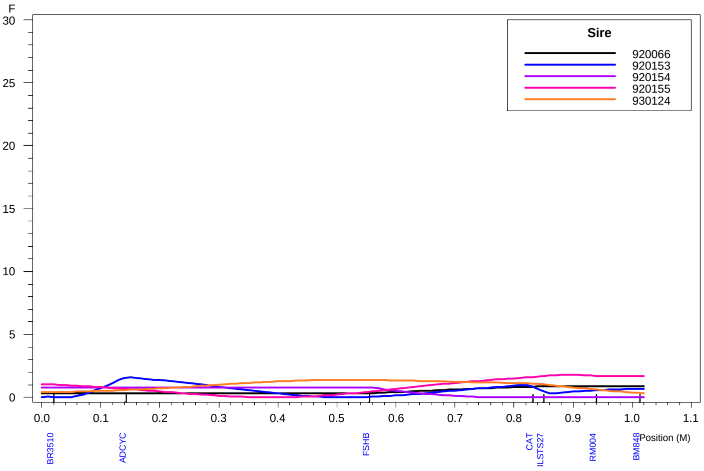

Haley-Knott QTL Analysis: Chromosome 15
ELISA1

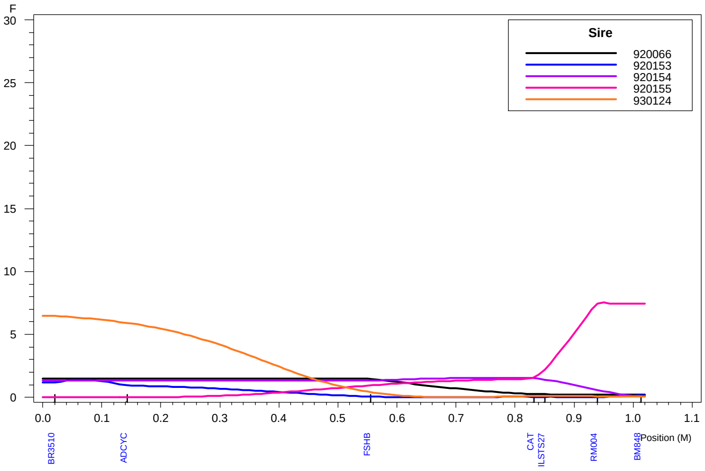

Haley-Knott QTL Analysis: Chromosome 15
ELISA2

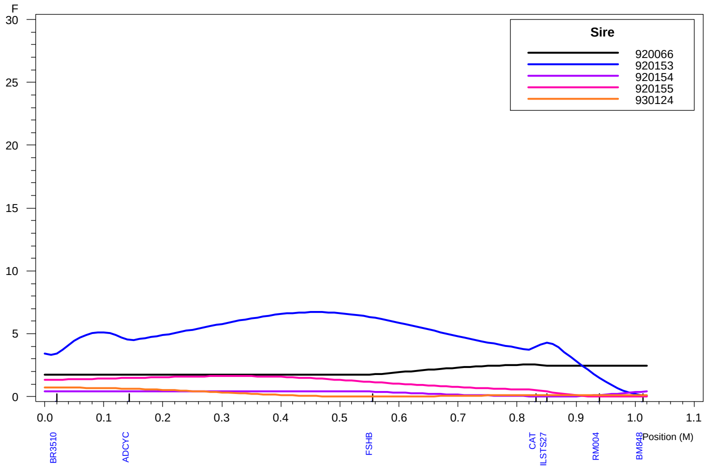

Haley-Knott QTL Analysis: Chromosome 15
ELISA3

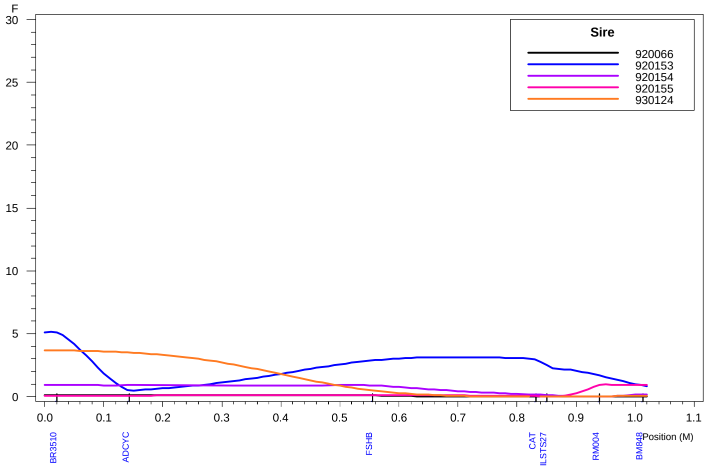

Haley-Knott QTL Analysis: Chromosome 15
ELISA4

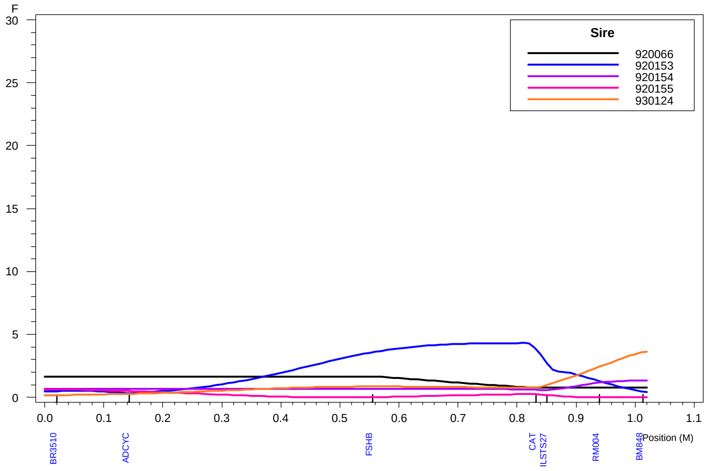

Haley-Knott QTL Analysis: Chromosome 15
ELISA5

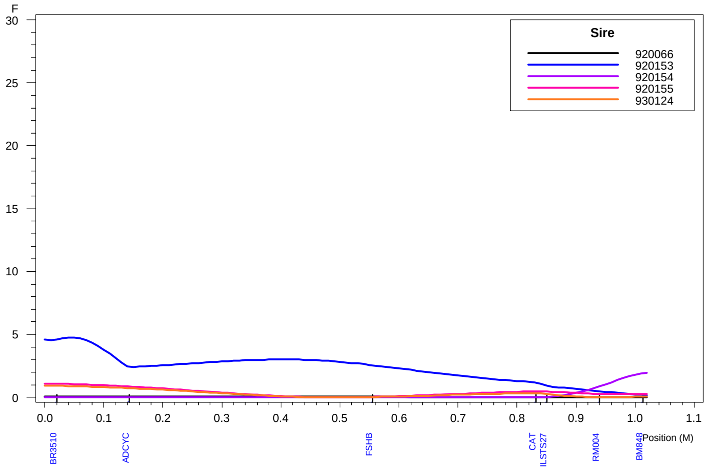

Haley-Knott QTL Analysis: Chromosome 15
LIGE

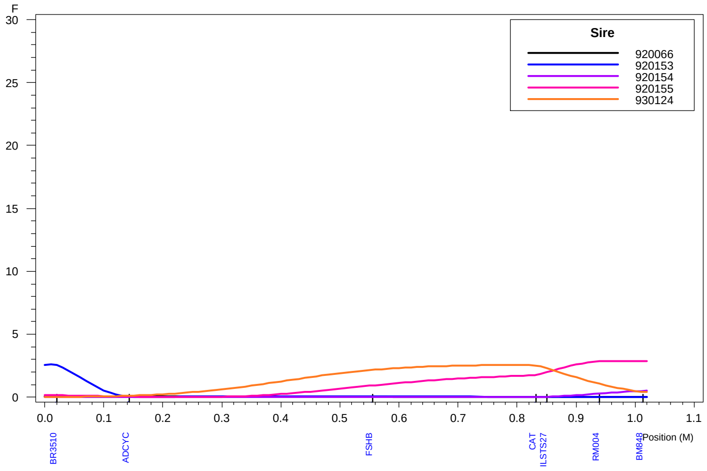

Haley-Knott QTL Analysis: Chromosome 15
WTFEC2

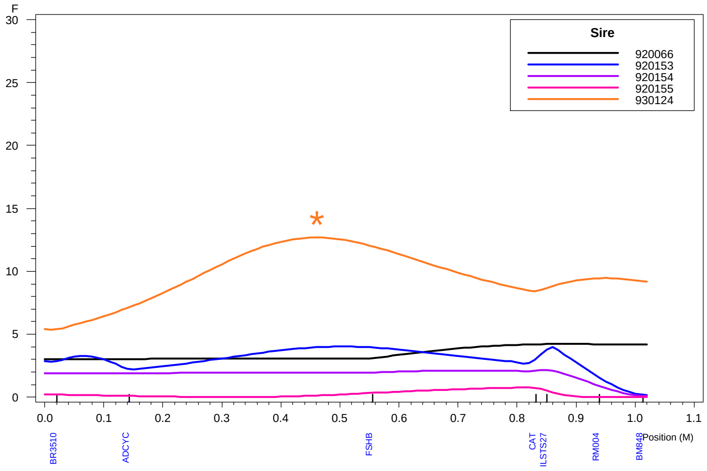

Supplement: Additional File 20 — Chr 15. Haley Knott linkage analysis of sheep chromosome 15. [file 1471-2164-7-178-S20.pdf]
