# Supplementary material for: Discovery of quantitative trait loci for resistance to parasitic nematode infection in sheep: I. Analysis of outcross pedigrees
Source: BMC Genomics. 2006 Jul 18;7:178. doi: 10.1186/1471-2164-7-178 (PMC1574317; doi:10.1186/1471-2164-7-178)

Linkage Analysis in the Parasite Outcross Flock: Chromosome 16

Information Content: Chromosome 16

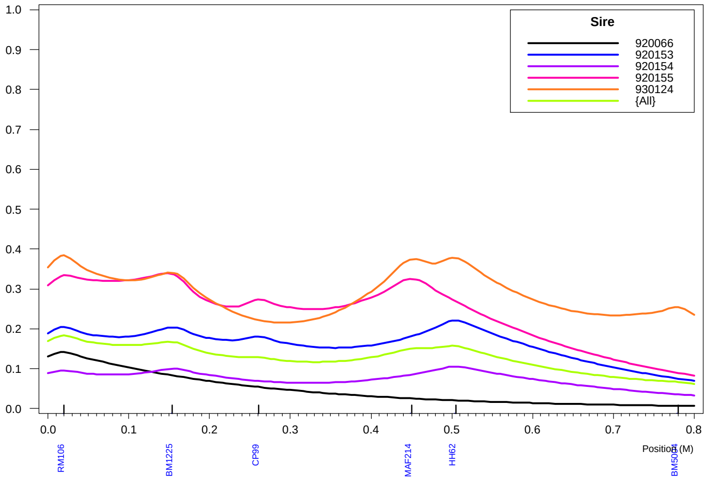

Haley-Knott QTL Analysis: Chromosome 16

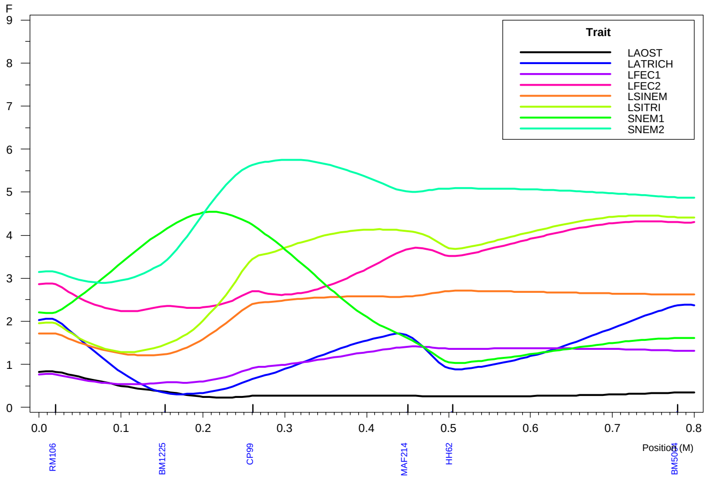

Haley-Knott QTL Analysis: Chromosome 16

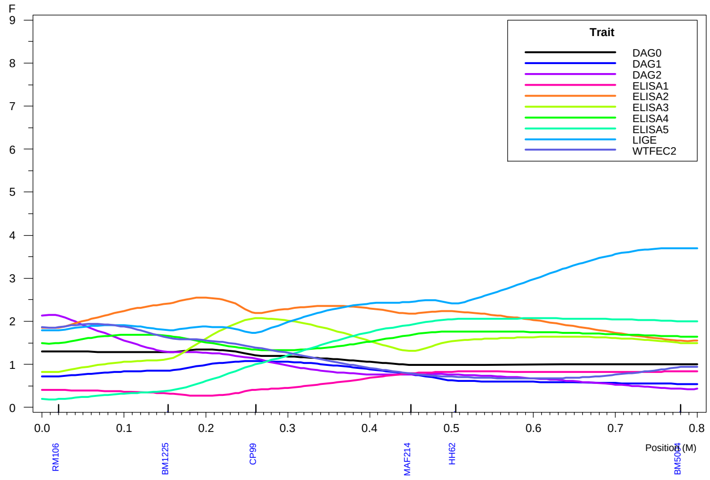

Haley-Knott QTL Analysis: Chromosome 16
LFEC1

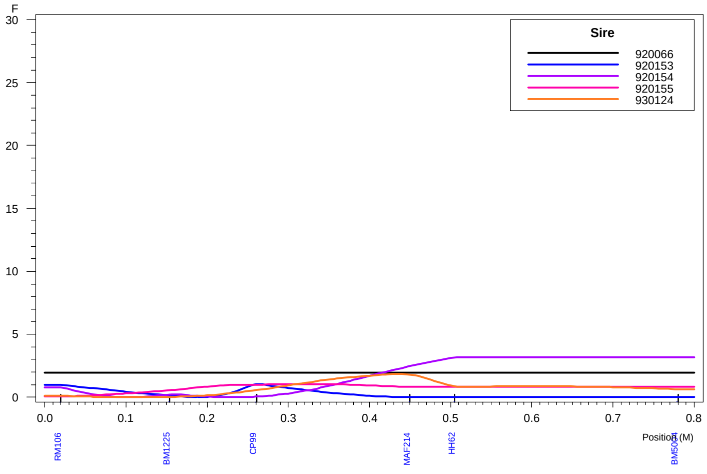

Haley-Knott QTL Analysis: Chromosome 16
LFEC2

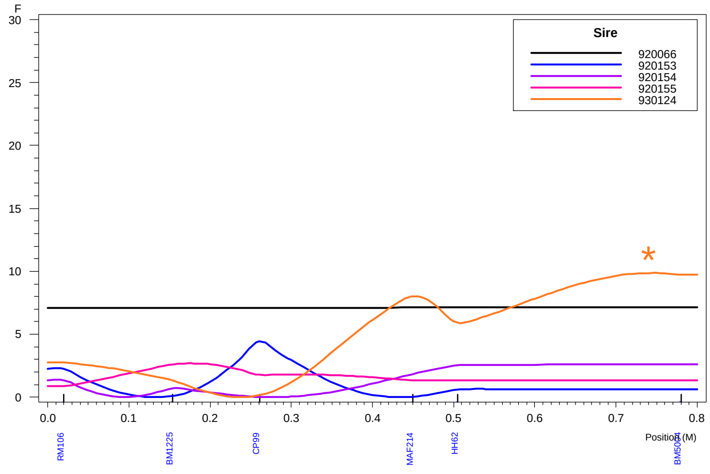

Haley-Knott QTL Analysis: Chromosome 16
SNEM1

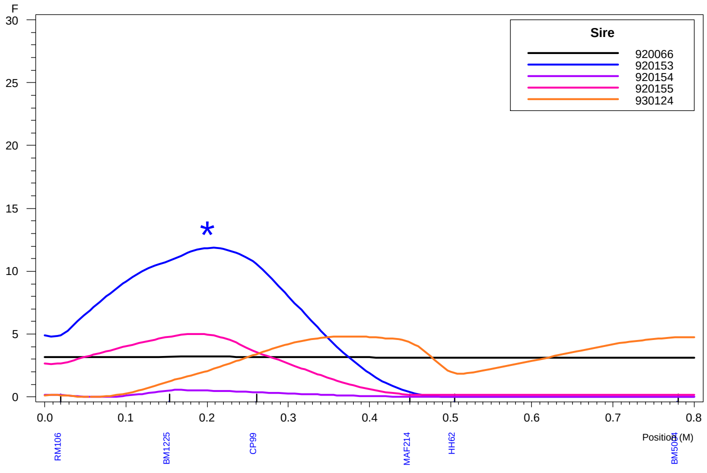

Haley-Knott QTL Analysis: Chromosome 16
SNEM2

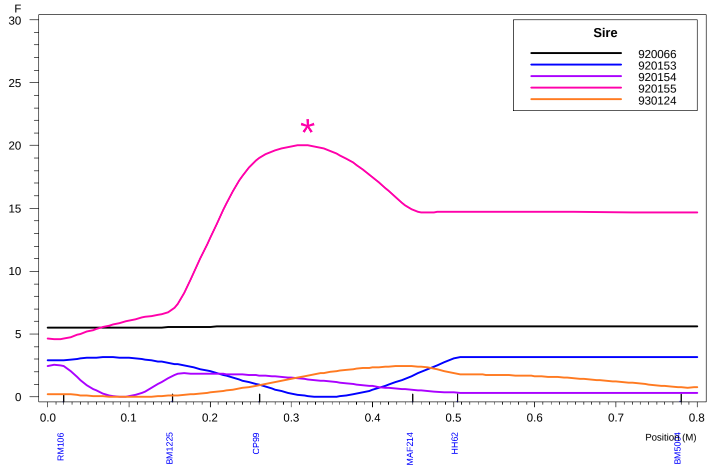

Haley-Knott QTL Analysis: Chromosome 16
LSINEM

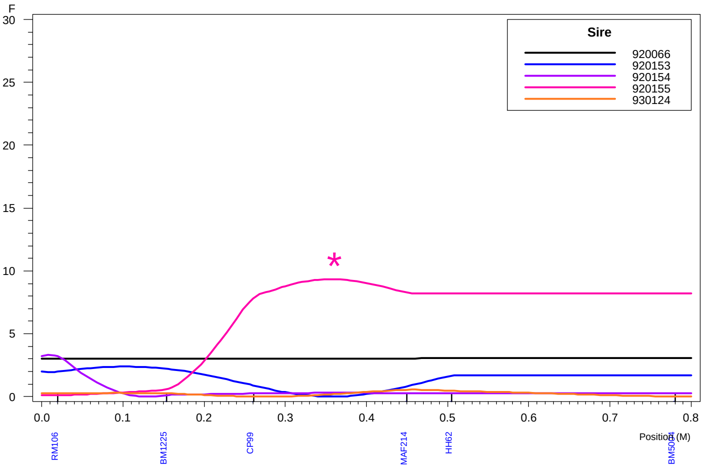

Haley-Knott QTL Analysis: Chromosome 16
LSITRI

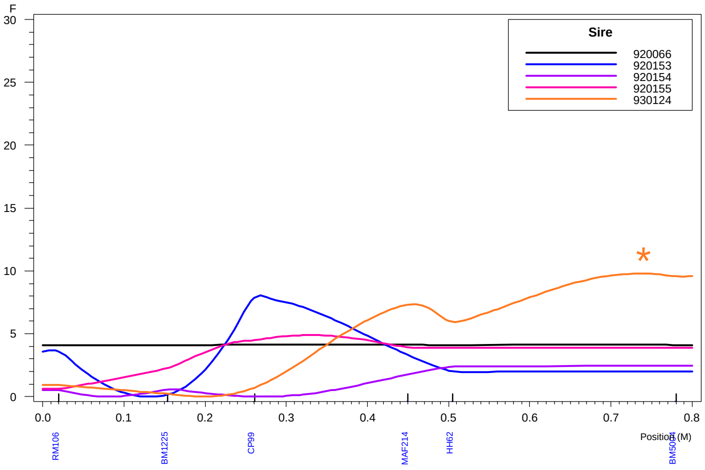

Haley-Knott QTL Analysis: Chromosome 16
LAOST

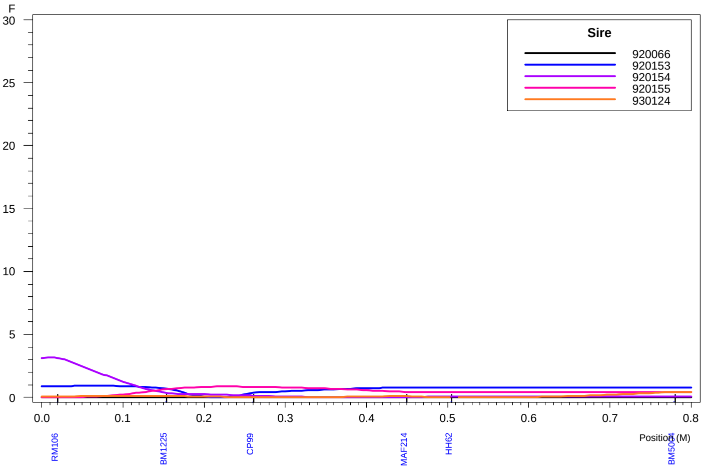

Haley-Knott QTL Analysis: Chromosome 16
LATRICH

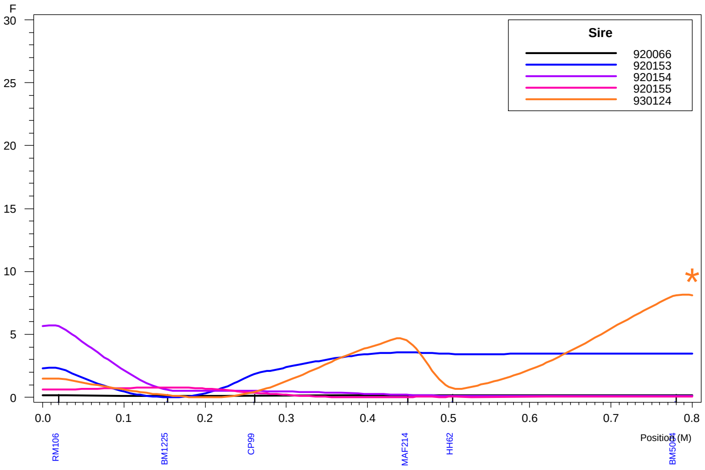

Haley-Knott QTL Analysis: Chromosome 16
DAG0

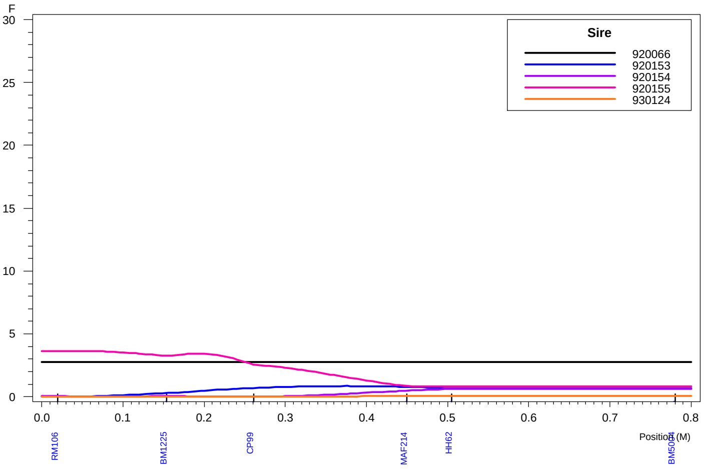

Haley-Knott QTL Analysis: Chromosome 16
DAG1

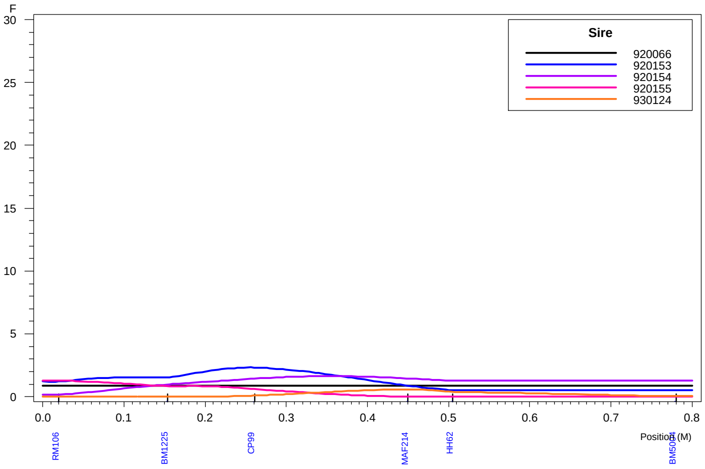

Haley-Knott QTL Analysis: Chromosome 16
DAG2

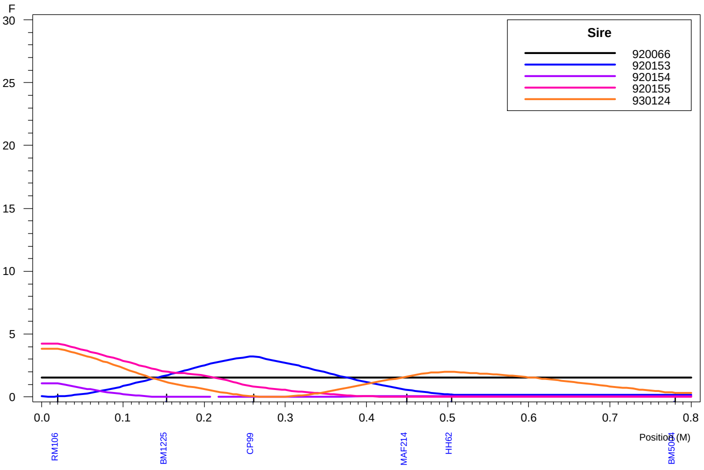

Haley-Knott QTL Analysis: Chromosome 16
ELISA1

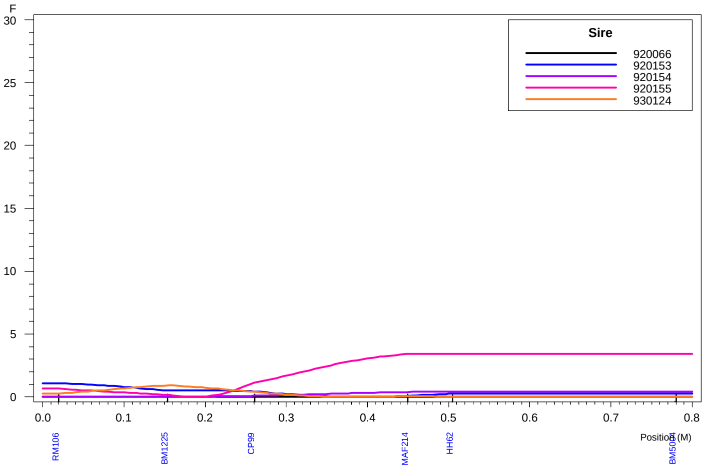

Haley-Knott QTL Analysis: Chromosome 16
ELISA2

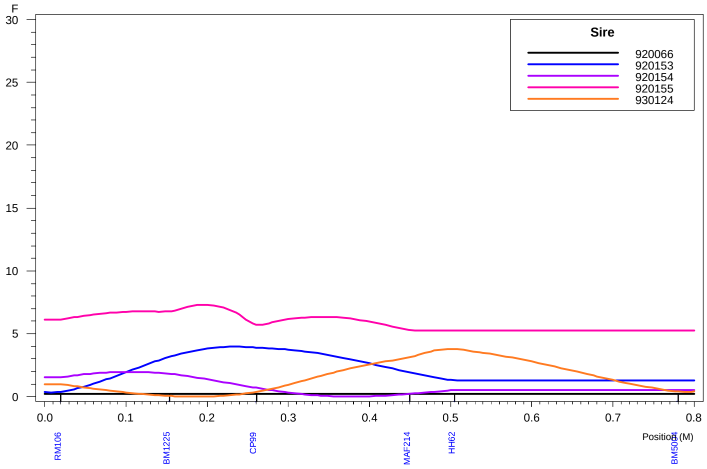

Haley-Knott QTL Analysis: Chromosome 16
ELISA3

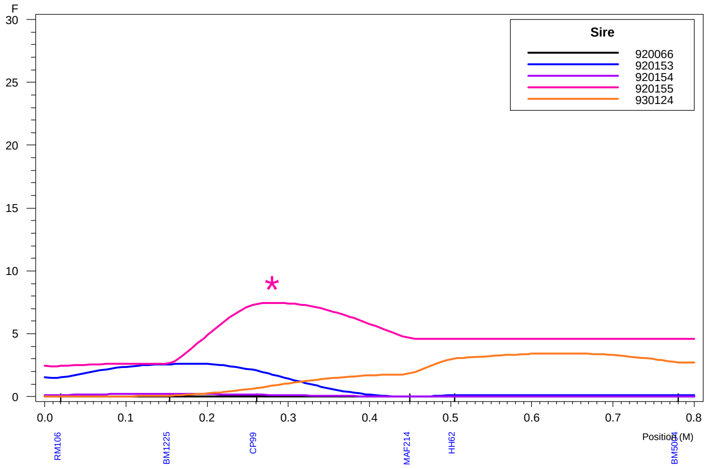

Haley-Knott QTL Analysis: Chromosome 16
ELISA4

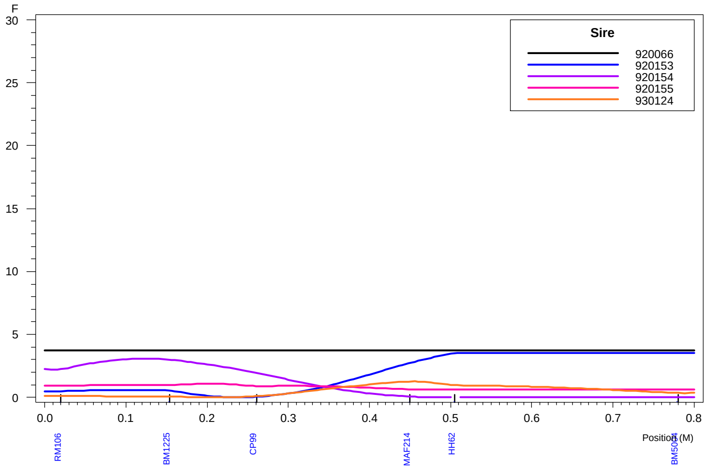

Haley-Knott QTL Analysis: Chromosome 16
ELISA5

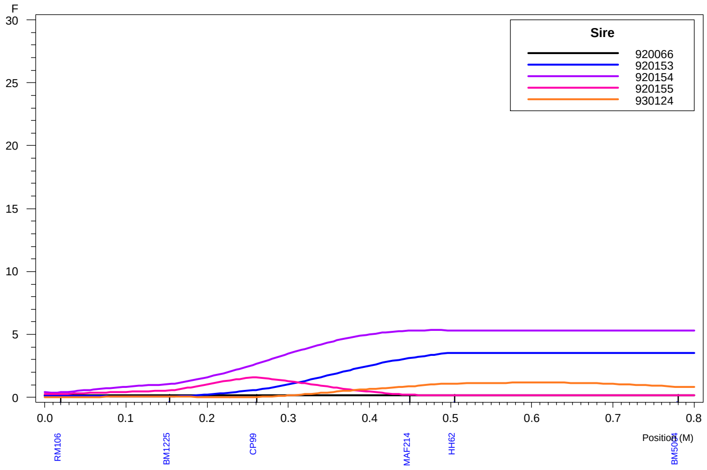

Haley-Knott QTL Analysis: Chromosome 16
LIGE

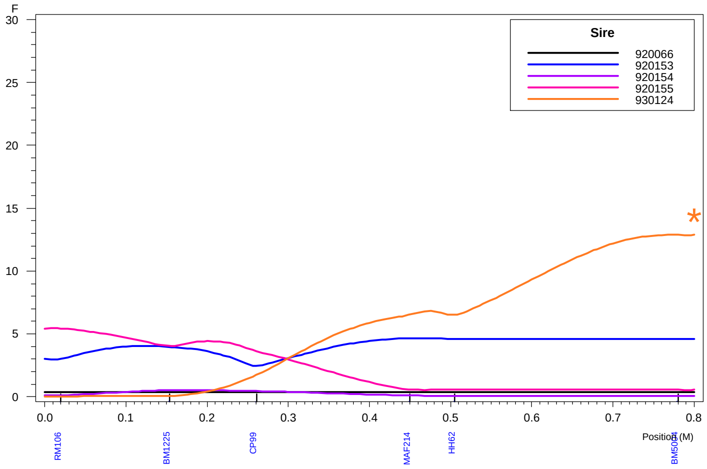

Haley-Knott QTL Analysis: Chromosome 16
WTFEC2

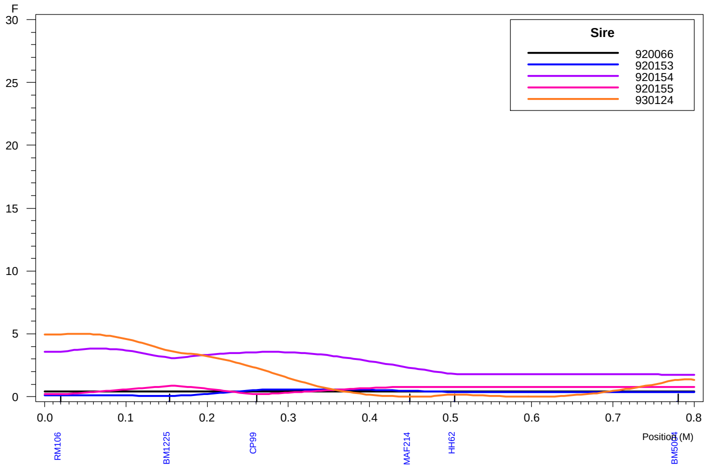

Supplement: Additional File 21 — Chr 16. Haley Knott linkage analysis of sheep chromosome 16. [file 1471-2164-7-178-S21.pdf]
