# Supplementary material for: Discovery of quantitative trait loci for resistance to parasitic nematode infection in sheep: I. Analysis of outcross pedigrees
Source: BMC Genomics. 2006 Jul 18;7:178. doi: 10.1186/1471-2164-7-178 (PMC1574317; doi:10.1186/1471-2164-7-178)
Supplement: Additional File 22 — Chr 17. Haley Knott linkage analysis of sheep chromosome 17. [file 1471-2164-7-178-S22.pdf]

Linkage Analysis in the Parasite Outcross Flock: Chromosome 17

Information Content: Chromosome 17

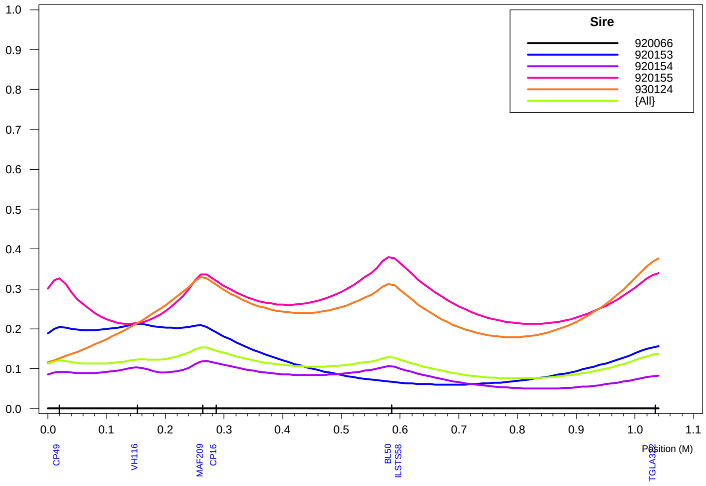

Haley-Knott QTL Analysis: Chromosome 17

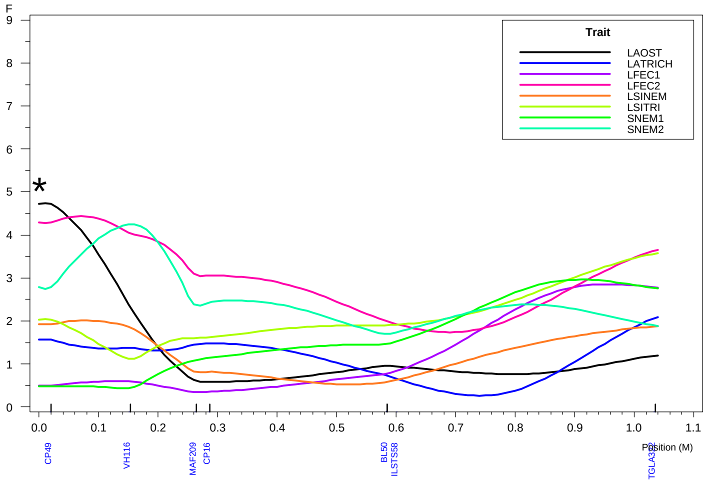

Haley-Knott QTL Analysis: Chromosome 17

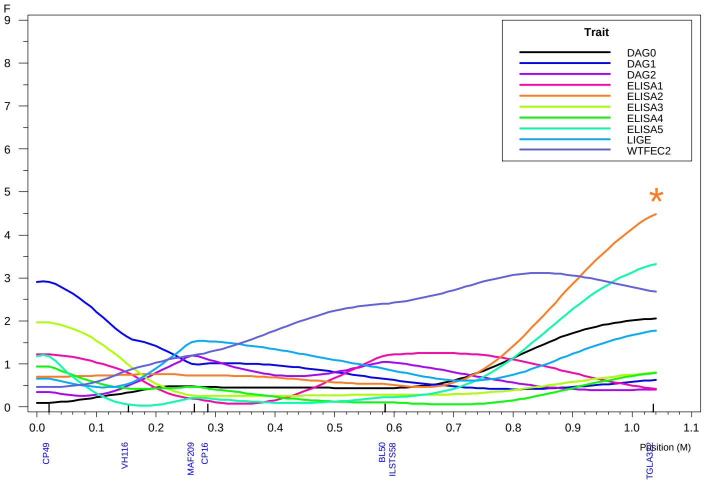

Haley-Knott QTL Analysis: Chromosome 17
LFEC1

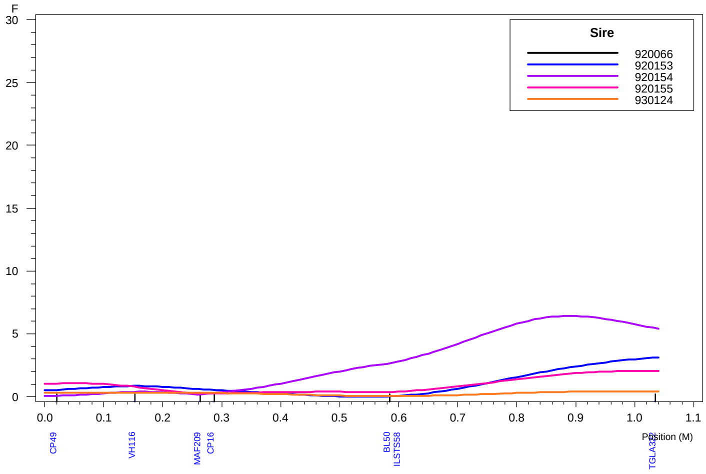

Haley-Knott QTL Analysis: Chromosome 17
LFEC2

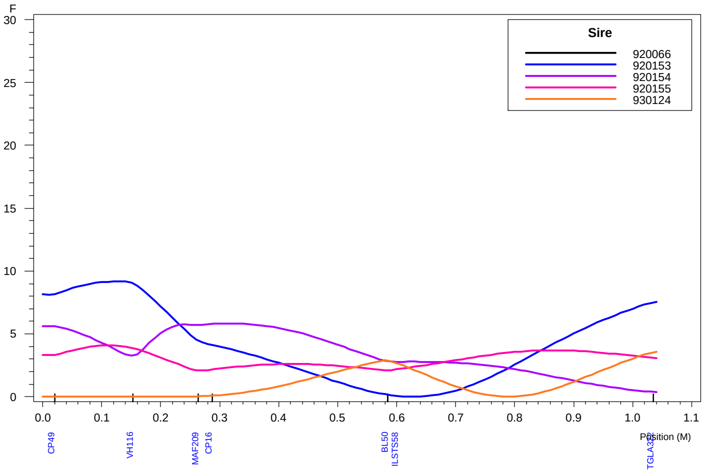

Haley-Knott QTL Analysis: Chromosome 17
SNEM1

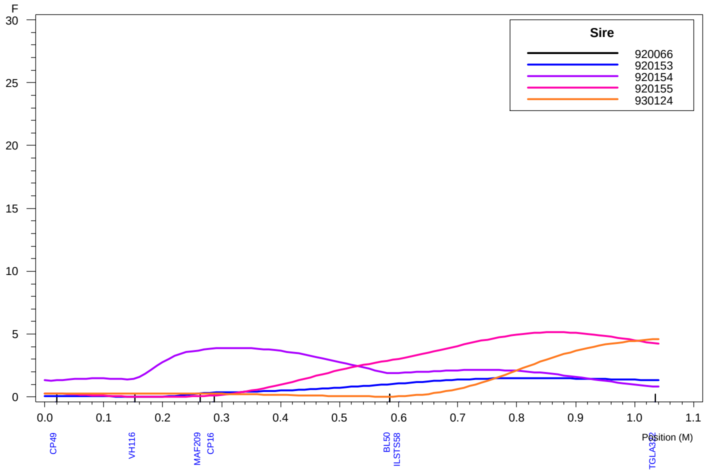

Haley-Knott QTL Analysis: Chromosome 17
SNEM2

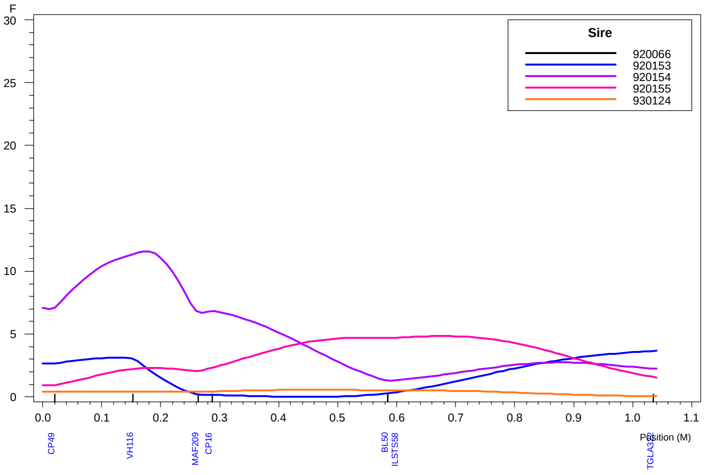

Haley-Knott QTL Analysis: Chromosome 17
LSINEM

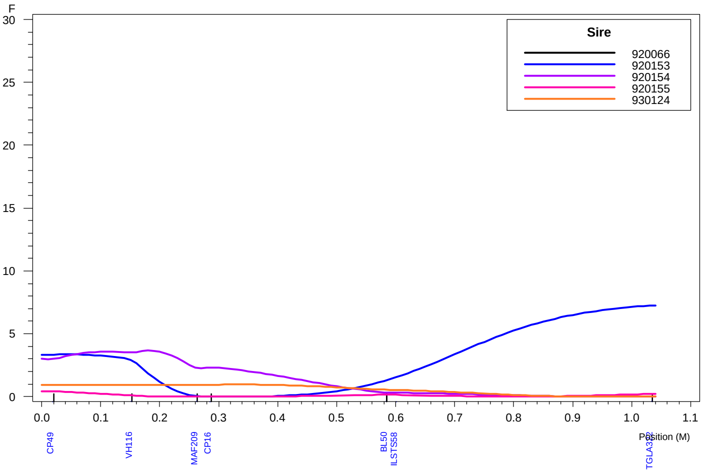

Haley-Knott QTL Analysis: Chromosome 17
LSITRI

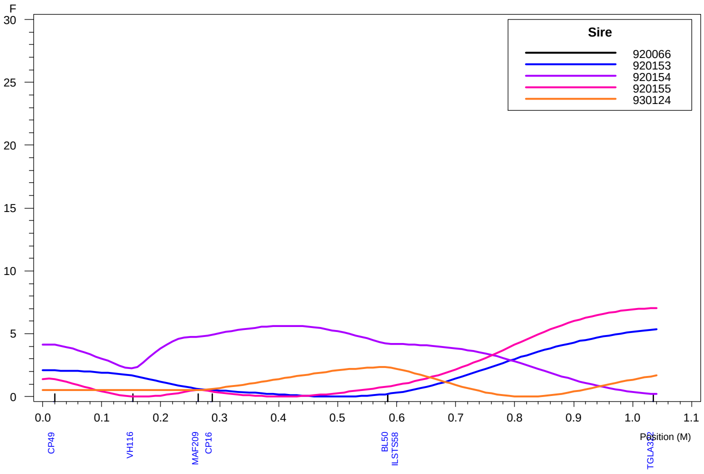

Haley-Knott QTL Analysis: Chromosome 17
LAOST

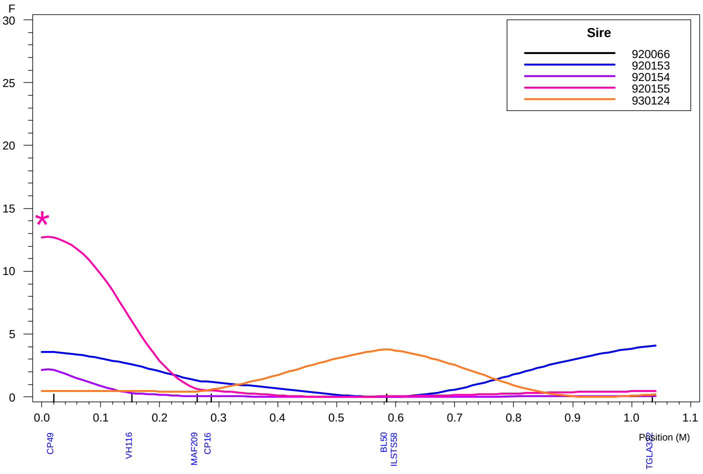

Haley-Knott QTL Analysis: Chromosome 17
LATRICH

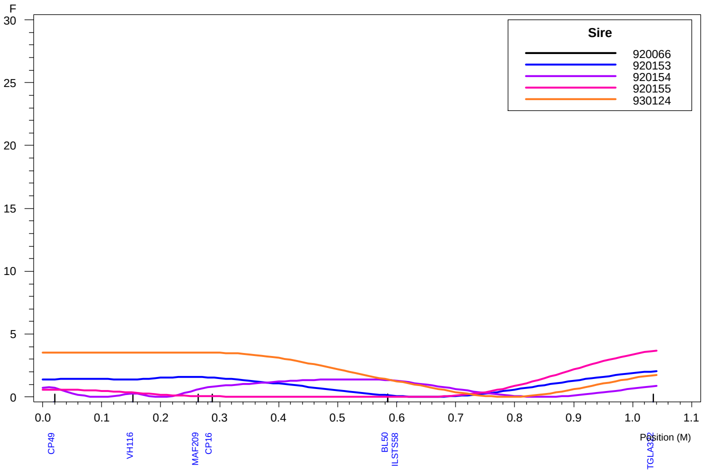

Haley-Knott QTL Analysis: Chromosome 17
DAG0

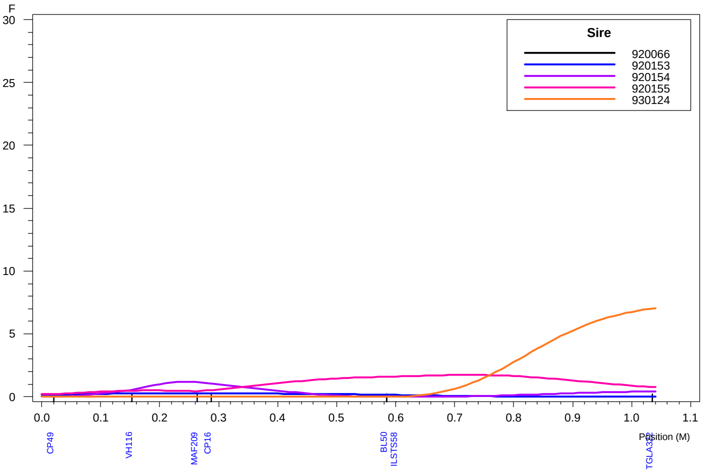

Haley-Knott QTL Analysis: Chromosome 17
DAG1

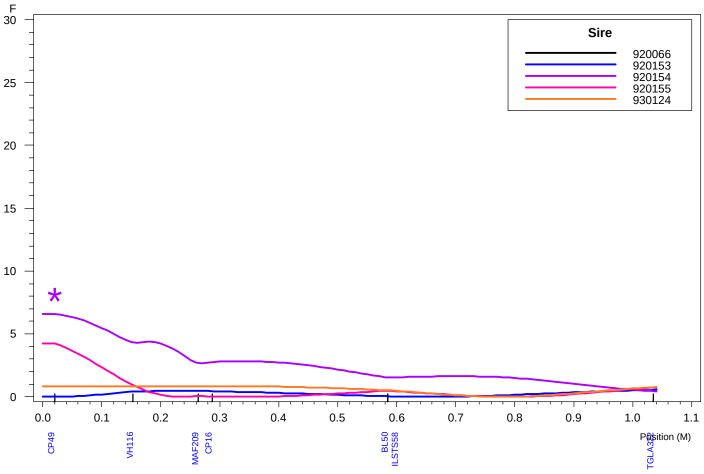

Haley-Knott QTL Analysis: Chromosome 17
DAG2

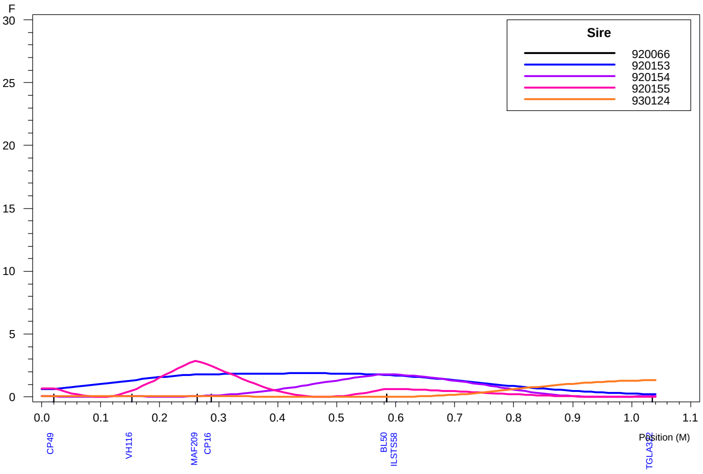

Haley-Knott QTL Analysis: Chromosome 17
ELISA1

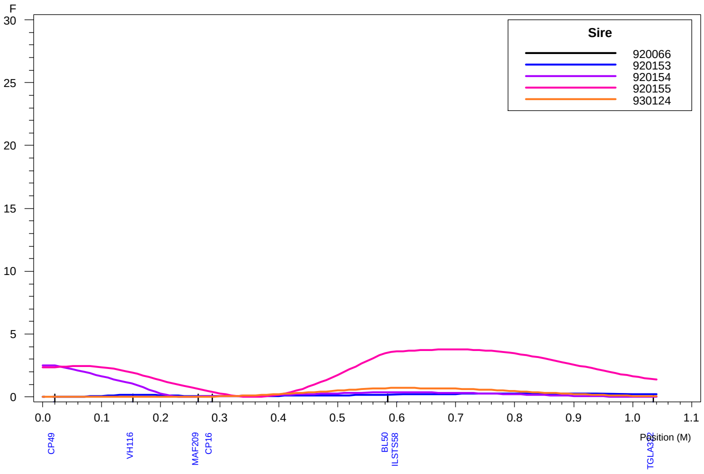

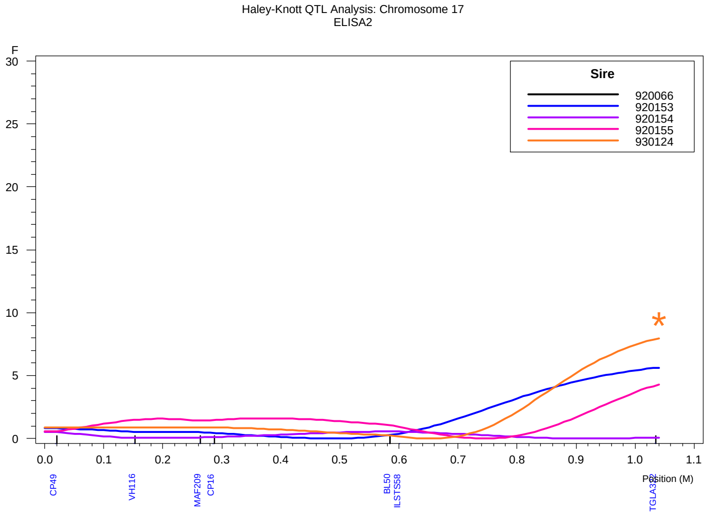
Haley-Knott QTL Analysis: Chromosome 17
ELISA2
F
Sire
920066
920153
920154
920155
930124
Position (M)
CP49
VH116
MAF209
CP16
BL50
ILSTS58
TGLA3

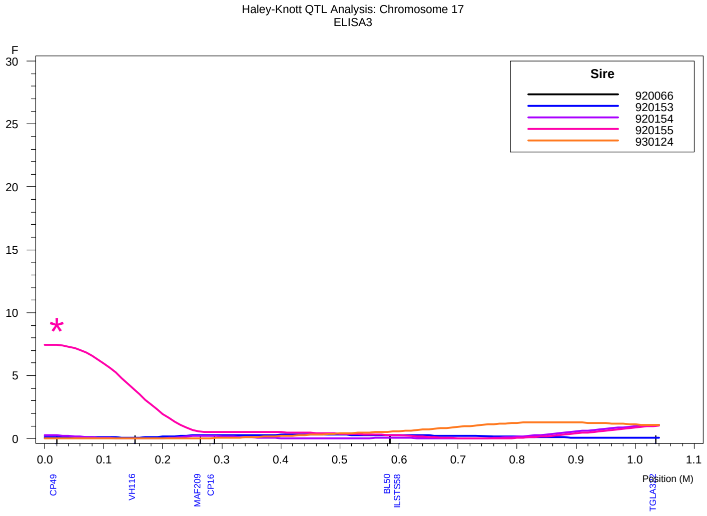
Haley-Knott QTL Analysis: Chromosome 17
ELISA3
F
Sire
920066
920153
920154
920155
930124
Position (M)
CP49
VH116
MAF209
CP16
BL50
ILSTS58
TGLA3

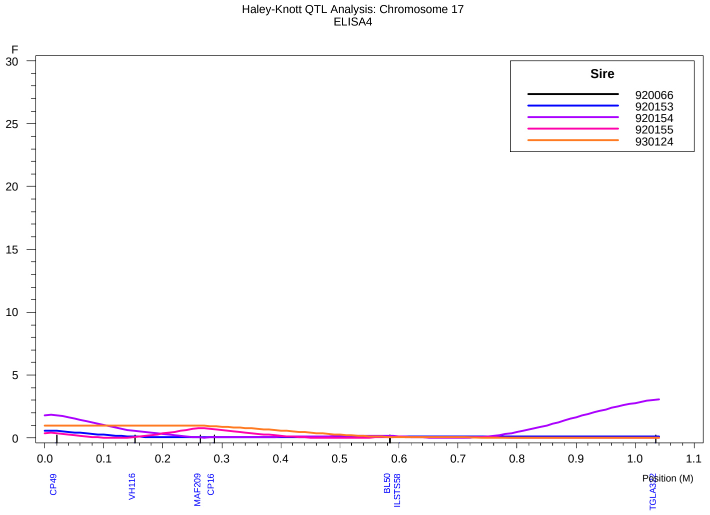
Haley-Knott QTL Analysis: Chromosome 17
ELISA4
F
Sire
920066
920153
920154
920155
930124
Position (M)
CP49
VH116
MAF209
CP16
BL50
ILSTS58
TGLA3

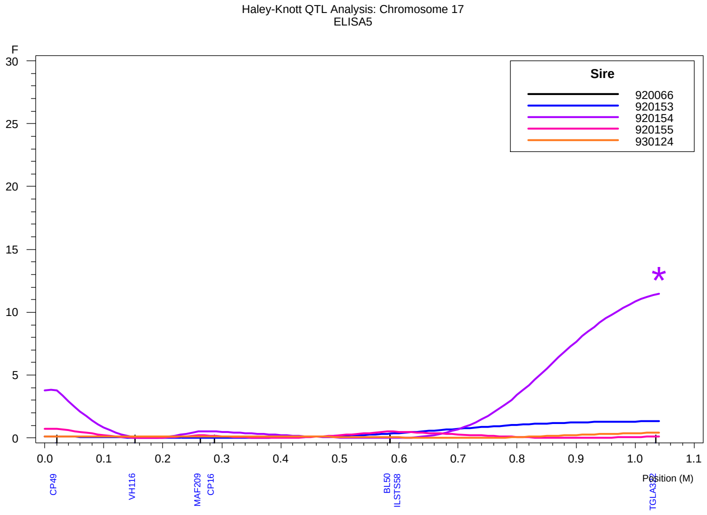
Haley-Knott QTL Analysis: Chromosome 17
ELISA5
F
Sire
920066
920153
920154
920155
930124
Position (M)
CP49
VH116
MAF209
CP16
BL50
ILSTS58
TGLA3

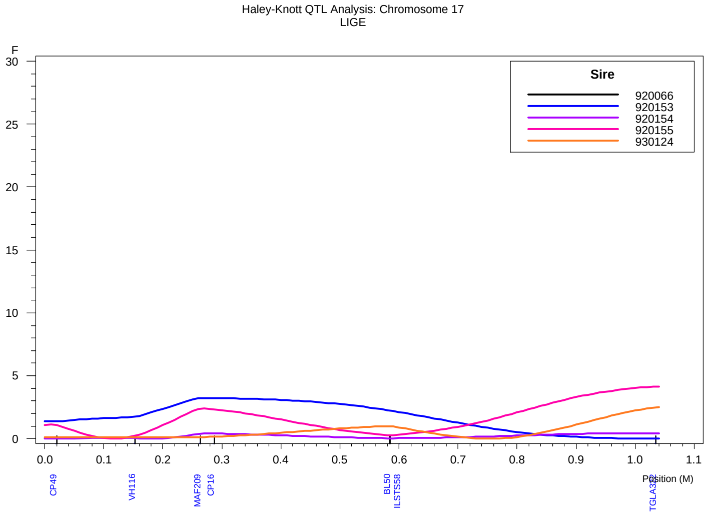
Haley-Knott QTL Analysis: Chromosome 17
LIGE
F
Sire
920066
920153
920154
920155
930124
Position (M)
CP49
VH116
MAF209
CP16
BL50
ILSTS58
TGLA3

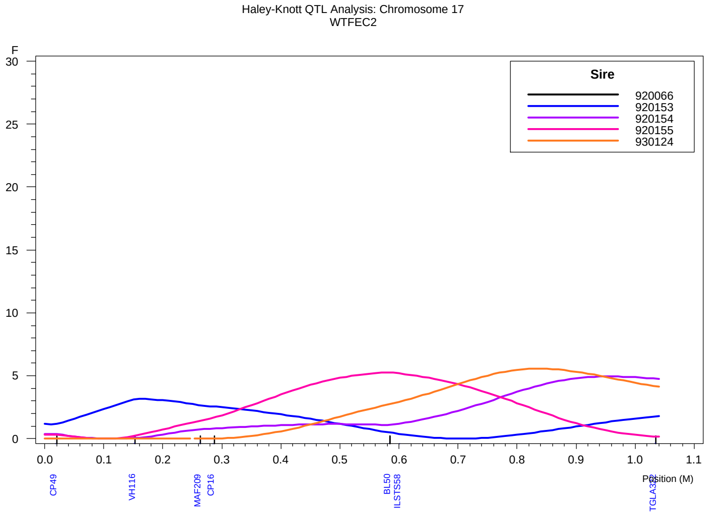
Haley-Knott QTL Analysis: Chromosome 17
WTFEC2
F
Sire
920066
920153
920154
920155
930124
Position (M)
CP49
VH116
MAF209
CP16
BL50
ILSTS58
TGLA3
