# Supplementary material for: Discovery of quantitative trait loci for resistance to parasitic nematode infection in sheep: I. Analysis of outcross pedigrees
Source: BMC Genomics. 2006 Jul 18;7:178. doi: 10.1186/1471-2164-7-178 (PMC1574317; doi:10.1186/1471-2164-7-178)

Linkage Analysis in the Parasite Outcross Flock: Chromosome 18

Information Content: Chromosome 18

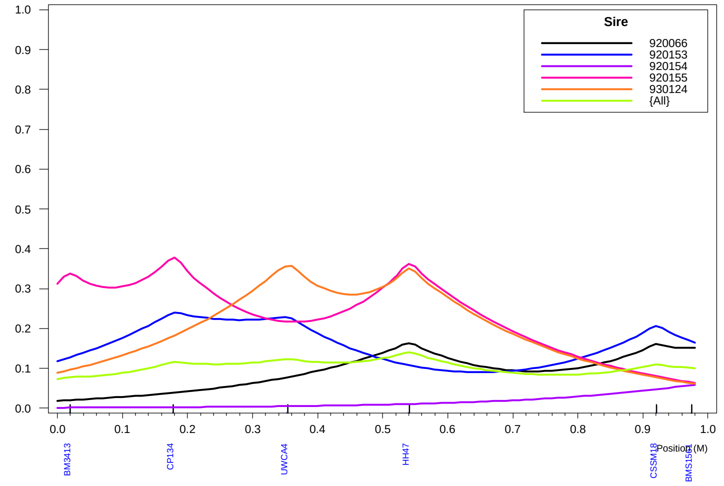

Haley-Knott QTL Analysis: Chromosome 18

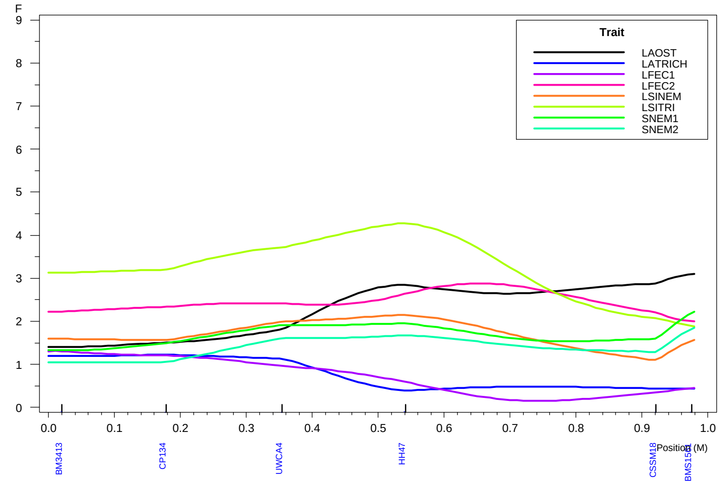

Haley-Knott QTL Analysis: Chromosome 18

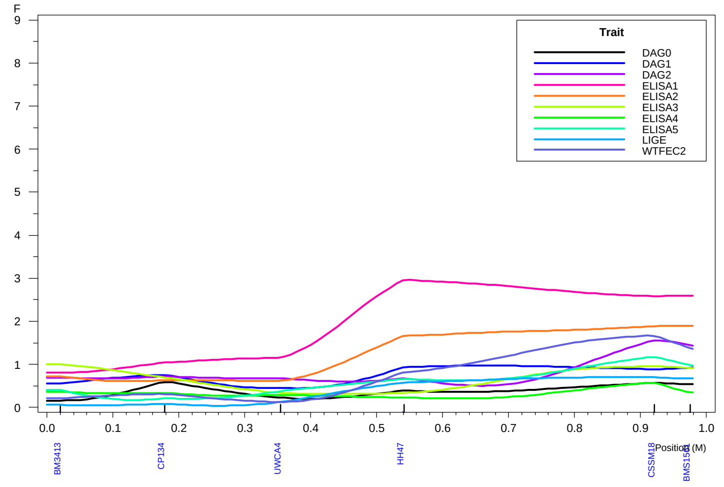

Haley-Knott QTL Analysis: Chromosome 18
LFEC1

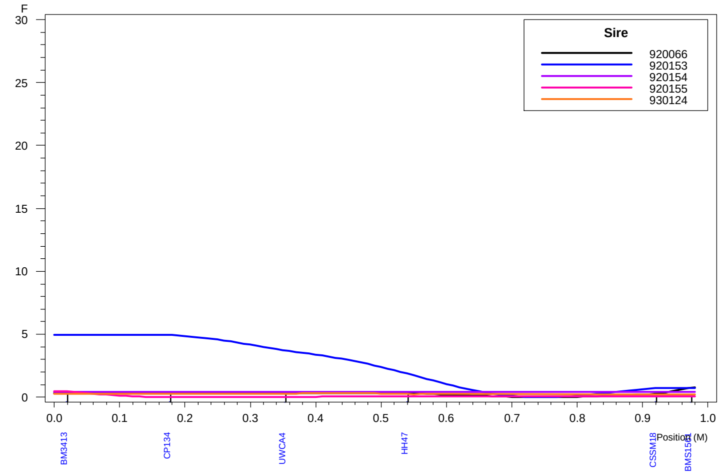

Haley-Knott QTL Analysis: Chromosome 18
LFEC2

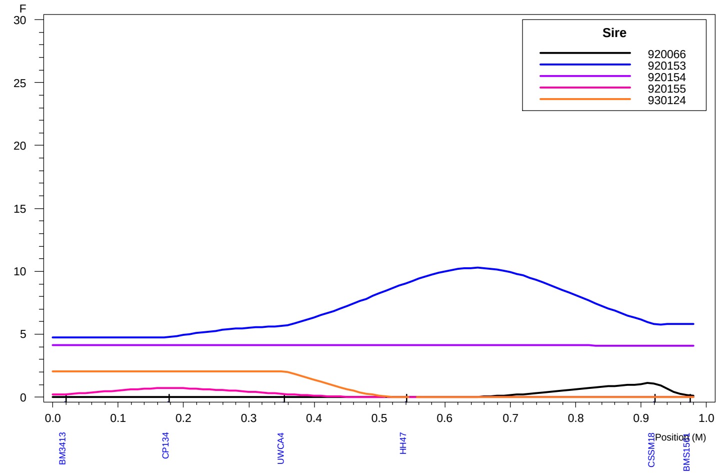

Haley-Knott QTL Analysis: Chromosome 18
SNEM1

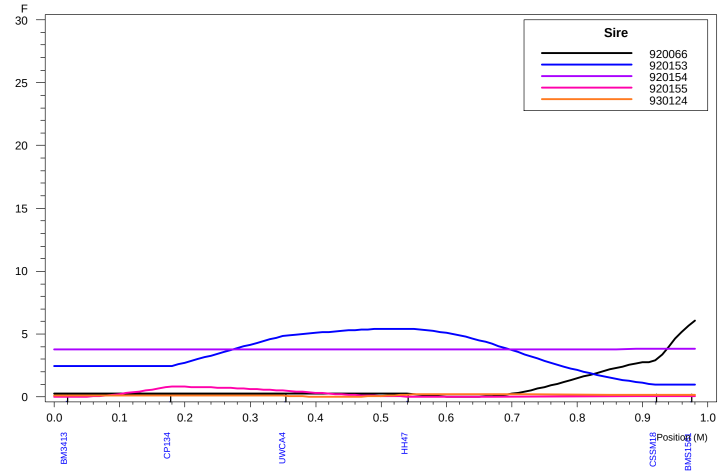

Haley-Knott QTL Analysis: Chromosome 18
SNEM2

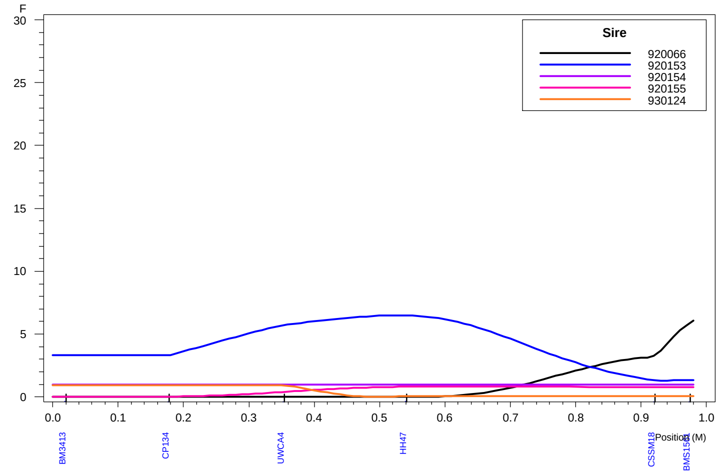

Haley-Knott QTL Analysis: Chromosome 18
LSINEM

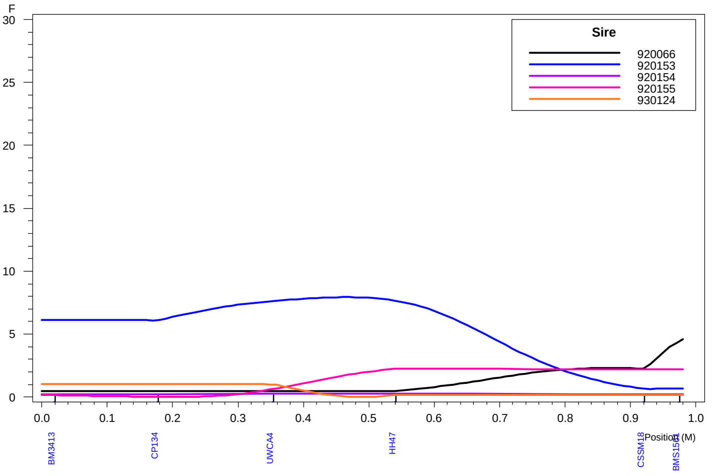

Haley-Knott QTL Analysis: Chromosome 18
LSITRI

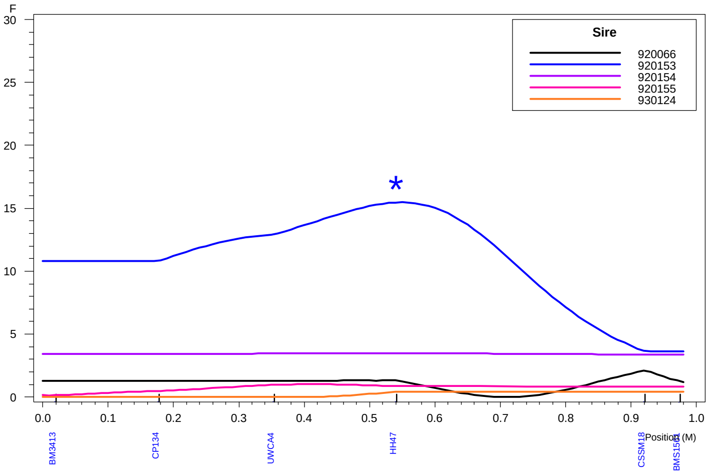

Haley-Knott QTL Analysis: Chromosome 18
LAOST

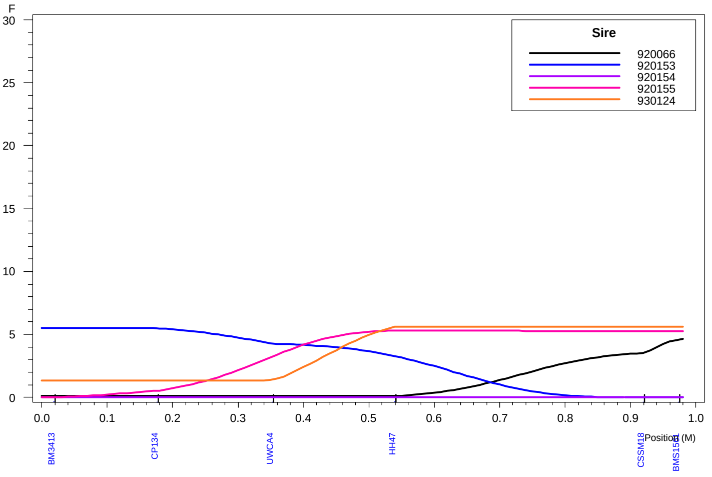

Haley-Knott QTL Analysis: Chromosome 18
LATRICH

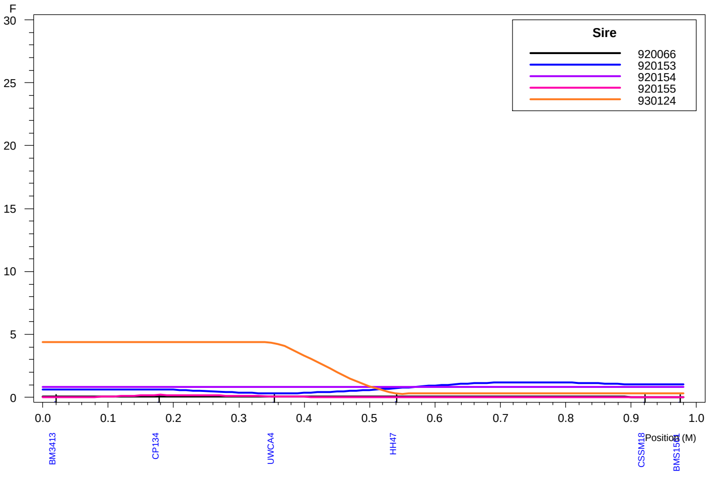

Haley-Knott QTL Analysis: Chromosome 18
DAG0

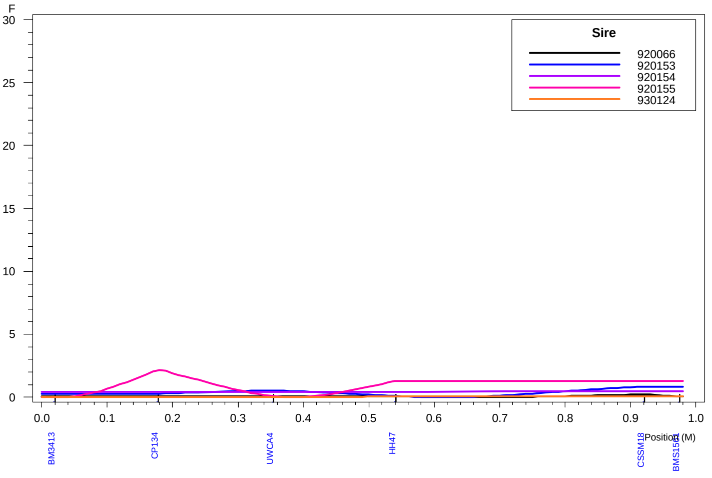

Haley-Knott QTL Analysis: Chromosome 18
DAG1

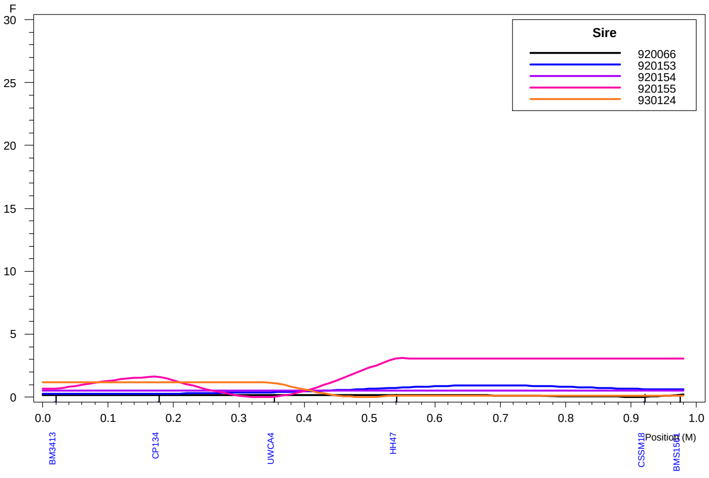

Haley-Knott QTL Analysis: Chromosome 18
DAG2

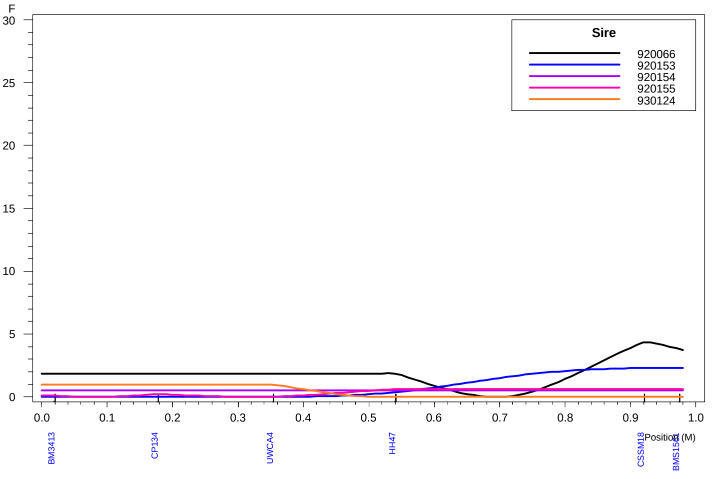

Haley-Knott QTL Analysis: Chromosome 18
ELISA1

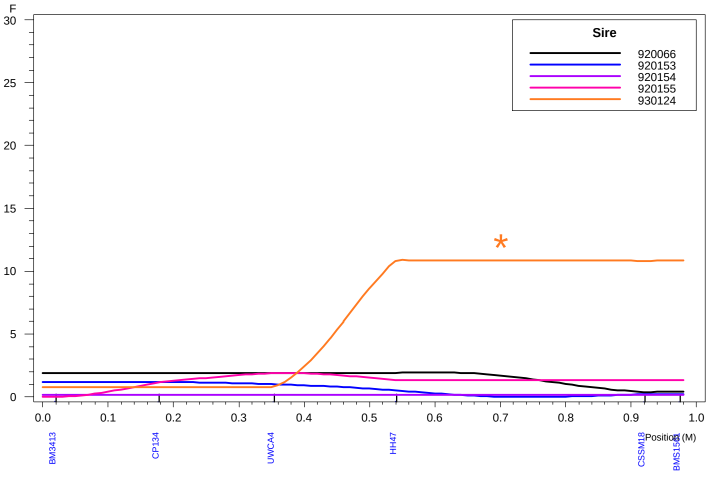

Haley-Knott QTL Analysis: Chromosome 18
ELISA2

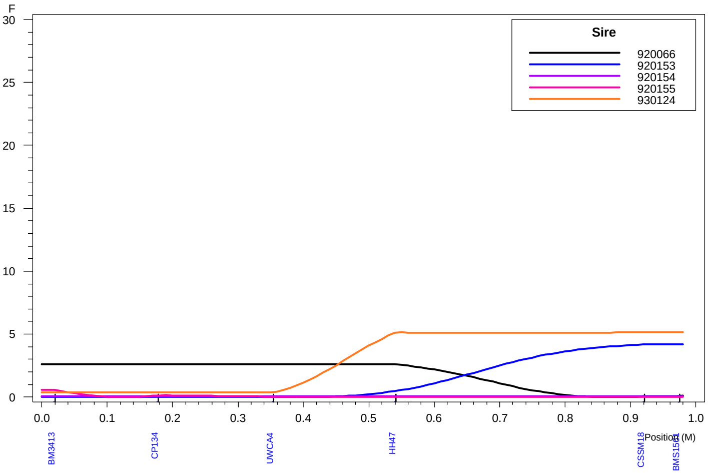

Haley-Knott QTL Analysis: Chromosome 18
ELISA3

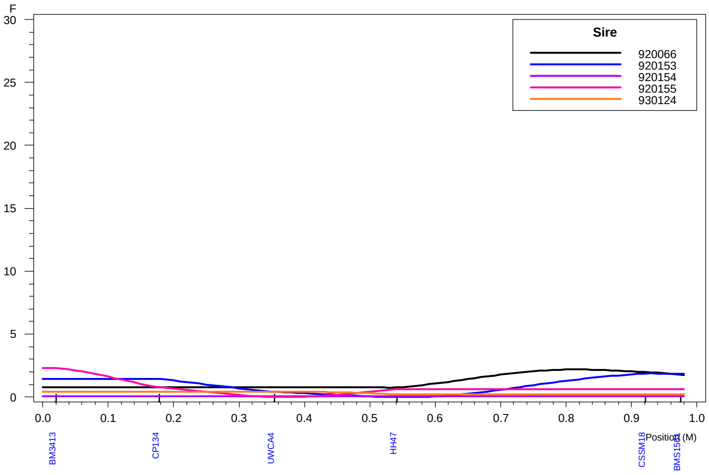

Haley-Knott QTL Analysis: Chromosome 18
ELISA4

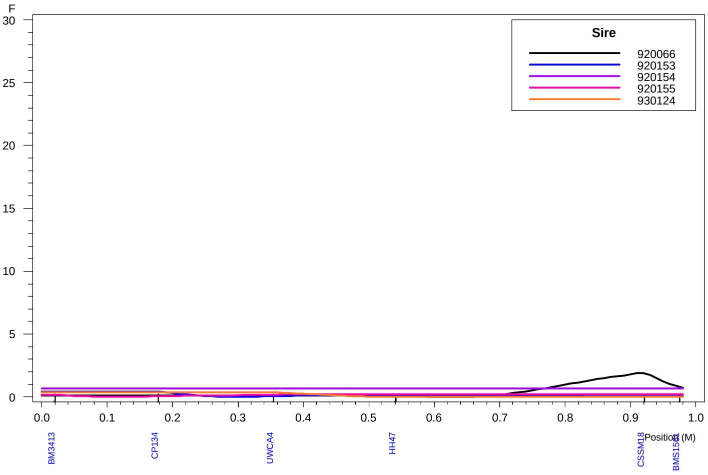

Haley-Knott QTL Analysis: Chromosome 18
ELISA5

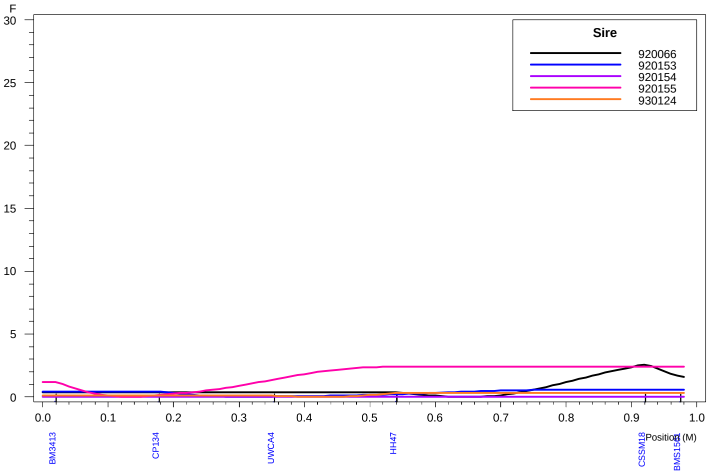

Haley-Knott QTL Analysis: Chromosome 18
LIGE

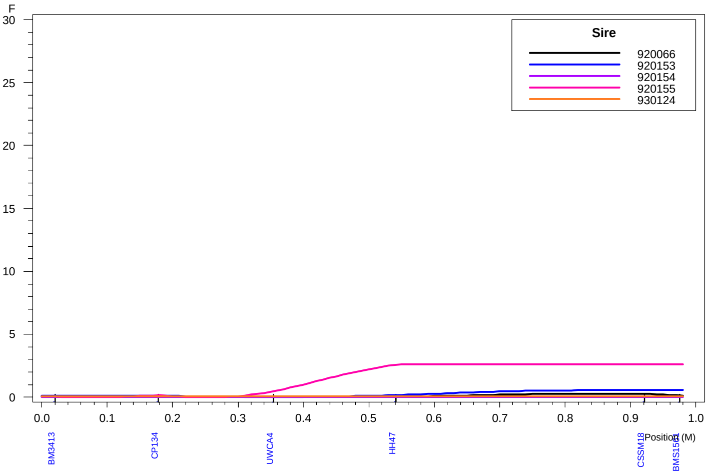

Haley-Knott QTL Analysis: Chromosome 18
WTFEC2

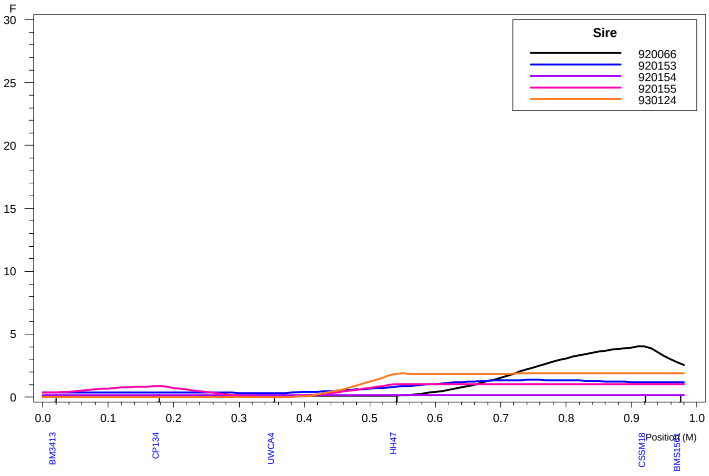

Supplement: Additional File 23 — Chr 18. Haley Knott linkage analysis of sheep chromosome 18. [file 1471-2164-7-178-S23.pdf]
