# Supplementary material for: Discovery of quantitative trait loci for resistance to parasitic nematode infection in sheep: I. Analysis of outcross pedigrees
Source: BMC Genomics. 2006 Jul 18;7:178. doi: 10.1186/1471-2164-7-178 (PMC1574317; doi:10.1186/1471-2164-7-178)

Linkage Analysis in the Parasite Outcross Flock: Chromosome 19

Information Content: Chromosome 19

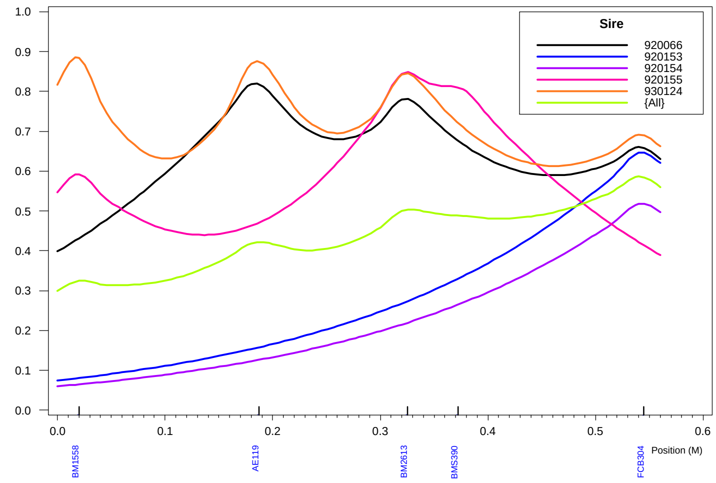

Haley-Knott QTL Analysis: Chromosome 19

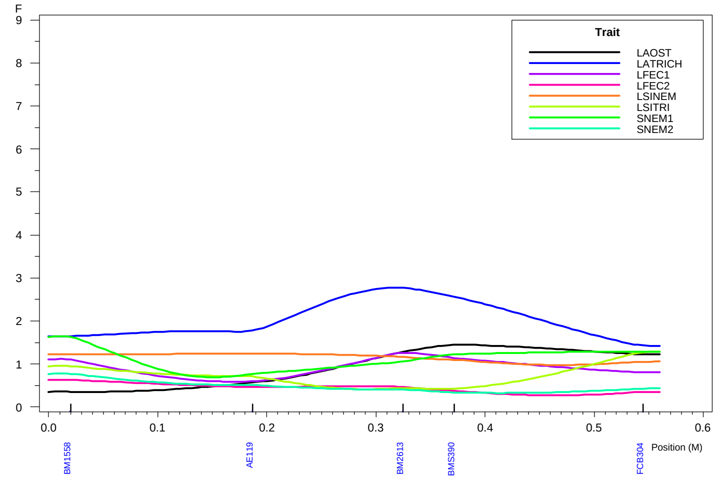

Haley-Knott QTL Analysis: Chromosome 19

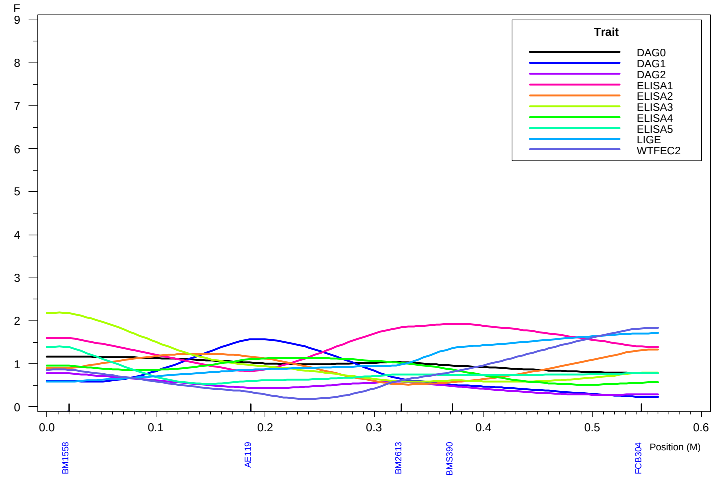

Haley-Knott QTL Analysis: Chromosome 19
LFEC1

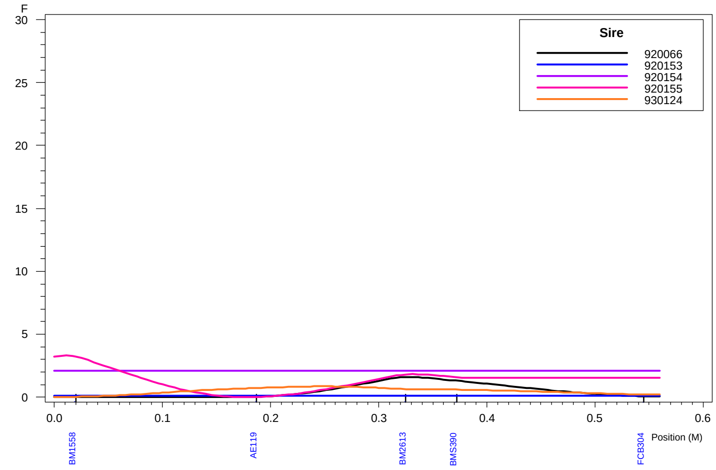

Haley-Knott QTL Analysis: Chromosome 19
LFEC2

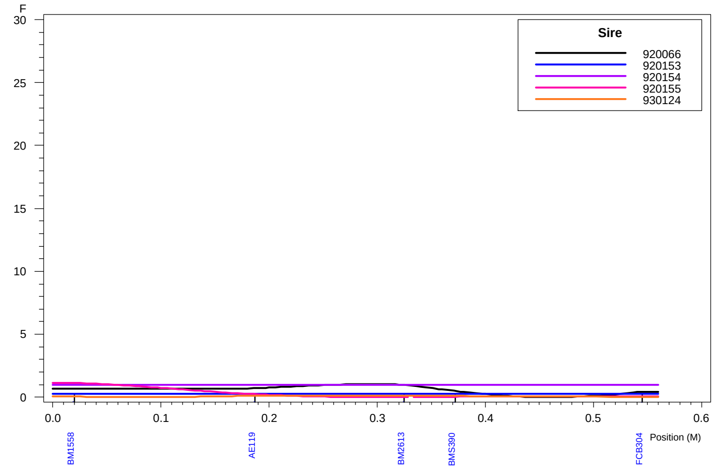

Haley-Knott QTL Analysis: Chromosome 19
SNEM1

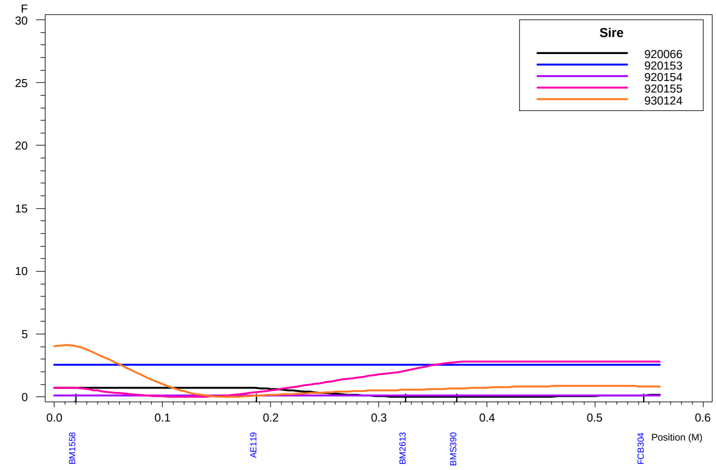

Haley-Knott QTL Analysis: Chromosome 19
SNEM2

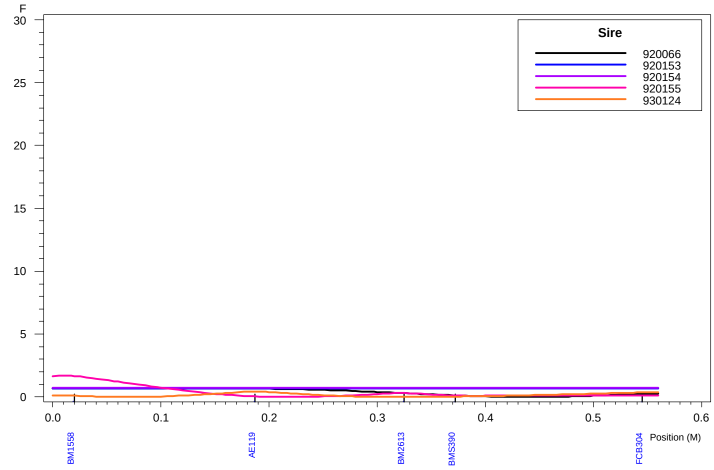

Haley-Knott QTL Analysis: Chromosome 19
LSINEM

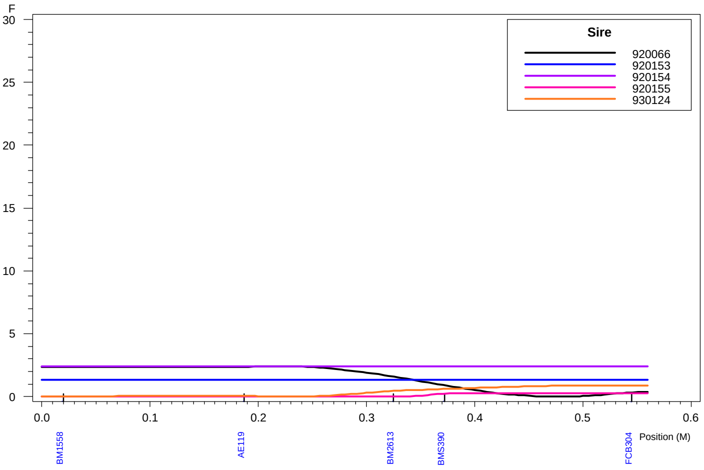

Haley-Knott QTL Analysis: Chromosome 19
LSITRI

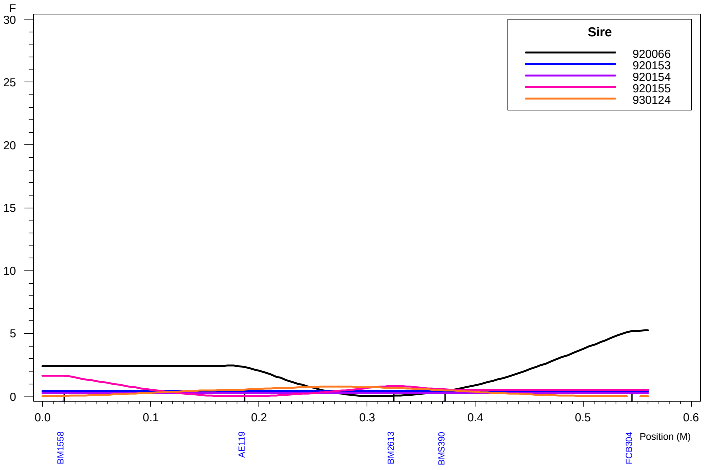

Haley-Knott QTL Analysis: Chromosome 19
LAOST

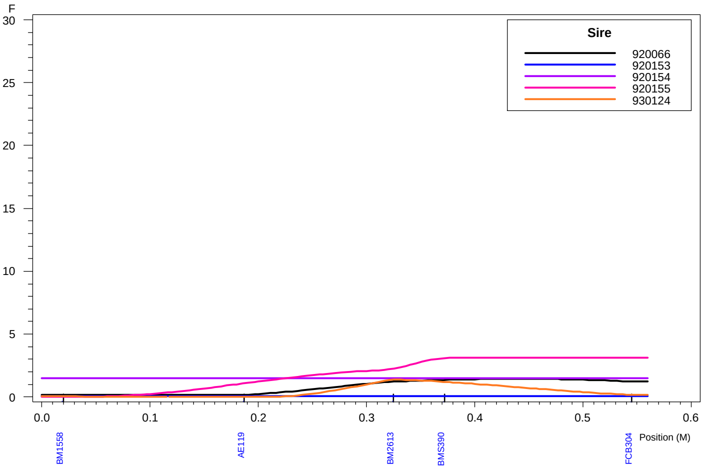

Haley-Knott QTL Analysis: Chromosome 19
LATRICH

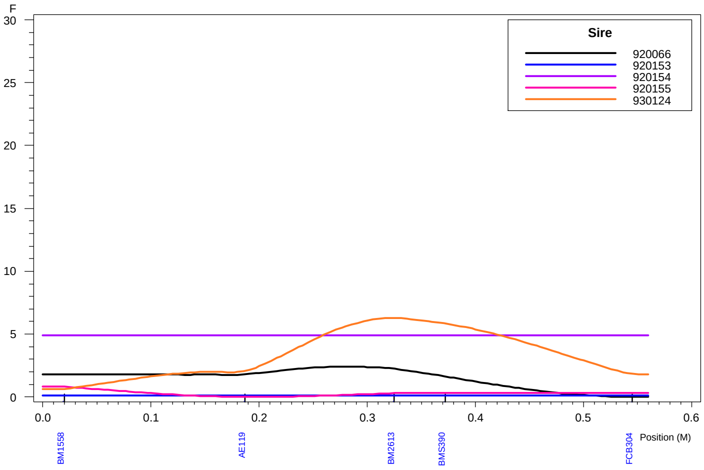

Haley-Knott QTL Analysis: Chromosome 19
DAG0

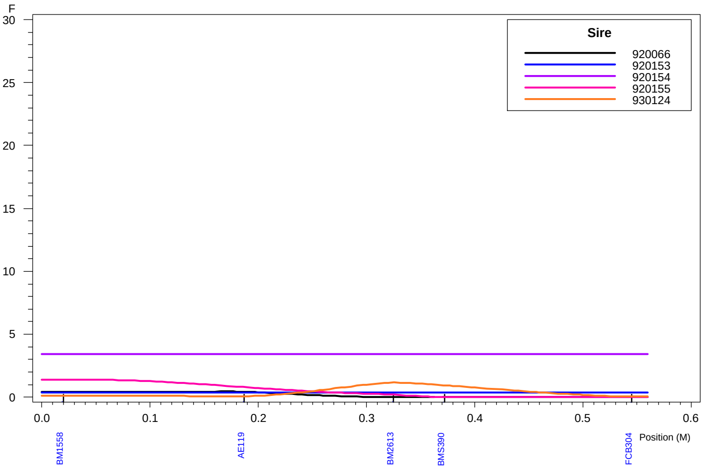

Haley-Knott QTL Analysis: Chromosome 19
DAG1

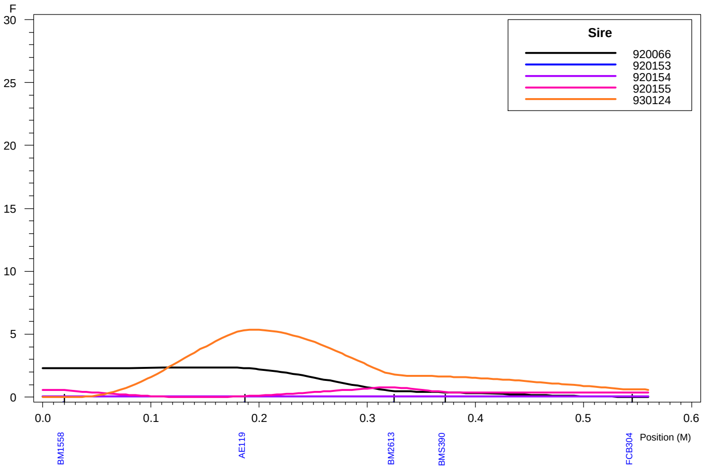

Haley-Knott QTL Analysis: Chromosome 19
DAG2

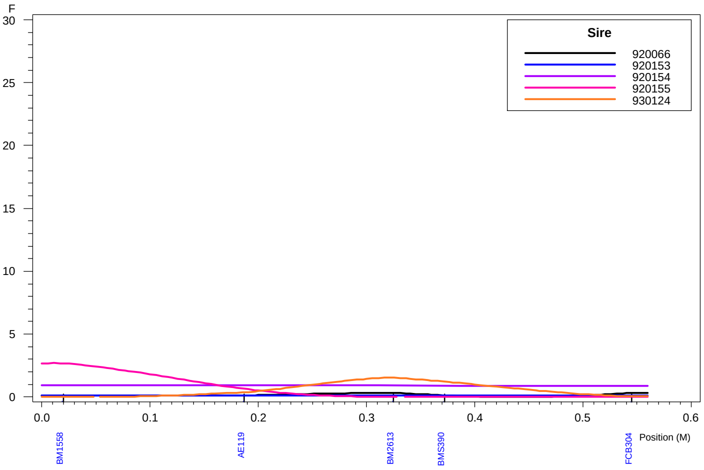

Haley-Knott QTL Analysis: Chromosome 19
ELISA1

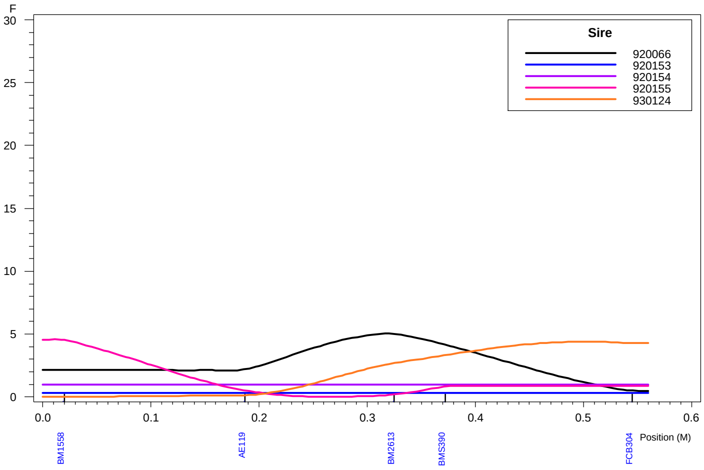

Haley-Knott QTL Analysis: Chromosome 19
ELISA2

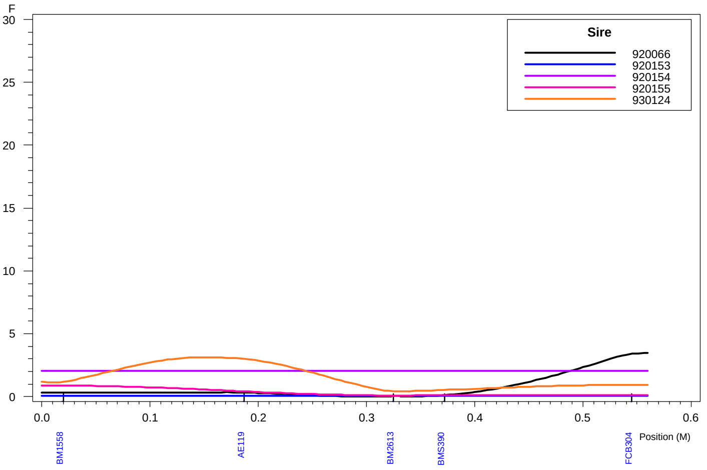

Haley-Knott QTL Analysis: Chromosome 19
ELISA3

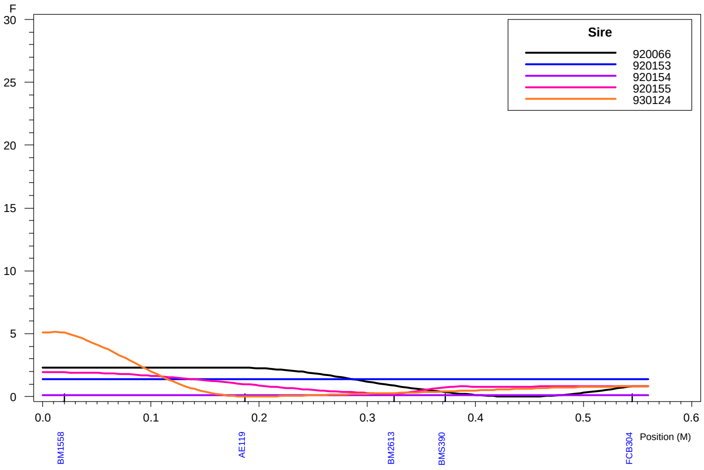

Haley-Knott QTL Analysis: Chromosome 19
ELISA4

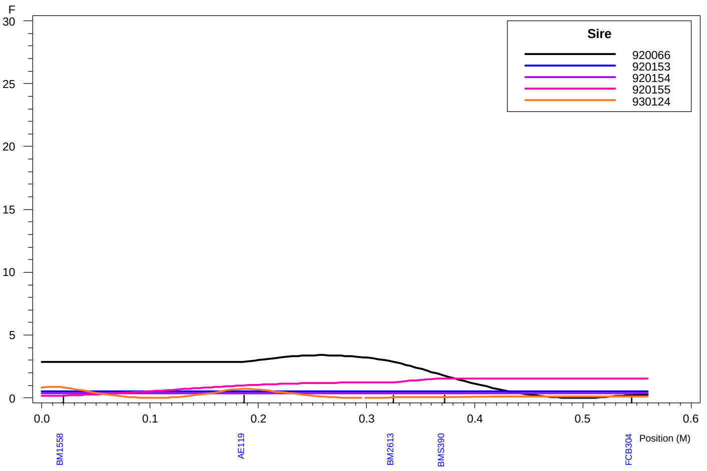

Haley-Knott QTL Analysis: Chromosome 19
ELISA5

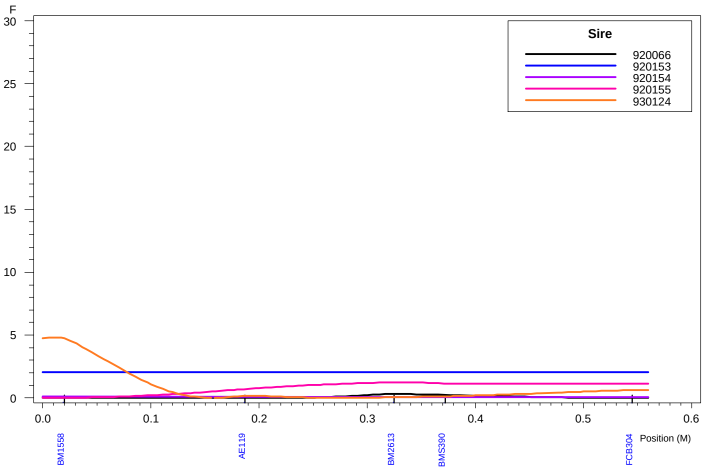

Haley-Knott QTL Analysis: Chromosome 19
LIGE

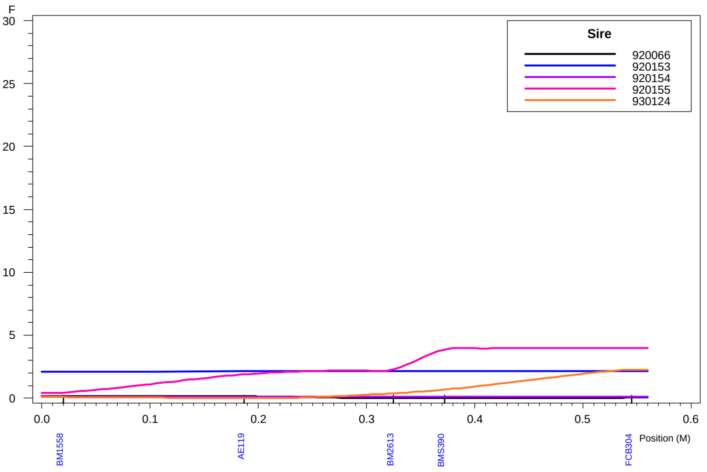

Haley-Knott QTL Analysis: Chromosome 19
WTFEC2

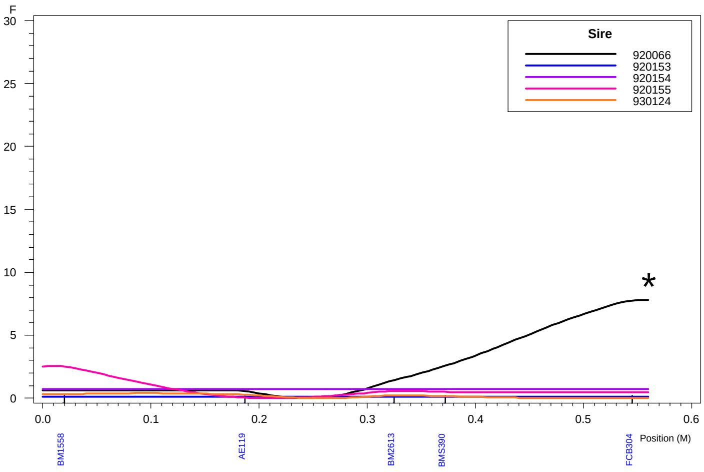

Supplement: Additional File 24 — Chr 19. Haley Knott linkage analysis of sheep chromosome 19. [file 1471-2164-7-178-S24.pdf]
