# Supplementary material for: Discovery of quantitative trait loci for resistance to parasitic nematode infection in sheep: I. Analysis of outcross pedigrees
Source: BMC Genomics. 2006 Jul 18;7:178. doi: 10.1186/1471-2164-7-178 (PMC1574317; doi:10.1186/1471-2164-7-178)
Supplement: Additional File 25 — Chr 20. Haley Knott linkage analysis of sheep chromosome 20. [file 1471-2164-7-178-S25.pdf]

Linkage Analysis in the Parasite Outcross Flock: Chromosome 20

Information Content: Chromosome 20

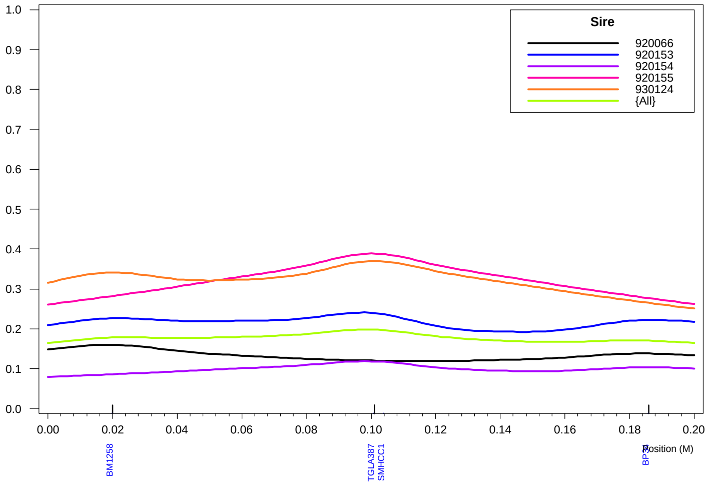

Haley-Knott QTL Analysis: Chromosome 20

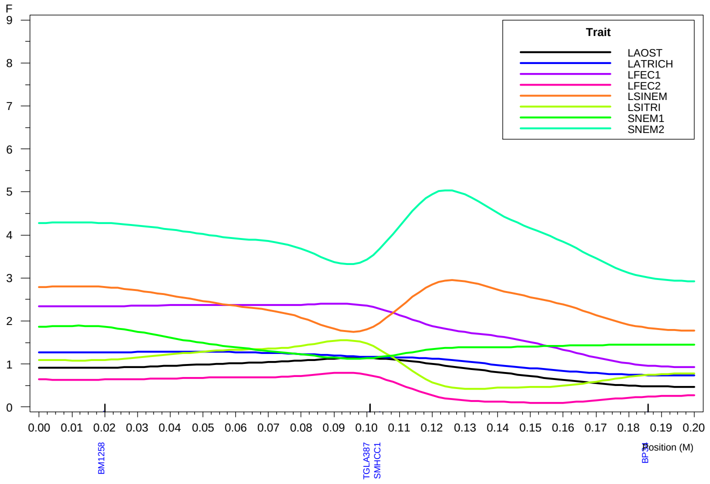

Haley-Knott QTL Analysis: Chromosome 20

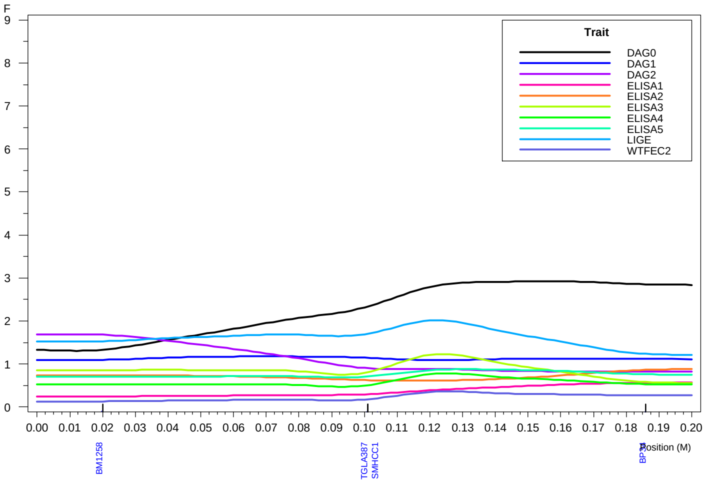

Haley-Knott QTL Analysis: Chromosome 20
LFEC1

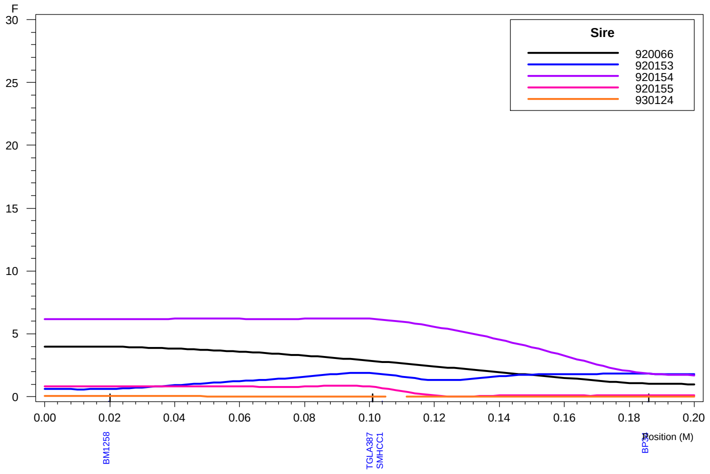

Haley-Knott QTL Analysis: Chromosome 20
LFEC2

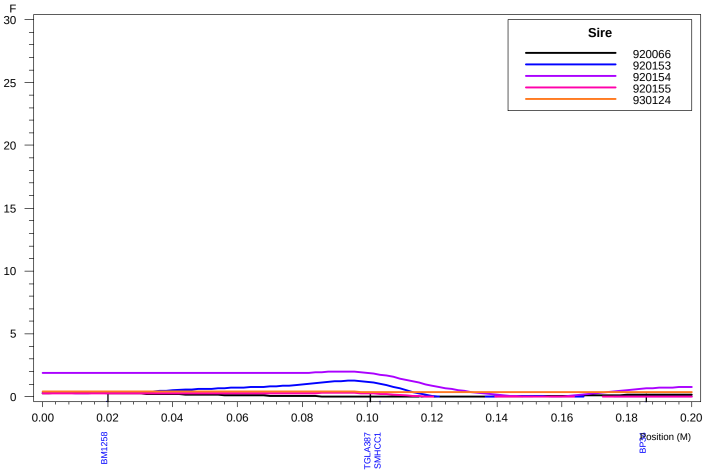

Haley-Knott QTL Analysis: Chromosome 20
SNEM1

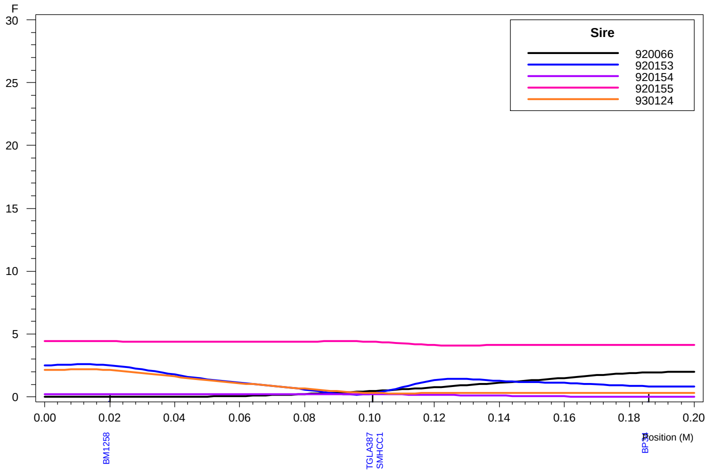

Haley-Knott QTL Analysis: Chromosome 20
SNEM2

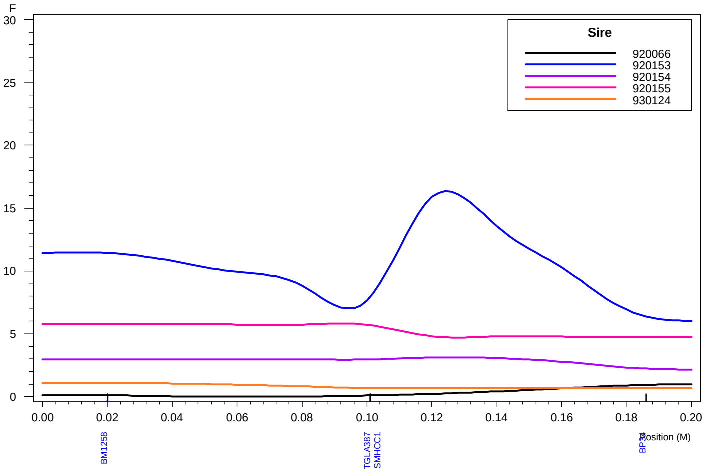

Haley-Knott QTL Analysis: Chromosome 20
LSINEM

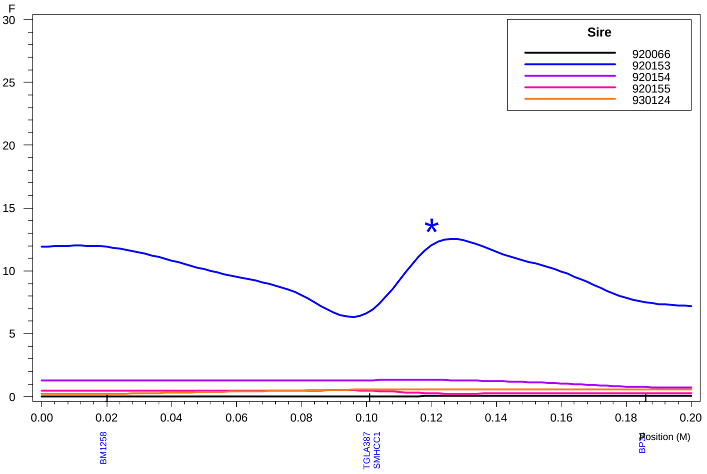

Haley-Knott QTL Analysis: Chromosome 20
LSITRI

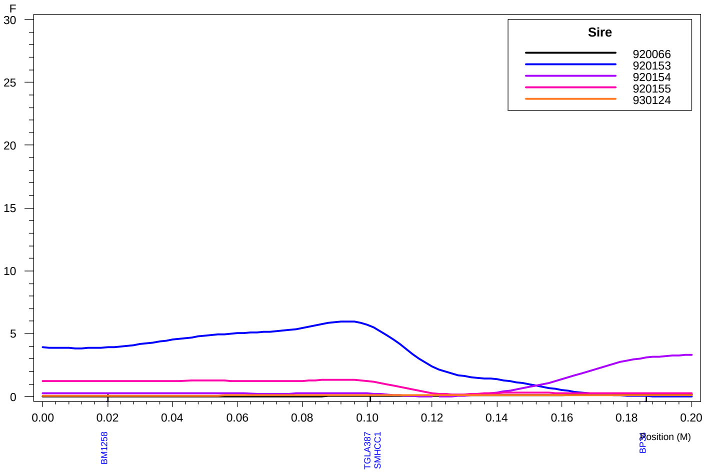

Haley-Knott QTL Analysis: Chromosome 20
LAOST

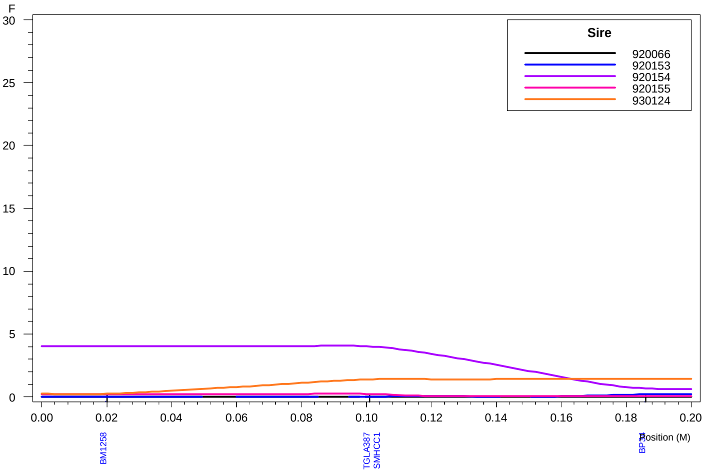

Haley-Knott QTL Analysis: Chromosome 20
LATRICH

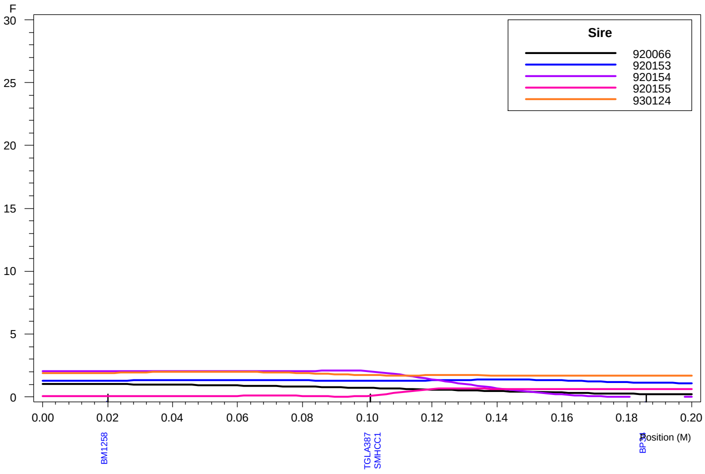

Haley-Knott QTL Analysis: Chromosome 20
DAG0

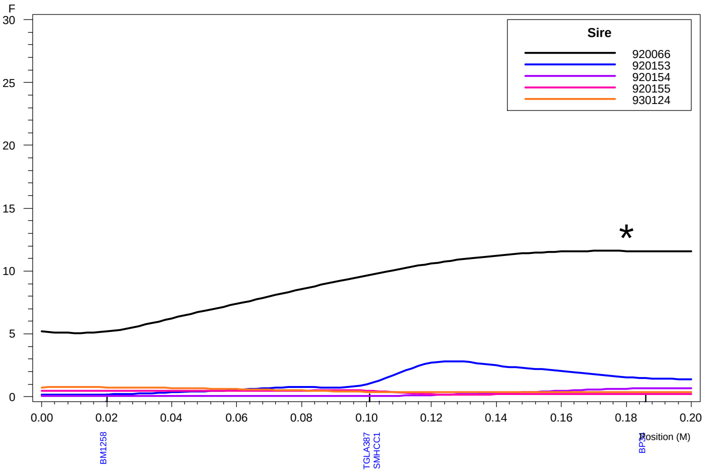

Haley-Knott QTL Analysis: Chromosome 20
DAG1

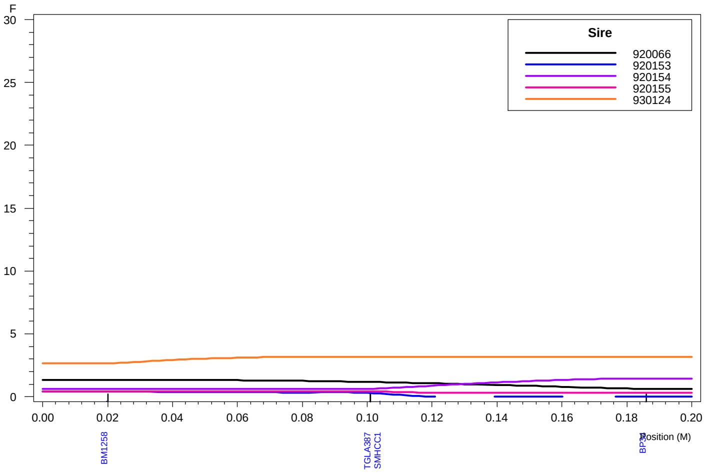

Haley-Knott QTL Analysis: Chromosome 20
DAG2

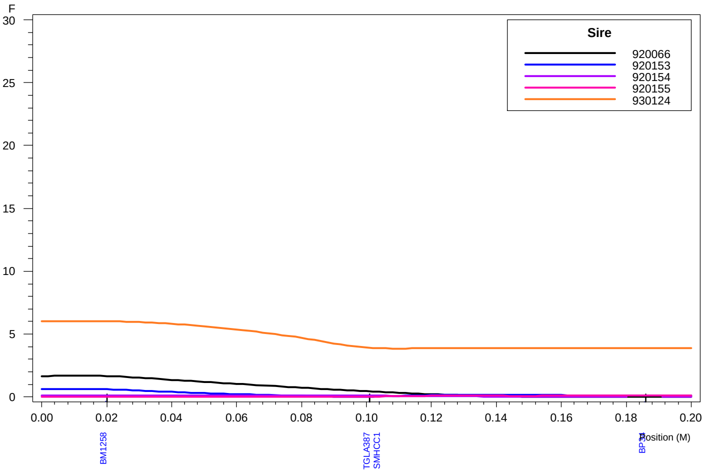

Haley-Knott QTL Analysis: Chromosome 20
ELISA1

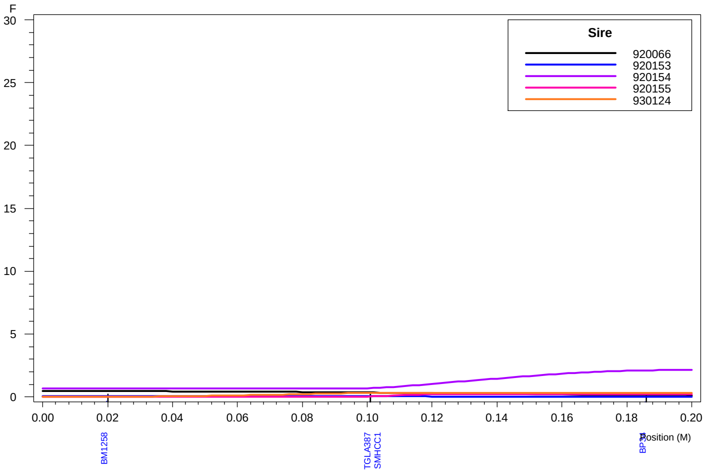

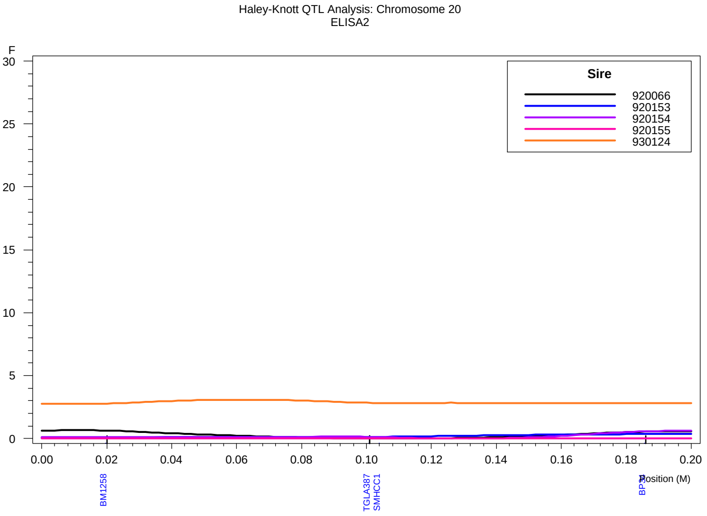
Haley-Knott QTL Analysis: Chromosome 20
ELISA2
F
Sire
920066
920153
920154
920155
930124
Position (M)
BM1258
TGLA387
SMHCC1
BP7

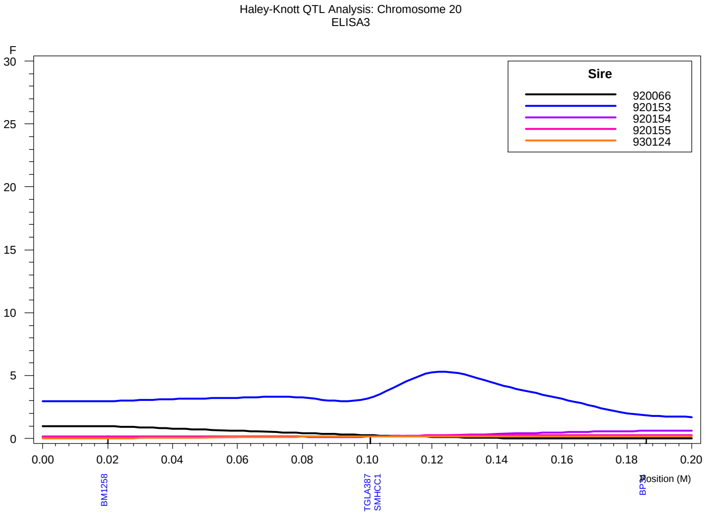
Haley-Knott QTL Analysis: Chromosome 20
ELISA3
F
Sire
920066
920153
920154
920155
930124
Position (M)
BM1258
TGLA387
SMHCC1
BP7

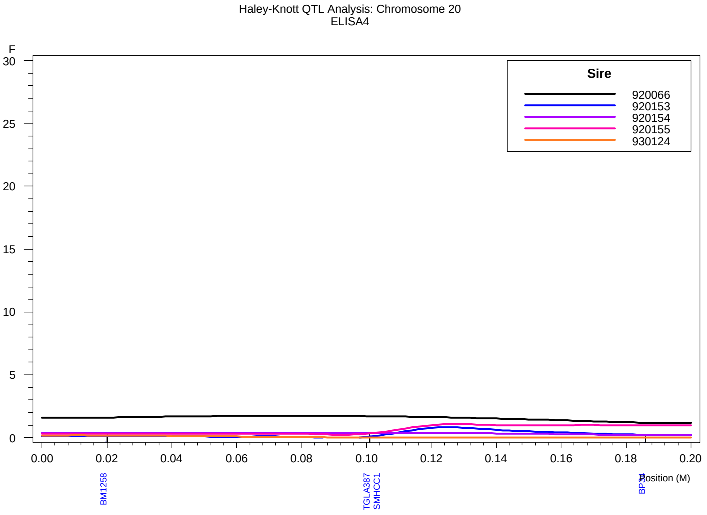
Haley-Knott QTL Analysis: Chromosome 20
ELISA4
F
Sire
920066
920153
920154
920155
930124
Position (M)
BM1258
TGLA387
SMHCC1
BP7

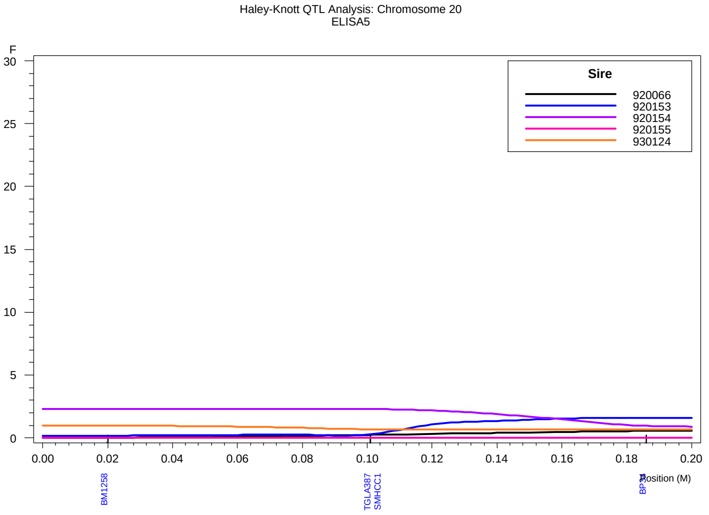
Haley-Knott QTL Analysis: Chromosome 20
ELISA5
F
Sire
920066
920153
920154
920155
930124
Position (M)
BM1258
TGLA387
SMHCC1
BP7

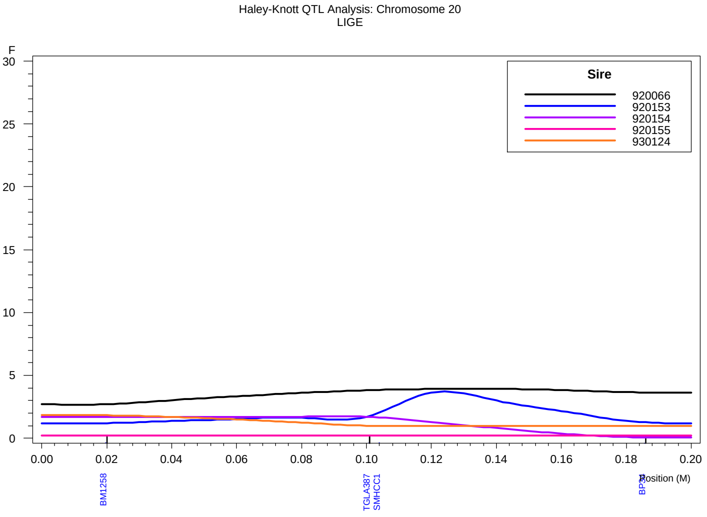
Haley-Knott QTL Analysis: Chromosome 20
LIGE
F
Sire
920066
920153
920154
920155
930124
Position (M)
BM1258
TGLA387
SMHCC1
BP7

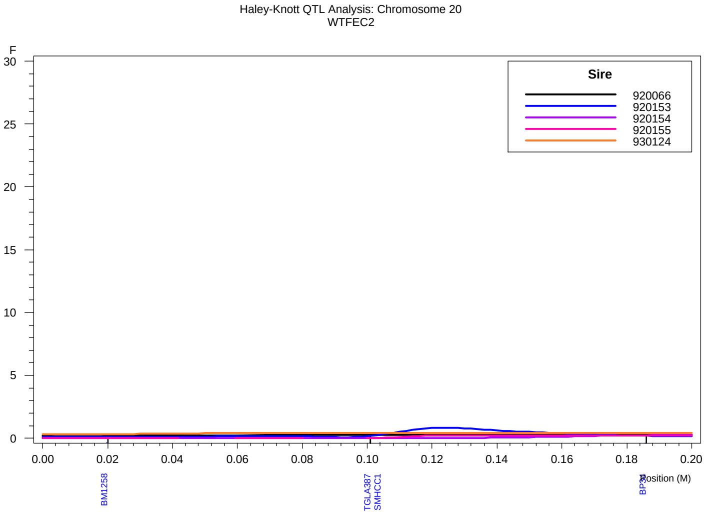
Haley-Knott QTL Analysis: Chromosome 20
WTFEC2
F
Sire
920066
920153
920154
920155
930124
Position (M)
BM1258
TGLA387
SMHCC1
BP7
