# Supplementary material for: Discovery of quantitative trait loci for resistance to parasitic nematode infection in sheep: I. Analysis of outcross pedigrees
Source: BMC Genomics. 2006 Jul 18;7:178. doi: 10.1186/1471-2164-7-178 (PMC1574317; doi:10.1186/1471-2164-7-178)
Supplement: Additional File 26 — Chr 21. Haley Knott linkage analysis of sheep chromosome 21. [file 1471-2164-7-178-S26.pdf]

Linkage Analysis in the Parasite Outcross Flock: Chromosome 21

Information Content: Chromosome 21

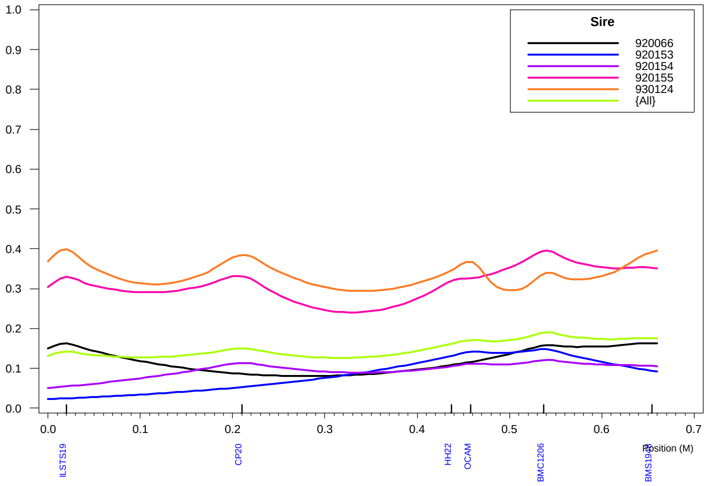

Haley-Knott QTL Analysis: Chromosome 21

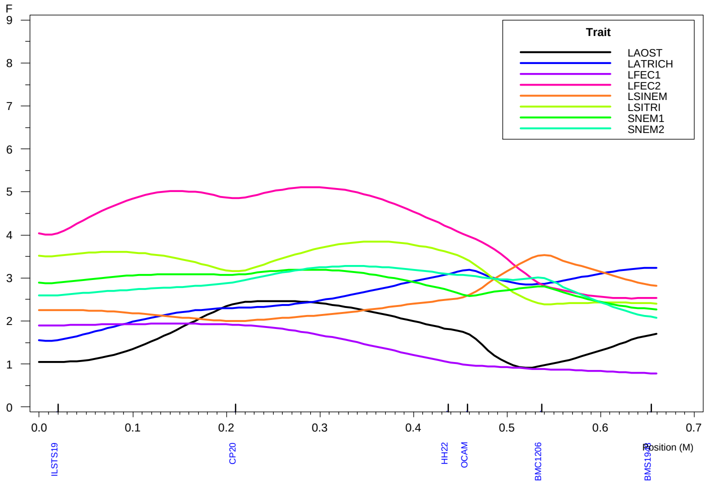

Haley-Knott QTL Analysis: Chromosome 21

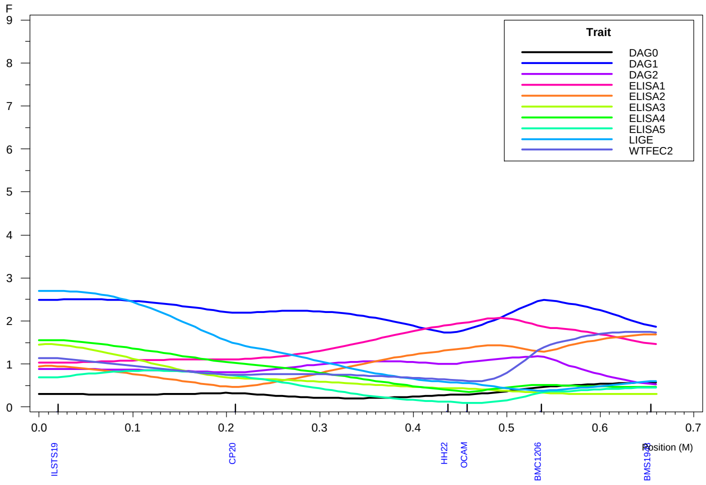

Haley-Knott QTL Analysis: Chromosome 21
LFEC1

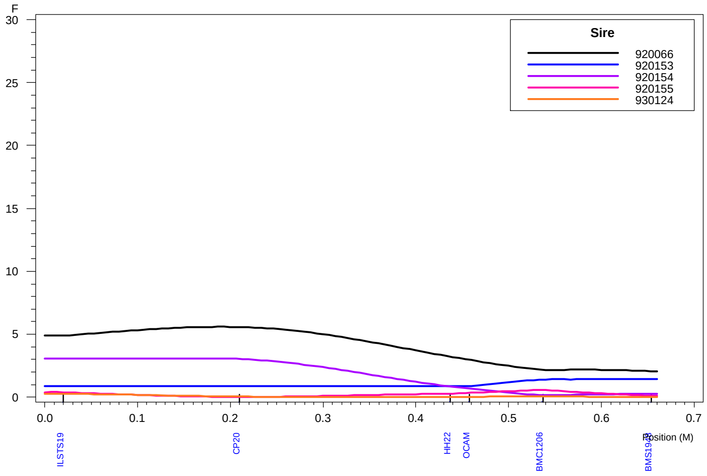

Haley-Knott QTL Analysis: Chromosome 21
LFEC2

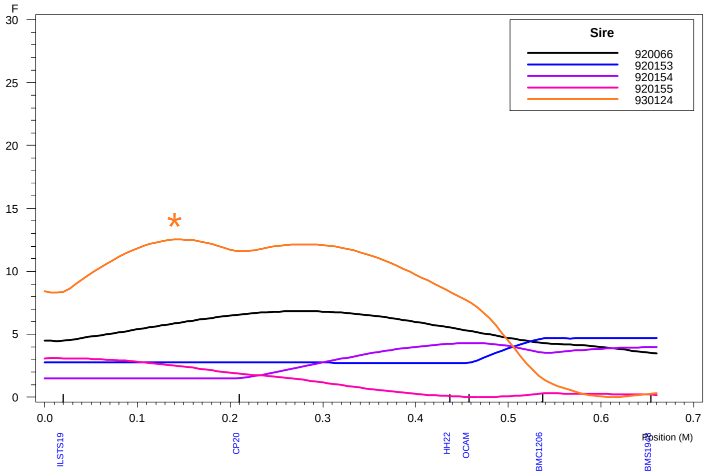

Haley-Knott QTL Analysis: Chromosome 21
SNEM1

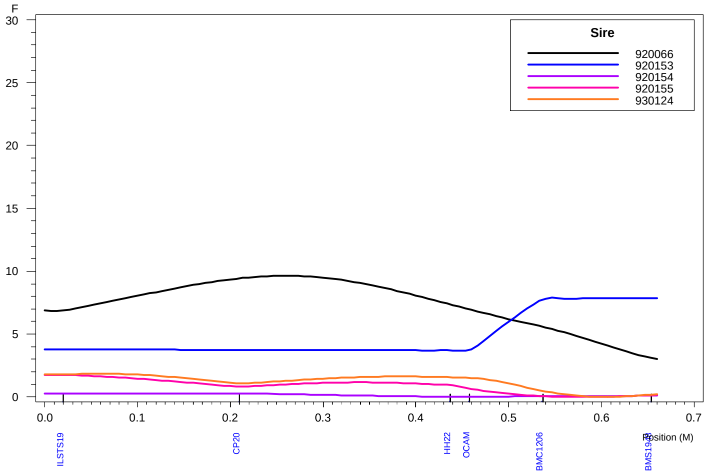

Haley-Knott QTL Analysis: Chromosome 21
SNEM2

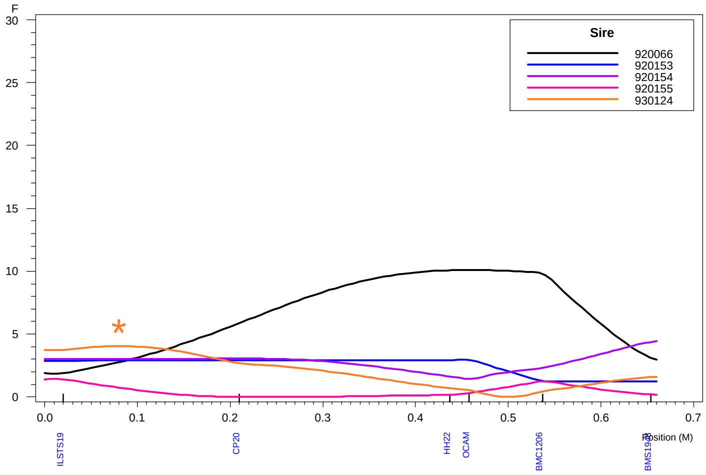

Haley-Knott QTL Analysis: Chromosome 21
LSINEM

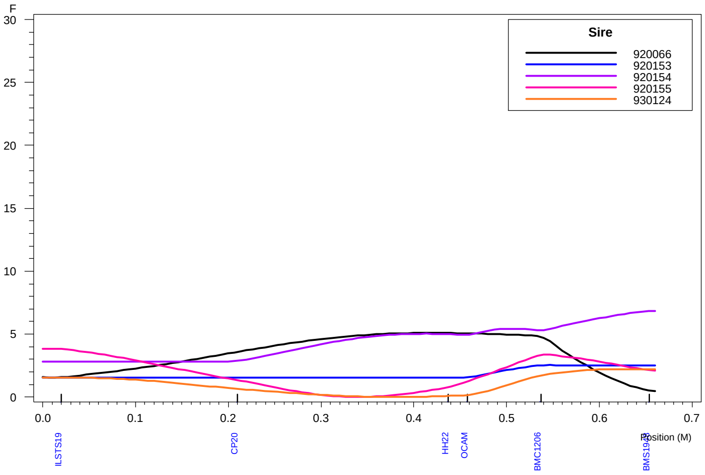

Haley-Knott QTL Analysis: Chromosome 21
LSITRI

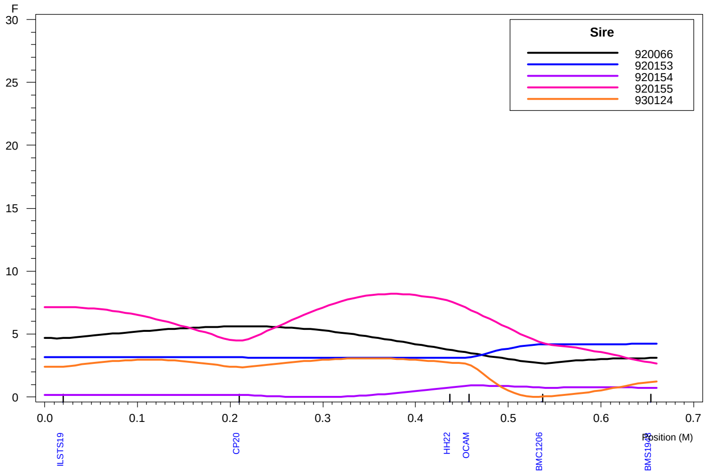

Haley-Knott QTL Analysis: Chromosome 21
LAOST

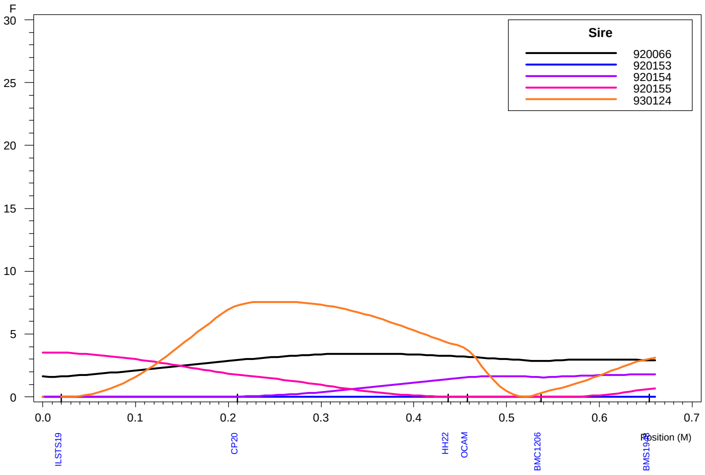

Haley-Knott QTL Analysis: Chromosome 21
LATRICH

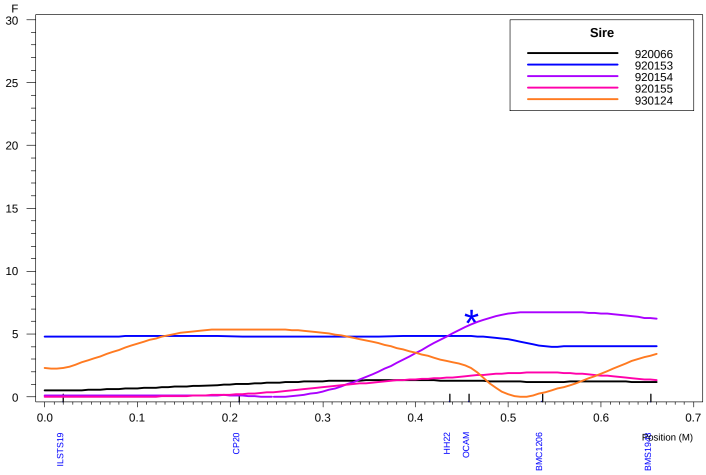

Haley-Knott QTL Analysis: Chromosome 21
DAG0

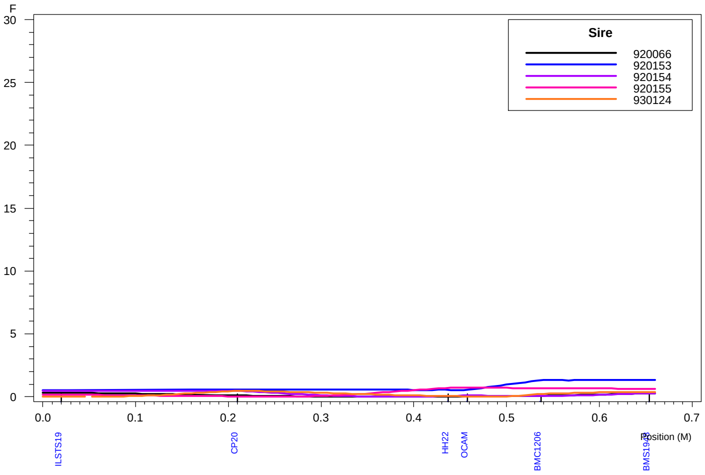

Haley-Knott QTL Analysis: Chromosome 21
DAG1

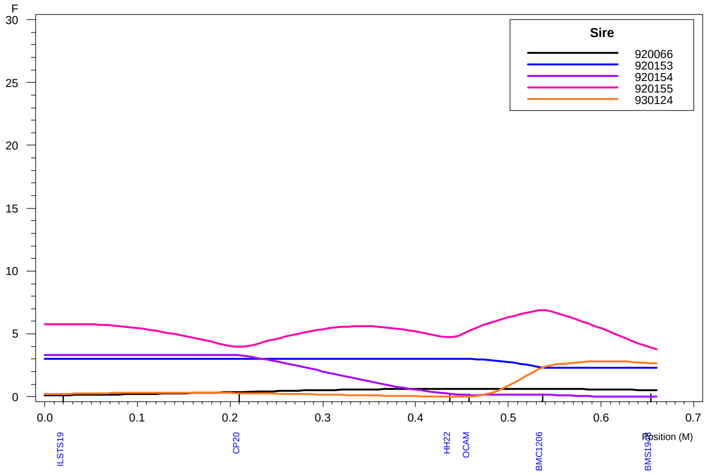

Haley-Knott QTL Analysis: Chromosome 21
DAG2

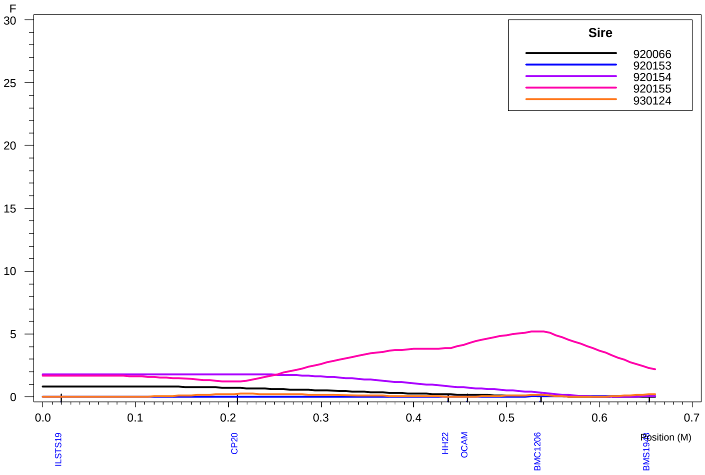

Haley-Knott QTL Analysis: Chromosome 21
ELISA1

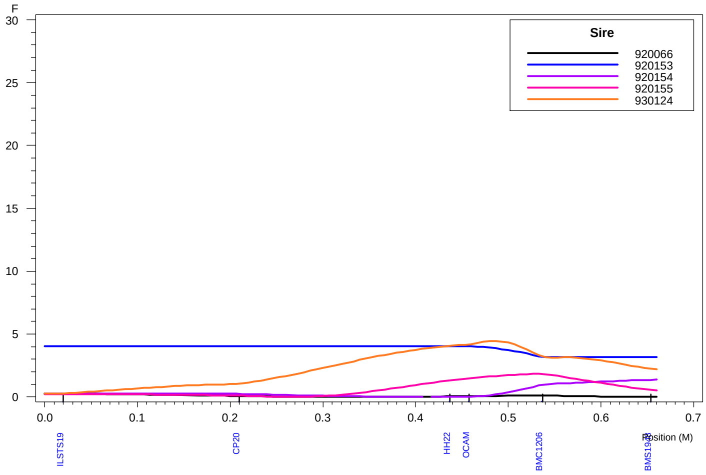

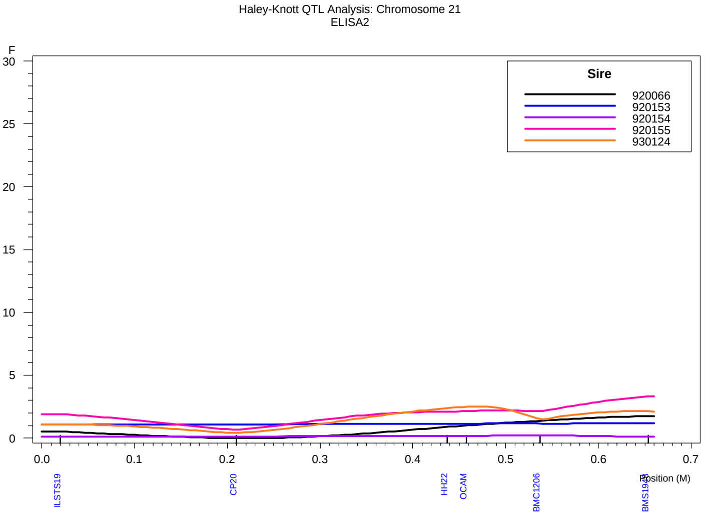
Haley-Knott QTL Analysis: Chromosome 21
ELISA2
F
30
25
20
15
10
5
0
Sire
920066
920153
920154
920155
930124
0.0
0.1
0.2
0.3
0.4
0.5
0.6
0.7
Position (M)
ILSTS19
CP20
HH22
OCAM
BMC1206
BMS1948

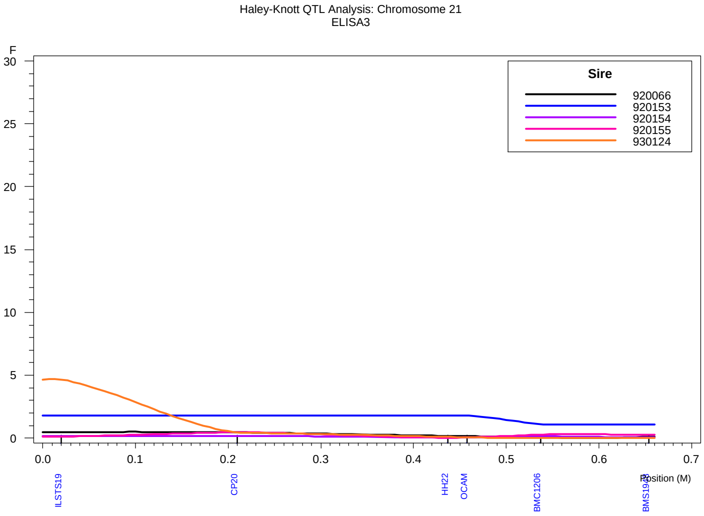
Haley-Knott QTL Analysis: Chromosome 21
ELISA3
F
30
25
20
15
10
5
0
Sire
920066
920153
920154
920155
930124
0.0
0.1
0.2
0.3
0.4
0.5
0.6
0.7
Position (M)
ILSTS19
CP20
HH22
OCAM
BMC1206
BMS1948

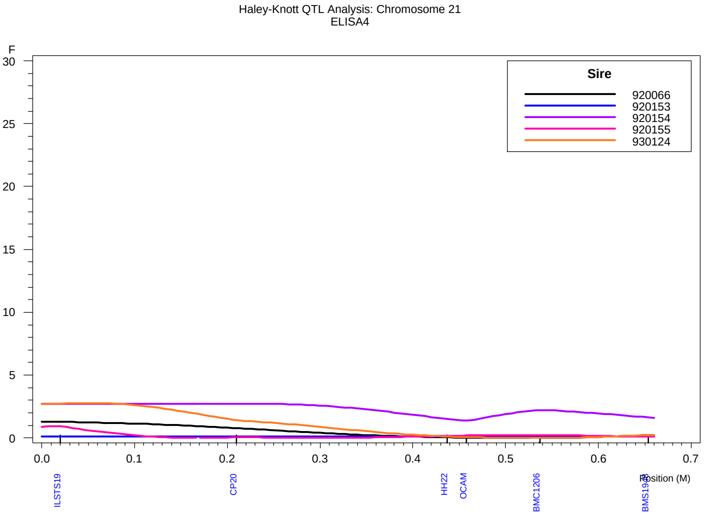
Haley-Knott QTL Analysis: Chromosome 21
ELISA4
F
30
25
20
15
10
5
0
Sire
920066
920153
920154
920155
930124
0.0
0.1
0.2
0.3
0.4
0.5
0.6
0.7
Position (M)
ILSTS19
CP20
HH22
OCAM
BMC1206
BMS1948

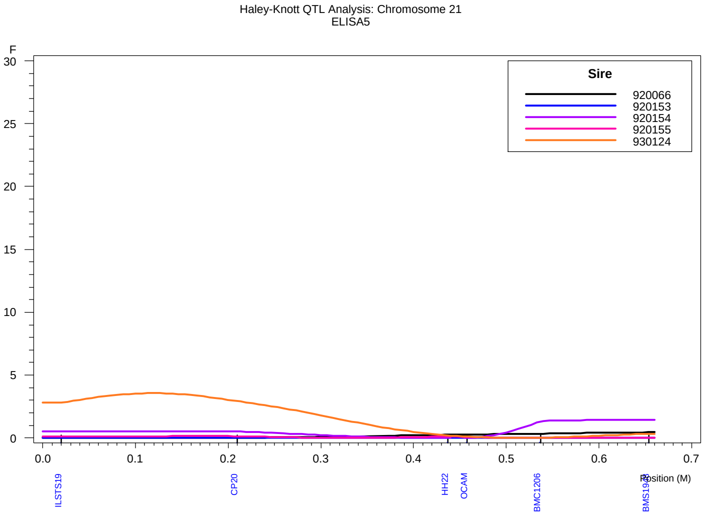
Haley-Knott QTL Analysis: Chromosome 21
ELISA5
F
30
25
20
15
10
5
0
Sire
920066
920153
920154
920155
930124
0.0
0.1
0.2
0.3
0.4
0.5
0.6
0.7
Position (M)
ILSTS19
CP20
HH22
OCAM
BMC1206
BMS1948

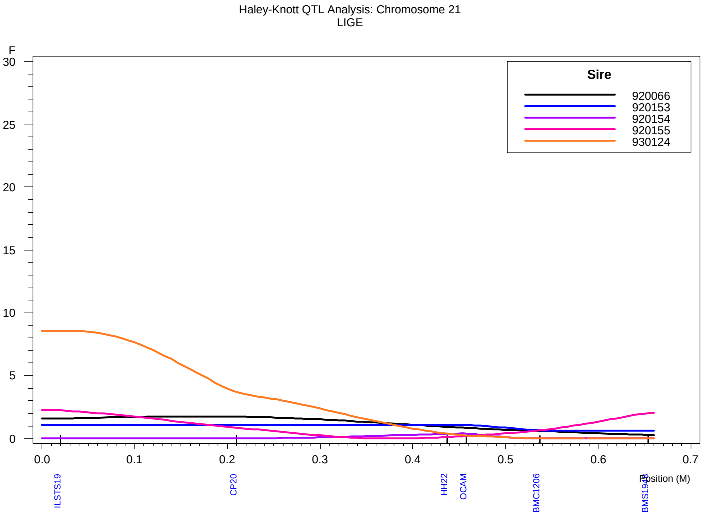
Haley-Knott QTL Analysis: Chromosome 21
LIGE
F
30
25
20
15
10
5
0
Sire
920066
920153
920154
920155
930124
0.0
0.1
0.2
0.3
0.4
0.5
0.6
0.7
Position (M)
ILSTS19
CP20
HH22
OCAM
BMC1206
BMS1948

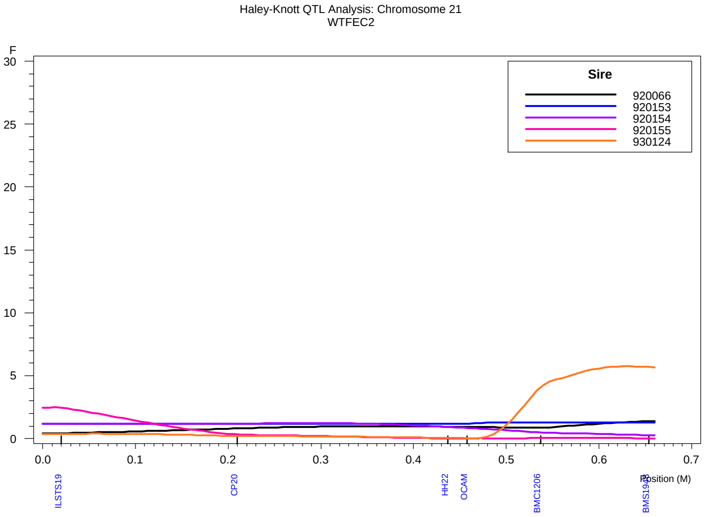
Haley-Knott QTL Analysis: Chromosome 21
WTFEC2
F
30
25
20
15
10
5
0
Sire
920066
920153
920154
920155
930124
0.0
0.1
0.2
0.3
0.4
0.5
0.6
0.7
Position (M)
ILSTS19
CP20
HH22
OCAM
BMC1206
BMS1948
