# Supplementary material for: Discovery of quantitative trait loci for resistance to parasitic nematode infection in sheep: I. Analysis of outcross pedigrees
Source: BMC Genomics. 2006 Jul 18;7:178. doi: 10.1186/1471-2164-7-178 (PMC1574317; doi:10.1186/1471-2164-7-178)

Linkage Analysis in the Parasite Outcross Flock: Chromosome 22

Information Content: Chromosome 22

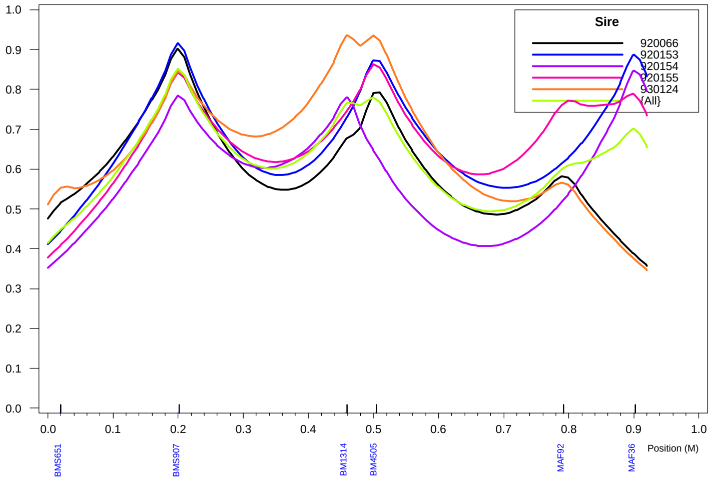

Haley-Knott QTL Analysis: Chromosome 22

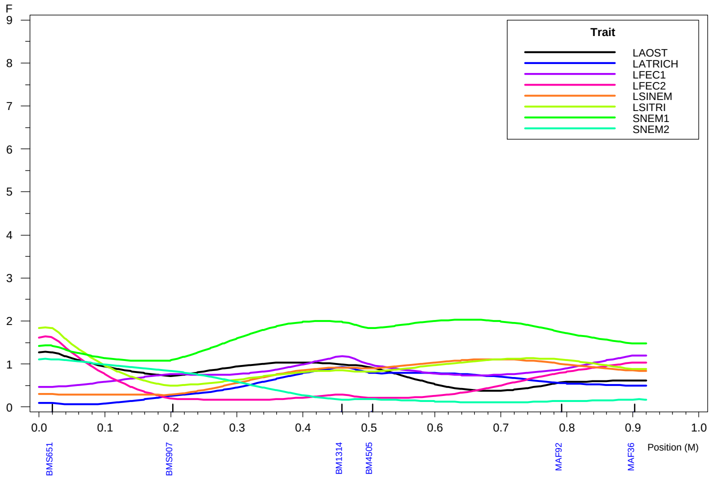

Haley-Knott QTL Analysis: Chromosome 22

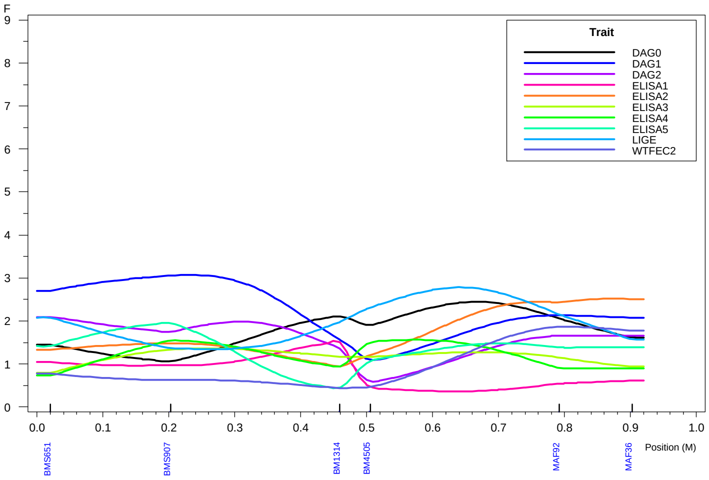

Haley-Knott QTL Analysis: Chromosome 22
LFEC1

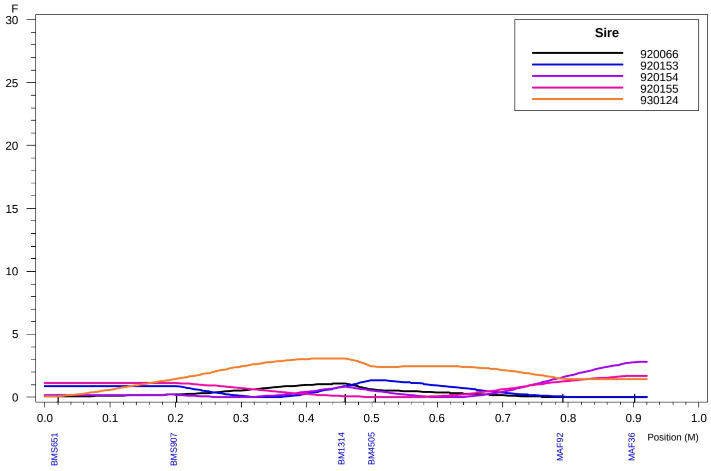

Haley-Knott QTL Analysis: Chromosome 22
LFEC2

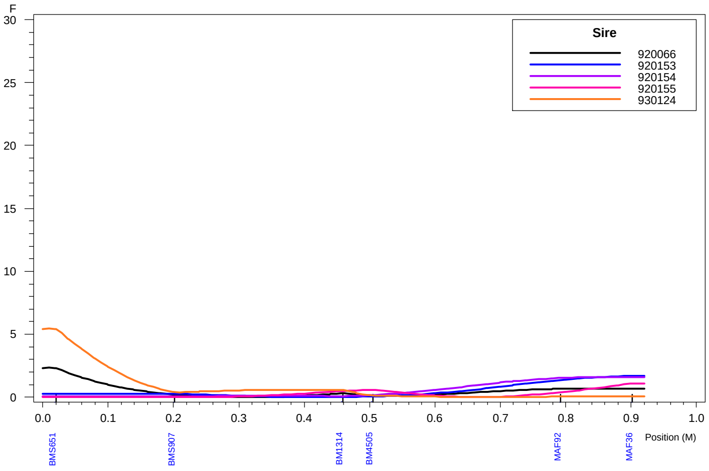

Haley-Knott QTL Analysis: Chromosome 22
SNEM1

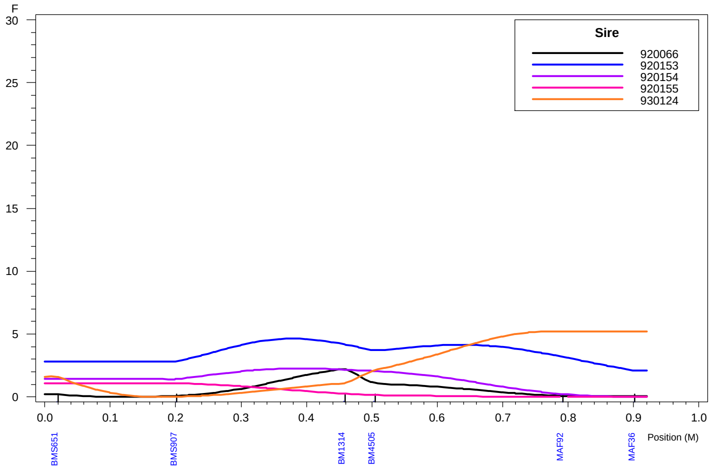

Haley-Knott QTL Analysis: Chromosome 22
SNEM2

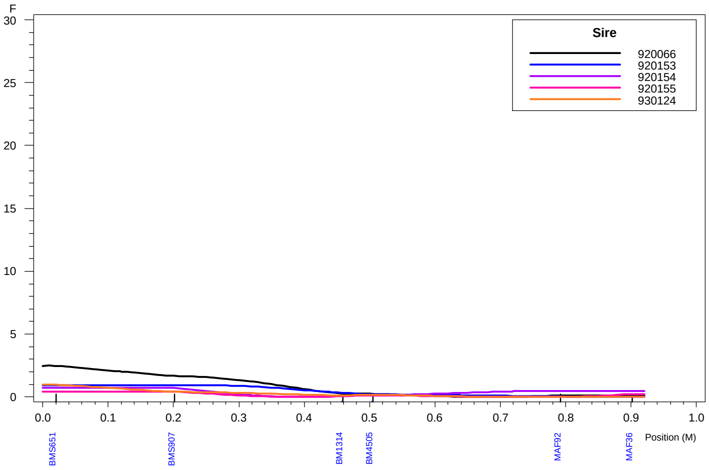

Haley-Knott QTL Analysis: Chromosome 22
LSINEM

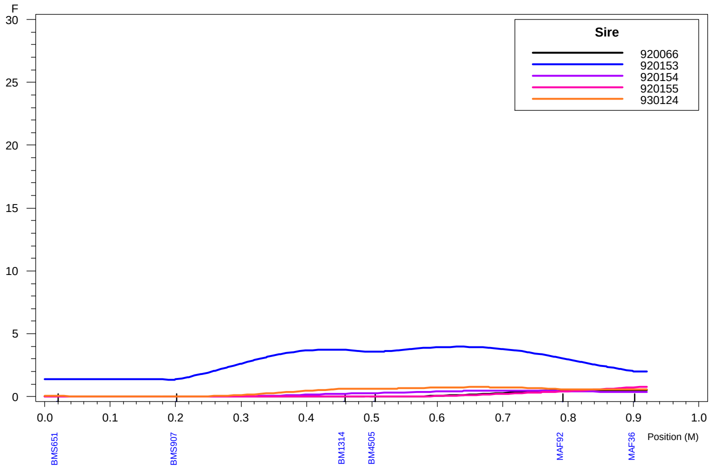

Haley-Knott QTL Analysis: Chromosome 22
LSITRI

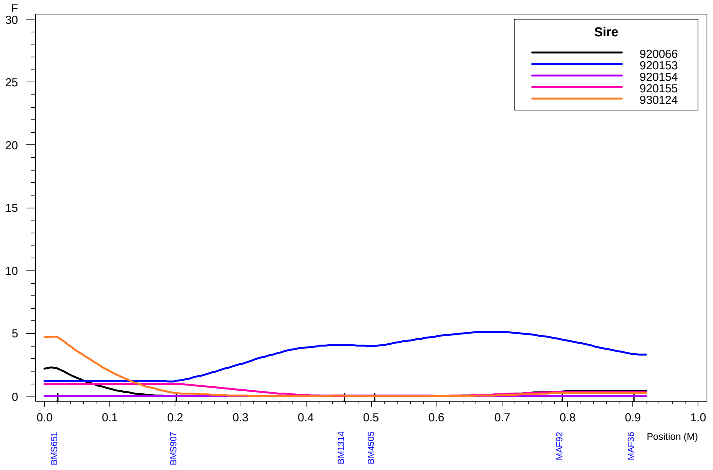

Haley-Knott QTL Analysis: Chromosome 22
LAOST

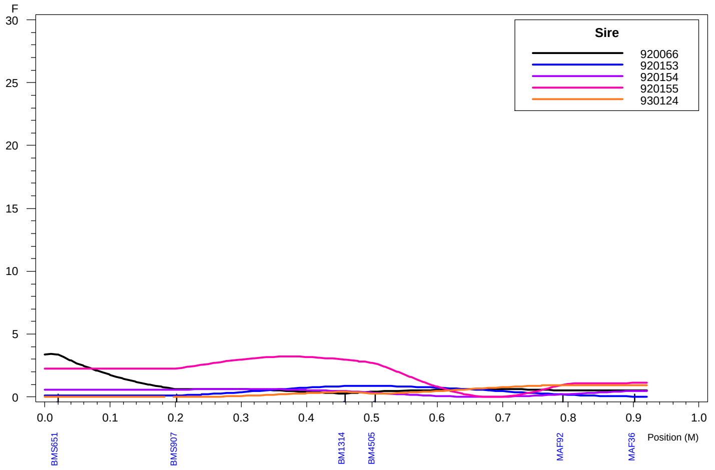

Haley-Knott QTL Analysis: Chromosome 22
LATRICH

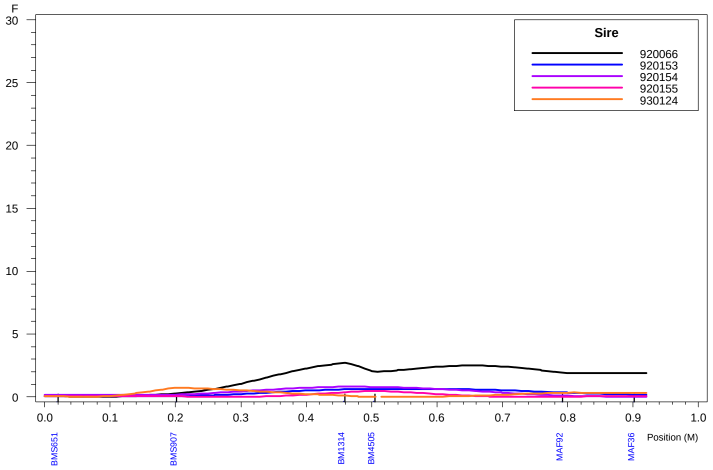

Haley-Knott QTL Analysis: Chromosome 22
DAG0

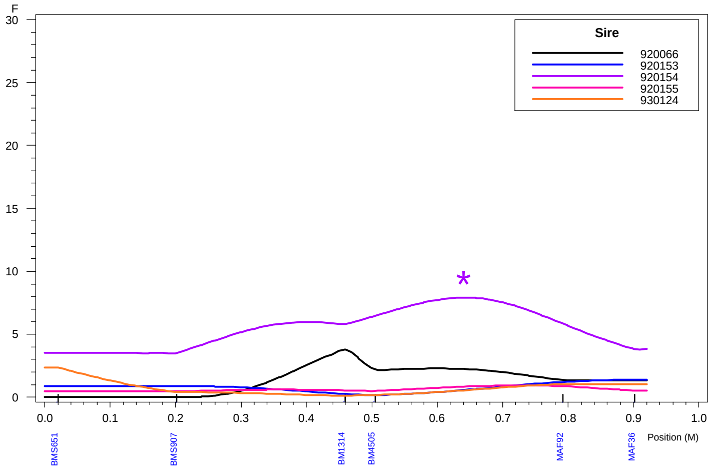

Haley-Knott QTL Analysis: Chromosome 22
DAG1

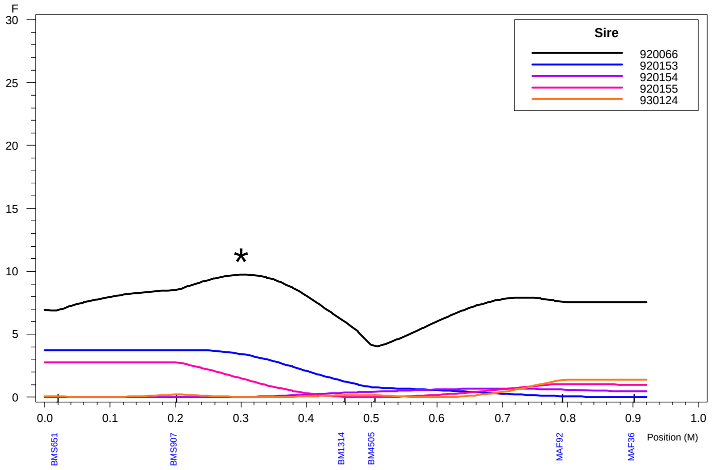

Haley-Knott QTL Analysis: Chromosome 22
DAG2

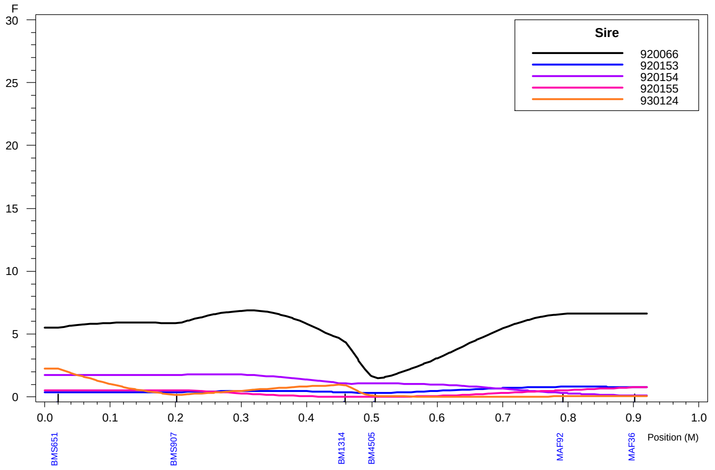

Haley-Knott QTL Analysis: Chromosome 22
ELISA1

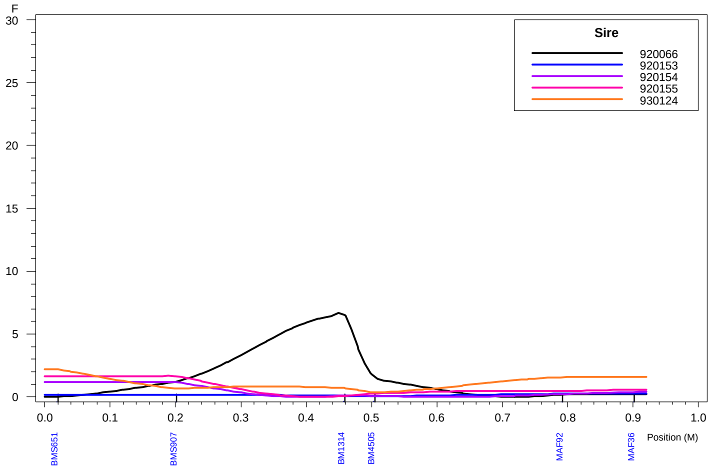

Haley-Knott QTL Analysis: Chromosome 22
ELISA2

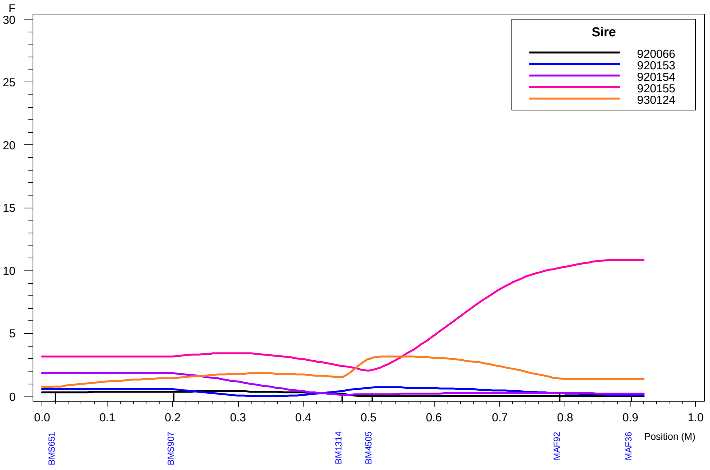

Haley-Knott QTL Analysis: Chromosome 22
ELISA3

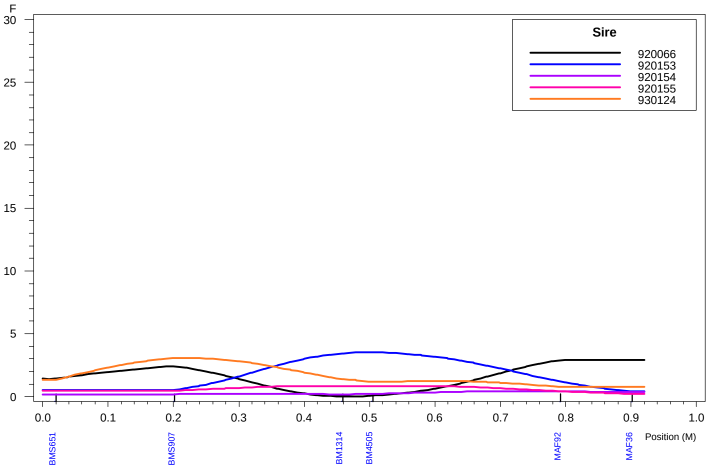

Haley-Knott QTL Analysis: Chromosome 22
ELISA4

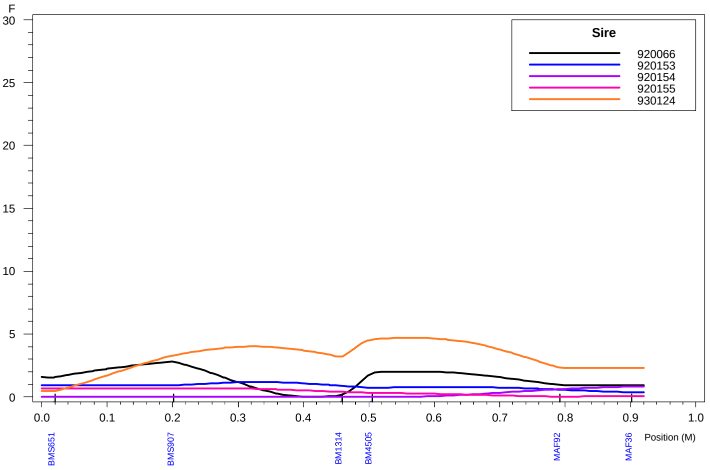

Haley-Knott QTL Analysis: Chromosome 22
ELISA5

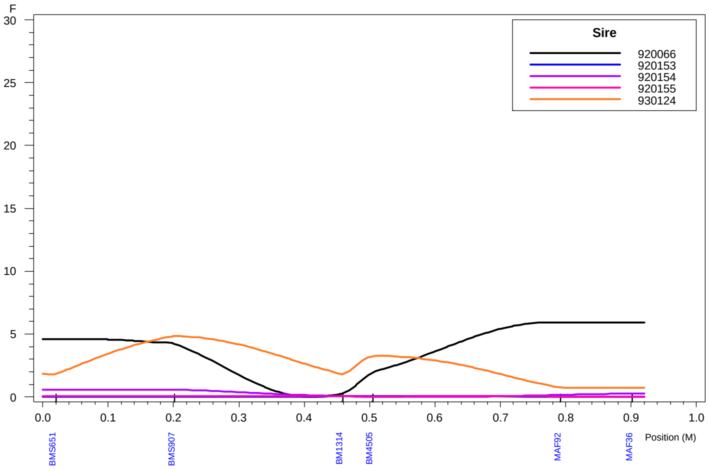

Haley-Knott QTL Analysis: Chromosome 22
LIGE

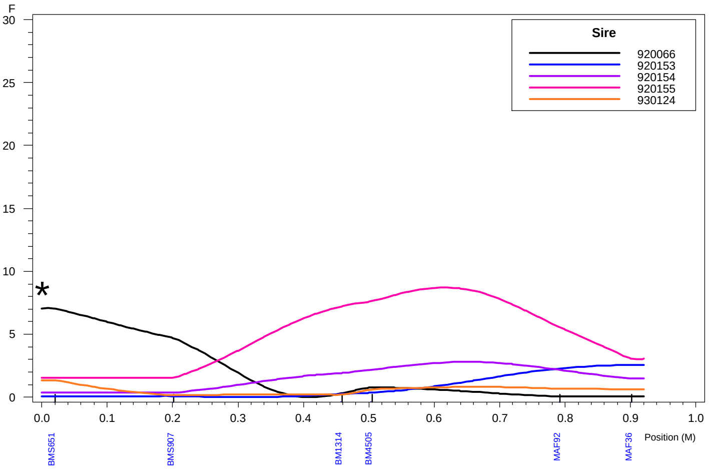

Haley-Knott QTL Analysis: Chromosome 22
WTFEC2

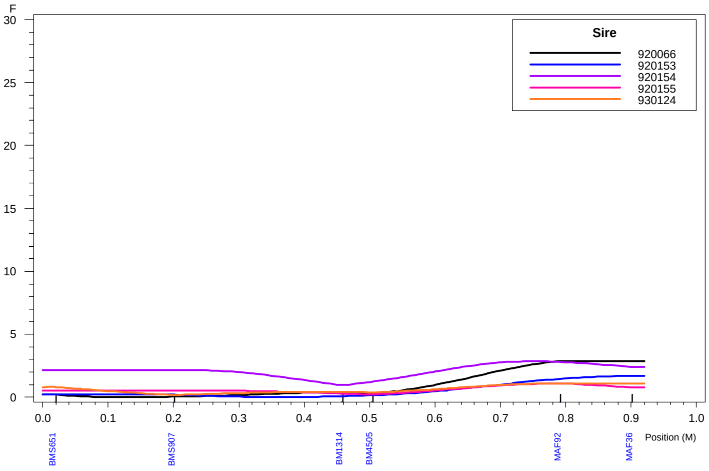

Supplement: Additional File 27 — Chr 22. Haley Knott linkage analysis of sheep chromosome 22. [file 1471-2164-7-178-S27.pdf]
