# Supplementary material for: Discovery of quantitative trait loci for resistance to parasitic nematode infection in sheep: I. Analysis of outcross pedigrees
Source: BMC Genomics. 2006 Jul 18;7:178. doi: 10.1186/1471-2164-7-178 (PMC1574317; doi:10.1186/1471-2164-7-178)
Supplement: Additional File 28 — Chr 23. Haley Knott linkage analysis of sheep chromosome 23. [file 1471-2164-7-178-S28.pdf]

Linkage Analysis in the Parasite Outcross Flock: Chromosome 23

Information Content: Chromosome 23

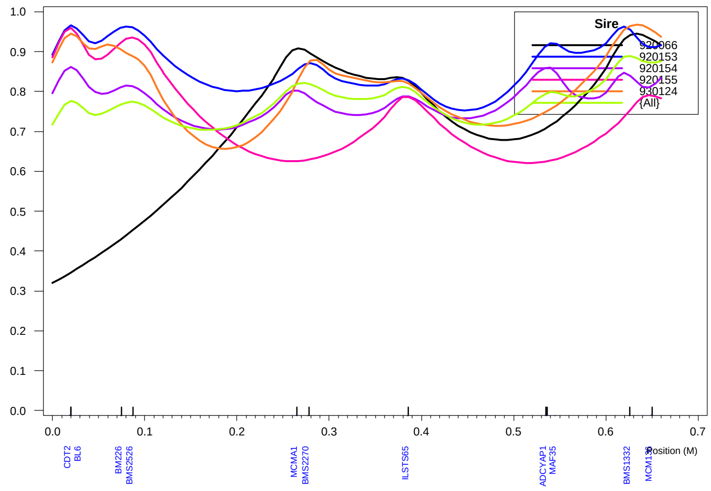

Haley-Knott QTL Analysis: Chromosome 23

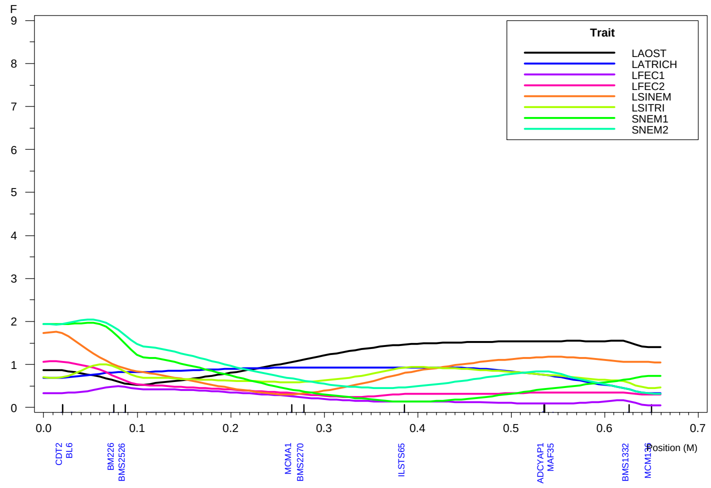

Haley-Knott QTL Analysis: Chromosome 23

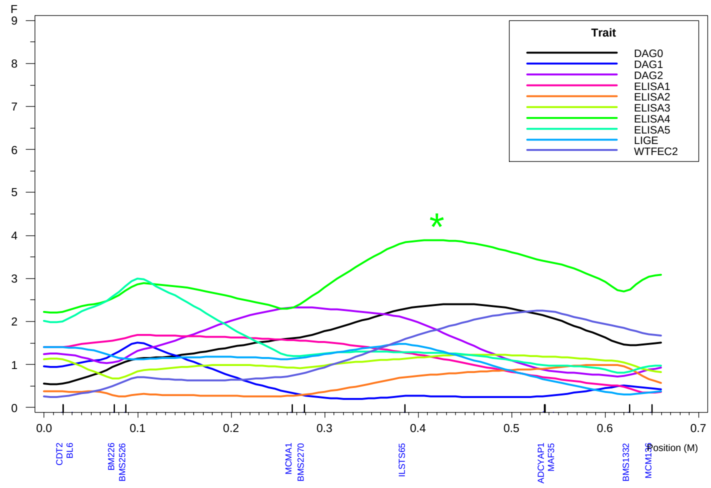

Haley-Knott QTL Analysis: Chromosome 23
LFEC1

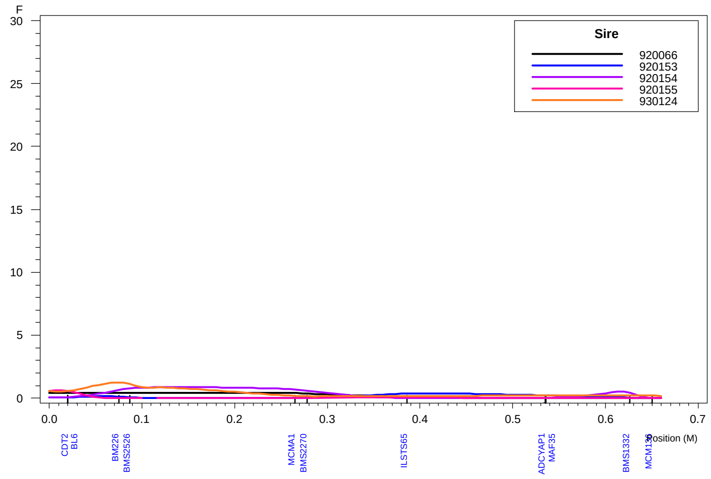

Haley-Knott QTL Analysis: Chromosome 23
LFEC2

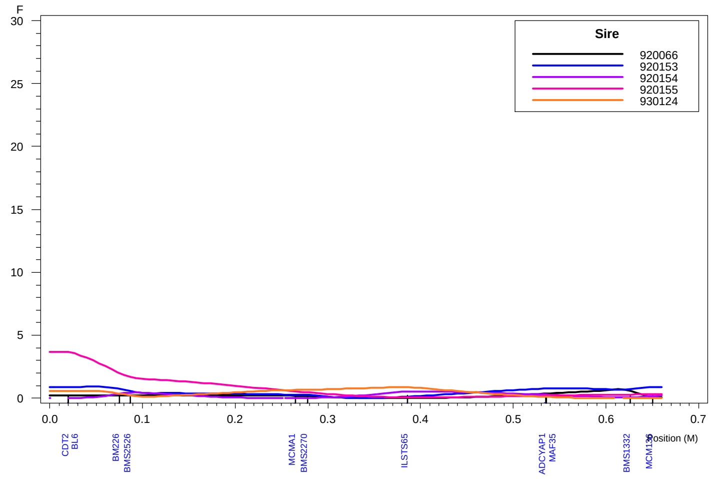

Haley-Knott QTL Analysis: Chromosome 23
SNEM1

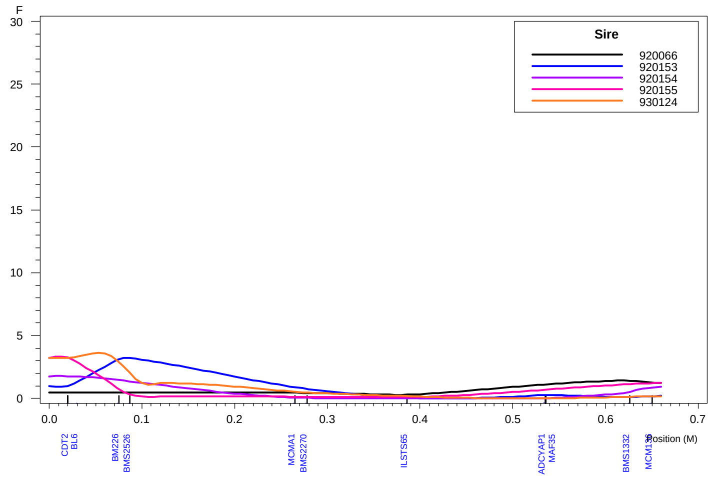

Haley-Knott QTL Analysis: Chromosome 23
SNEM2

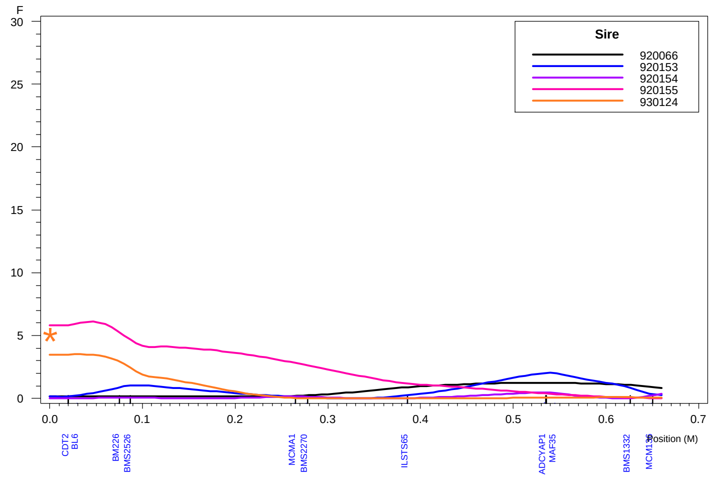

Haley-Knott QTL Analysis: Chromosome 23
LSINEM

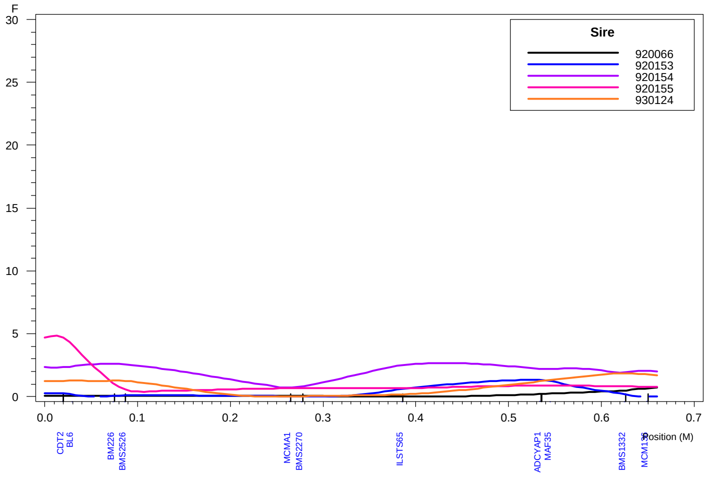

Haley-Knott QTL Analysis: Chromosome 23
LSITRI

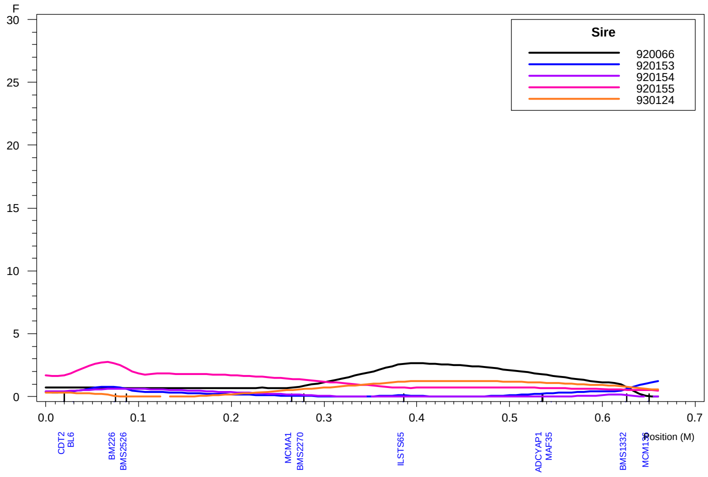

Haley-Knott QTL Analysis: Chromosome 23
LAOST

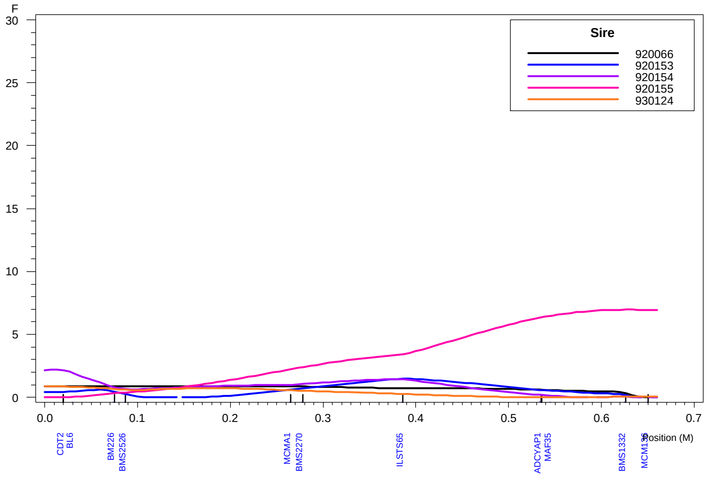

Haley-Knott QTL Analysis: Chromosome 23
LATRICH

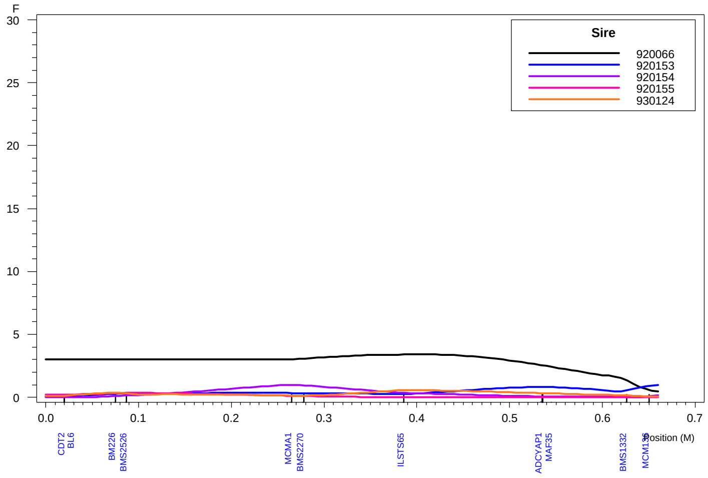

Haley-Knott QTL Analysis: Chromosome 23
DAG0

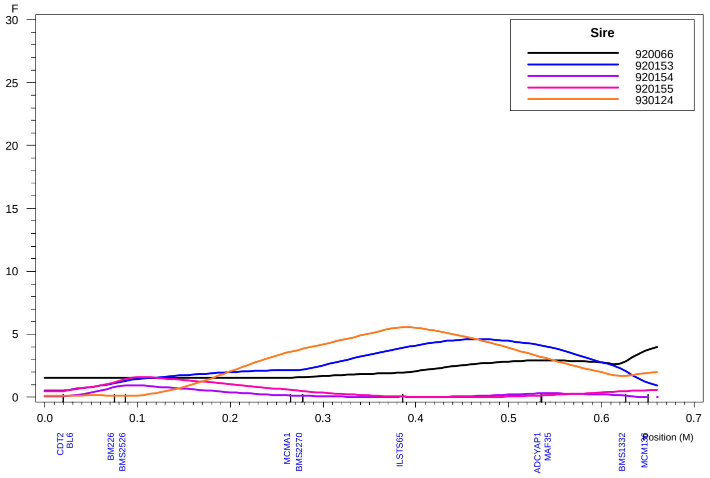

Haley-Knott QTL Analysis: Chromosome 23
DAG1

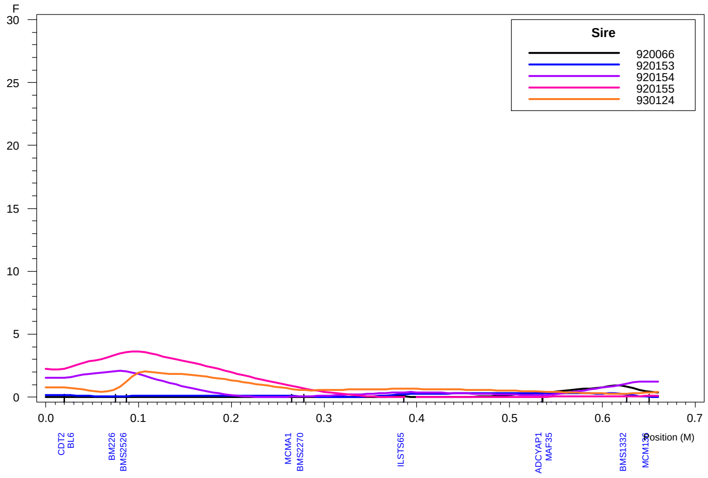

Haley-Knott QTL Analysis: Chromosome 23
DAG2

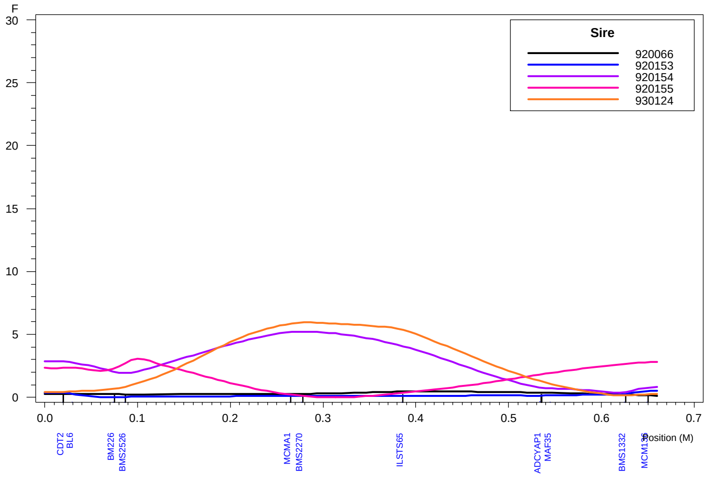

Haley-Knott QTL Analysis: Chromosome 23
ELISA1

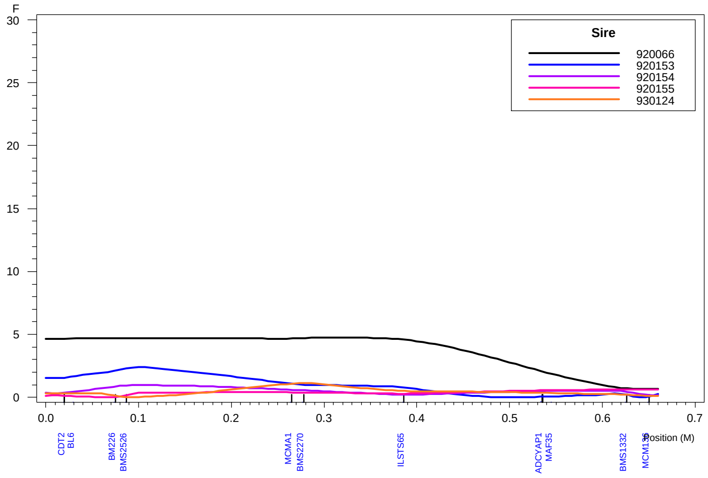

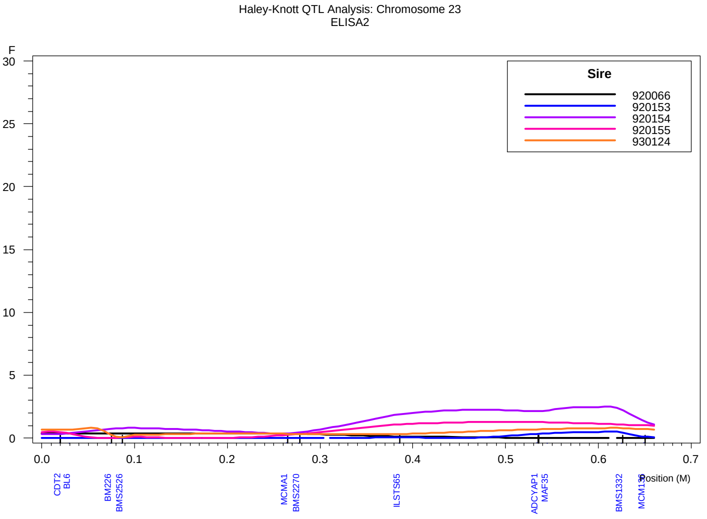

Haley-Knott QTL Analysis: Chromosome 23
ELISA2
F
Sire
920066
920153
920154
920155
930124
Position (M)
CDT2
BL6
BM226
BMS2526
MCMA1
BMS2270
ILSTS65
ADCYAP1
MAF35
BMS1332

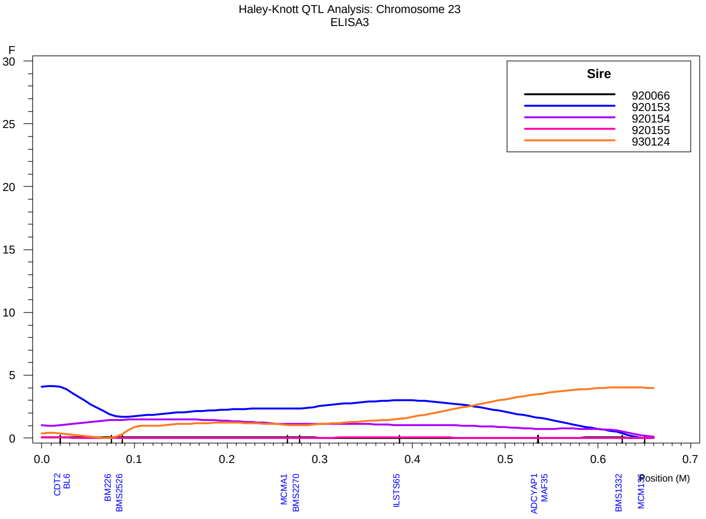

Haley-Knott QTL Analysis: Chromosome 23
ELISA3
F
Sire
920066
920153
920154
920155
930124
Position (M)
CDT2
BL6
BM226
BMS2526
MCMA1
BMS2270
ILSTS65
ADCYAP1
MAF35
BMS1332

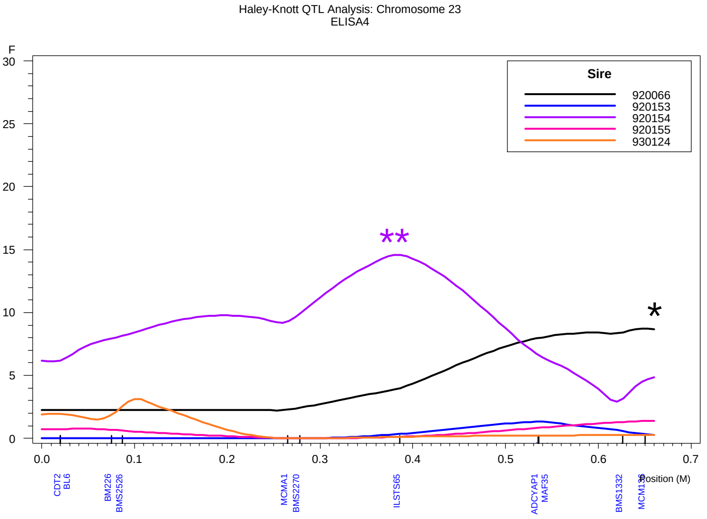

Haley-Knott QTL Analysis: Chromosome 23
ELISA4
F
Sire
920066
920153
920154
920155
930124
**
*
Position (M)
CDT2
BL6
BM226
BMS2526
MCMA1
BMS2270
ILSTS65
ADCYAP1
MAF35
BMS1332

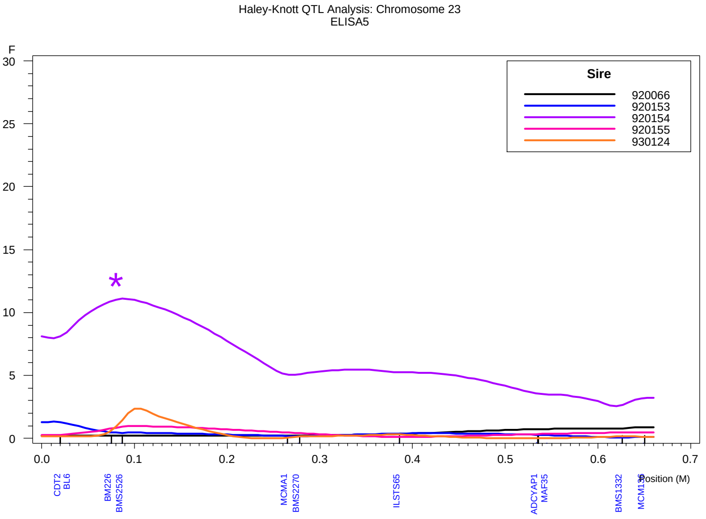

Haley-Knott QTL Analysis: Chromosome 23
ELISA5
F
Sire
920066
920153
920154
920155
930124
*
Position (M)
CDT2
BL6
BM226
BMS2526
MCMA1
BMS2270
ILSTS65
ADCYAP1
MAF35
BMS1332

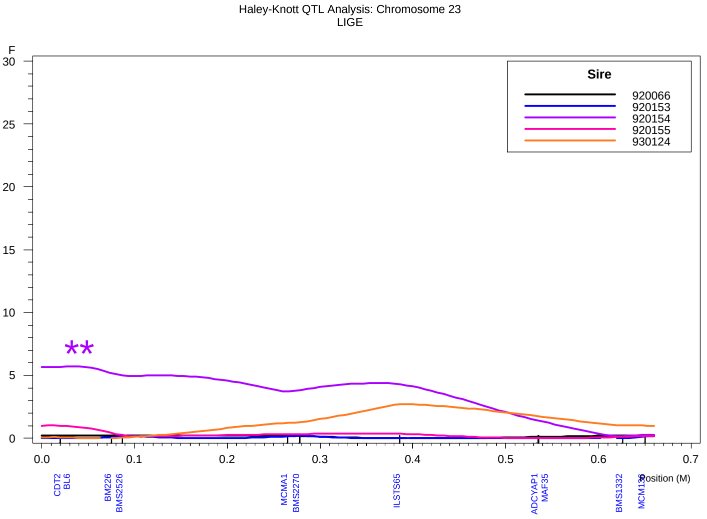

Haley-Knott QTL Analysis: Chromosome 23
LIGE
F
Sire
920066
920153
920154
920155
930124
**
Position (M)
CDT2
BL6
BM226
BMS2526
MCMA1
BMS2270
ILSTS65
ADCYAP1
MAF35
BMS1332

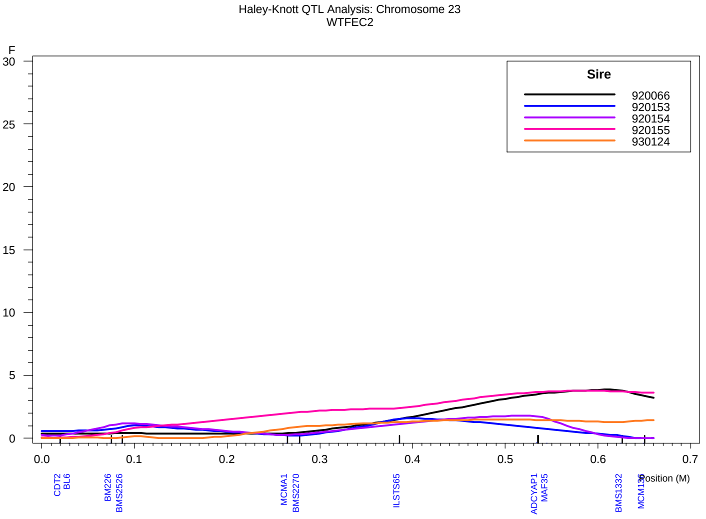

Haley-Knott QTL Analysis: Chromosome 23
WTFEC2
F
Sire
920066
920153
920154
920155
930124
Position (M)
CDT2
BL6
BM226
BMS2526
MCMA1
BMS2270
ILSTS65
ADCYAP1
MAF35
BMS1332
