# Supplementary material for: Discovery of quantitative trait loci for resistance to parasitic nematode infection in sheep: I. Analysis of outcross pedigrees
Source: BMC Genomics. 2006 Jul 18;7:178. doi: 10.1186/1471-2164-7-178 (PMC1574317; doi:10.1186/1471-2164-7-178)

Linkage Analysis in the Parasite Outcross Flock: Chromosome 24

Information Content: Chromosome 24

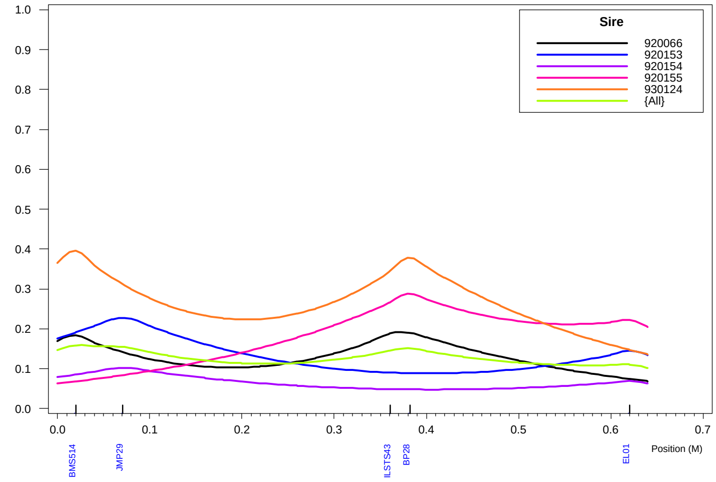

Haley-Knott QTL Analysis: Chromosome 24

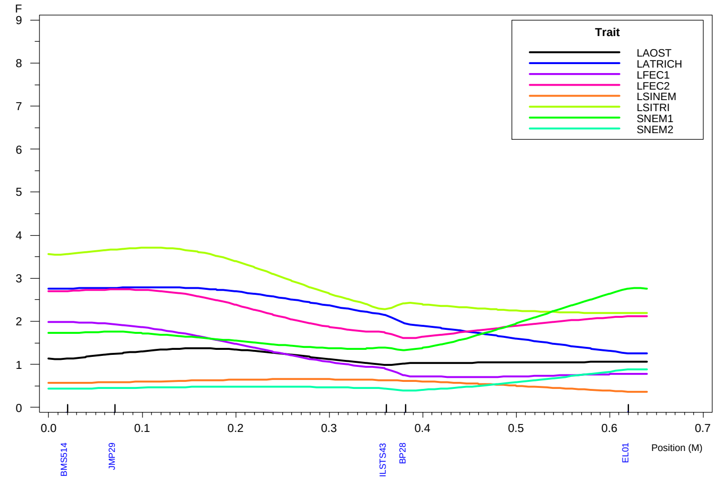

Haley-Knott QTL Analysis: Chromosome 24

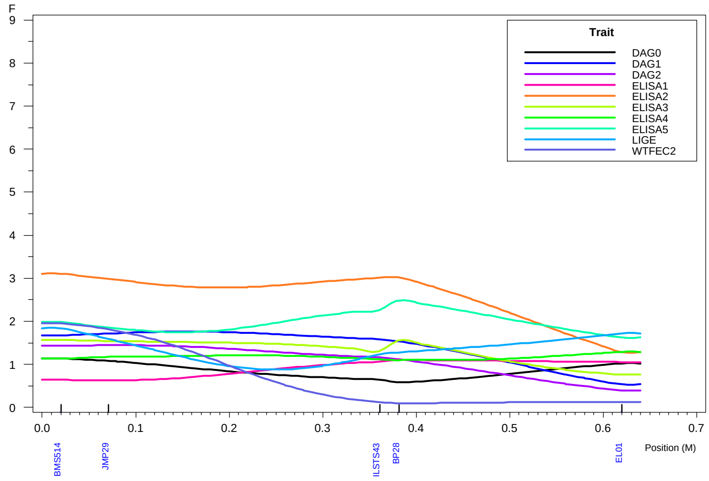

Haley-Knott QTL Analysis: Chromosome 24
LFEC1

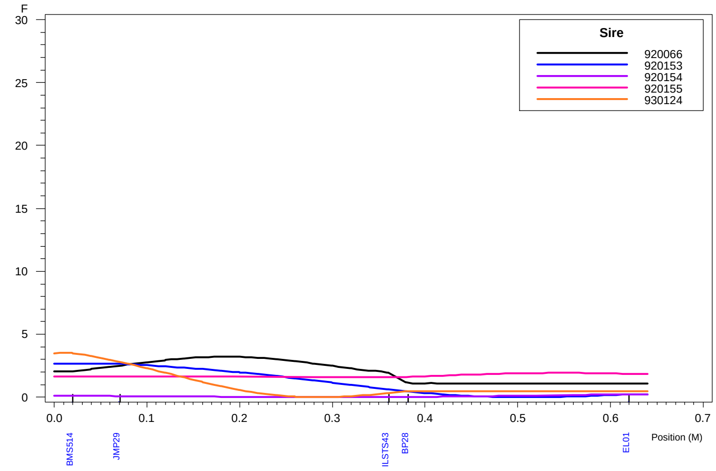

Haley-Knott QTL Analysis: Chromosome 24
LFEC2

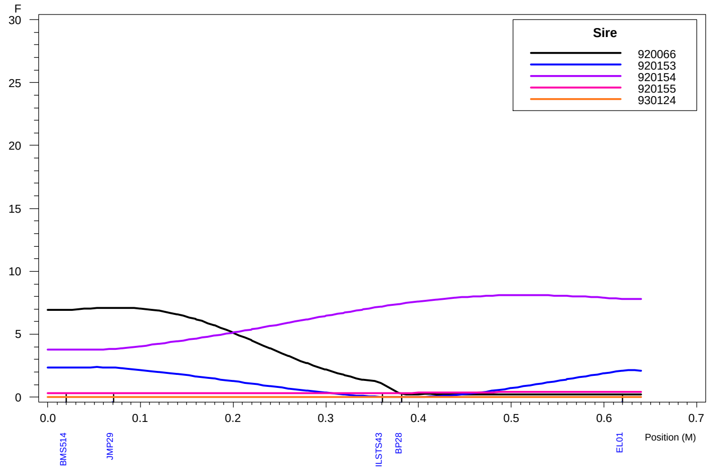

Haley-Knott QTL Analysis: Chromosome 24
SNEM1

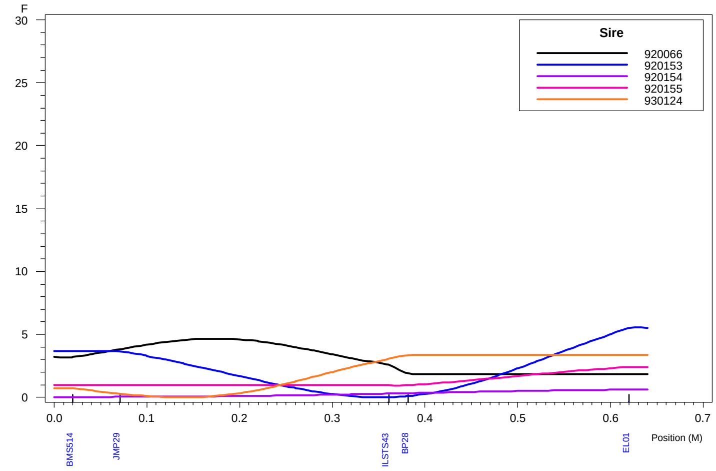

Haley-Knott QTL Analysis: Chromosome 24
SNEM2

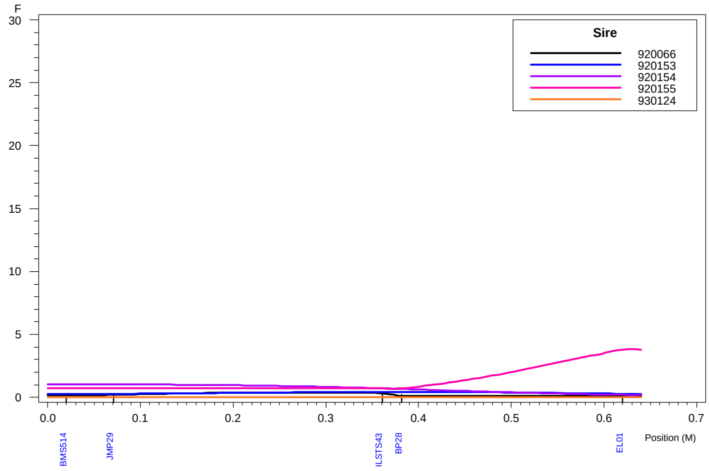

Haley-Knott QTL Analysis: Chromosome 24
LSINEM

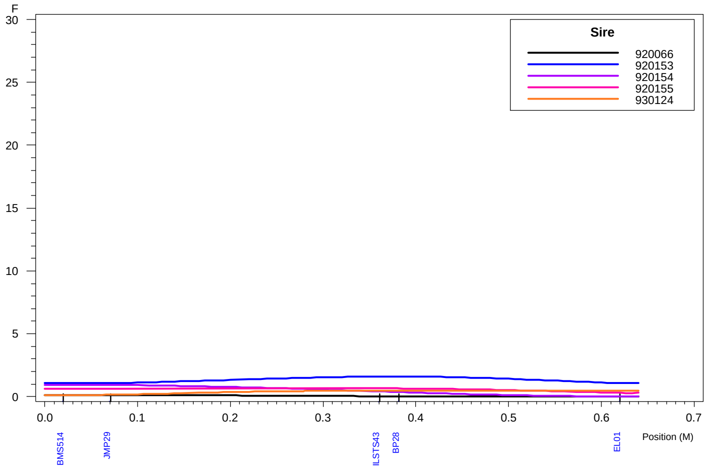

Haley-Knott QTL Analysis: Chromosome 24
LSITRI

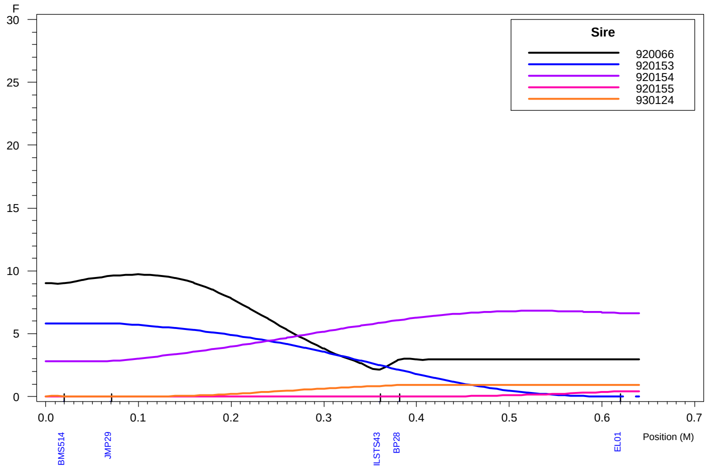

Haley-Knott QTL Analysis: Chromosome 24
LAOST

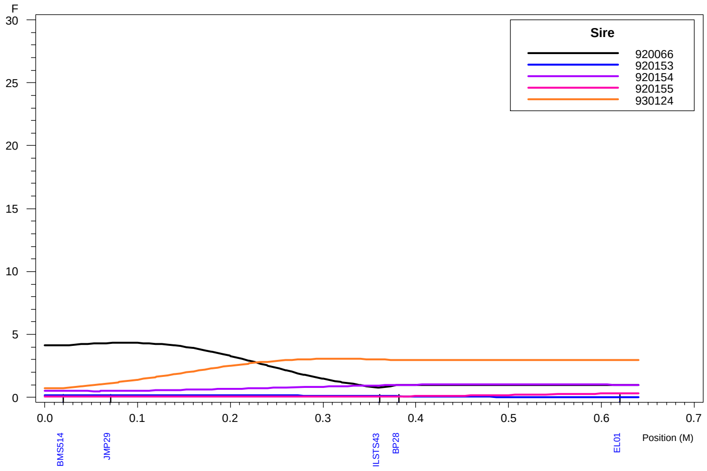

Haley-Knott QTL Analysis: Chromosome 24
LATRICH

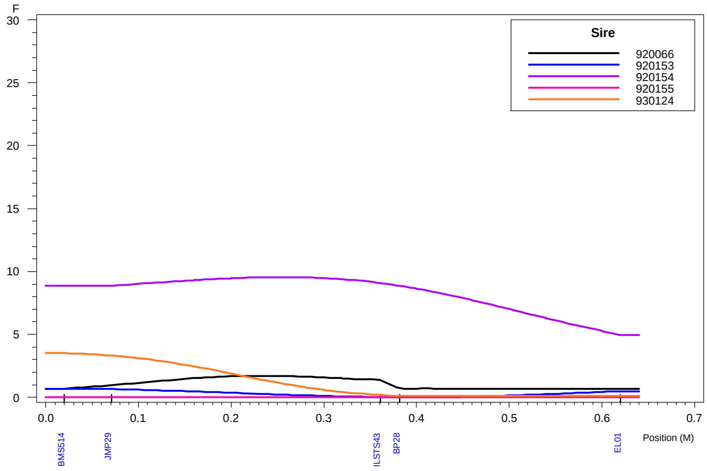

Haley-Knott QTL Analysis: Chromosome 24
DAG0

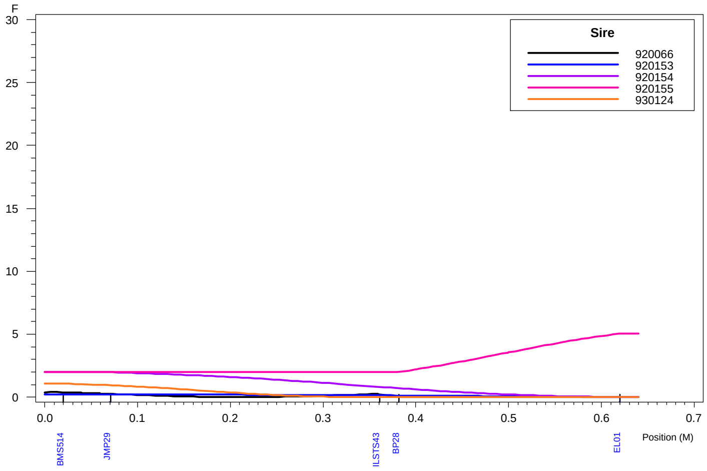

Haley-Knott QTL Analysis: Chromosome 24
DAG1

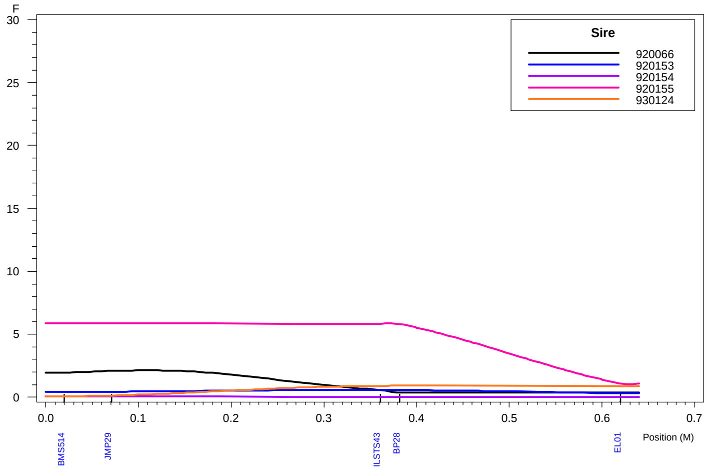

Haley-Knott QTL Analysis: Chromosome 24
DAG2

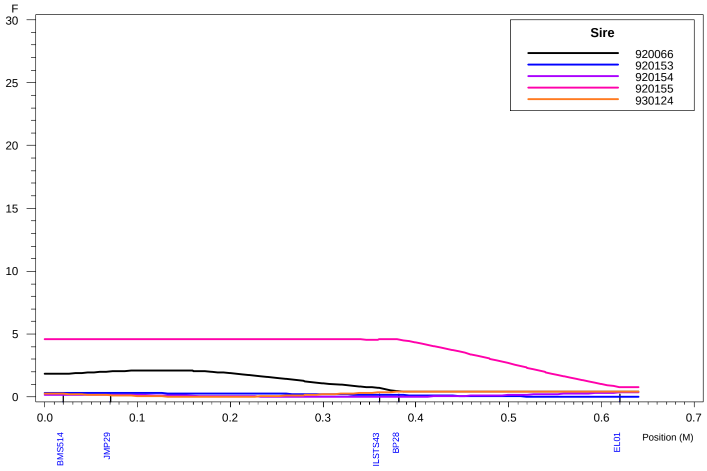

Haley-Knott QTL Analysis: Chromosome 24
ELISA1

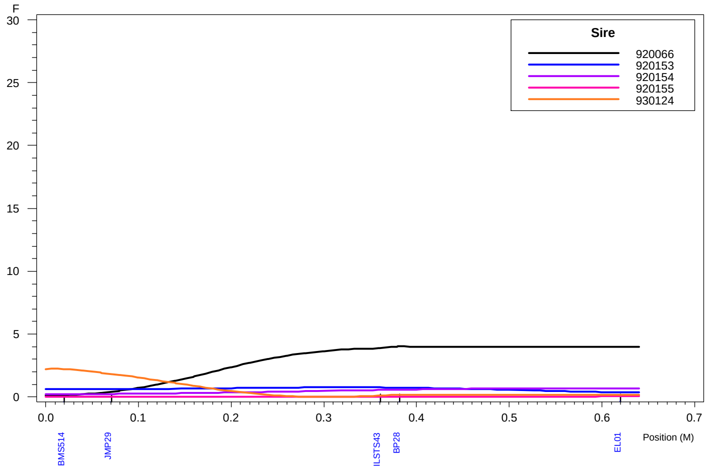

Haley-Knott QTL Analysis: Chromosome 24
ELISA2

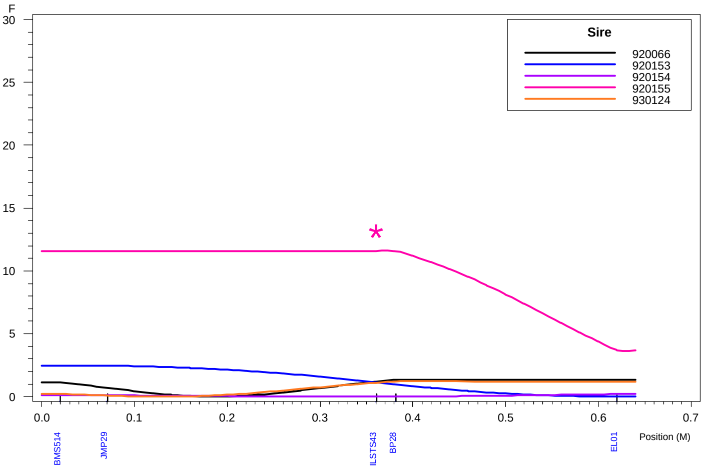

Haley-Knott QTL Analysis: Chromosome 24
ELISA3

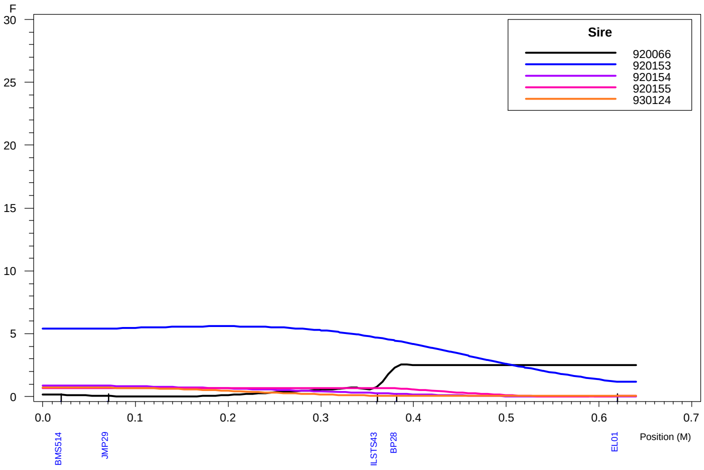

Haley-Knott QTL Analysis: Chromosome 24
ELISA4

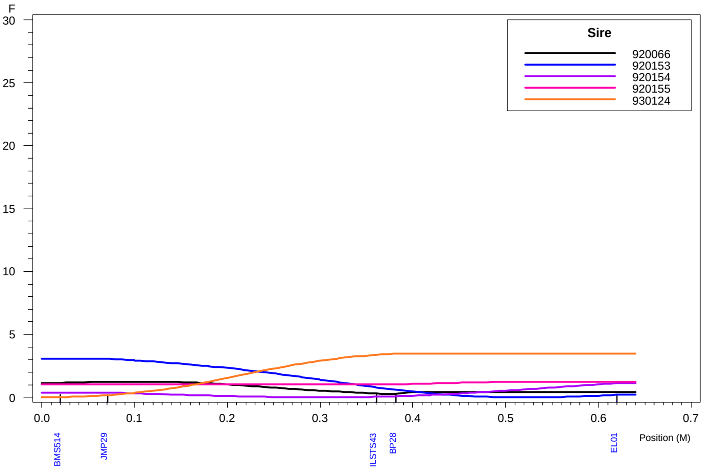

Haley-Knott QTL Analysis: Chromosome 24
ELISA5

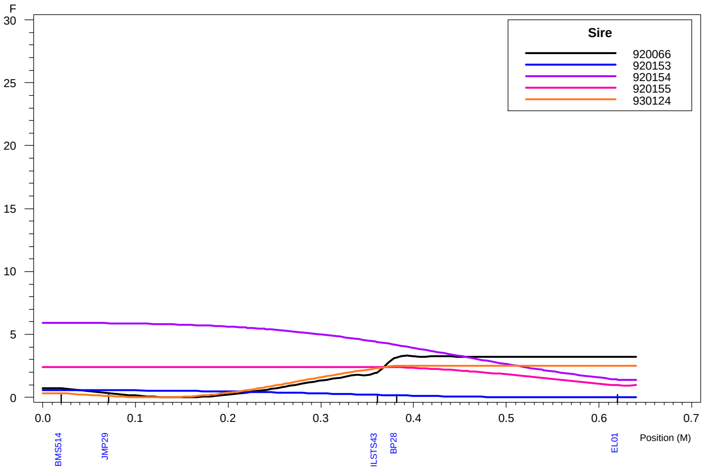

Haley-Knott QTL Analysis: Chromosome 24
LIGE

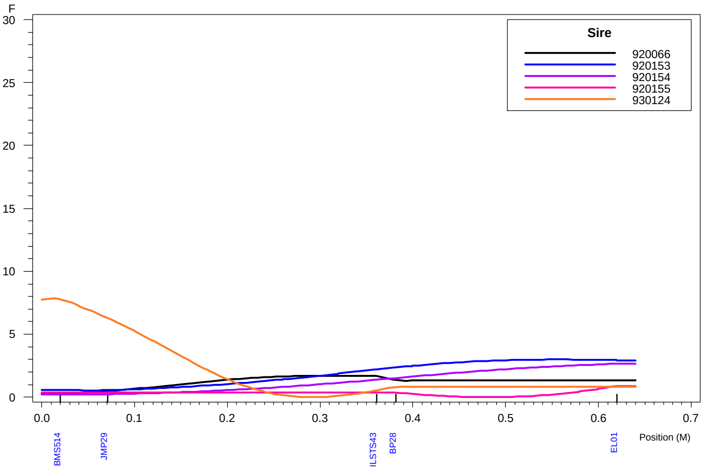

Haley-Knott QTL Analysis: Chromosome 24
WTFEC2

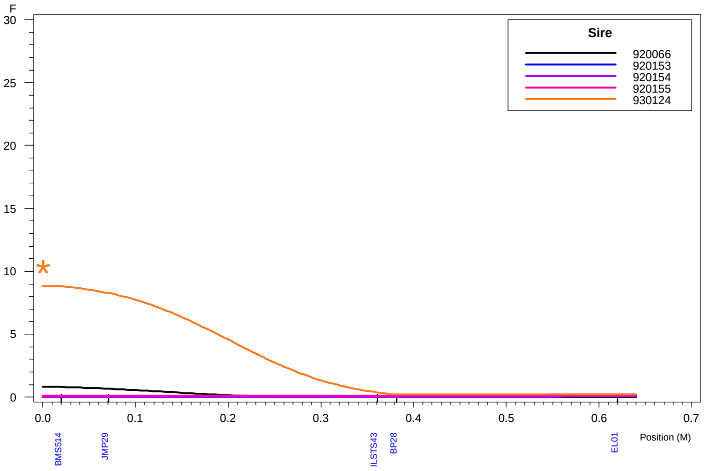

Supplement: Additional File 29 — Chr 24. Haley Knott linkage analysis of sheep chromosome 24. [file 1471-2164-7-178-S29.pdf]
