# Supplementary material for: Discovery of quantitative trait loci for resistance to parasitic nematode infection in sheep: I. Analysis of outcross pedigrees
Source: BMC Genomics. 2006 Jul 18;7:178. doi: 10.1186/1471-2164-7-178 (PMC1574317; doi:10.1186/1471-2164-7-178)
Supplement: Additional File 30 — Chr 25. Haley Knott linkage analysis of sheep chromosome 25. [file 1471-2164-7-178-S30.pdf]

Linkage Analysis in the Parasite Outcross Flock: Chromosome 25

Information Content: Chromosome 25

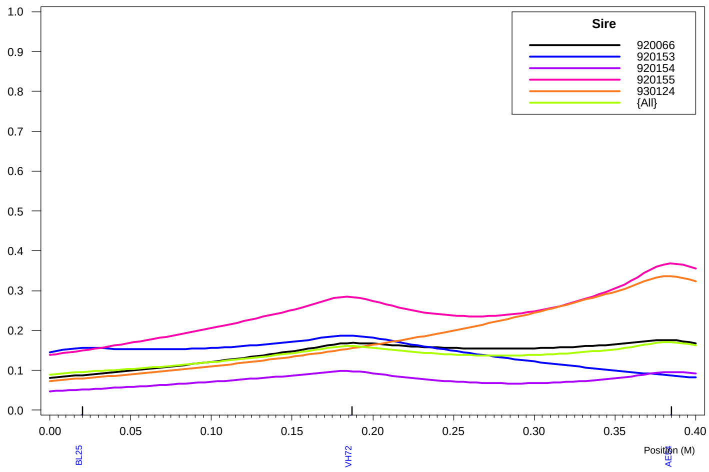

Haley-Knott QTL Analysis: Chromosome 25

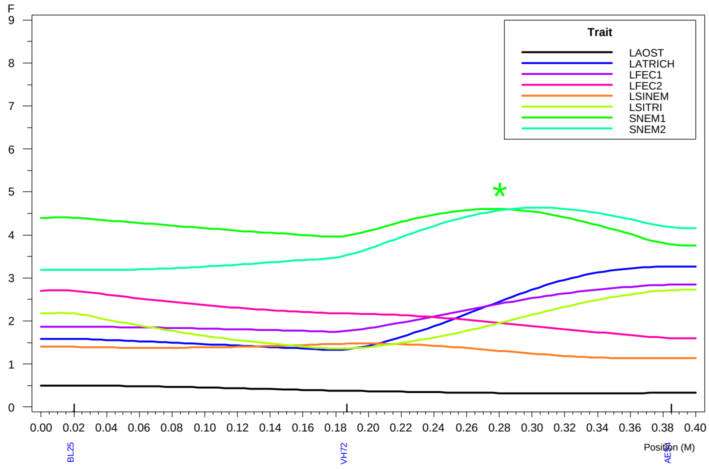

Haley-Knott QTL Analysis: Chromosome 25

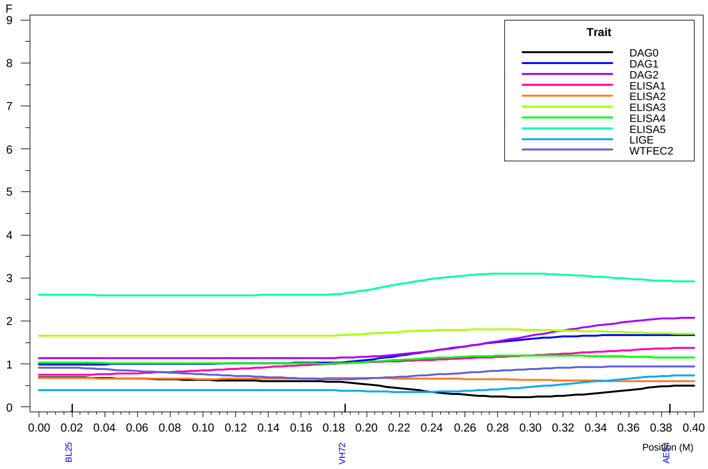

Haley-Knott QTL Analysis: Chromosome 25
LFEC1

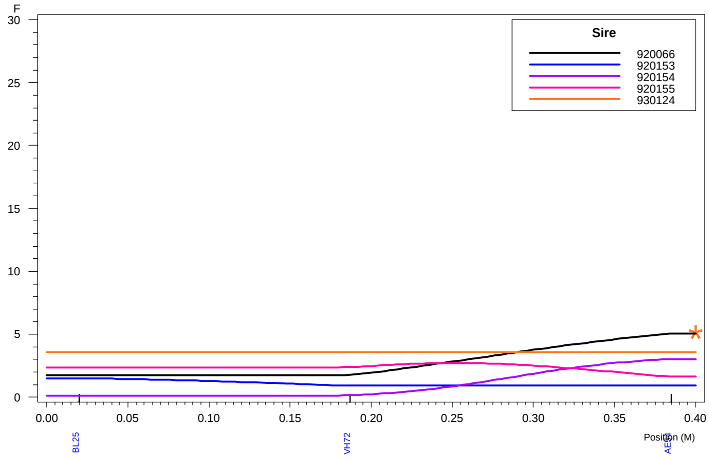

Haley-Knott QTL Analysis: Chromosome 25
LFEC2

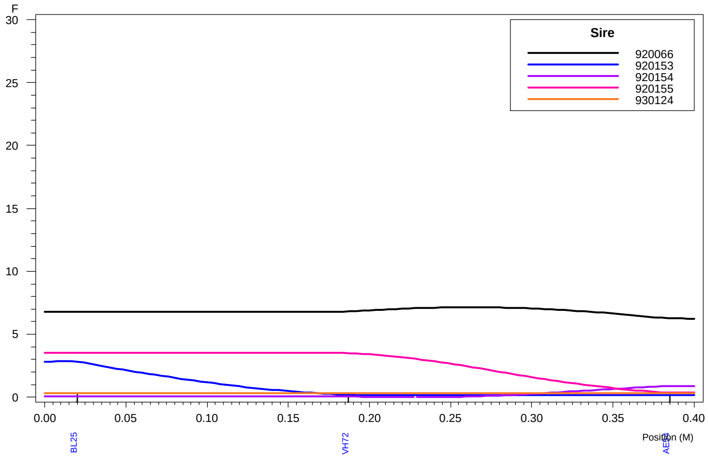

Haley-Knott QTL Analysis: Chromosome 25
SNEM1

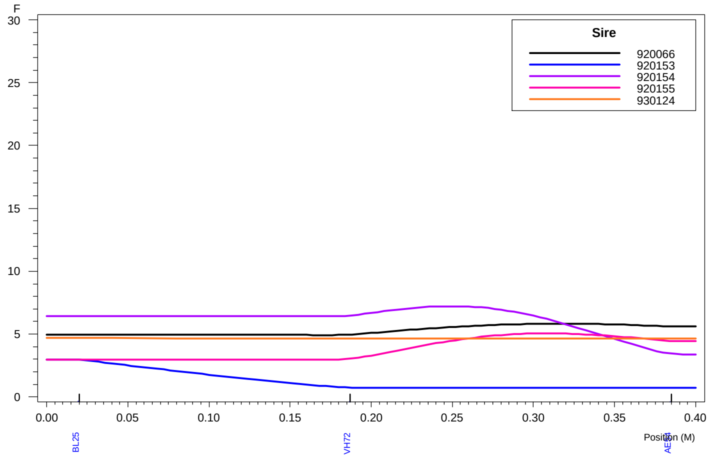

Haley-Knott QTL Analysis: Chromosome 25
SNEM2

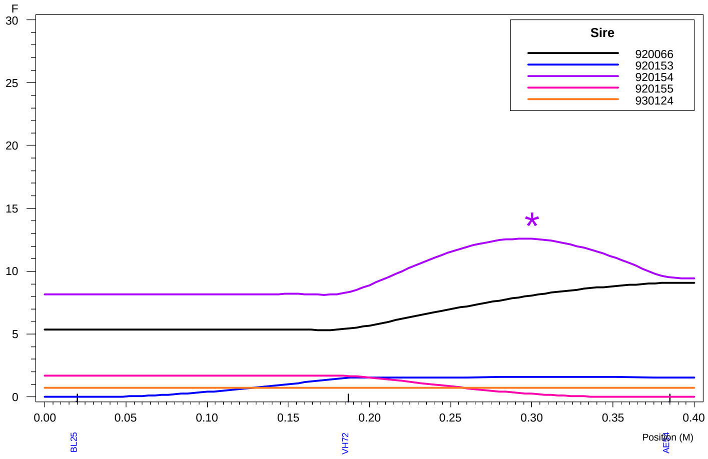

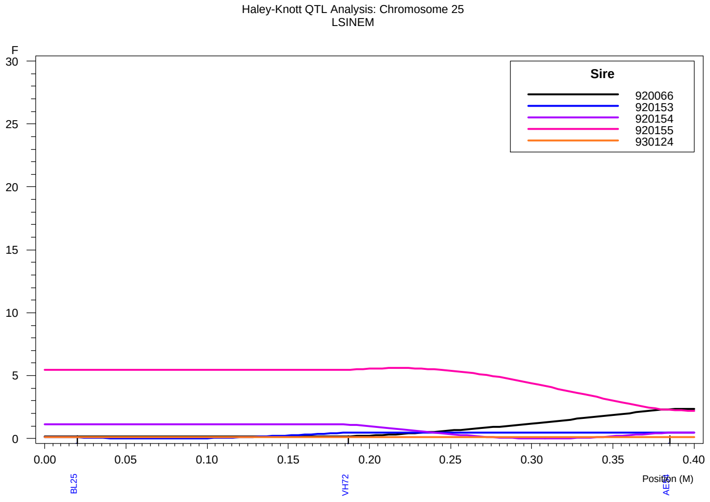
Haley-Knott QTL Analysis: Chromosome 25
LSINEM
F
Sire
920066
920153
920154
920155
930124
Position (M)
BL25
VH72

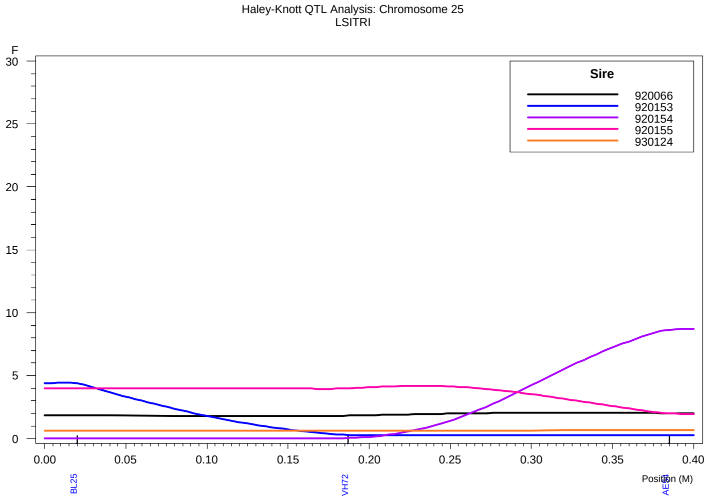
Haley-Knott QTL Analysis: Chromosome 25
LSITRI
F
Sire
920066
920153
920154
920155
930124
Position (M)
BL25
VH72

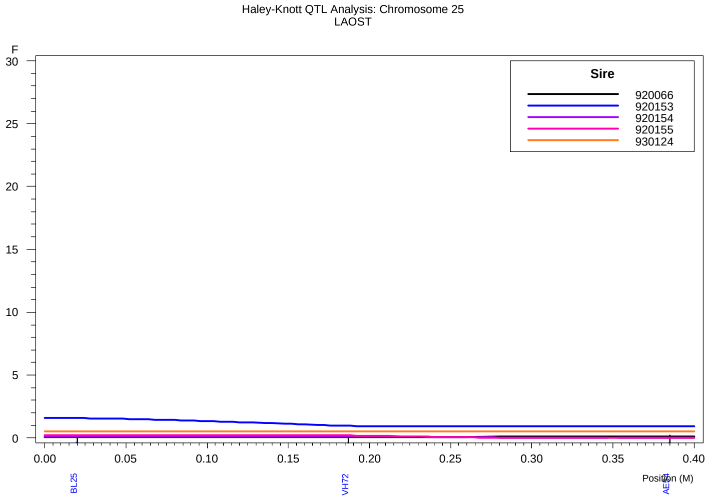
Haley-Knott QTL Analysis: Chromosome 25
LAOST
F
Sire
920066
920153
920154
920155
930124
Position (M)
BL25
VH72

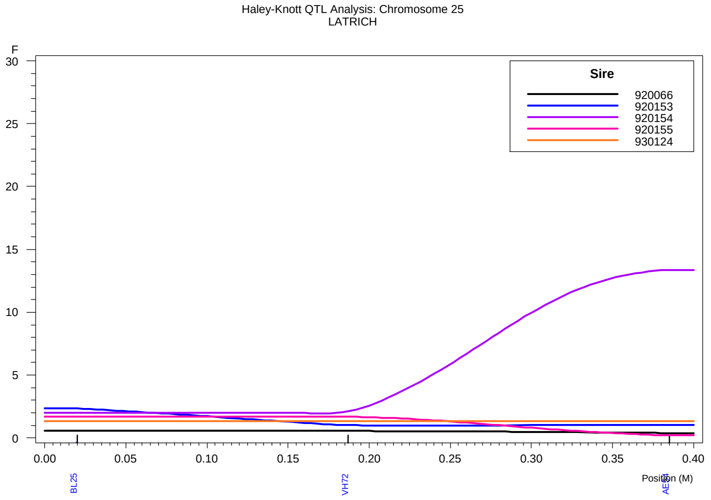
Haley-Knott QTL Analysis: Chromosome 25
LATRICH
F
Sire
920066
920153
920154
920155
930124
Position (M)
BL25
VH72

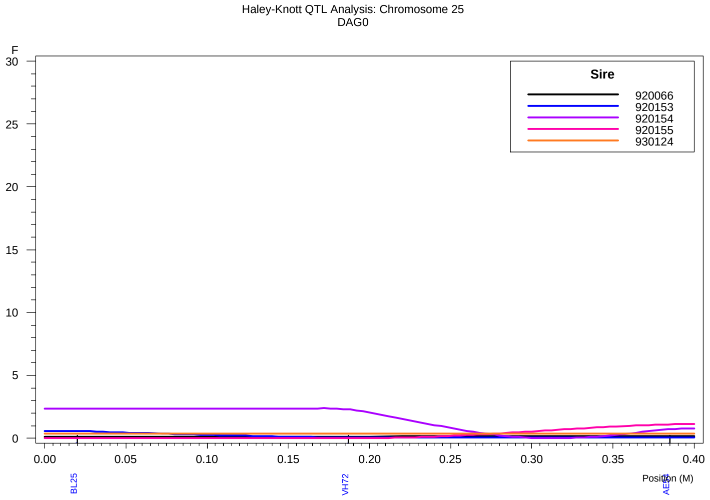
Haley-Knott QTL Analysis: Chromosome 25
DAG0
F
Sire
920066
920153
920154
920155
930124
Position (M)
BL25
VH72

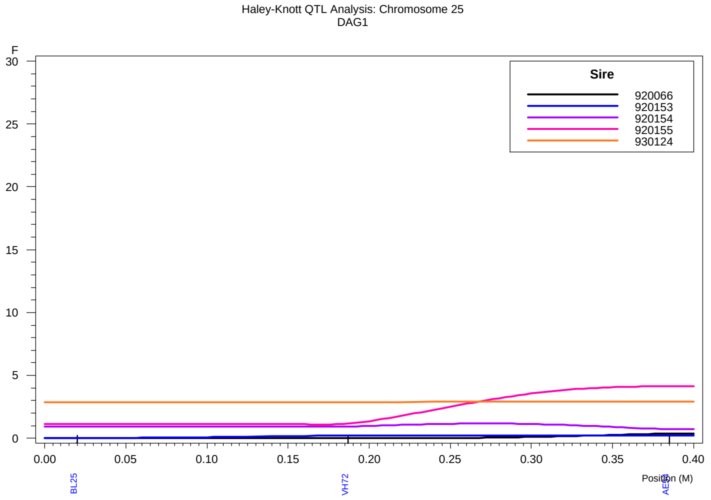
Haley-Knott QTL Analysis: Chromosome 25
DAG1
F
Sire
920066
920153
920154
920155
930124
Position (M)
BL25
VH72

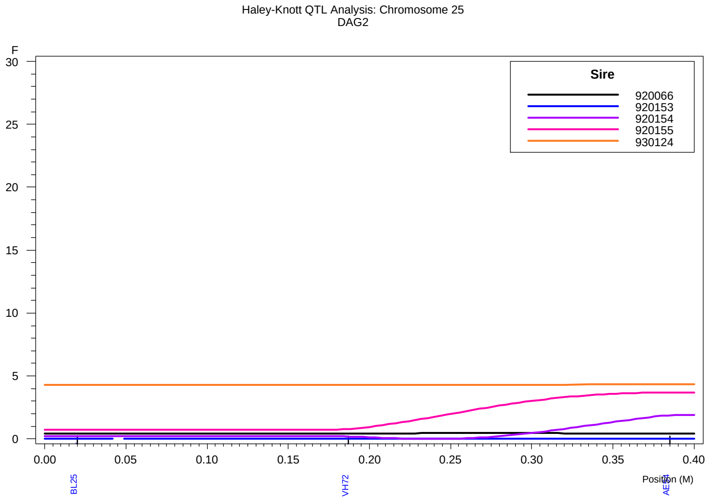
Haley-Knott QTL Analysis: Chromosome 25
DAG2
F
Sire
920066
920153
920154
920155
930124
Position (M)
BL25
VH72

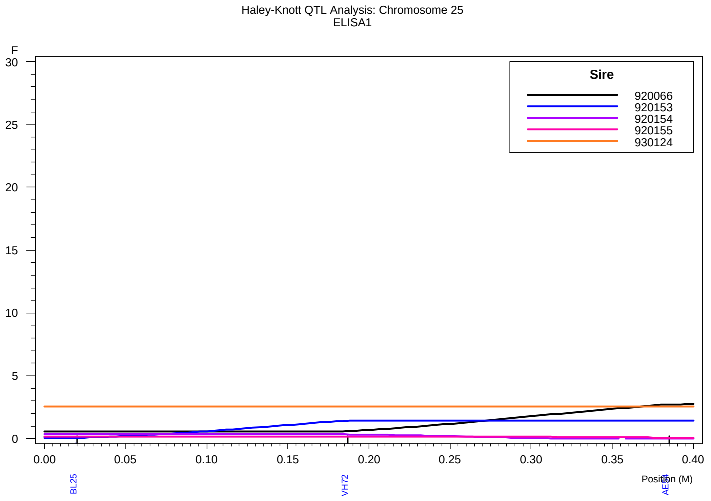
Haley-Knott QTL Analysis: Chromosome 25
ELISA1
F
Sire
920066
920153
920154
920155
930124
Position (M)
BL25
VH72

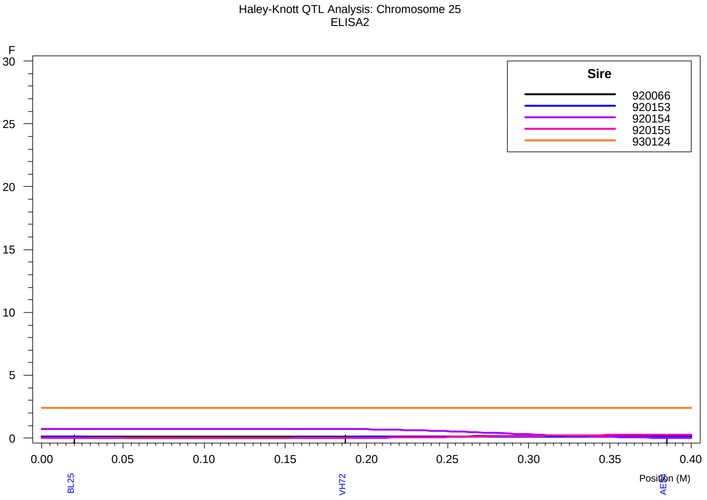
Haley-Knott QTL Analysis: Chromosome 25
ELISA2
F
Sire
920066
920153
920154
920155
930124
Position (M)
BL25
VH72
AE

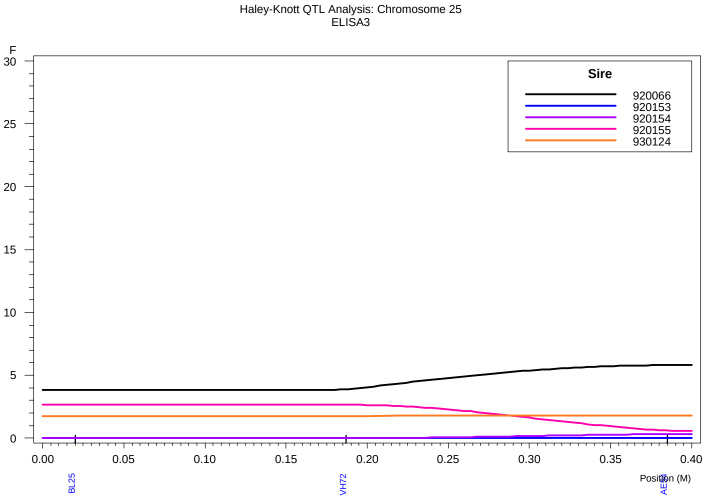
Haley-Knott QTL Analysis: Chromosome 25
ELISA3
F
Sire
920066
920153
920154
920155
930124
Position (M)
BL25
VH72
AE

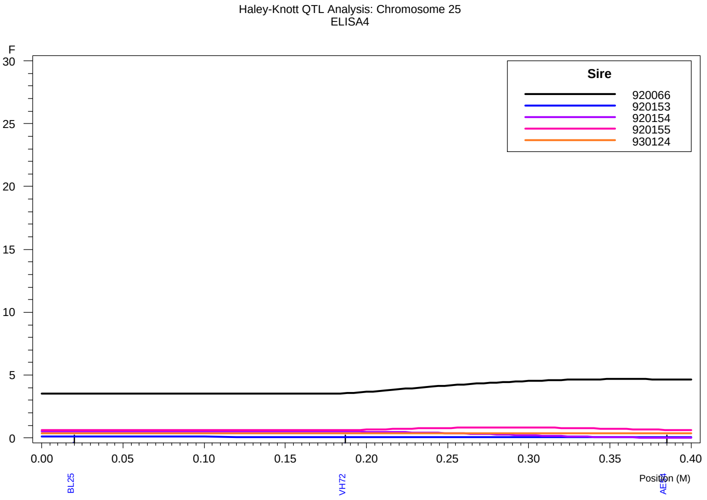
Haley-Knott QTL Analysis: Chromosome 25
ELISA4
F
Sire
920066
920153
920154
920155
930124
Position (M)
BL25
VH72
AE

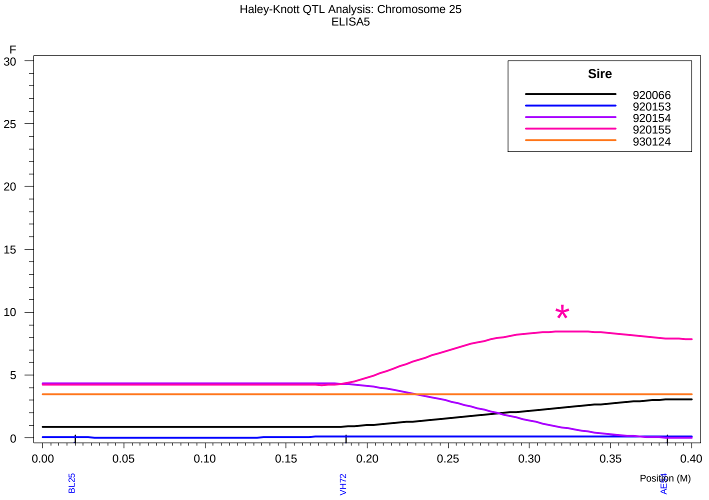
Haley-Knott QTL Analysis: Chromosome 25
ELISA5
F
Sire
920066
920153
920154
920155
930124
*
Position (M)
BL25
VH72
AE

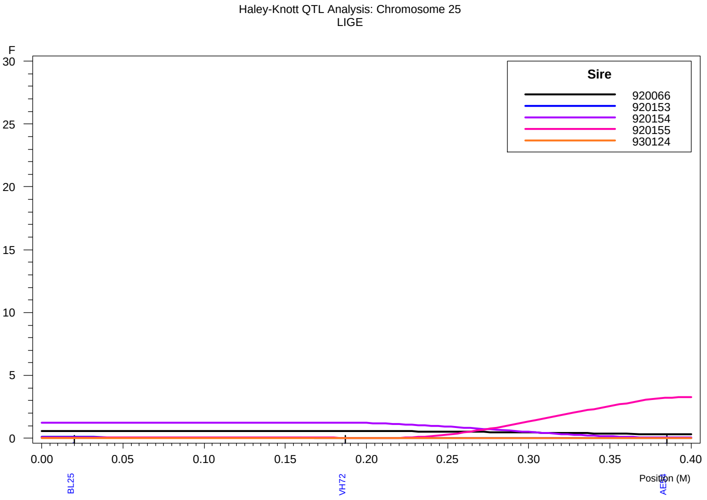
Haley-Knott QTL Analysis: Chromosome 25
LIGE
F
Sire
920066
920153
920154
920155
930124
Position (M)
BL25
VH72
AE

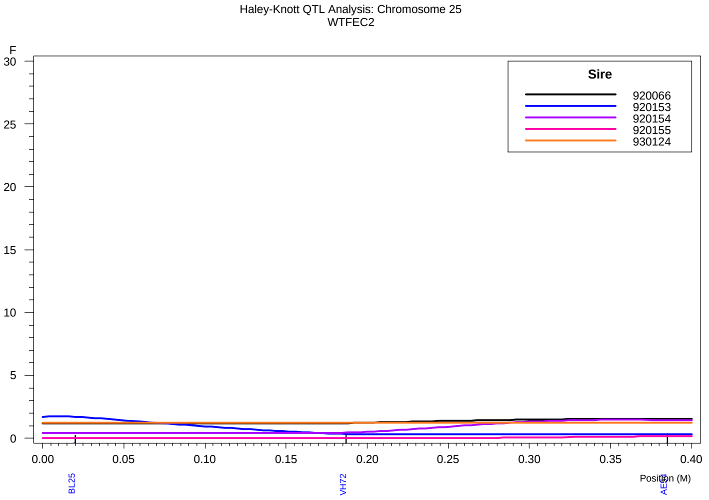
Haley-Knott QTL Analysis: Chromosome 25
WTFEC2
F
Sire
920066
920153
920154
920155
930124
Position (M)
BL25
VH72
AE
