# Supplementary material for: Discovery of quantitative trait loci for resistance to parasitic nematode infection in sheep: I. Analysis of outcross pedigrees
Source: BMC Genomics. 2006 Jul 18;7:178. doi: 10.1186/1471-2164-7-178 (PMC1574317; doi:10.1186/1471-2164-7-178)
Supplement: Additional File 31 — Chr 26. Haley Knott linkage analysis of sheep chromosome 26. [file 1471-2164-7-178-S31.pdf]

Linkage Analysis in the Parasite Outcross Flock: Chromosome 26

Information Content: Chromosome 26

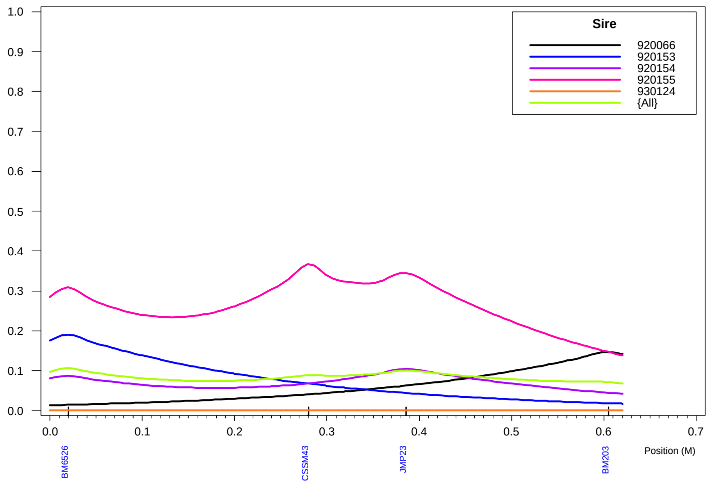

Haley-Knott QTL Analysis: Chromosome 26

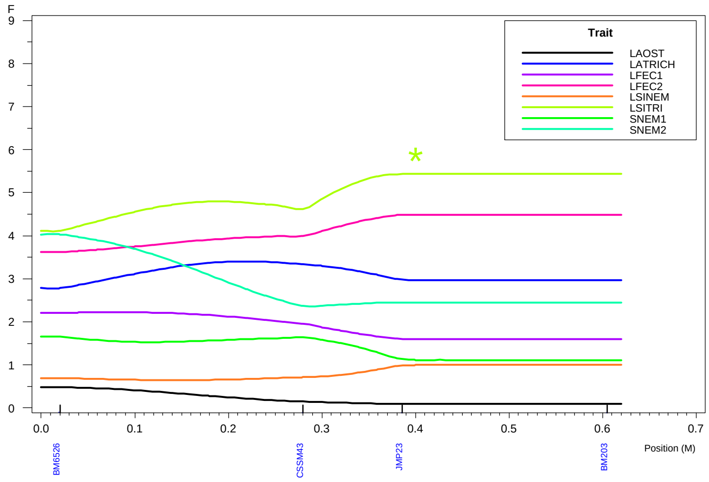

Haley-Knott QTL Analysis: Chromosome 26

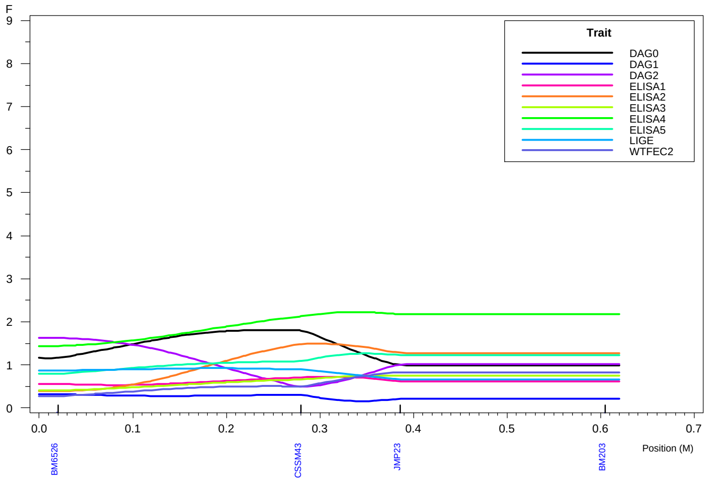

Haley-Knott QTL Analysis: Chromosome 26
LFEC1

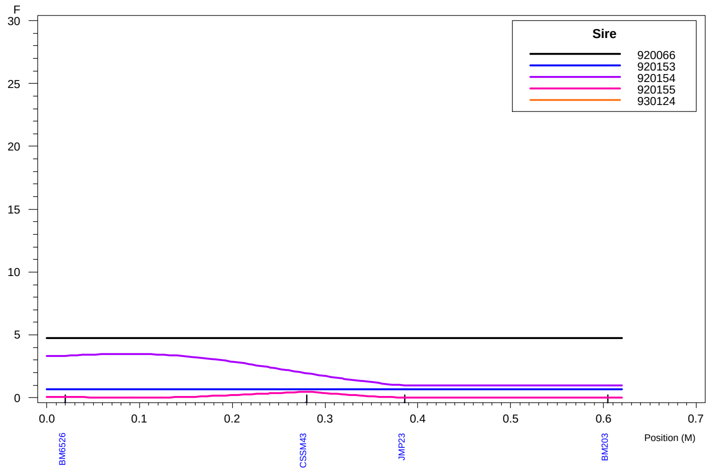

Haley-Knott QTL Analysis: Chromosome 26
LFEC2

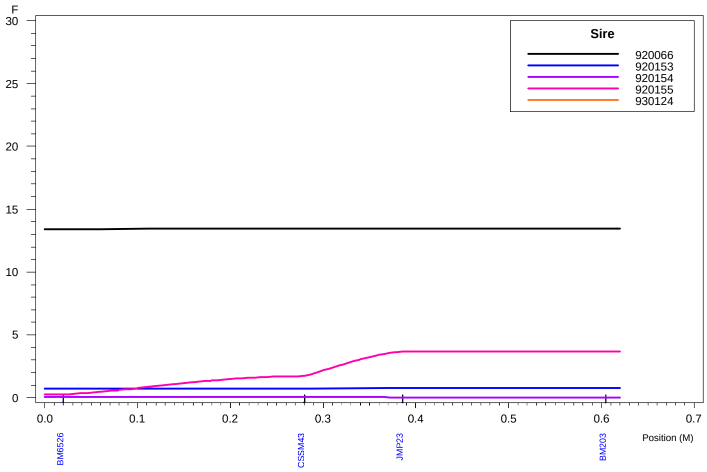

Haley-Knott QTL Analysis: Chromosome 26
SNEM1

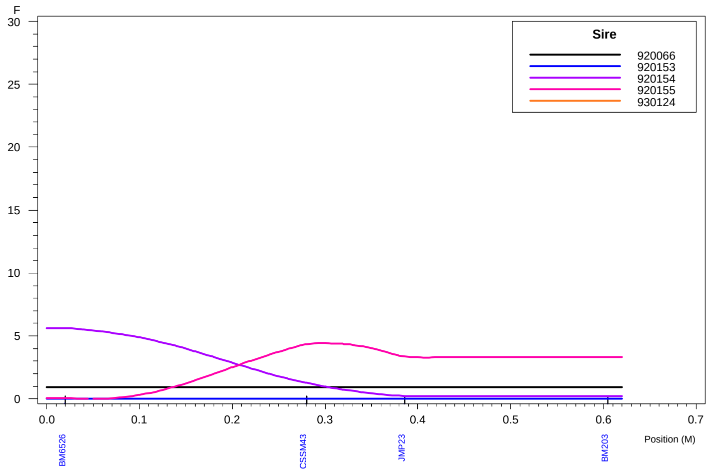

Haley-Knott QTL Analysis: Chromosome 26
SNEM2

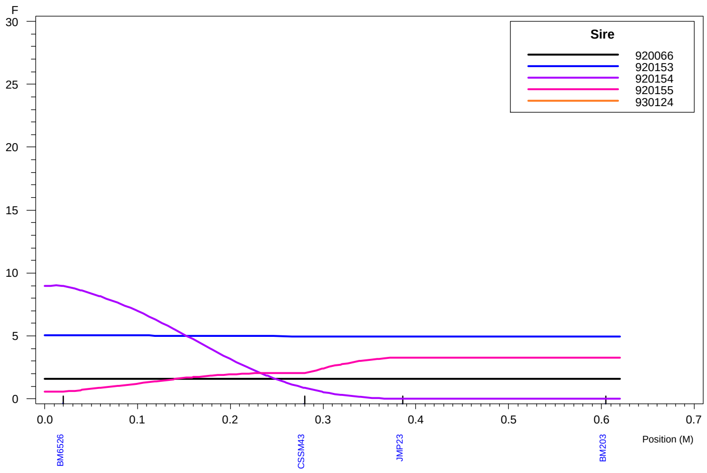

Haley-Knott QTL Analysis: Chromosome 26
LSINEM

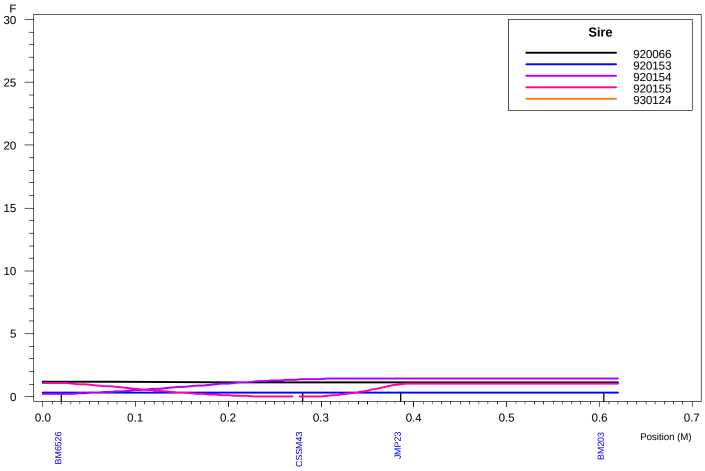

Haley-Knott QTL Analysis: Chromosome 26
LSITRI

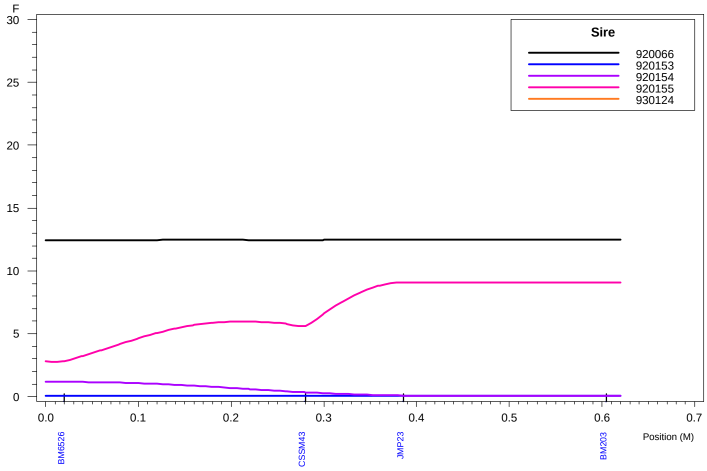

Haley-Knott QTL Analysis: Chromosome 26
LAOST

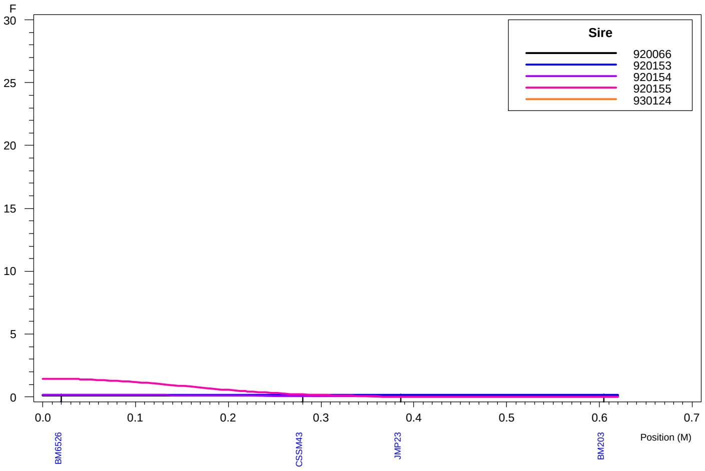

Haley-Knott QTL Analysis: Chromosome 26
LATRICH

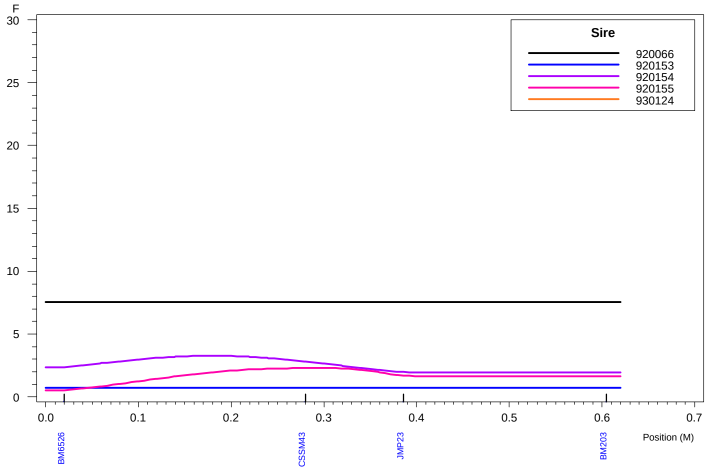

Haley-Knott QTL Analysis: Chromosome 26
DAG0

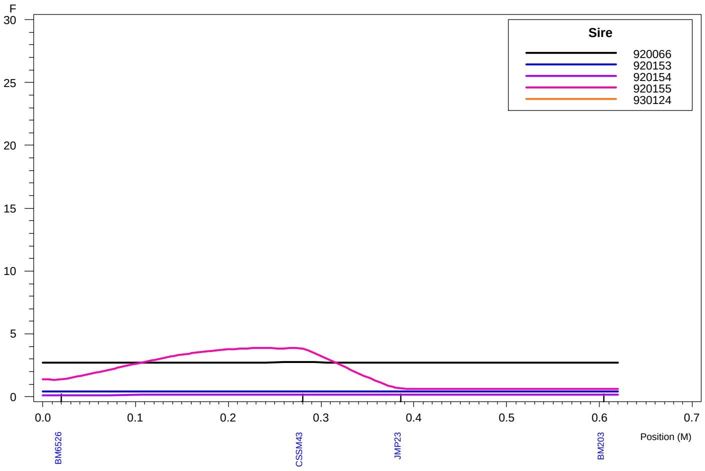

Haley-Knott QTL Analysis: Chromosome 26
DAG1

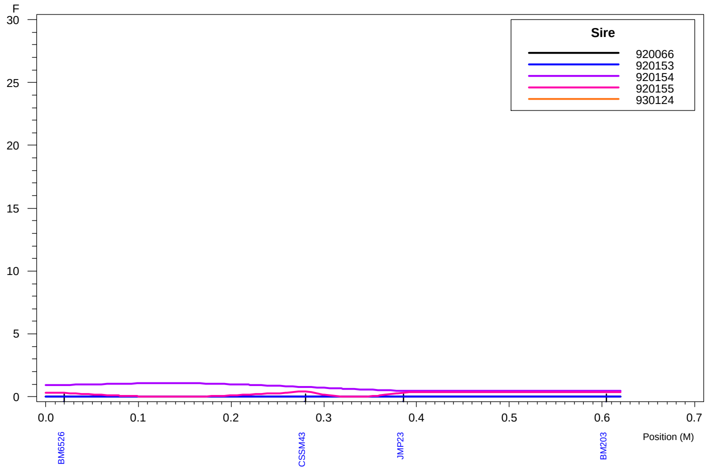

Haley-Knott QTL Analysis: Chromosome 26
DAG2

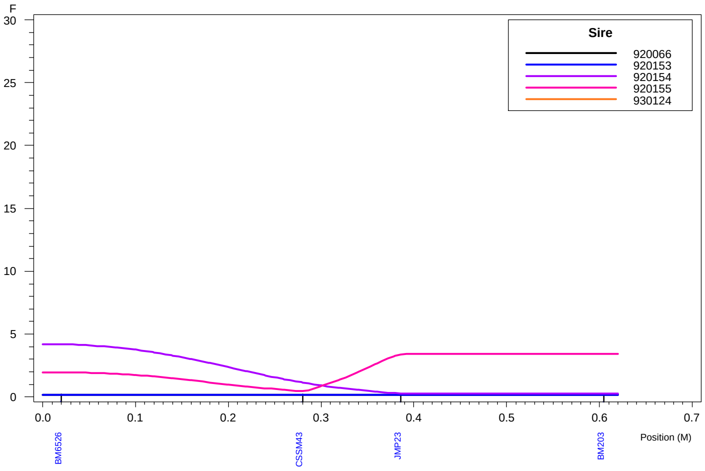

Haley-Knott QTL Analysis: Chromosome 26
ELISA1

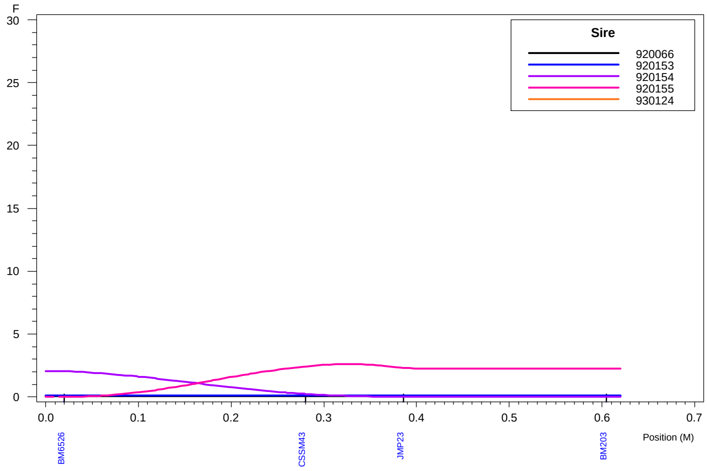

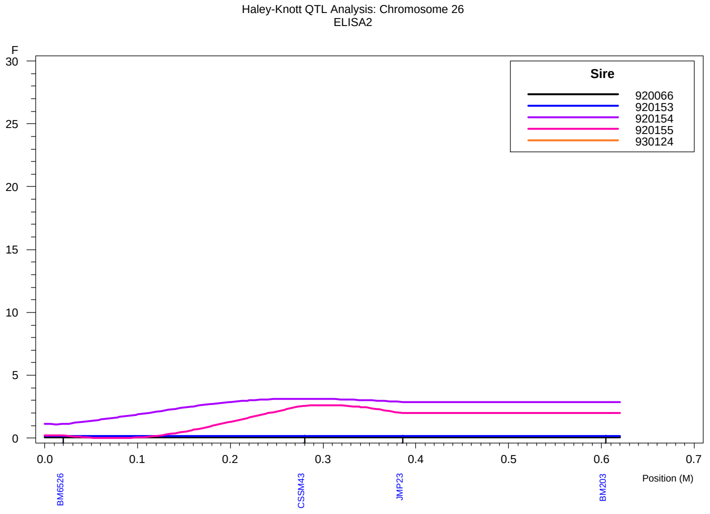
Haley-Knott QTL Analysis: Chromosome 26
ELISA2
F
Sire
920066
920153
920154
920155
930124
Position (M)
BM6526
CSSM43
JMP23
BM203

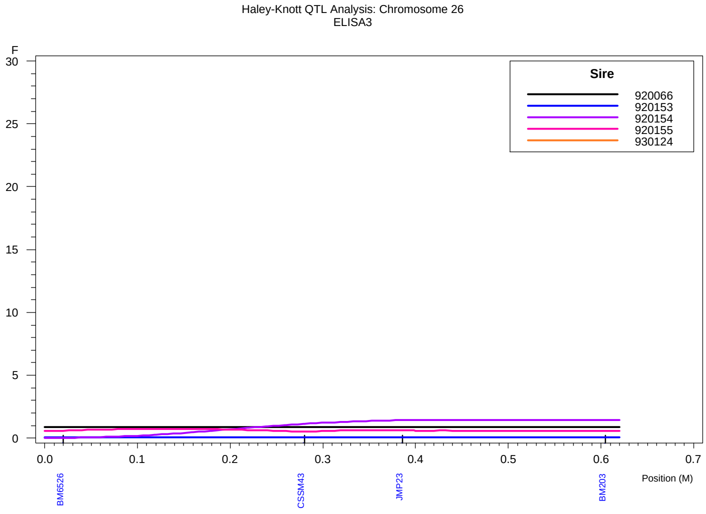
Haley-Knott QTL Analysis: Chromosome 26
ELISA3
F
Sire
920066
920153
920154
920155
930124
Position (M)
BM6526
CSSM43
JMP23
BM203

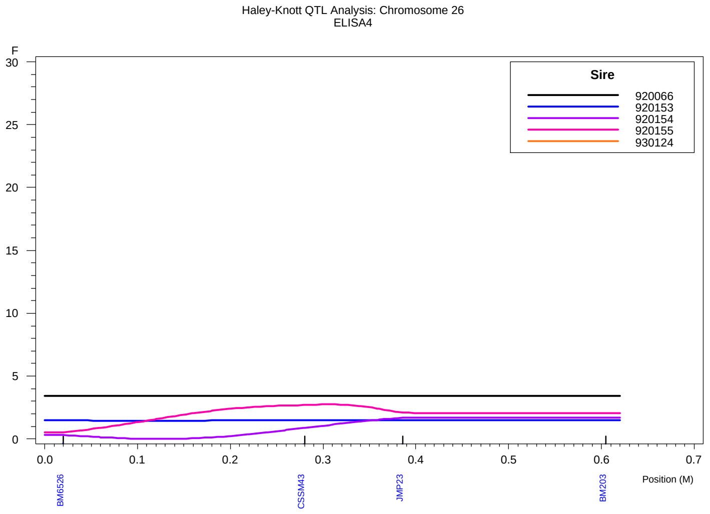
Haley-Knott QTL Analysis: Chromosome 26
ELISA4
F
Sire
920066
920153
920154
920155
930124
Position (M)
BM6526
CSSM43
JMP23
BM203

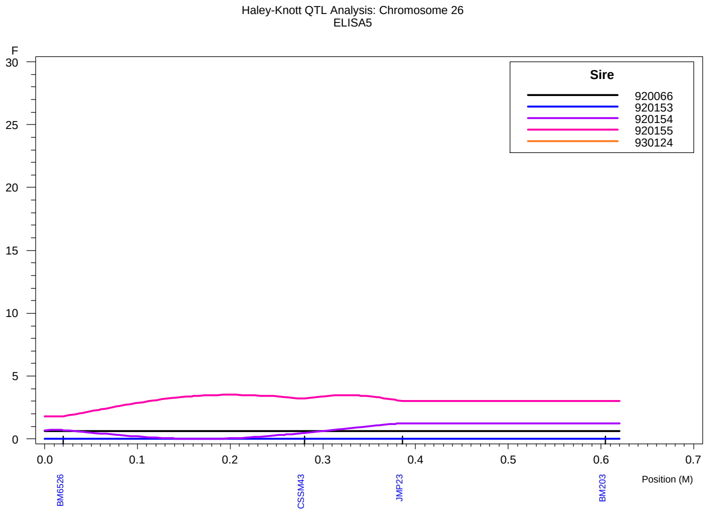
Haley-Knott QTL Analysis: Chromosome 26
ELISA5
F
Sire
920066
920153
920154
920155
930124
Position (M)
BM6526
CSSM43
JMP23
BM203

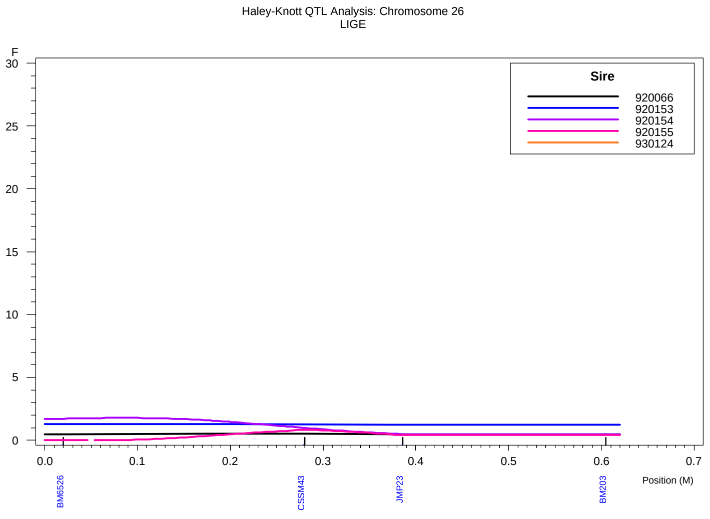
Haley-Knott QTL Analysis: Chromosome 26
LIGE
F
Sire
920066
920153
920154
920155
930124
Position (M)
BM6526
CSSM43
JMP23
BM203

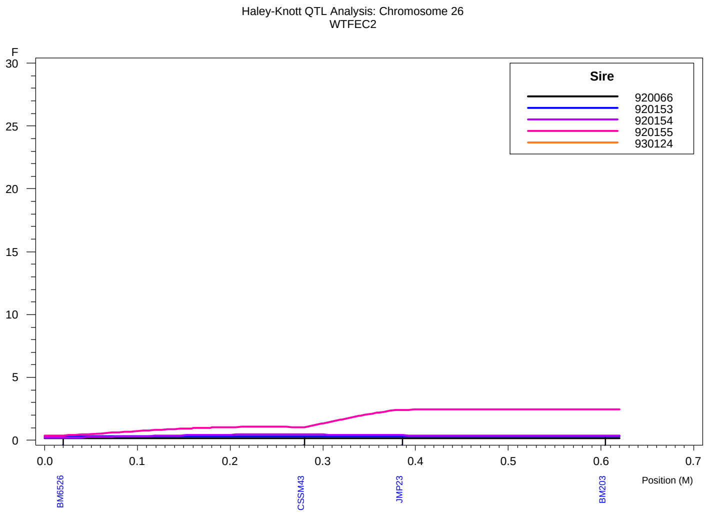
Haley-Knott QTL Analysis: Chromosome 26
WTFEC2
F
Sire
920066
920153
920154
920155
930124
Position (M)
BM6526
CSSM43
JMP23
BM203
